# Report from the 1st MYCOKEY International Conference Global Mycotoxin Reduction in the Food and Feed Chain Held in Ghent, Belgium, 11–14 September 2017

**DOI:** 10.3390/toxins9090276

**Published:** 2017-09-08

**Authors:** Sarah De Saeger, Antonio Logrieco

**Affiliations:** 1Department of Bioanalysis, Laboratory of Food Analysis, Faculty of Pharmaceutical Sciences, Ghent University, 9000 Ghent, Belgium; 2Institute of Sciences of Food Production, ISPA-CNR, Via G. Amendola, 122/O, I-70126 Bari, Italy; antonio.logrieco@ispa.cnr.it

## 1. Acknowledgment

This conference is organized within the framework of the **H2020**—**Research and Innovation Action**—Societal Challenge 2—“Food security, sustainable agriculture and forestry, marine, maritime and inland water research and the bioeconomy challenge”—GA 678781 **MycoKey** “Integrated and innovative key actions for mycotoxin management in the food and feed chain”.


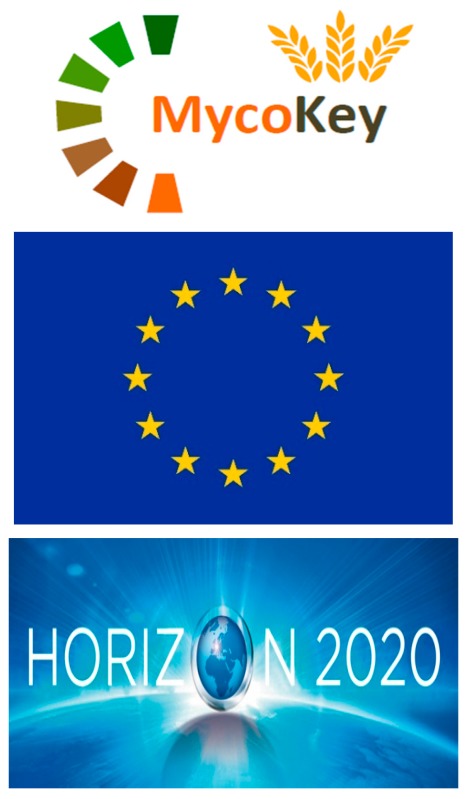


## 2. Preface

Mycotoxins play a significant role in food and feed safety. Legislative limits for a range of mycotoxins worldwide, as well as the presence of emerging mycotoxins, have resulted in an increased number of official controls deriving from national food safety plans and food trade purposes. The challenges in mycotoxin and toxigenic mould research are still enormous, due to the frequency, complexity and variability in their occurrence.

MycoKey (www.mycokey.eu) aims to deliver in 2019 the first integrated ICT tool to address mycotoxin contamination along the food and feed chain. MycoKey will integrate innovative key actions into a cheap and user-friendly application, able to provide real-time information and suggestions for mycotoxin management to several stakeholders. Thanks to the participation of several Chinese partners, this will strengthen both global knowledge on mycotoxins and effective cooperation with China.

Ghent is one of the most beautiful historic cities in Europe. It combines a mixture of past and present. From St Michael’s Bridge, there is a wonderful view of the skyline of Ghent, with the three impressive towers of St Nicholas’ Church, the Belfry and St Bavo’s cathedral, where the world-famous painting “The Adoration of the Mystic Lamb”, by Jan van Eyck, hangs. Much of the medieval city of Ghent has been preserved throughout the city. The old port (photo) with its guild halls on the Graslei and Korenlei is just one example of the beautiful views this city has to offer you. I welcome you all to the city of Ghent.

## 3. Organizing Committee

**De Saeger Sarah**, Ghent University, Ghent, Belgium

**Antonissen Gunther**, Ghent University, Merelbeke, Belgium

**Audenaert Kris**, Ghent University, Ghent, Belgium

**Catteuw Amelie**, Ghent University, Merelbeke, Belgium

**Cito Nunzia**, ISPA-CNR, Bari, Italy

**Croubels Siska**, Ghent University, Merelbeke, Belgium

**De Baere Siegrid**, Ghent University, Merelbeke, Belgium

**De Boevre Marthe**, Ghent University, Ghent, Belgium

**De Lobelle Annie**, Ghent University, Ghent, Belgium

**Devreese Mathias**, Ghent University, Merelbeke, Belgium

**Eeckhout Mia**, Ghent University, Ghent, Belgium

**Fathi Abdallah Mohamed**, Ghent University, Ghent, Belgium

**Boris Bekaert**, Ghent University, Ghent, Belgium

**Haesaert Geert**, Ghent University, Ghent, Belgium

**Logrieco Antonio**, ISPA-CNR, Bari, Italy

**Maene Peter**, Ghent University, Ghent, Belgium

**Rajkovic Andreja**, Ghent University, Ghent, Belgium

**Vandeputte Ellen**, Ghent University, Ghent, Belgium

## 4. Scientific Committee

**Audenaert Kris**, Ghent University, Ghent, Belgium

**Avantaggiato Giuseppina**, ISPA, Bari, Italy

**Battiliani Paola**, UCSC, Piacenza, Italy

**Bandyopadhyay Ranajit**, IITA, Oyo State, Nigeria

**Bhatnagar Deepak**, USDA-ARS, New Orleans, USA

**Chulze Sofia**, UNRC, Cordoba, Argentina

**Croubels Siska**, Ghent University, Merelbeke, Belgium

**De Saeger Sarah**, Ghent University, Ghent, Belgium

**Devreese Mathias**, Ghent University, Merelbeke, Belgium

**Eeckhout Mia**, Ghent University, Ghent, Belgium

**Haesaert Geert**, Ghent University, Ghent, Belgium

**Jie Feng**, CAAS, Beijing, China

**Karlovsky Petr**, Georg-August-University Goettingen, Goettingen, Germany

**Krska Rudolf**, IFA Tulln BOKU, Tulln, Austria

**Leslie John**, Kansas State University, Manhattan, USA

**Liao Yu-Cai**, Huazhong Agricultural University, Wuhan-Hubei, China

**Miller David**, Carleton University, Ottawa, Ontario, Canada

**Laitila Arja**, VTT Technical Research Centre, Espoo, Finland

**Logrieco Antonio**, ISPA-CNR, Bari, Italy

**Moretti Antonio**, ISPA-CNR, Bari, Italy

**Pascale Michelangelo**, ISPA-CNR, Bari, Italy

**Peiwu Li**, OCRI-CAAS, Wuhan-Hubei, China

**Rajkovic Andreja**, Ghent University, Ghent, Belgium

**Van der Lee Theo**, Wageningen UR, Wageningen, The Netherlands

**Verstraete Frans**, European Commission, Brussels, Belgium

**Vogelgsang Susanne**, Agroscope, Zurich, Switzerland

**Von Holst Christoph**, European Commission, Brussels, Belgium

**Waalwijk Cees**, Wageningen UR, Wageningen, The Netherlands

**Wanquan Chen**, CAAS, Beijing, China

**Wu Aibo**, INS-CAS, Shanghai, China

## 5. Plenary Session Lectures

### 5.1. Mycokey—A New Start of EU-China Cooperation on Mycotoxin Management

Jie F.

State Key Laboratory for Biology of Plant Diseases and Insect Pests, Institute of Plant Protection, Chinese Academy of Agriculture Sciences, Beijing, China; jfeng@ippcaas.cn

**Abstract:** Mycotoxins contamination is one of the most important problems worldwide in food and feed safety. In China, scientists started mycotoxin control research since the middle of last century. In the last decades, with the global warming and the change of cropping systems, toxigenic fungi like *Fusarium* spread to the north gradually, resulting in more and more severe mycotoxin contamination. Also as economic globalization develops further, mycotoxin caused bad influence on international food trade. Therefore, several national food safety plans were drew up including bilateral cooperation projects between Europe and China. Based on these collaboration, a number of achievements on mycotoxin control were made. However, the challenges in mycotoxin research are still enormous. Because mycotoxin management involved the whole food and feed chain but these projects just focus on a single subject respectively such as management in the field, plant breeding, mycotoxin detection and so on. Mycokey is a milestone for EU-China cooperation on mycotoxin which aims at developing smart, integrated, sustainable solutions and innovative tool kits to reduce the major mycotoxins in economically important food and feed chains. In this project, we established a strong multidisciplinary consortium composed by European and Chinese scientists for the first time. In Chinese part, eleven partners were involved in seven tasks including mycotoxin and disease monitoring and forecasting, population dynamics of toxigenic fungi, breeding for resistance, chemical control, biological control, mycotoxin detection techniques and detoxification of mycotoxin, which cover the whole food chain from field to consumer. All the tasks fully collaborated with European side. In the first year, all work has been carried out well. We believe we will make plentiful and substantial achievements in the next three years.

### 5.2. An Integrated ToolBox for Mycotoxin Management for Safer Food and Feed: The MyToolBox Approach

Krska R. ^1,^*, Poschmaier B. ^1^ and de Nijs M. ^2^

^1^ Center for Analytical Chemistry, Department of Agrobiotechnology, University of Natural Resources and Life Sciences, Vienna, Austria

^2^ RIKILT Wageningen University & Research, Wageningen, The Netherlands

* Correspondence: rudolf.krska@boku.ac.at

**Abstract:** Since March 2016, the EU project MyToolBox is working on an integrated approach to reduce moulds and mycotoxins along the whole food and feed chain. During its four years implementation period, the €5 million project will develop a series of integrated measures, to achieve significant reduction in losses of crops due to fungal and mycotoxin contamination. Due to its significant implications for food and feed safety, food security and international trade, the occurrence of fungal and, subsequently, mycotoxin contamination in various crops is of major concern. By mobilising a comprehensive multi-actor approach towards reducing risk due to mycotoxin contamination in crops all along the feed and food chains, MyToolBox involves pre-harvest interventions undertaken at farm level, together with post-harvest interventions undertaken from storage through to food processing into the finished food or feed product. About 40% of the consortium are industry partners, of which five partners are end-users from the farming community. While the majority of the elven consortium members originate from EU member states, three partners are from China, and others from Serbia, the Ukraine and Turkey, which shows a clear interest in promoting solutions beyond the European Union. Besides pursuing a field-to-fork approach, the MyToolBox project will also consider safe use options of mycotoxin contaminated batches such as microbial energy conversion to efficiently produce biofuels. Ensuring food & feed security and safety within a sustainable economic environment throughout the entire production chain is a major motivation behind MyToolBox. The mycotoxin commodity combinations that will be addressed are the most prevalent *Fusarium* mycotoxins (Deoxynivalenol, T-2/HT-2 toxins, zearalenone and fumonisins) in wheat, oats, maize and animal feed chains, ochratoxin A in wheat and aflatoxins in maize, peanuts and dried fruit (figs).

Depending on the commodity and the type of intervention, the MyToolBox consortium aims to achieve mycotoxin reductions of 20–90%. This will be assisted by developing information and decision support tools for each level of the chain, integrated into the ergonomic, secure web-based and mobile-friendly MyToolBox platform. This MyToolBox platform will guide the end-user to the most effective measure(s) to reduce biological contamination in crops, and will provide the necessary intelligence to ensure these measures take into account the prevailing conditions such as geographical location, meteorological conditions, land-use, crop management, storage and intended end use with relevance to specific crops. In cooperation with the Chinese partners, MyToolBox also aims to develop a sound scientific basis for standard-setting of authorisation of mycotoxin detoxifying feed additives in China, and consequently improve market access of relevant EU products.

### 5.3. MycoKey App: An ICT Solution to Facilitate Mitigation of Mycotoxin Risks

Van der Lee T.A.J. ^1^,*, Molendijk L.P.G. ^1^, Audenaert K. ^2^, landschoot S. ^2^, Verwaeren J. ^2^, Leggieri M.C. ^3^, Paola B. ^3^, Perrone G. ^4^, Logrieco A.F. ^4^ and Been T.H. ^1^

^1^ Wageningen University & Research, Wageningen, The Netherlands

^2^ Ghent University, Ghent, Belgium

^3^ Università Cattolica del Sacro Cuore, Piacenza, Italy

^4^ National Research Council, Institute of Sciences of Food Production, Bari, Italy

* Correspondence: theo.vanderlee@wur.nl

**Abstract:** The MycoKey app is developed as an ICT solution to facilitate mycotoxin risks mitigation by various stakeholders in the chain. Different work packages of MycoKey generate, validate and integrate knowledge that would provide useful information for risk assessment and would help to raise awareness, alert and specifically notify stake holders and provide options for mitigation of mycotoxin risks. This knowledge needs to be customized in order to effectively assist stakeholders. The MycoKey app, a mobile accessible platform, will deliver this customized information on a smartphone, tablet or computer. This app will generate a dashboard experience for accessing all relevant information for growers, advisors, grower associations, stakeholders in the production chain as well as policy-makers. It provides information on the risk of mycotoxins and, when required, will suggest management activities to mitigate and reduce risks. The app is user protected by a personal password and data can be private, shared with friends and advisors or anonymized and shared to other stakeholders. Governmental planners and policy makers will have access to shared, public databases and satellite data, as such biomass indices, land-use and mycotoxin risks can be estimated per region. The MycoKey app has different functionalities for smart phone (data entry and retrieval) and computer platforms (data entry and retrieval and analysis). Recalculation using different intervention strategies allows integration of management strategies in the risk model and calculations of “what if” scenarios. We hope to demonstrate the MycoKey app in Ghent for the first time!

This work is supported by the MycoKey-Project H2020 (E.U.3.2-678781).

## 6. Keynote Lectures

### 6.1. Climate Change, Fungal Pathogens, And Global Food Security

Bebber D.P. *, Chaloner T. and Fisher M.L.D.C

Department of Biosciences, University of Exeter, Stocker Road, Exeter EX44QD, UK

* Correspondence: d.bebber@exeter.ac.uk

**Abstract:** Fungal plant pathogens (FPPs) present a growing threat to food security and ecosystem management. The interactions between plants and their natural enemies are influenced by environmental conditions, and thus global warming and climate change could affect FPP ranges and impact. Observations of changing FPP distributions over the twentieth century suggest that growing agricultural production and trade have been most important in disseminating FPPs, but there is some evidence for a latitudinal bias in range shifts that indicates a global warming signal. Species distribution models using climatic variables as drivers suggest that ranges will shift attitudinally in the future.

We illustrate the potential impact of climate change on fungal pathogens with two examples affecting coffee, an important cash crop for developing countries. First, we test the hypothesis that climate change increased the likelihood of the 2008-11 outbreak of Coffee Leaf Rust (*Hemileia vastatrix*) in Colombia. Modelling the response of plant diseases to climate change is hampered by the difficulty of estimating pathogen-relevant microclimatic variables from standard meteorological data. The availability of increasingly sophisticated high-resolution climate reanalyses may help overcome this challenge. We develop a model of germination and infection risk, and drive this model using estimates of leaf wetness duration and canopy temperature from the Japanese 55-Year Reanalysis (JRA-55). We find no evidence for an overall trend in disease risk in coffee-growing regions of Colombia from 1990 to 2015, therefore we reject the climate change hypothesis. However, there was a significant elevation in predicted CLR infection risk from 2008–2011. A decrease in canopy surface water after 2011 may have helped terminate the outbreak. Second, we investigate the ecophysiology of mycotoxin-producing fungi from Brazilian coffee fruits, and model the potential growth rate of these fungi on arabica and canephora coffee species in 2012–2013, and under two climate change scenarios for 2070. Five fungal species were identified from coffee fruits and beans, including *Aspergillus flavus* and *Fusarium equiseti*. *Aspergillus flavus* grew at water activities of 0.93 and 0.89 but not at 0.80, and had the greatest growth at 25C. *Fusarium equiseti* only grew at a water activity of 0.93 and at 15C and 25C. Modelling of the potential mycotoxin production of these two species indicated similar geographical patterns for both but potentially greater susceptibility of arabica coffee in the future. Little is known of the ecology of contamination in coffee plantations. Given that the modelling suggests the most valuable coffee variety, arabica, could become more exposed to mycoxin contamination in Brazil as the climate continues to change, further research is needed to secure the future of the world’s favourite beverage.

### 6.2. The Socio-Economic Impact of Mycotoxin Contamination in Africa

Njobeh P. * and Ifeoluwa A.

Department of Biotechnology and Food Technology, University of Johannesburg, Johannesburg, South Africa

* Correspondence: pnjobeh@uj.ac.za

**Abstract:** Contamination of food and feed commodities accentuates a serious threat to food security worldwide, and central elements to these are microorganisms, specifically, fungi belong mainly to the *Aspergillus*, *Penicillium*, and *Fusarium* genera that produce mycotoxins. Mycotoxins are a diverse range of harmful secondary metabolites, first reported in early 1960s when aflatoxin marked as a killer agent from “Hell” was discovered as the cause of “Turkey X” disease in Great Britain. Subsequently, both aflatoxins and other mycotoxins have since became hazardous contaminants, found in about 25% of the global food and feed commodities worldwide. This situation is particularly more grievous in Africa with perfect temperature and relatively high humidity conditions suitable for fungal proliferation and attendant mycotoxin production, including the toxic aflatoxins, fumonisins, ochratoxins, deoxynivalenol and to an extent, zearalenone. This is further aggravated by unsafe food habits, and poor perceived understanding or awareness of the African population on issues that relate to fungal and mycotoxin contamination coupled to the inadequate regulatory mechanisms in place. Sequel to this prevailing situation are numerous reported profound adverse health and socio-economy consequences.

Since mycotoxins have properties of biological and chemical weapons, they have been used as chemical warfare agents with considerable evidence to suggest that some of them including aflatoxin and trichothecene mycotoxins have been included in some bioweapons programs. In Africa, the adverse socio-economic and health impact of mycotoxin contamination cannot be overemphasized. This includes reduced food availability that could result in famine or widespread malnutrition and ultimately food insecurity. It also brings about regulatory rejections, reduced market value of contaminated produce, forced use of alternative food sources, increased livestock and human diseases, as well as mortality in extreme cases. Moreover, this does not exclude the increased cost on research and regulatory activities aimed at reducing health risks associated with mycotoxin contamination and risk assessment. It is overwhelming to note that in Africa, an annual cost of over USD 750 million is accrued to aflatoxins contamination of crops alone, while the European Union regulation on aflatoxin B_1_ of 2 μg/kg would cost food exporters an annual loss of over USD 670 million. Following those reports, at the United Nations Conference in Brussels in 2001, the then United Nations Secretary General Kofi Annan lamented on such irrational losses to Africa accrued through these stringent mycotoxin regulations. Although these regulations cannot be subdued or eliminated, our ongoing study shows poor practice and understanding as well as low awareness of mycotoxins in Africa. This is coupled to the lack of efficient good manufacturing practices. To better limit the socio-economic impact of mycotoxins in Africa, there is a necessity for the appropriate authorities and relevant stakeholders to sensitize the populace on mycotoxins and ensure effective measures are in place to restrain the menace of these deadly naturally occurring toxins.

**Keywords:** fungi; mycotoxins; food safety; socio-economic; health and Africa

## 7. Oral Presentations

### 7.1. Distribution of Mycotoxin Biosynthetic Genes in 200 Fusarium Genomes

Proctor R.H. ^1^,*, Kim H.-S. ^1^, Vilani A. ^2^, Alexander N.J. ^1^, Brown D.W. ^1^, Busman M. ^1^, Cardoza R.E. ^3^, Amy K. ^1^, Lee T. ^4^, Lindo L. ^3^, Moretti A. ^2^, O’Donnell K. ^1^, Stanley A. ^1^, Vaughan M.M. ^1^, Susca A. ^2^, Gutiérrez S. ^2^ and McCormick S. ^1^

^1^ US Department of Agriculture, Agriculture Research Service, Peoria, IL, USA

^2^ Institute of Sciences of Food Production, National Research Council, Bari, Italy

^3^ Area of Microbiology, University School of Agricultural Engineers, University of León, Campus de Ponferrada, Ponferrada, Spain

^4^ Microbial Safety Team, National Institute of Agricultural Sciences, Rural Development Administration, Wanju, Korea

* Correspondence: robertproctor@ars.usda.gov

**Abstract:**
*Fusarium* is a species-rich genus of fungi that causes disease on most crop plants and produces diverse secondary metabolites (SMs), including some of the mycotoxins of greatest concern to food and feed safety. To determine the potential SM diversity within *Fusarium* as well as the distribution and evolution of mycotoxin biosynthetic gene clusters, we have assessed the presence and absence of SM biosynthetic gene clusters in genome sequences of >200 isolates representing 22 multi-species lineages (species complexes) and four single-species lineages of *Fusarium*. The results indicate that collectively *Fusarium* species have the genetic potential to produce hundreds of structurally distinct families of SMs, but that there is tremendous variation in distribution of SM clusters among species and lineages. Some clusters occur in one or a few lineages, while others occur in most lineages. Also, some SM clusters occur in most or all species within lineages, while others occur more sporadically in fewer species. Phylogenetic analyses suggest that differences in distribution have resulted from variation in vertical inheritance, horizontal transfer, and loss of clusters during the evolutionary diversification of *Fusarium*.

Functional characterization of selected biosynthetic genes in *Fusarium* and other fungi indicate that variability in production of analogs of the same mycotoxin family can arise through acquisition, loss, and changes in functions of genes in the corresponding gene cluster. These findings add to a growing body of evidence that qualitative differences in mycotoxin production is affected by variation in presence and absence of clusters as well as variation in content and functions of genes within clusters.

### 7.2. Insight into Genome Variability in the Fusarium Incarnatum-Equiseti Species Complex through Comparative Analysis of Secondary Metabolic Biosynthetic Gene Clusters

Villani A. ^1,^*, Kim H.-S. ^2^, Proctor R.H. ^2^, Brown D.W. ^2^, De Saeger S. ^3^, Logrieco A.F. ^1^, Moretti A. ^1^ and Susca A. ^1^

^1^ Institute of Sciences of Food Production, CNR, Via Amendola, 122/O, 70126 Bari, Italy

^2^ National Center for Agricultural Utilization Research, U.S. Department of Agriculture, 1815 N. University St., Peoria, IL 61604, USA

^3^ Faculty of Pharmaceutical Sciences, Laboratory of Food Analysis, Ghent University, Ghent, Belgium

* Correspondence: alessandra.villani@ispa.cnr.it

**Abstract:** The genus *Fusarium* comprises 22 species complexes that together include approximately 300 phylogenetically distinct species. A major focus in *Fusarium* literature is to understand the genetic basis of niche specialization, secondary metabolites (SM) production, and host interactions in closely related species. In this study, we focused on the *Fusarium incarnatum-equiseti* species complex (FIESC), which comprises 31 phylogenetically distinct species. Members of FIESC are regarded as important plant and human pathogens, able to produce a wide range of mycotoxins, including both type A and B trichothecenes, beauvericin, butenolide, equisetin, and zearalenone. Although their wide occurrence and toxigenic potential, little is known about inter species genome variability or the molecular organization and distribution of the SM gene clusters within the complex. Therefore, we investigated the distribution and variability of known and putative novel SM biosynthetic gene clusters within FIESC, by generating and analyzing genomes of twelve members of the complex. Phylogenomic analysis, based on 26 housekeeping and whole-genome sequences, inferred phylogenies that were consistent with but more highly resolved than previously described phylogenies inferred from four genes. Genome mining of SM biosynthetic gene clusters through antiSMASH analysis showed that about 3–4% of predicted genes in a genome are involved in secondary metabolism. We confirmed production of several SMs product of several gene clusters by chemical analysis. The trichothecene cluster was present in all twelve genomes, but differed in presence, absence and arrangement of genes relative to the cluster in the *F. sambucinum* species complex. Overall, the number of PKS and NRPS was comparable to that of other *Fusarium* species. Specifically, eight well-characterized SM clusters, four unknown PKS and nine unknown NRPS are shared among all the twelve genomes, while 20 other SM biosynthetic clusters are variably distributed among several genomes investigated. Interestingly, one NRPS-derived SM gene cluster and three PKS-derived SM gene clusters are uniquely present in FIESC among all the *Fusarium* species investigated to date, representing new potential SM to further investigate. Overall, our results indicate considerable variation exists in the genetic potential of FIESC members to produce SMs, including mycotoxins. The results also reveal genetic variation within FIESC that is associated with previously observed phenotypic variability.

This research was supported by Horizon 2020 project Mycokey (GAN. 678781).

### 7.3. The Life Cycle of Fusarium in Different Crop Rotation Systems in China

Zhang H.

Institute of Plant Protection, Chinese Academy of Agricultural Sciences, Beijing, China; zhanghao@caas.cn

**Abstract:** Wheat-maize and wheat-rice rotation within one year are the main crop rotation systems in Northern and Southern China respectively. *Fusarium* can infect all the three cereals, leading to yield loss and mycotoxin contamination. Therefore the knowledge of life cycle of *Fusarium* in these areas is important for IPM of the disease. In recent years, we did a large-scale population analysis of *Fusarium* populations on wheat, maize and rice in the main grain-producing areas. More than 2000 *Fusarium* isolates were collected and the species and chemotype were identified. The maize and wheat population showed much higher diversity than wheat at species level. *F. graminearum* with 15ADON chemotype is the first and third largest population on wheat and maize respectively, indicating it can circulate between the two hosts in Northern China. *F. boothii* is the fourth largest population on maize, but absent on wheat. Virulence tests with *F. boothii* and *F. graminearum* showed adaptation to the host. In Southern China, *F. asiaticum* is predominant on both wheat and rice stubble, indicating rice stubble is the main media for their overwintering. DON producing *F. asiaticum* decreased from east to west and NIV was the main chemotype in southwest region. We also found significant more NIV producers on rice than wheat in the same area, which revealed the rice preference of NIV population. In agreement with this, predominant NIV chemotype were also identified in Fujian Province, where rice is the main crop and there is almost no wheat growing.

### 7.4. Genome-Wide Association Mapping of Fusarium Head Blight Resistance to Mycotoxin Accumulation and to Spread in Wheat

Wu L., Zhang Y., He Y., Jiang P., Ma H. and Zhang X. *

Institute of Food Crops, Jiangsu Academy of Agricultural Sciences, Nanjing, China

* Correspondence: xuzhang@jaas.ac.cn

**Abstract:** Fusarium head blight (FHB) that caused by *Fusarium graminearum* is a devastating disease of wheat worldwide. The contamination of the infected grain with mycotoxins such as deoxynivalenol (DON) poses a significant threat to the health of humans and livestock. A set of 223 commercial wheat cultivars from different regions in China was phenotyped for disease rate of spikelets (Type II resistance) and DON accumulation in grains after harvest (Type III resistance). 90 k wheat chip array was used to identify 16,335 single-nucleotide polymorphisms (SNPs) covering all 21 wheat chromosomes. Genetic loci associated with Type II and III resistance were evaluated by genome-wide association study (GWAS) using a compressed mixed linear model (cMLM). Three chromosome regions significantly associated with Type II resistance were detected on chromosomes 5A, 2B, and 3B in two environment tests, while chromosome regions on chromosomes 2A, 4A and 2B were associated with Type III resistance. It is assumed that the same locus harboring SNP clusters on chromosome 2B is associated with both Type II and Type III resistance. These SNPs could have the potential to be used for wheat breeding for FHB Type II and Type III resistance and for functional validation of candidate genes governing FHB resistance.

### 7.5. Contribution Of Mitochondria and Peroxisomes on the Mycotoxin Biosynthesis and Fungal Virulence Via Distinct Regulatory Pathways in Fusarium Graminearum

Chen Y., Zheng S., Ju Z. and Ma Z. *

Institute of Biotechnology, Zhejiang University, Hangzhou 310058, China

* Correspondence: zhma@zju.edu.cn

**Abstract:**
*Fusarium graminearum* is the predominant pathogenic fungus that cause *Fusarium* head blight (FHB) disease in wheat, barley and other cereals around the world. FHB can result in severe direct yield losses. In addition, infested grains are contaminated with mycotoxins, such as deoxynivalenol (DON) and zearalenones, and harmful to humans. The understanding of *F. graminearum* mycotoxin biosynthesis and pathogenesis process is vital to effective disease and mycotoxins management. At the beginning of study, we observed the mitochondral and peroxisomal proliferation was highly stimulated during the mycotoxin biosynthesis and plant infection process of *F. graminearum*. Currently, regulatory mechanisms of mitochondria and peroxisomes in these cellular processes were poorly understood. Here, we investigated the biological functions of mitochondrial membrane protein FgLetm1 and peroxisomal docking complex to uncover roles of these two organelles in mycotoxins synthesis and virulence. Results indicated that the disruption of mitochondrial FgLETM1 significantly reduced the concentration of endogenous ROS, reduced the TRI genes expression, decreased DON biosynthesis and attenuated virulence of *F. graminearum in planta*. Meanwhile, dysfunctional peroxisomes resulted in the shortage of acetyl-CoA, the precursor of trichothecenes, and subsequently decreased the mycotoxins production. Deletion mutants of peroxisomal docking peroxins resulted in the increment of sensitivity towards host oxidative burst and CWI stress agents, and subsequently decreased the survival and virulence during the fungus-host interaction. Collectively, this is the first report to demonstrate that the integrity of mitochondria and peroxisomes are important for DON biosynthesis and virulence in *Fusarium* species.

### 7.6. Specialized Metabolite Clusters in Fusarium

Waalwijk C. ^1,^*, Hoogendoorn K. ^1,2^, van der Lee T.A.J. ^1^ and Medema M.H. ^2^

^1^ Biointeractions & Plant Health, Wageningen Plant Research, Wageningen, The Netherlands

^2^ Bioinformatics Group, Wageningen University, Wageningen, The Netherlands

* Correspondence: cees.waalwijk@wur.nl

**Abstract:** The genomes of many toxigenic fungi that have recently become available provide an unique opportunity to discover potentially new specialized metabolites (SMs), including mycotoxins. The genes involved in their production are usually clustered in biosynthetic gene clusters (BGCs) and current estimates of the number of BGCs in various *Fusarium* species largely exceed the number of known SMs produced by these fungi. A combination of bioinformatic tools was applied to identify BGCs across annotated *Fusarium* genomes and they were grouped into annotated families. BGCs are mostly located in so-called non-conserved regions that are typically exhibiting high variation and low expression. Comparative analyses allowed the study of gain/loss events of BGCs throughout the history of the *Fusarium* genus. The results raise several new hypotheses on how gene clusters evolve and how they may contribute to pathogenicity or to differences in the host specificity observed between *Fusarium* species.

This systematic overview of biosynthetic diversity in *Fusarium* paves the way for targeted natural product discovery based on automated identification of species-specific pathways as well as on the connection of species ecology.

### 7.7. Diving into Guinean Gulf Aspergillus Section Flavi Diversity: Description of a New Aflatoxin Producing Species

Carvajal-Campos A. ^1^, Tadrist S. ^1^, Manizan A.L. ^2^, Akaki D.K. ^3^, Bailly S. ^1^, Oswald I. ^1^, Lorber S. ^1^, Brabet C. ^4^ and Puel O. ^1,^*

^1^ Toxalim (Research Centre in Food Toxicology), Université de Toulouse, INRA, ENVT, INP-Purpan, UPS, Toulouse, France

^2^ Université Nangui Abrogoua, Abidjan, Côte d’Ivoire

^3^ INP-HB (Institut National Polytechnique Félix Houphouët-Boigny), Yamoussoukro, Côte d’Ivoire

^4^ Centre de Coopération Internationale en Recherche Agronomique pour le Développement (CIRAD)—Département PERSYST—UMR QualiSud, Montpellier, France

* Corresponding author: olivier.puel@inra.fr

**Abstract:**
*Aspergillus* section *Flavi* is a well-studied group of molds including several species characterized as aflatoxin producers. Aflatoxins, especially aflatoxin B1 (AFB1), are considered as mycotoxins of high health risk due to their carcinogenic, mutagenic and teratogenic potential (IARC 2012). Nevertheless, species in this section are capable of producing some other mycotoxins, such as cyclopiazonic acid, aflatrem.

Water and temperature are key parameters for the fungus growth and AFB1 production and, due to physiological properties of aflatoxigenic fungi, it was usually admitted that species belonging to *Aspergillus* section *Flavi* are very current fungi in regions where hot climate favours their development. In tropical and subtropical countries, they grow principally in feed and food commodities, like cottonseeds, spices, peanuts, nuts, maize being the most favorable substratum. Over the last two decades, species composition of the section *Flav*i has experienced several modifications. Currently, this section is composed by 26 described species confirmed by a polyphasic approach that includes morphological, phylogenetic and secondary metabolites profile characterizations. However, identification of new species continues to be a main issue when these types of molds are found in commodities. For instance, an underestimation of their diversity could lead to an improper risk assessment and increase ingestion of mycotoxins by humans or animals.

Based on the polyphasic approach evidence, herein we present a new aflatoxigenic species belonging to *A. Flavus* clade. The description is based on fourth strains isolated from peanuts and peanut paste harvested in Ivory Coast. This new species is able to produce aflatoxins B and G. For the phylogenetic characterization, we used a concatenated database of nine genes (*ITS*, *calmodulin*, *β-tubulin*, *ppgA*, *PreA*, *PreB*, *mcm7*, *RPBH*, *AflF*). In addition, the determination of the mating type showed that all strains are heterothallic with three strains assigned to *MAT1.1* and one strain to *MAT1.2*. Morphological characterization of the new strains was performed on MEA, CYA, AFPA, CREA media at 25 °C for seven days. Analysis of secondary metabolites was performed by LC/MS.

Taken together, results suggest that this new species belonging to the section Flavi is closest to *Aspergillus parvisclerotigenus*, a species which is frequently isolated in western Africa (Benin, Nigeria).

### 7.8. Investigating the Alternaria Disease Progression and Species Composition on Potato in Belgium

Vandecasteele M. ^1,^*, Landschoot S. ^1^, Höfte M. ^2^, De Saeger S. ^3^, Audenaert K. ^1^ and Haesaert G. ^1^

^1^ Department of Applied Bioscience Engineering, Faculty of Bioscience Engineering, Ghent University, Valentin Vaerwyckweg 1, 9000 Ghent, Belgium

^2^ Department of Crop Protection, Faculty of Bioscience Engineering, Ghent University, Coupure Links 653, 9000 Ghent, Belgium

^3^ Department of Bioanalysis, Faculty of Pharmacological Sciences, Ghent University, Ghent, Belgium

* Correspondence: Michiel.Vandecasteele@UGent.be

**Abstract:**
*Alternaria* species are a serious threat for potato cultivation since heavy infections can lead to significant yield and quality losses. The infection causes necrotic symptoms, which cannot be visually distinguished. Over the past years, *Alternaria* on potato has become increasingly important in NW Europe. Although the exact cause for this emerging problem remains elusive, it might be attributed to the combined effect of climate change, a reduced use of the fungicide mancozeb, the increased specificity of active ingredients to control *Phytophtora infestans* and the production of high-yielding susceptible cultivars. Furthermore, little is known about the Belgian *Alternaria* population composition and the contribution of both *A. solani* and *A. alternata* to the disease. The main goal of this research is to identify the primary causal agents of *Alternaria* disease on potato, to determine inter- and intraspecific diversity within the *Alternaria* population in Flanders, and to unravel the complex interaction between stress-related hormones and the *Alternaria* infection. To achieve these objectives, 22 locations were monitored throughout Flanders during the growing seasons of 2014 and 2015. Results of this disease survey unequivocally show that the disease incidence and—pressure for both seasons was low and that crops grown in sandy soils appear to be more prone to *Alternaria* disease. In a second part, we identified the population composition at the species level on different time points during the growing season. Therefore, infected leaf samples were collected from the field and using a microscopic and molecular approach, we concluded not only that *A. arborescrens* is the most abundant species present on infected leafs, but also that small-spored species like *A. alternata* and *A. arborescens* rather than the large-spored *A. solani*, were predominant at the beginning of the growing season. The disease escalated only when *A. solani* species were accumulating. Indeed, based on high-throughput virulence assays, we observed that *A. solani* was much more virulent than *A. alternata* or *A. arborescens*. Next, a subset of isolates will be used to investigate the complex interaction between host-specific toxins and stress-related hormones such as ethylene or auxins during the infection process. Indeed, previous research shows that ethylene is a key component in upstream signaling of Programmed Cell Death induced by host-specific AAL-toxin in tomato.

### 7.9. Recent Developments in Rapid Multi-Mycotoxin Detection

Peiwu Li ^1,2,3,4,5,^*, Zhaowei Zhang ^1,2,3^, Fei Ma ^1,2,3^ and Qi Zhang ^1,2,3,^*

^1^ Oil Crops Research Institute of the Chinese Academy of Agricultural Sciences, Wuhan 430062, China

^2^ Key Laboratory of Biology and Genetic Improvement of Oil Crops, Ministry of Agriculture, Wuhan 430062, China

^3^ Key Laboratory of Detection for Mycotoxins, Ministry of Agriculture, Wuhan 430062, China

^4^ Laboratory of Risk Assessment for Oilseeds Products (Wuhan), Ministry of Agriculture, Wuhan 430062, China

^5^ Quality Inspection and Test Center for Oilseeds Products, Ministry of Agriculture, Wuhan 430062, China

* Correspondence: peiwuli@oilcrops.cn (P.L.); zhangqi01@caas.cn (Q.Z.)

**Abstract:** Mycotoxin detection is crucially important to avoid mycotoxin contamination in agro-foods. Single mycotoxin detection has been developed in the past years, however, co-occurrence of mycotoxins in major agro-foods is a frequent phenomenon. Therefore, rapid detection for multi-mycotoxins in agro-foods are urgently required. This presentation reviews the recent developments in rapid multi-mycotoxin detection.

Firstly, series of monoclonal & recombinant antibodies, nanobodies and generic antibodies against mycotoxins were successfully developed as the key recognition reagents with boasted high sensitivity and specificity. Secondly, rapid multi-mycotoxin detection methods were developed where series of rapid dipstick methods & kits were investigated from single to multi-mycotoxin characterized by a high specificity, sensitivity and high throughput. Thirdly, the confirmatory detection for multi-mycotoxins was studied by using (high) resolution LC/MS-MS. Via stable isotopic dilution and immunoaffinity column, the non-targeted screening of multi-mycotoxins was conducted by using HPLC- or UPLC-MS/MS, confirmatively. The multi-level mass spectrometric library and electronic labeling system was established, covering most biotoxins, pesticides and their metabolites, regulated by many countries.

Nowadays, the agro-food safety in developing countries is hampered by an underdeveloped economy, small-scale and scattered production modes and locations at mycotoxin contaminated-prone areas. The request of multi-mycotoxin detection and control from farm to table is urgent. To meet the demand of the multi-mycotoxin detection in developing countries, it is suggested that the rapid detection of multi-mycotoxins in agro-foods is focused on the principle of 5S (speed, sensitivity, specificity, simultaneousness, and small-sized reader).

### 7.10. Novel Immunoassay Format for Rapid Screening of Mycotoxins—HT-2 Toxin Detection as an Example

Nevanen T.K. *, Arola H., Laitila A. and Tullila A.

VTT Technical Research Centre of Finland, Espoo, Finland

* Correspondence: tarja.nevanen@vtt.fi

**Abstract:** Traditionally the fast diagnostics of mycotoxins have been carried out using ELISA or lateral flow immunoassays with poly- or monoclonal antibodies. These assays are based on indirect competitive detection. We have developed a novel non-competitive immunoassay format, called immune complex assay, that is based on recombinant antibody fragments. Immune complex assay for HT-2 toxin utilizes the primary antibody binding to HT-2 toxin and the immunocomplex antibody recognizing the primary antibody—HT-2 toxin complex. Immune complex assays are simple to perform and have positive readouts. In addition they provide specificity and sensitivity to ELISA and homogeneous assays.

As an example, the performance of the immune complex assay for HT-2 toxin is presented here. Recombinant antibody fragments were isolated in vitro from the antibody gene libraries, produced in *E. coli* bacteria and purified by metal affinity chromatography. For the homogeneous immune complex assay both antibodies were labelled with FRET compatible fluorescent dyes. For the rapid ELISA the immune complex antibody was genetically fused with the alkaline phosphatase-enzyme.

The primary antibody has 100% cross-reactivity for HT-2 and T2-toxins. However, the immune complex assay is specific to the HT-2 toxin only. The assay performance with real sample matrix was evaluated in case of homogeneous FRET assay using spiked wheat extract. The linear range of the FRET assay was 25–400 μg/kg for HT-2 toxin. The LOD and LOQ values were 19 μg/kg and 55 μg/kg, respectively. One-step homogeneous non-competitive immune complex assay is simple when antibody reagents are pre-dried in a vial: just add sample and measure in 10 min.

Immunocomplex antibody-enzyme fusion protein can be used as a single detection molecule in ELISA and thus the number of incubation and washing steps can be reduced. Three naturally contaminated grain matrices were used to evaluate the assay. Limits of detection values were 13 μg/kg, 4 μg/kg and 16 μg/kg for wheat, barley and oat, respectively.

The immune complex assay can be applied to various immunoassay formats including ELISA, lateral flow assay and sensors. ELISA assays can be miniaturized and multiplexed to serve better the mycotoxin screening purposes.

### 7.11. Real Time Electrochemical Profiling Assay Method for Detection of Aflatoxin B1 in Dried Fig

Ozer H. ^1,^*, Basegmez H.I.O. ^1^, Uludag Y. ^2^, Esen E. ^2^ and Muhammad T. ^2,3^

^1^ Food Institute—MRC—The Scientific and Technological Research Council of Turkey (TUBITAK), Kocaeli, Turkey

^2^ Bioelectronic Devices and Systems Group, UEKAE—BILGEM—The Scientific and Technological Research Council of Turkey (TUBITAK), Kocaeli, Turkey

^3^ College of Chemistry & Chemical Engineering, Xinjiang University, Xinjiang Key Laboratory of Oil and Gas Fine Chemicals, Urumqi, China

* Correspondence: hayrettin.ozer@tubitak.gov.tr

**Abstract:** We introduce a new biosensor platform (MiSens) for the detection of aflatoxin B1 (AFB1) based on real time electrochemical profiling (REP^TM^) assay method coupled with the use of natural and artificial antibodies (polymers) as affinity ligands. Sample clean-up was performed with aflatoxin-targeted polymer and the samples were then analysed using natural antibody-based MiSens biosensor assay. The new biosensor allows real-time and on-site detection of AFB1 in dried figs with a rapid, sensitive, fully automated and miniaturized system and expected to have an immense economic impact for food industry.

In this work AF standards and contaminated food matrix (dried fig) were used and the REP^TM^ platform (MiSens biosensor) were compared with the reference methods. With this aim, MiSens system was utilized after extraction and the results were compared with HPLC with immunoaffinty cleanup.

The results clearly indicate that the aflatoxin detection performance of MiSens assay is comparable to immunoaffinity column coupled HPLC results; hence, can be utilized as an alternative on-site aflatoxin detection method. As a conclusion; The new biosensor allows on-time detection of Aflatoxin B1 in real samples with a rapid, sensitive, fully automated and miniaturized system and will have an immense economic impact for food industry.

The project has received funding from BILGEM—TUBITAK (The Scientific and Technological Research Council of Turkey) (grant No.: 100121), the Republic of Turkey Ministry of Development Infra-structure Grant (No.: 2011K120020), TUBITAK BIDEB 2221 and the European Union’s Horizon 2020 research and innovation programme under grant agreement No. 678781.

### 7.12. Biotechnological Synthesis Of Deoxynivalenol-3-Glucoside and Its Fully Labeled ^13^c Analogue with Heterologously Expressed Enzymes and Their Analytical Application in a Stable Isotope Dilution Assay

Kuster L. ^1,^*, Berthiller F. ^2^, Michlmayr H. ^3^, Malachova A. ^2^, Fiby I. ^2^ and Adam G. ^3^

^1^ Romer Labs Holding GmbH, Getzersdorf, Austria

^2^ Center for Analytical Chemistry, IFA-Tulln, BOKU, Tulln, Austria

^3^ Department of Applied Genetics and Cell Biology, BOKU, Tulln, Austria

* Correspondence: lilian.kuster@romerlabs.com

**Abstract:** The formation of masked mycotoxins is a major detoxification strategy of crops. Usually, a glucose molecule or a sulfate is conjugated to the mycotoxin. Although these masked toxins do not further harm the plant, their toxicity to humans and animals might re-emerge with the cleavage of the added masking molecule in the gastrointestinal tract of mammals during digestion. Toxicological data on masked mycotoxins are scarce, and current results and knowledge on the real risks and effects of these compounds are insufficient.

Deoxynivalenol-3-glucoside (D3G) is known as the most important masked metabolite of deoxynivalenol (DON) and can be found mainly in wheat, maize and barley. High levels of D3G have already been described in cereals and barley malt and therefore as well in beer samples. As this toxin is not synthesized by *Fusarium* itself, the analytical reference material cannot be produced by a simple fermentation.

Usually D3G was obtained by isolation from infected plants, a rather tedious procedure leading to low yields and co-isolation of numerous undesired compounds from the plant. To improve the production of D3G for the use as analytical standard we followed a completely new strategy. A heterologously expressed enzyme, a glycosyltransferase from rice (OsUGT79), was used for the conjugation of the glucose molecule to DON. In our two step approach UDP-glucose was first synthesized by applying a heterologously expressed sucrose synthase (AtSUS1) from *Arabidopsis thaliana* using sucrose and UDP as substrate and in the second step linked to DON via the glycosyltransferase.

With this novel procedure it is now possible to obtain very high yields of D3G and also of fully ^13^C isotope labeled D3G, by using ^13^C labeled deoxynivalenol and ^13^C sucrose for synthesis.

^13^C-D3G and ^13^C-DON was then used to develop a stable isotope dilution assay for the accurate determination of DON and D3G in cereals. After extraction, a mixture of both internal standards was spiked into the cereal extract, which was consecutively measured by LC-MS/MS. DON and D3G could easily be separated under reversed phase conditions, while the internal standards showed perfect co-elution with the respective mycotoxins. Matrix effects in wheat and maize were effectively compensated using this internal calibration.

The above described developments show a completely new biotechnological approach for producing analytical standards and subsequently allow the highly accurate determination of contamination levels with D3G and DON in cereals for a proper risk assessment.

### 7.13. LC-MS Based Multi-Mycotoxin Methods—Are They Applicable to Complex Feed Matrices?

Sulyok M. *, Steiner D. and Krska R.

Department IFA-Tustrilln, University of Natural Resources and Life Sciences Vienna (BOKU), Konrad Lorenzstr. 20, A-3430 Tulln, Austria

* Correspondence: michael.sulyok@boku.ac.at

**Abstract:** Analytical methods based on liquid chromatography coupled to tandem mass spectrometry have become state of the art for the analysis of mycotoxins in the past few years. This is especially true for well-defined grain-, nut- or dried fruit-based matrices as it has been shown by numerous groups that matrix effects are under control using matrix-matched calibration or isotopically labelled internal standards.

Animal feed may be a complex mixture of grains and numerous ingredients that have not been addressed by many analytical methods so far (e.g., soy, dried distillers grain soluble, grass or grain silages, bone meal, etc.). As the exact composition exhibits significant variations between individual samples of a given feed type, it might be expected that relative matrix effects as well as variations in extraction efficiency negatively influence the method performance. As a consequence, the use of isotopically labelled standards or standard addition of each sample might be mandatory. Based on data deriving from spiking experiments of both the final feed formulation as well as the individual ingredients this presentation aims to discuss the applicability of the LC-MS/MS based dilute and shoot for quantitative analysis of animal feed.

### 7.14. Liquid Chromatography—High Resolution Mass Spectrometry Screening of Fusarium Toxins in Wheat: Evaluation of Analytcal Performances through in House Validation and Small Scale Collaborative Study

Ciasca B. ^1^, Pascale M. ^1^, Altieri V.G. ^2^, Longobardi F. ^2^, Catellani D. ^3^, Suman M. ^3^ and Lattanzio V.M.T. ^1,^*

^1^ Institute of Sciences of Food Production, National Research Council of Italy, Bari, Italy

^2^ Department of Chemistry, University “Aldo Moro” of Bari, Bari, Italy

^3^ Barilla SpA, Advanced Laboratory Research, Parma, Italy

* Correspondence: veronica.lattanzio@ispa.cnr.it

**Abstract:** A strong trend toward using highly selective high-resolution mass spectrometry technologies for rapid screening of multiple mycotoxins has been observed in recent years. Consequently, within the European Union, specific performance requirements for screening methods for mycotoxins have been issued in the Regulation 519/2014/EU that apply not only to bioanalytical methods based on immuno-recognition, but also to mass spectrometry approaches. This regulation establishes that the “aim of the validation is to demonstrate the fitness for purpose of the screening method”, and focuses the entire validation procedure on the determination of cut-off value, false negative and false suspect rates. However, performance characteristics such as sensitivity, selectivity, and precision are embedded in these parameters. In the present study, a liquid chromatography high-resolution mass spectrometry method was developed for the simultaneous determination of the major *Fusarium* toxins in wheat, namely deoxynivalenol, 3- and 15-acetyl deoxynivalenol, T-2 and HT-2 toxins, zearalenone, enniatins A, A_1_, B, B_1_, and beauvericin. A sample preparation protocol was optimized, based on a double extraction (methanol followed by acetonitrile/water mixture), and purification through solid phase extraction C18 columns, achieving satisfactory recoveries for the target mycotoxins covering a wide range of polarities. Analytical performances of the developed method were evaluated through both in house validation and small scale interlaboratory study. The validation study, designed according to the Commission Regulation 519/2014/EU, was performed at screening target concentrations (STC) close to EU maximum permitted or indicative levels. The in house validation procedure provided the precision of the response under repeatability conditions and the intermediate precision (the latter resulting lower than 42% for all mycotoxins), the matrix effect from different wheat varieties on the precision, the cut off value, and the rate of false positive results for samples containing the target mycotoxins at 20% of the STC, that resulted lower than 1% in all cases. A small scale collaborative study was therefore carried out to determine reproducibility and robust and laboratory independent cut off values. Finally, analysis of naturally contaminated samples and reference materials confirmed method suitability for screening of *Fusarium* mycotoxins in wheat, to check compliance with EU maximum permitted or recommended levels.

This work has been supported by the Italian Ministry of Education, University and Research, MIUR, SAFE&SMART project “New enabling technologies for food safety and food chain integrity within a global scenario”.

### 7.15. Secondary Metabolite Profiling of Food-Borne Alternaria

Cabral L.D.C. ^1^, Pavicich M.A. ^1^, Andersen B. ^2^, Nielsen K.F. ^2^ and Patriarca A. ^1,^*

^1^ Laboratorio de Microbiología de Alimentos/Departamento de Química Orgánica/Facultad de Ciencias Exactas y Naturales, Universidad de Buenos Aires/Buenos Aires, Argentina

^2^ Section for Microbial ecology and physiology/DTU Bioengineering/Technical University of Denmark/Kgs. Lyngby, Denmark

* Correspondence: andreap@qo.fcen.uba.ar

**Abstract:** The genus *Alternaria* includes both plant pathogenic and saprophytic species, which can affect crops in the field or cause post-harvest spoilage of plant fruits and kernels. *Alternaria* species have the ability to produce a wide variety of secondary metabolites, which play important roles in food safety. Alternariol (AOH), alternariol monomethylether (AME), tenuazonic acid (TA), altenuene (ALT), tentoxin (TEN) and altertoxins (ATXs) are the main compounds thought to pose a risk to human health. However, food-borne *Alternaria* species are able to produce many more metabolites, whose toxicity has been tested incompletely or not tested at all. To understand the full chemical potential of food associated *Alternaria* spp., and their distribution on crops and food products, the secondary metabolite profiles of *Alternaria* strains isolated from food substrates were analysed.

A total of 360 strains isolated from tomato (107), pepper (64), wheat (94), blueberry (44), apple (45), and walnut (6) were analysed. The morphological characterization was made according to Simmons (2007), based on the three-dimensional sporulation pattern, microscopic morphology, and colony characteristics. The metabolite profiling was done on DRYES (14 days, 25 °C) by micro-extraction with ethyl acetate-1% formic acid (Andersen et al., 2015). Detection was performed by UHPLC-HRMS. MS was performed in ESI^+^ and ESI^−^ in the scan range *m*/*z* 100–1000, with a mass accuracy <1.5 ppm. Data processing involved an aggressive dereplication approach, matching *Alternaria* and related genera compounds with data based on accurate mass and isotopic pattern, and UV/Vis data. MS/HRMS were further conducted for matching fragmentations with the molecular structure.

Through morphological identification the strains were classified into the species-groups (sp.-grp.) *A. alternata* (7), *A. arborescens* (39), *A. tenuissima* (261), and *A. infectoria* (19). The remaining 34 strains could not be assigned to a specific sp.-grp. and were referred to as *Alternaria* sp. All sp.-grp. were distributed among the different food products except for *A. infectoria*, which was only isolated from wheat. A total of 45 known secondary metabolites produced by *Alternaria* spp. from food were detected. Additionally, 44 compounds of unknown structure were produced by these strains. The metabolite profiles overlapped among the food species, except for strains belonging to the *A. infectoria* sp.-grp., which had no metabolites in common with the other strains. AOH and AME were the compounds produced by most of the strains (283 and 285, respectively), followed by Pyrenochaetic acid A (269), ALT (248), Altechromone A (247), TEN (224), TA (209) and its isopropyl derivative (186), ATX-I (159), and Altersetin (145). More than 100 strains were also able to produce alterperylenol, 3-Hydroxyalternariol 5-*O*-methyl ether, ATX-II, altenuisol, altenusin, and dihydrotentoxin. These metabolites were produced by strains from all food products analysed. Toxicological data on these compounds would be necessary to design regulatory guidelines on *Alternaria* metabolites in food products.

### 7.16. Ochratoxin a in Rye—A Challenge for Industry and Analytics

Reichel M. ^1,2,^*, Staiger S. ^1^, Mänz J.S. ^1^ and Biselli S. ^2^

^1^ Eurofins WEJ Contaminants, Hamburg, Germany

^2^ Eurofins Rapidust Analysis, Hamburg, Germany

* Correspondence: mareikereichel@eurofins.de

**Abstract:** Occurrence of ochratoxin A (OTA) in grain is a result of *Aspergillus* or *Penicillium* infection on the field or later during storage. In Middle and Northern European cereals post-harvest infection due to humid storage prevails. As some *Penicillium* species already grow at water activities around 0.8, moderate variations in temperature can already cause ideal conditions for contamination in grain lots. In the past two years, increasing numbers of industrial recalls of rye products in Germany were reported. Rye is more susceptible to fungal growth than e.g., wheat as sorption isotherms are flat resulting in higher water activity at same relative humidity. However, increasing occurrence data may also be a result of increasing number of tests revealing a problem that was long times underestimated. A reason for this might be the difficulty to control grain lots on OTA and particularly to find the hot spots with high toxin loads.

As a result of cost-intensive recalls, insurance companies and industrial customers start to force mills to set up extensive OTA control. For the mills this challenge can only be solved with high effort and high costs. A control plan of an exemplary bread mill with storage capacity of 120.000 t and milling capacity of 280.000 t/a will be shown. The plan bases on extensive raw material control to find contaminations before grain processing. However, even dynamic sampling and ~2000 OTA tests per year resulted in insufficient detection of OTA hot spots. This is due to insufficient performance of on-site tests but also resulting from limitations in sampling. So, currently, the only possibility to ensure that OTA contaminations in flours are below the legal limit is to test additionally in the final product and block any batch in storage until the result of an external lab is available.

To overcome this expensive and inefficient monitoring a joint project was set-up to test whether dust sampling can be used to ensure proper raw material testing. Within the last years dust sampling was shown to be an effective way for fast and reliable control of grain lots for *Fusarium* toxins as deoxynivalenol. Also for aflatoxin in corn lots a significant reduction of the sampling induced variability from 67% to 15% at 20 μg/kg was shown. So, the potential to improve monitoring for low levels of *Aspergillus* toxins by dust sampling is given. However, a new method can never be better than a method you compare it to. Hence, the biggest challenge to develop a new strategy for OTA monitoring is to generate reliable reference data. First results and experiences to set up OTA data models for dust sampling of rye as well as study designs to prove reliability of dust compared to conventional sampling are discussed. Furthermore, the potential of dust analysis to improve on-site testing is shown. More sensitive detection further improves the potential to identify small OTA hot spots in grain lots. Based on both effects, enhanced reliability of sampling and improved rapid detection, a first evaluation of the potential of dust sampling for better and more cost-efficient monitoring plans for OTA in small grains is given.

### 7.17. EU Wheat Farmers’ Intentions to Improve Their Mycotoxin Management

Janssen E. ^1,^*, Mourits M. ^1^, van der Fels-Klerx I. ^1,2^ and Oude Lansink A. ^1^

^1^ Business Economics Group, Wageningen University and Research, Wageningen, The Netherlands

^2^ RIKILT, Wageningen University and Research, Wageningen, The Netherlands

* Correspondence: esmee.janssen@wur.nl

**Abstract:** Mycotoxin management is directed to increase the quality and safety of grains. Because mycotoxins are difficult or even impossible to remove further along the chain, mycotoxin management is mainly focussed on reducing initial fungal infection and production of mycotoxins in the field and during storage. Since farmers play a key role in this prevention and control of mycotoxin contamination, it is important to understand their behaviour regarding mycotoxin management. According to the Theory of Planned Behaviour, intentions can be used as a proximal measure of behaviour. Intentions are determined by three underlying behavioural constructs: attitudes, subjective norms and perceived behavioural control (Ajzen, 1991). Knowledge of these constructs regarding a certain behaviour will give a better insight in how to stimulate a change in behaviour in the future.

The aim of this study is to support the decision making process in mycotoxin management along the chain by assessing the intention of wheat farmers regarding the improvement of their mycotoxin management plan. In addition, the effect of relevant incentive mechanisms to stimulate this improvement will be assessed. For this purpose, a questionnaire based on the Theory of Planned Behaviour has been developed and will be distributed among different socio-demographic groups of EU wheat farmers. The results of the questionnaire will be analysed using multivariate regression analyses and will be ready for the conference to be presented and discussed.

## Reference

IcekAjzen. The theory of planned behavior. *Organ. Behav. Hum. Decis. Process.*
**1991**, *50*, 179–211.

### 7.18. Formulation and Delivery of the Biological Control Agents Clonostachys Rosea and Trichoderma Harzianum to Reduce Mycotoxins in Wheat

Gimeno A. ^1,^*, Bänziger I. ^1^, Kägi A. ^1^, Drakopoulos D. ^1^, Keller B. ^2^ and Vogelgsang S. ^1^

^1^ Research Group Ecology of Noxious and Beneficial Organisms, Research Division Plant Protection, Agroscope, Zurich, Switzerland

^2^ Molecular Plant Biology and Phytopathology, Department of Plant and Microbial Biology, University of Zurich, Zurich, Switzerland

* Correspondence: alejandro.gimeno@agroscope.admin.ch

**Abstract:** The orientation towards sustainable agricultural practices requires new and improved methods for the control of Fusarium Head Blight (FHB) in wheat to reduce the contamination of grain with hazardous mycotoxins. Biological control of FHB with microbial antagonists is raising a lot of interest to minimise the application of chemical fungicides. However, at this point, no microbial products against FHB are available to the farmer. In terms of efficacy and handling, the transition from laboratory to field remains a major obstacle to overcome. Within the scope of the Horizon 2020 project MycoKey, the goal of this PhD study is to investigate different strategies to control *Fusarium graminearum* (FG; teleomorph *Gibberella zeae*), the main species within the FHB disease complex and a potent producer of deoxynivalenol (DON) and zearalenone (ZEA). The fungal antagonist *Clonostachys rosea* (CR; teleomorph *Bionectria ochroleuca*) is the main biological control agent (BCA) for field, climate chamber and in vitro experiments. In cooperation with MycoKey partners, the near commercial BCA *Trichoderma harzianum* strain ITEM908 is included in all major experiments. The main objectives are: (1) Formulation of the antagonists for improved handling and efficacy and (2) Elucidating the effect of BCA applications onto FG infected maize residues or flowering wheat heads in a maize-wheat crop rotation.

Two field experiments have been initiated in autumn 2016 and a wettable powder (WP) formulation of CR was developed. Furthermore, the shelf life of the WP in cold storage and at room temperature is under investigation. The data from field and climate chamber trials will include quantification of the mycotoxins DON and ZEA, assessment of FG incidence with seed health tests as well as quantification of FG DNA with qPCR. Other experiments to examine the UV sensitivity of different CR isolates with and without the addition of UV protectants are ongoing. Preliminary results of the current study will be presented and discussed

### 7.19. Inhibition of DON Production by Fusarium Graminearum via Exposure to Trichoderma Harzianum

Tian Y., Liu N. and Wu A. *

SIBS-UGENT-SJTU Joint Laboratory of Mycotoxin Research, Key Laboratory of Food Safety Research, Institute for Nutritional Sciences, Shanghai Institutes for Biological Sciences, Chinese Academy of Sciences, University of Chinese Academy of Sciences, 294 Taiyuan Road, Shanghai 200031, China

* Correspondence: abwu@sibs.ac.cn

**Abstract:** Deoxynivalenol (DON) family mycotoxins (DONs) are generally including DON prototype, acetyl derivatives (3/15ADON) and modified forms (DON-3-*O*-glucoside, D3G), which are ubiquitously contaminated in wheat grains and thus aroused more and more attention on their synergistic side effects to human and animal health. Also, the control strategies of co-occurring DONs are increasingly in need related to food and feed safety. Very recently, we discovered effective inhibition of DON production by Fusarium graminearum via confrontation with Trichoderma harzianum, with co-occurrence of masked DON via glycosylation (D3G). Also, after RNAseq analysis and molecular characterization, we found some new candidate genes and proteins related to DON biosynthesis, as well as transferases for DON to D3G in wheat grains, which still need more subsequent confirmation on the functionalities responsible for DONs production. In total, we are looking for more scientific data as theoretical advances and more practical functional isolates with effective inhibition and degradation, which will definitely contribute to effective control of DONs, not merely DON itself, but derivatives and modified forms in foods.

### 7.20. First Year Experience with Large-Scale Application of an Aspergillus Flavus Biocontrol Agent for Aflatoxin Prevention in Italian Maize

Battilani P. ^1,^*, Mauro A. ^2^ and Cotty P.J. ^3^

^1^ Department of Sustainable Crop Production, Università Cattolica del Sacro Cuore, via Emilia Parmense 84, 29122 Piacenza, Italy

^2^ International Institute of Tropical Agriculture, P. BOX 34441 Dar es Salaam, Tanzania

^3^ U.S. Department of Agriculture-Agricultural Research Service, School of Plant Sciences, The University of Arizona Forbes Building, Room 303, 1140 East South Campus Drive, Tucson, AZ 85721, USA

* Correspondence: paola.battilani@unicatt.it

**Abstract:** Aflatoxins cause serious concern in many geographic areas and in diverse crops as a result of toxicity to humans and domestic animals. Carry-over of aflatoxins from animal feed to milk and milk products put further pressure on maize, milk, and cheese producers. Biocontrol, implemented through careful selection of non-aflatoxigenic genotypes of *Aspergillus flavus*, has been confirmed as the most effective tool for farmers to improve the safety of their production. Registered biopesticide products for aflatoxin prevention are commercially available in the U.S. and Africa and are in the process of becoming registered in Italy. The *A. flavus* genotype that is the active ingredient of the Italian biocontrol product was selected from *A. flavus* genotypes native to Italy that were collected starting in 2003. The active ingredient has natural genetic defects that eliminate abilities to produce both aflatoxins and cyclopiazonic acid. This genotype is also very competitive against aflatoxin-producing genotypes. In 2012, field trials intended to assess efficacy on maize during the natural contamination process, resulted in statistically significant reductions in aflatoxins of approximately 90%. This supported the intent to further develop this biopesticide. The selected *A. flavus* genotype was deposited in May 2013 at the MUCL fungal collection (MUCL54911) and a patent for the biopesticide was issued in September 2015, N.001417885. MUCL54911 became the active ingredient in the biopesticide AF-X1. In 2016, the biopesticide AFX1 was evaluated across approximately 15.000 ha in north Italy with a temporary authorization for use in commercial agriculture. Six maize collection centers participated in the monitoring of aflatoxin contamination levels in grain produced across the area. Data was collected from around 1500 farms and the maize was assessed for whether the aflatoxin content was below two thresholds, 3 and 20 μg/kg. Three μg/kg was required for maize intended as feed for dairy animals. Based on contamination data, only 1% of maize crops treated with AFX1 produced grain above 3 μg/kg. Whereas, grain from 49% of untreated crops had aflatoxin levels above 3 μg/kg and were not able to be sold to dairies. None of the maize treated with AFX1 exceeded 20 μg/kg. However, 26% of maize crops not treated with AFX1 produced grain that exceeded 20 μg/kg. In summary, AF-X1 was very effective at preventing unacceptable levels of aflatoxins in Italian maize. The milk, cheese, and farming industries hope these very positive results will facilitate the registration process and result in farmers rapidly obtaining unlimited access to this tool useful for improving food and feed safety in the maize value chain.

### 7.21. Biologically Based Methods for Reduction of the Fumonisin Mycotoxins in MAIZE

Alberts J.F. ^1,^*, Schatzmayr G. ^2^, Moll W-D. ^2^ and Gelderblom W.C.A. ^1^

^1^ Mycotoxicology and Chemoprevention Research Group/ Institute of Biomedical and Microbial Biotechnology, Cape Peninsula University of Technology/ Bellville, South Africa

^2^ BIOMIN Research Center/ BIOMIN, Tulln, Austria

* Correspondence: albertsh@cput.ac.za

**Abstract:** Home-grown maize is a major dietary staple in southern Africa and known to be frequently contaminated with unacceptable levels of the fumonisin mycotoxins. Rural subsistence farming communities in South Africa have some of the highest recorded levels of fumonisin (B_1_) in the world, with exposure to FB_1_ in adults more than four times above the provisional maximum tolerable daily intake (2 μg FB_1_/kg body weight/day) as set by the Joint Food and Agriculture Organization of the United Nations and the World Health Organization (FAO/WHO) Expert Committee on Food Additives. Although local commercial maize is contaminated with lower levels, daily exposure could still be a risk factor for disease development in impoverished communities.

The lack of effective and environmentally safe chemical control methods against fungal growth and mycotoxin production in food crops, has led to investigations into biologically safe alternatives to prevent these contaminants from entering the food chain. Biological pesticides and methods involving natural resources such as plants, microorganisms, genetic factors thereof and clay minerals are popular alternatives being evaluated.

*Fusarium* growth and fumonisin production are effectively reduced by several natural and biological methods, pre- and postharvest. Pre-harvest control methods include breeding for resistant maize cultivars; introduction of biocontrol microorganisms; application of phenolic plant extracts; and expression of antifungal proteins and fumonisin degrading enzymes in transgenic maize cultivars. Postharvest approaches include the removal of fumonisins from food by natural clay adsorbents and enzymatic degradation of fumonisins through decarboxylation and deamination by recombinant carboxylesterase and aminotransferase enzymes, respectively. However, these approaches are mainly aimed at commercialization and application in areas with established infrastructure, i.e., developed countries.

Other approaches such as simple, practical and affordable mycotoxin reduction techniques at household level, for subsistence maize farming communities in developing countries, are becoming increasingly important, i.e., hand-sorting, winnowing, washing, flotation and dehulling of maize. Integration of some of these community-based approaches with technological advances remains a challenge, but should be further developed and made accessible.

This paper will introduce a novel fumonisin esterase FumD maize kernel wash method for reducing fumonisin levels in rural home-grown maize. The enzymatic maize kernel wash method could find application specifically in the rural subsistent farmer context, and likely to impact positively on food safety and security, especially in rural populations reliant on maize as a dietary staple.

### 7.22. Efficacy of a Fungal and Bacterial Antagonist for Controlling Growth, FUM1 Gene Expression and Fumonisin B_1_ by Fusarium Verticillioides, on Maize cobs of Different Ripening Stages

Samsudin N.I.P. ^1,2^, Rodriguez A. ^1,3^, Medina A. ^1^ and Magan N. ^1,^*

^1^ Applied Mycology Group, Environment and AgriFood Theme, Cranfield University, Cranfield, Beds. MK43 0AL, UK

^2^ Department of Food Science & Food Safety Centre, Faculty of Food Science and Technology, Universiti Putra Malaysia, 43400 UPM Serdang, Selangor, Malaysia

^3^ Food Hygiene and Safety, Meat and Meat Products Research Institute, Faculty of Veterinary Science, University of Extremadura, Avda. de la Universidad, s/n, 10003 Caceres, Spain

* Correspondence: n.magan@cranfield.ac.uk

**Abstract:** Previous studies identified two biocontrol agents (*Clonostachys rosea* 016; gram negative bacterium) with potential for control of fumonisin B_1_ production in vitro and in stored maize under different water availabilities (Samsudin & Magan, 2016). These have now been complemented by studies of the efficacy of these two antagonists on maize cobs of different ripening stages: R_3_, Milk (0.985 a_w_); R_4_, Dough (0.976 a_w_); R_5_, Dent (0.958 a_w_). The cobs were inoculated with 50:50 mixtures of the pathogen:antagonist inoculum ratio and stored in environmental chambers to maintain these conditions for 10 days at 25 and 30 °C. The growth rate of *F.verticillioides*, the relative expression of the *FUM*1 gene and fumonisin B_1_ (FB_1_) production were quantified (Samsudin et al., 2017). Water activity (a_w_) × temperature had significant impacts on growth, *FUM*1 gene expression and FB_1_ production by the strain of *F.verticillioides* on maize cobs of different maturities. The *C. rosea* 016 antagonist significantly reduced FB_1_ contamination on maize cobs by >70% at 25 °C, and almost 60% at 30 °C regardless of maize ripening stage. For the bacterial antagonist FB_1_ levels on maize cobs were significantly decreased in some treatments only. These results suggest that efficacy of antagonists to control mycotoxin production in ripening maize cobs needs to take account of the ecophysiology of the pathogen and the antagonist to ensure that effective control can be achieved.

## References

Samsudin, N.I.P.; Magan, N. Efficacy of potential biocontrol agent thresholds for control of Fusarium verticillioides and fumonisin B1 production under different environmental conditions on maize-based medium. *World Mycotoxin J.*
**2016**, *9*, 205–213.Samsudin, N.I.P.; Rodriguez, A.; Medina, A.; Magan, N. Efficacy of fungal and bacterial antagonists for controlling growth, FUM1 gene expression and fumonisin B1 production by Fusarium verticillioides on maize cobs of different ripening stages. *Int. J. Food Microbiol.*
**2017**, *246*, 72–77.

### 7.23. Antifungal Activity of the Allyl Isothiocyanate Against Aspergillus Flavus and Penicillium Nordicum in Corn, Wheat and Barley

Quiles J.M. ^1^, Torrijos R. ^1^, Saladino F. ^1^, Luciano F.B. ^2^, Mañes J. ^1^ and Meca G. ^1,^*

^1^ Laboratorio de Química de los Alimentos y Toxicología de la Facultat de Farmàcia, Universitat de València. Av. Vicent Andrés Estellés s/n, 46100 Burjassot, Spain

^2^ Departamento de ciência animal, Escola de Ciências da Vida, Pontificia Universidade Católica do Paraná. Rua Imaculada Conceição 1155, 80901-215 Curitiba, Paraná, Brazil

* Correspondence: giuseppe.meca@uv.es

**Abstract:** Glucosinolates are metabolites found in plants belonging to the family Brassicaceae, once in the cytoplasm, they are hydrolyzed by the enzyme myrosinase, resulting in the formation of three main groups of substances: nitriles, thiocyanates and isothiocyanates (ITCs). The antifungal activity of the ITCs is due to the strong electrophilic properties of these compounds and also they can react easily with nucleophiles such as amines, amino acids, alcohols, water, and sulfites during food treatment and under physiological conditions and also with several functional groups of many mycotoxins. The aims of this study were to evaluate the antifungal properties of the bioactive compound allyl isothiocyanate (AITC) against *Aspergillus flavus* (AFs producer) and *Penicillium nordicum* (OTA producer) on wheat corn and barley. The experiments were carried out in a lab scale silo system that was composed by a glass jars containing 500 g of cereals wheat and barley (contaminated with 1 × 10^4^ spores/g of *P. nordicum*) and corn (contaminated with with 1 × 10^4^ spores/g *A. flavus*). The cereals were treated with 5, 25 and 50 ppm of gaseous AITC, hermetically closed and incubated during 10, 20, 30, 60 and 90 days at 21 °C. The cereals control group did not receive any treatment. After each incubation time, fungal population and mycotoxin content were determined respectively. Results obtained on *A. flavus* evidenced that using 5 ppm of AITC the reduction of the fungal growth in comparison with the control experiment was of 0.2 Log, whereas using 25 and 50 ppm the *A. flavus* reduction evidenced were of 0.2 and 0.5 log respectively. Considering the AFs production, in the control experiments at 90 days incubation were detected 0.54 and 0.36 ppm of the AFB_1_ and AFB_2_ respectively, whereas in all the trials carried out using the AITC the bioactive compounds AFB_1_ and AFB_2_ were no detected in any of the incubation time points analyzed, evidencing a good capacity of the AITC to reduce from one hand the *A. flavus* growth and on the other hand to inhibit the production of the AFs. The results obtained on *P. nordicum* inhibition growth and also on the OTA reduction in wheat and barley were comparable with those obtained on corn. In this study we demonstrated also the capacity of the AITC to react with the FBs presents naturally in corn reducing the concentration of these toxic compounds at 90 day from 50 to 58% respectively.

### 7.24. Effectiveness of Agrochemicals in Reducing Fusarium Vertillioides (sacc.) Nirenberg Infection and Contamination of Fumonisins in Maize of Low Land Agro-Ecosystems in Tanzania

Madege R. ^1^, Audenaert K. ^2^, Kimanya M. ^3^, Tiisekwa B. ^1^, de Meulenaer B. ^4^, Bekaert B. ^2^ and Haesaert G. ^2^^,^*

^1^ College of Agriculture, Sokoine University of Agriculture, Tanzania

^2^ Department of Applied Biosciences, Faculty of Bioscience Engineering, Ghent University, Belgium

^3^ School of life Sciences and Bio Engineering, The Nelson Mandela African Institution of Science and Technologies, Tanzania

^4^ Deparment of Food Safety and Food Quality, Faculty of Bioscience Engineering, Ghent University, Belgium

* Correspondence: geert.haesaert@ugent.be

**Abstract:**
*Fusarium verticillioides* is the most common *Fusarium* species associated with maize ear rot in Tanzania. In a two-year trial, we investigated the effectiveness of pesticides and fertilizer in reducing occurrence of *Fusarium verticillioides* and its mycotoxins in maize of lowland agro ecosystem of Tanzania. Cob and kernel injury in maize treated with insecticides in combination with fungicides was three times lower than maize treated with fungicide alone. The additive effects of organochloride insecticides (Thionex 35EC (350 g/L endosulphan) and fungicides Rustal 375 EC (125 g/L triadimenol, 250 g/L tebucunazole) comprised a comprehended reduction in ear and kernel rot, reduction in fungal biomass and reduction of fumonisins. With regard to fertilizer, the effects of amendment of 40 kgN/ha of Urea (46%) and 30 kgP/ha of Triple super phosphate (52% P_2_O_5_) on FB1 and FB2 were not different from untreated control except when these fertilizer amendments were combined with insecticide and fungicides. Significant correlations between climatic variables and contamination of fumonisins were established. This study clearly demonstrated that application of insecticide amended with fungicides at anthesis can potentially reduce infection of *F. verticillioides* and contamination of FB1, FB2 and consequently the total fumonisins which is an important finding for the local Tanzanian farmers.

### 7.25. Bacillus Subtilis as a Biocontrol Organism Towards Penicillium Roqueforti s.l. in Silage: In Vitro and In Vivo Evaluation

Wambacq E. ^1,^*, Audenaert K. ^2^, Höfte M. ^3^, De Saeger S. ^4^ and Haesaert G. ^1^

^1^ Division of Plant Production, Department of Applied Biosciences, Faculty of Bioscience Engineering, Ghent University, Ghent, Belgium

^2^ Laboratory for Applied Mycology and Phenomics, Department of Applied Biosciences, Faculty of Bioscience Engineering, Ghent University, Ghent, Belgium

^3^ Laboratory for Phytopathology, Department of Crop Protection, Faculty of Bioscience Engineering, Ghent University, Ghent, Belgium

^4^ Laboratory of Food Analysis, Department of Bioanalysis, Faculty of Pharmaceutical Sciences, Ghent University, Ghent, Belgium

* Correspondence: eva.wambacq@ugent.be

**Abstract:** In Belgium, silages are often infected by the toxigenic fungal species *Penicillium roqueforti s.s.* and *P. paneum*. These two fungal species, referred to as *P. roqueforti s.l.*, are closely related and are very well adapted to silage conditions, making it difficult to prevent their growth and possible mycotoxin production in silages. Chitarra et al. (2013) demonstrated that an antifungal compound in the culture supernatant of *Bacillus subtilis* strain NRRL B-23189 inhibits the germination of *P. roqueforti s.s.* conidiospores. Therefore, the antagonistic effect of this specific *B. subtilis* strain has been assessed against a *P. roqueforti s.s.* and a *P. paneum* isolate.

In an in vitro assay, maize silage infusion was combined with different amounts of *B. subtilis* test solution: *B. subtilis* culture supernatant or cell suspension, both obtained after 48 h of incubation in Brain-Heart Infusion broth. The different culture media were infected with 1.10^4^
*P. roqueforti s.l.* conidiospores per mL and incubated aerobically at 20 °C during five days. Fungal growth was monitored by spectrophotometric determination of the optical density (OD) at 620 nm (N = 4). Spore germination was evaluated microscopically after 24 h (N = 3). Spore survival was monitored after 1, 2, 4, 8 and 24 h of incubation (N = 3). A quantitative screening of roquefortine C production during the five-days incubation period was performed by LC-MS/MS (N = 1). Interestingly, a severe reduction of *P. roqueforti s.l.* spore survival was observed after 24 h of incubation by both *B. subtilis* supernatant or cell suspension addition to maize silage infusion and moreover, roquefortine C production was not increased. These experimental results render *B. subtilis* a promising candidate silage inoculant to counteract the growth of and possible mycotoxin production by *P. roqueforti s.l.* in silages.

To check if *B. subtilis* NRRL B-23189 as a silage inoculant is able to reduce *P. roqueforti s.l.* numbers in vivo in artificially infected grass silage, a microsilo experiment was performed. Grass was mown, prewilted, chopped and treated with 10 mL per kilogram fresh matter of test solution: sterile physiological water (negative control) or *B. subtilis* cell suspension (5 × 10^5^ cfu g^−1^ fresh matter). For both objects, four 2.75 liter microsilos were filled. All microsilos were desiled after 56 days and samples were taken for enumeration of *P. roqueforti s.l.* Inoculation with *B. subtilis* did not result in significantly lower *P. roqueforti s.l.* numbers compared to the negative control. Therefore, this microsilo experiment with grass could not attribute biocontrol activity towards *P. roqueforti s.l.* to *B. subtilis* as a silage inoculant in vivo.

### 7.26. Succinate Dehydrogenase Inhibitor Fungicides Effectiveness Against Aspergillus Flavus and *Fusarium* Species

Masiello M., Somma S., Ghionna V., Logrieco A.F. and Moretti A. *

Institute of Sciences of Food Production, National Research Council (ISPA-CNR), Bari, Italy

* Correspondence: antonio.moretti@ispa.cnr.it

**Abstract:** Many toxigenic fungi mainly belonging to *Aspergillus* and *Fusarium* genera can colonize maize plants. In particular, the contamination of the maize kernels by *A. flavus* is worrisome since this fungus can produce Aflatoxin B1, a mycotoxin causing hepatocellular carcinoma. Moreover, also the so called “Fusarium maize ear rot” disease is caused by several species of the *Fusarium* genus that produce harmful mycotoxins. In particular, *F. proliferatum* and *F. verticillioides* both produce the carcinogenic mycotoxins fumonisins, while *F. graminearum sensu stricto* produces the strong inhibitor of proteic synthesis deoxynivalenol. According with different geographical areas, all the above mentioned species are reasons of concern for the consequences of their impact on human and animal health. Nowadays, according to Integrated Pest Management guidelines, agronomic, genetic and chemical strategies are suggested to reduce fungal and related mycotoxin contaminations. However, there is poor knowledge on the fungicides effectiveness to control “maize ear rot” disease. Moreover, only few molecules are registered for maize seed coating and no chemical compounds are registered for spray treatment. In the last years, Succinate Dehydrogenase Inhibitor (SDHI) fungicides showed excellent performances against several fungal genera on different crops. SDHIs inhibit fungal respiration by blocking the ubiquinone-binding site of the enzyme succinate-ubiquinone oxido-reductase (SDH, so-called complex II) in the mitochondrial electron transport chain.

The sensitivity of *Fusarium* species and *Aspergillus flavus* to two SDHIs, Boscalid and Isopyrazam, was evaluated by colony growth and conidial germination assays.

Boscalid was unable to inhibit *Fusarium* mycelial growth after three days (average of inhibition at highest concentration ranged between 0 and 30%), while after 10 days of incubation, the mycelial growth was totally not affected. Isopyrazam showed a higher effectiveness than Boscalid against *Fusarium* up to 10 days of incubation (average of inhibition at highest fungicide quite 100%). Same response was observed in conidial germination test: Boscalid did not inhibit conidial germination also at the highest concentration tested (500 mg/kg), while Isopyrazam inhibited the germ tube elongation also at the lowest dose (2 mg/kg), with an average of inhibition around 97%.

*Aspergillus flavus* showed a higher sensitivity than *Fusarium* species to both SDHIs up to 10 days of incubation (average of inhibition at lower dose, 100% for Boscalid and 80% for Isopyrazam).

Since in many phytopathogenic fungi mutations in genes encoding succinate dehydrogenase sub-units are responsible for different level of sensitivity to SDHIs, studies are in progress in order to explain molecular mechanisms associated to different sensitivity among fungal genera and the different response of two SDHI fungicides belonging to same chemical group.

This research was supported by Horizon 2020 project Mycokey (GA N. 678781).

### 7.27. Control of Pre-Harvest Aflatoxin Contamination in Corn Using Rna Interference-Mediated Approaches

Cary J. *, Majumdar R., Gilbert M., Sickler C., Wei Q. and Rajasekaran K

USDA, ARS, Southern Regional Research Center, New Orleans, LA 70124, USA

* Correspondence: jeff.cary@ars.usda.gov

**Abstract:** Among the aspergilli, *Aspergillus flavus* frequently contaminates agricultural commodities such as corn, peanut, tree nuts and cottonseed with aflatoxins. Ingestion of contaminated foods, especially corn, has been implicated in acute toxicoses while chronic, low-level exposure can lead to immune suppression and liver cancer in humans. In addition to the health risks associated with aflatoxins there are significant adverse economic impacts to producers due to market rejections of contaminated crops, livestock losses and costs associated with monitoring for aflatoxin contamination. Pre-harvest aflatoxin contamination of corn is a very complex problem affected by a multitude of genetic, environmental and nutritional factors. To incorporate resistance into crop plants, we are employing RNAi interference (RNAi)-based silencing, a form of host-induced gene silencing (HIGS) that can target genes with high specificity before they are translated into proteins. Using this approach, our objectives are (1) determine the contribution of corn genes predicted to be involved in resistance to *A. flavus* infection and aflatoxin contamination; and (2) facilitate host plant-directed down-regulation of genes critical to *A. flavus* virulence and toxin production. The corn pathogenesis-related gene *PRms* has been implicated as playing a role in host resistance to *A. flavus*. Expression of *PRms* has been shown to be induced in the kernel upon infection by *A. flavus*. We have generated transgenic corn lines expressing hairpin RNAs under the control of the maize endosperm-specific zein gene promoter (27*Zn*) for silencing of the *PRms* gene. Screening of transgenic *PRms*-RNAi kernels infected with a GFP-expressing *A. flavus* strain using a kernel screening assay (KSA) demonstrated a significant increase in both fungal growth and aflatoxin production in transgenic RNAi lines compared to controls. These results indicate that *PRms* contributes significantly to resistance to aflatoxin contamination in corn. Previous studies conducted by our group and our collaborators have identified a number of key genes in *A. flavus* that are critical for growth and aflatoxin production [e.g., *amyA* (alpha-amylase); aflatoxin regulatory and biosynthetic genes such as *aflR*, *ver-1*, *pksA*; and global regulators of fungal growth and secondary metabolism such as *veA*, *nsdC*, and *hbx1*] that can serve as targets for HIGS. We are currently evaluating corn kernels collected from transgenic events transformed with an *A. flavus* alpha-amylase (*amy*) gene-RNAi vector designed to silence the fungal amylase gene. Seed collected from unique *amy*-RNAi transgenic events demonstrated increased resistance to fungal growth and aflatoxin production following infection of seed. Further progress to date on control of pre-harvest aflatoxin contamination in corn using an RNA interference-mediated approach to silence genes critical for *A. flavus* growth and/or toxin production will be provided.

### 7.28. Cropping Systems to Decrease the Risk of Mycotoxins in Wheat

Drakopoulos D. ^1,2,^*, Kägi A. ^1^, Forrer H. ^1^, Jenny E. ^1^, Gimeno A. ^1^, Bänziger I. ^1^, Six J. ^2^ and Vogelgsang S. ^1^

^1^ Research Group Ecology of Noxious and Beneficial Organisms, Research Division Plant Protection, Agroscope, Zurich, Switzerland

^2^ Group of Sustainable Agroecosystems, Institute of Agricultural Sciences, ETH Zurich, Switzerland

* Correspondence: dimitrios.drakupoulos@agroscope.admin.ch

**Abstract:** Fusarium Head Blight (FHB) is one of the most important cereal diseases worldwide causing not only significant reductions in grain yield but also severe contaminations of the harvested products with mycotoxins jeopardising food and feed safety. The predominant species of FHB disease complex is *Fusarium graminearum* (FG; teleomorph *Gibberella zeae*). In maize-wheat rotations with reduced- or no-till systems, the remaining maize crop residues on the soil surface serve as overwintering substrate and thus represent an important inoculum source for infection of the subsequent cereal crop. Hence, agronomic practices such as suitable crop rotations and management of crop residues with ploughing are commonly used to control FHB in wheat cropping systems. Nevertheless, continuous ploughing has also several drawbacks, such as increased soil erosion risks and decreased soil fertility in the upper soil layers.

Within the framework of the Horizon 2020 project MycoKey, the main objective of this PhD study is to develop strategies to suppress FHB and thus to prevent subsequent mycotoxin accumulation in wheat through intercropping, cover cropping or biofumigant crops in a reduced/no-till maize-wheat rotation. For this purpose, several field experiments were initiated: (a) Maize-intercropping systems and management of crop residues; (b) Use of cover crops as an interval within a maize-wheat rotation; (c) Application of mustard-based botanicals and freshly mulched material from *Brassica* fields onto FG infected maize residues. Collected data will include deposition of discharged *G. zeae* spores (spore traps), disease rating in the field, crop yield, *Fusarium* species incidence and severity (seed health tests and qPCR, respectively) and mycotoxin analysis (LC-MS/MS). In vitro studies showed that certain mustard-based botanicals suppress mycelium growth and conidia germination of three FG strains and therefore could be promising treatments to manage FHB infection and mycotoxin contamination in cereals. Preliminary results of this study will be presented and discussed.

### 7.29. Agronomic Practices and Mycotoxin Contamination in Maize: A Case Study of Subsistence Farming Households in Manicaland and Mashonaland West Provinces of Zimbabwe

Ndemera M. ^1,2,^*, Landschoot S. ^3^, De Boevre M. ^1^, Nyanga L.K. ^2^ and De Saeger S. ^1^

^1^ Laboratory of Food Analysis, Ghent University, Ottergemsesteenweg 460, 9000 Ghent, Belgium

^2^ Institute of Food Nutrition and Family Sciences, University of Zimbabwe, P.O. Box MP167, Mount Pleasant, Harare, Zimbabwe

^3^ Department of Applied Bioscience Engineering, Ghent University, Valentin Vaerwyckweg 1, 9000 Ghent, Belgium

* Correspondence: melody.hove@ugent.be

**Abstract:** To fully elucidate and understand maize agronomic practices by Zimbabwean subsistence farming populations an investigative field survey was conducted in the selected provinces of Mashonaland West and Manicaland. Agronomic data and associated climatic data was collected during the 2014/2015 agricultural season. A total of 158 maize samples were also collected from the household’s harvest at harvest, three months post-harvest and six months post-harvest. Analysis and quantification of mycotoxin contamination in the maize samples was performed using a validated multi-mycotoxin LC-MS/MS analysis method for the detection and quantification of 23 mycotoxins. The sampled maize was mainly contaminated by *Fusarium* spp. mycotoxins. Fumonisin B1 (FB1) was the most prevalent, occurring in 23, 47 and 47% of samples at harvest, three moths post-harvest and six-months post-harvest respectively. The corresponding means of positive samples were 609, 597 and 289 μg/kg respectively. The choice of seed and fertilizer application were significant in modulating mycotoxin contamination in maize pre-harvest. There was no significant change in mycotoxin contamination post-harvest. High temperatures were also associated with high levels of FB1 particularly at the flowering stage of the maize. Rainfall was positively correlated with FB1 contamination. Good agricultural practices were attributed to influencing mycotoxin contamination insubsistence farmed maize from Zimbabwe.

### 7.30. Assessment and Mitigation of Aflatoxin and Fumonisin Contamination in Animal Feeds in Rwanda

Nishimwe K. ^1,2,^*, Bowers E. ^1^, Ayabagabo JdD. ^2^, Habimana R. ^2^, Mutiga S. ^3^ and Maier D. ^1^

^1^ Iowa State University, Iowa, USA

^2^ University of Rwanda, Kigali, Rwanda

^3^ Bioscience Eastern and Central Africa–International Livestock Research Institute (BecA-ILRI), Nairobi, Kenya

* Correspondence: nishimwe@iastate.edu

**Abstract:** Aflatoxins and fumonisins are fungal metabolites that contaminate crops and animal feeds under favorable growth conditions. They are of importance to public and animal health as they are associated with or are causative agents of certain cancers in humans. The Feed the Future Innovation Lab for Livestock Systems (LSIL) based at University of Florida launched several competitively-funded multi-disciplinary, integrated and applied research and capacity-building projects. Iowa State University secured a 1-year LSIL-funded research project with the aim of assessing and mitigating aflatoxin and fumosin contamination in animal feeds in Rwanda.

Specifically, this project focuses on:
Quantify aflatoxin and fumonisin levels in animal feeds at different points in the animal feeds value chain.Establish a surveillance and early detection system for aflatoxin and fumonisin presence and mitigation in animal feeds.Raise awareness of aflatoxin and fumonisin contamination and prevention among stakeholders involved in the animal feeds value chain.Provide input to the regulatory framework in regards to policies for the mitigation of aflatoxin and fumonisin contamination in animal feeds.

Preliminary results to improve food and feed safety for a better livelihood in Rwanda will be shared in this presentation.

### 7.31. Validated Lc-Ms/Ms Multi-Methods to Detect 25 Mycotoxins and In Vivo Phase I Metabolites in Biological Fluids of Pigs and Broiler Chickens and Application to Screening Studies for Mycotoxin Exposure

Lauwers M. ^1,2,^*, De Baere S. ^1^, Antonissen G. ^1^, Letor B. ^2^, Van Limbergen T. ^3^, Maes D. ^3^, Devreese M. ^1^ and Croubels S. ^1,^*

^1^ Department of Pharmacology, Toxicology and Biochemistry, Faculty of Veterinary Medicine, Ghent University, Salisburylaan 133, 9820 Merelbeke, Belgium

^2^ Innovad, Antwerpen-Berchem, Belgium

^3^ Department of Obstetrics, Reproduction and Herd Health, Faculty of Veterinary Medicine, Ghent University, Salisburylaan 133, 9820 Merelbeke, Belgium

* Corresponding authors: marianne.lauwers@ugent.be; siska.croubels@ugent.be

**Abstract:** Worldwide surveys show that mycotoxins occur in more than 70% of the tested feed samples. In 38% of these samples multiple mycotoxins were found [1]. Therefore, methods that can simultaneously detect multiple mycotoxins are preferred. Until now, mycotoxin exposure in animals is mainly investigated by feed analysis. However, it is well known that so called ‘hot spots’ are responsible for an uneven distribution and non-proportional spread of mycotoxins in feed, hampering representative sample collection and evaluation of mycotoxin exposure in animals. This can be overcome by the analysis of biological matrices (e.g., plasma, urine, faeces), since the mycotoxin concentration may be more constant and information about the exposure of the animal on an individual level is possible.

The presented methods aim to detect mycotoxins and their in vivo phase I metabolites in plasma and excreta of broiler chickens and in plasma, urine and faeces of pigs. The targeted mycotoxins belong to the regulated groups, i.e., aflatoxins, ochratoxin A, *Fusarium* mycotoxins (T2-toxin, zearalenone, deoxynivalenol, fumonisins) and to two groups of emerging mycotoxins, i.e., *Alternaria* mycotoxins and enniatins.

Sample preparation of pig plasma was accomplished by deproteinization with acetonitrile. An additional clean-up step using an Ostro^®^-plate was required for chicken plasma to remove lipophilic substances. The sample preparation for all other matrices was achieved with a pH-dependent liquid-liquid extraction using different extraction solvents, depending on the matrix. The liquid chromatography-tandem mass spectrometry (LC-MS/MS) analysis was done using a HSS-T3 UPLC column with appropriate pre-column on an Acquity UPLC system coupled to a Xevo TQ-S mass spectrometer operating in positive and negative electrospray ionisation mode. All methods were in-house validated according to European and international guidelines [2,3].

Subsequently, these methods were applied to screen the occurrence of mycotoxins and in vivo phase I metabolites in samples collected from different broiler chicken and swine farms in Europe, where problems with mycotoxins were suspected. Besides, their phase II in vivo metabolites were determined using HR-MS.
Kovalsky, P.; Kos, G.; Nährer, K.; Schwab, C.; Jenkins, T.; Schatzmayr, G.; Sulyok, M.; Krska, R. Toxins. 2016, 8(12).VICH GL49, FDA, 2015.Volume 8, EC, 2005.

### 7.32. In Vivo Contribution of α-Zearalenol, β-Zearalenol, Zearalenone-14-Glucoside and Zearalenone-14-Sulphate to Zearalenone Exposure in Pigs: Oral Bioavailibility, Hydrolysis and Toxicokinetics

Catteuw A. ^1,^*, Broekaert N. ^1^, De Saeger S. ^2^, De Boevre M. ^2^, De Baere S. ^1^, Lauwers M. ^1^, Gasthuys E. ^1^, Gehring R. ^3^, Devreese M. ^1^ and Croubels S. ^1^

^1^ Department of Pharmacology, Toxicology and Biochemistry, Faculty of Veterinary Medicine, Ghent University, Salisburylaan 133, 9820 Merelbeke, Belgium

^2^ Department of Bioanalysis, Faculty of Pharmaceutical Sciences, Ghent University, Ottergemsesteenweg 460, 9000 Ghent, Belgium

^3^ Institute of Computational Comparative Medicine, College of Veterinary Medicine, Kansas State University, Manhattan, KS, USA

* Correspondence: Amelie.Catteuw@UGent.be

**Abstract:** With an incidence of 80% in unprocessed cereals, zearalenone (ZEN) is one of the most important *Fusarium* mycotoxins, due to its widespread occurrence and toxic properties. Besides ZEN, so called modified forms are frequently detected in food and feed as well. They are produced by plants and rival fungi as part of a natural defence strategy against xenobiotic compounds like mycotoxins. The goal of this study was to determine the absolute oral bioavailability, (presystemic) hydrolysis/biotransformation and toxicokinetic characteristics of ZEN, α-zearalenol (α-ZEL), β-zearalenol (β-ZEL), zearalenone-14-glucoside (ZEN14G) and zearalenone-14-sulphate (ZEN14S) in pigs. Crossover animal trials were performed by single intravenous (IV) and oral (PO) administration of 331 μg ZEN/kg bodyweight and equimolar doses of α-ZEL, β-ZEL, ZEN14G and ZEN14S. Systemic plasma concentrations of ZEN, the modified forms of ZEN mentioned above as well as the major phase I metabolites α- and β-ZEL, were quantified using liquid chromatography-tandem mass spectrometry (LC-MS/MS). Liquid chromatography coupled to high-resolution mass spectrometry (LC-HR-MS) was used to unravel phase II metabolism of ZEN and the modified forms. Additionally, portal plasma was analysed to study presystemic hydrolysis and biotransformation of the administered mycotoxins. Data were processed *via* tailor-made compartmental toxicokinetic models. After IV administration of ZEN, a partial phase I biotransformation of ZEN to α-ZEL, and not to β-ZEL, occured. A partial conversion to ZEN was demonstrated after both α-ZEL and β-ZEL administration. No conversion between α-ZEL and β-ZEL was observed. Elimination half-life of all modified forms was found to be shorter than the elimination half-life of ZEN itself, which can be explained by the higher polarity compared to ZEN. After oral administration, plasma concentrations of ZEN, ZEN14G, ZEN14S as well as α-ZEL and β-ZEL, were below the limit of quantification. This can be attributed to limited oral absorption and/or extensive first pass metabolism of ZEN and its modified forms. Indeed, glucuronide metabolites of ZEN, α-ZEL and β-ZEL could be detected after PO administration, demonstrating these phase II metabolites are appropriate biomarkers for ZEN exposure in pigs. ZEN14G undergoes partial hydrolysis after IV (20.0 ± 1.59%) and PO administration. In remarkable contrast to ZEN14G, no ZEN was detected after both IV and PO administration of ZEN14S. This indicates that no presystemic nor systemic hydrolysis to ZEN takes place. In conclusion, it can be stated that the fate of ZEN and its modified forms is highly compound specific in the pig and results highlight the importance of revealing appropriate biomarkers (ZEN- and ZEL-glucuronide) for ZEN exposure in pigs.

### 7.33. Effects of Fumonisins and Deoxynivalenol on Turkeys and the Efficacy of a Mycotoxin Deactivating Enzyme and a Bacterial Strain to Counteract

Schaumberger S. ^1,^*, Masching S. ^1^, Doupovec B. ^2^ and Hofstetter U. ^1^

^1^ BIOMIN Holding GmbH, Getzersdorf, Austria

^2^ BIOMIN Research Center, Tulln, Austria

* Correspondence: simone.schaumberger@biomin.net

**Abstract:** In 2016 more than 16,500 samples of different feed and raw materials were analyzed as part of the BIOMIN Mycotoxin Survey Program. On a global basis, about 34% of all investigated samples were contaminated with fumonisins and 53% of all samples were contaminated with deoxynivalenol, being the most prevalent ones. The high prevalence of fumonisins and deoxynivalenol as well as the negative impact on poultry’s health and performance demands the necessity of successful counteraction of these toxins.

For a successful registration of products and/or components to counteract mycotoxins in animals in the European Union, studies with respective biomarkers are requested. EFSA filed a positive opinion on the use of a fumonisin esterase (FUMzyme^®^) and a life bacterial strain (Biomin^®^ BBSH 797) in diets for all avian species in autumn 2016. FUMzyme^®^ biotransforms the toxic fumonisin B1 (FB1), cleaving off two tricarballylic acid side chains resulting in the much less toxic hydrolyzed fumonisin B1 (HFB1). The level of FB1 and HFB1 in feces are recognized biomarkers. Turkeys were fed 15 ppm of FB1 with and without the fumonisin degrading enzyme. A decreased level of FB1 and an increased level of HFB1 in feces proved the capacity of the fumonisin esterase to degrade FB1 to HFB1. The sphinganine to sphingosine ratio (Sa/So) in serum was analyzed as and additional marker. Sa/So was decreased in the group receiving the enzyme confirming the efficacy of the additive.

Biomin^®^ BBSH 797 is the name for a feed additive containing viable cells of a Genus nov. (formerly *Eubacterium*) sp. nov. bacterium (DSM 11798) which degrades deoxynivalenol (DON) by deepoxydation. As reported in literature (Wan, D. et al., 2014) DON-3-sulfate is the major metabolite of DON in poultry. The resulting de-epoxy metabolite of Biomin^®^ BBSH 797 activity is DOM-3-sulfate. DON, DOM-1, DON-3-sulfate and DOM-3-sulfate were used as biomarkers in feces. and to investigate the efficacy of the product. Turkeys were fed DON contaminated feed with and without Biomin^®^ BBSH 797. In the group that received both, DON contaminated feed and Biomin^®^ BBSH 797, the level of DON-3-sulfate was significantly reduced by transforming into DOM-3-sulfate, showing the successful biotransformation capacity by the bacterial strain.

The above mentioned trials show the effects of DON and FUM on biomarkers. An additive based on a specific enzyme to detoxify fumonisins and a bacterial strain to biotransform trichothecenes, reduces the effects of frequently occurring mycotoxins on turkeys.

### 7.34. Effect of Feed-Borne *Fusarium* Mycotoxins on the Gut Microbiome Composition in Broiler Chickens and in Pigs

Antonissen G. ^1,2,^*, Van Immerseel F. ^2^, Michiels A. ^3^, Eeckhaut V. ^2^, Vermeulen K. ^2^, Reisinger N. ^4^, Baeyen S. ^5^, Haegeman A. ^5^, Audenaert K. ^6^, De Saeger S. ^7^, Maes D. ^3^, Haesebrouck F. ^2^, Ducatelle R. ^2^ and Croubels S. ^1^

^1^ Department of Pharmacology, Toxicology and Biochemistry

^2^ Department of Pathology, Bacteriology and Avian Diseases

^3^ Department of Obstetrics, Reproduction and Herd Health, Faculty of Veterinary Medicine, Ghent University, Salisburylaan 133, 9820 Merelbeke, Belgium

^4^ Biomin Research Center, Tulln, Austria

^5^ Plant Sciences Unit, Flanders Research Institute for Agriculture, Fisheries and Food, Melle, Belgium

^6^ Department of Applied Biosciences, Faculty of Bioscience Engineering, Ghent University, Ghent, Belgium

^7^ Department of Bioanalysis, Faculty of Pharmaceutical Sciences, Ghent University, Ghent, Belgium

* Correspondence: Gunther.Antonissen@UGent.be

**Abstract:** The mycotoxins deoxynivalenol (DON) and fumonisins (FBs) exert toxicity toward the intestinal epithelial barrier and immune system. Besides, following ingestion of contaminated feed, the intestinal microbiota will be exposed to these toxins. The gut harbours a complex community of over trillions microbial cells which influence animal physiology, metabolism, nutrition and immune function, while disruption of the gut microbiota has been linked with poor intestinal health. The aim of this presentation is to discuss comparatively the impact of mycotoxins on intestinal microbiota composition in broiler chickens and pigs. In a first study, the impact of DON and FBs on the composition of the intestinal microbiota in broiler chickens of different ages was assessed. Chickens of 1 day old were fed either a control diet, a DON contaminated diet (3.3–3.7 mg DON/kg feed) or a FBs contaminated diet (15.5–18.3 mg FB_1_ + FB_2_/kg feed). Subsequently, after a feeding period of one, three and five weeks chickens were euthanized and ileal and caecal content samples were collected. In a second study, the impact of DON and its acetylated forms on the gut microbiome in piglets of 3 weeks old was investigated. Piglets were fed either a control diet or a DON contaminated diet (0.4 mg DON, 0.3 mg 3-acetylDON, and 0.8 mg 15-acetylDON/kg feed) for 5 weeks. Following euthanasia, colon content samples were collected. Intestinal microbiome profiling was performed by next-generation sequencing of 16S ribosomal DNA. The hyper-variable V4 region was amplified by using the 515F-806R primer set, and the amplicon library was prepared with Nextera adaptors. Subsequently, PCR fragments were sequenced by MiSeq Illumina sequencing (96 samples per lane, 2 × 300 bp 5 GB raw data per lane).

The results showed that the impact of DON and FBs on the intestinal microbiota is species- and age-dependent. Mycotoxin exposure modulated the abundance of several members of the family of *Lachnospiraceae, Ruminococcacceae*, *Peptococaceae*, *Coriobacteriaceae*, *Bifidobacteriaceae*, *Clostridiaceae*, and *Veillonellaceae.* Several of the affected microbial genera are involved in the production of short-chain fatty acids such as butyric acid.

### 7.35. Biosynthesis of the Hydroxamic Acid Aspergillic Acid in Toxigenic Fungus Aspergillus Flavus

Lebar M.D. ^1,^*, Cary J. ^1^, Carter-Wientjes C. ^1^, Mack B. ^1^, Majumdar R. ^1^, Di Mavungu J.D. ^2^ and De Saeger S. ^2^

^1^ Southern Regional Research Center, ARS-USDA, New Orleans, LA, USA

^2^ Laboratory of Food Analysis, Department of Bioanalysis, Faculty of Pharmaceutical Sciences, Ghent University, 9000 Ghent, Belgium

* Correspondence: matthew.lebar@ars.usda.gov

**Abstract:**
*Aspergillus flavus* produces aflatoxins, which are toxic and carcinogenic secondary metabolites, and can colonize important food staples. In silico analysis of the *A. flavus* genome revealed 56 gene clusters encoding for secondary metabolites. We are interested in how these metabolites affect fungal development, survival, and virulence. We are particularly interested in *A. flavus* metabolites that are produced during infection of maize seed. RNA-Seq analysis of all predicted *A. flavus* secondary metabolic gene cluster ‘backbone’ genes during maize kernel infection showed that in addition to the aflatoxin cluster polyketide synthase (PKS) gene, *aflC*, one of the earliest genes expressed was the uncharacterized Cluster 11 nonribosomal peptide synthetase (NRPS) gene, *asaB* (AFLA_023020). We focused on five genes in Cluster 11, which encode the putative NRPS, desaturase/hydroxylase (AFLA_023010), P450 oxidoreductase (AFLA_023030), MFS transporter (AFLA_023050), and C6 transcription factor (AFLA_023040). LC-MS analysis of extracts from knockout mutants of these genes showed that they were responsible for the synthesis of the previously characterized antimicrobial mycotoxin aspergillic acid. Extracts of the NRPS knockout showed no production of aspergillic acid or its precursors. Knockout of the P450 oxidoreductase afforded a pyrazinone metabolite, the aspergillic acid precursor deoxyaspergillic acid. The formation of hydroxyaspergillic acid was abolished in the desaturase/hydroxylase knockout. The antimicrobial properties of aspergillic acid are attributed to its ability to chelate iron as defense against competing organisms. Iron chelation, which generates the red pigment ferriaspergillin, could also be used offensively, as a virulence factor aiding the fungus in colonizing maize kernels.

### 7.36. The Understanding of OTA Biosynthesis: New Insight from Aspergillus Carbonarius

Gallo A. ^1,^*, Ferrara M. ^2^, Gambacorta L. ^2^, Epifani F. ^2^, Solfrizzo M. ^2^, Logrieco A.F. ^2^ and Perrone G. ^2^

^1^ National Research Council, Institute of Sciences of Food Production, Lecce, Italy

^2^ National Research Council, Institute of Sciences of Food Production, Bari, Italy

* Correspondence: antonia.gallo@ispa.cnr.it

**Abstract:** The increasing availability of fungal genomes and bioinformatic tools have led to the identification of clusters of known metabolites and to the prediction of novel cryptic clusters for still unknown metabolites. However, most of the clusters identified by genome analysis are still to be deeply examined to completely understand the pathway steps and the regulatory network behind the metabolite biosynthesis [1]. The genome sequencing of *Aspergillus carbonarius* has advanced the knowledge of the molecular mechanism of biosynthesis of ochratoxin A (OTA), one of the most important mycotoxin contaminating several commodities. Differently from other mycotoxins, the elucidation of the genetic background of OTA biosynthesis has remained uncompleted for a long time. *Aspergillus carbonarius* is the major responsible of OTA contamination of wine and other grape products in the Mediterranean area, constituting a great health risk and cause of important economic losses [2]. The analysis of *A. carbonarius* genome has revealed the presence of a great number of PKSs and NRPSs, enzymes having an essential role in the synthesis of fungal secondary metabolites. Subsequently, the identification of the PKS putatively involved in the biosynthesis of OTA has led to an extensive study of the adjacent genomic region, in the attempt to identify other genes involved and to define the OTA biosynthesis cluster. The roles of three key genes—*AcOTApks, AcOTAnrps* and *AcOTAhal*—have been demonstrated by gene knock-out approach and the order of the fundamental enzymatic steps in the biosynthesis pathway of OTA has been clarified. These studies demonstrated that the enzymatic step involving the addition of phenilalanine to the polyketide ring takes place before the chlorination step. Moreover, it was demonstrated that OTα is not a precursor of OTA but rather a product of OTA hydrolysis [3,4]. Other predicted genes in the cluster need to be further investigated to fully clarify the structural and regulatory mechanisms of toxin production, among which the genes coding a p450 monooxygenase, a transcription factor, a transporter protein and an aspartyl protease. Transcriptomic analyses are in progress to study and clarify at a deeper level the complex genetic picture of the fungus during OTA biosynthesis.

## References

Brakhage A.A. Nature Reviews Microbiology **2013**, *11*, 21–32.Perrone G. et al. *Aspergillus in the Genomic Era*; Academic Publishers: Wageningen, The Gelderland, 2008; pp. 179–212.Gallo A. et al. *Appl. Environ. Microbiol*. **2012**, *78*, 8208–8218.Ferrara M. et al. *Appl. Environ. Microbiol.*
**2016**, *82*, 5631–5641.

This activity is in progress within the MycoKey-Project H2020-(E.U.3.2-678781).

### 7.37. The Velvet Complex in Blue Mold Fungus Penicillium Expansum: Impact of Vea on Development, Aggressiveness and Secondary Metabolism

Assaf C.E.H., Snini S., Bailly S., Lippi Y., Jamin E., Martin J-F., Oswald I., Lorber S. and Puel O. *

Toxalim (Research Centre in Food Toxicology), Université de Toulouse, INRA, ENVT, INP-Purpan, UPS, Toulouse, France

* Correspondence: olivier.puel@inra.fr

**Abstract:**
*Penicillium expansum*, the principal agent of the blue mold disease, is a destructive phytopathogen causing decay in apples during post-harvest handling and storage. It is considered as a major concern due to its wide occurrence and capacity to produce citrinin and patulin, two well-known mycotoxins. As other major mycotoxins, patulin and citrinin are the final products of enzymatic cascades where enzymes are activated at the same time and the new synthesized products are consecutively metabolized by the next enzymes. This phenomenon is made possible thanks to the cluster organization of genes encoding for enzymes involved in the biosynthesis. These genes are often co-activated by a specific transcription factor located inside clusters. Although most of the studies on *P. expansum* have focused on patulin, the genome of this fungus exhibits other predicted secondary metabolite clusters, based on bioinformatics analysis and other specific studies. In filamentous fungi, the activation of specific transcription factor and by consequence the production of fungal secondary metabolites is controlled, at a higher hierarchical level, by transcription global factors encoded by genes not located in gene clusters. These genes regulate numerous physiological processes, and generally respond to several environmental cues. Among them, the VeA factor is a component of the “velvet complex”, a protein complex responding to abiotic factors, and more particularly to light. Depending on fungal species, VeA is involved in different physiological processes such as development, asexual and sexual reproduction, secondary metabolism and virulence. How *veA* affects the virulence of *P. expansum* on apples and the secondary metabolism with special emphasis on the production of important secondary metabolites (patulin and citrinin) is still unknown.

To address this question, a null Pe∆*veA* mutant and a complemented Pe∆*veA:veA* strain were generated in *Penicillium expansum*. Pathology studies performed on Golden Delicious cultivar indicated that the null mutant could still infect apples but with a lower growth rate as compared to the wild type and complemented strains. Disruption of *veA* caused a quasi-inability to produce patulin and citrinin on synthetic media (MEA and PDA) with a drastic decrease in the expression of patulin (*patA*-*patO*) and citrinin (Pexp_005510-Pexp_005590) genes. Moreover, the null mutant was not able to produce patulin when it grew in apples. The study was extended to the whole *P. expansum* secondary metabolism by the evaluation of the impact on VeA on the expression of the backbone genes of secondary metabolites found in the genome of the *P. expansum* d1 strain including PKSs, NRPSs and DMATSs genes and by a non-targeted LC-MS analysis. Metabolomic analysis displayed a global impact on secondary metabolism whilst the gene expression analysis showed a positive or negative regulation of 15/30 backbone genes. Our findings support the hypothesis that *P. expansum* secondary metabolism is modulated by the transcriptional regulator factor VeA contributing in part to the pathogenicity of the fungus.

### 7.38. Kinetics of TRI Gene Expression and T-2 and HT-2 Production by Fusarium Langsethiae

Verheecke-Vaessen C. *, Medina A. and Magan N

Applied Mycology Group, School of Water, Energy and Environment, Cranfield University, Cranfield MK43 0AL, Bedfordshire, UK

* Correspondence: c.verheecke@cranfield.ac.uk

**Abstract:** Since the species was named in 2004, *Fusarium langsethiae* has been found to be an increasing problem in Nordic countries, Central Europe and the UK/Ireland (Edwards et al., 2009; Imathiu et al., 2010; Torp and Nirenberg, 2004). This symptom-less fungus can lead to high amounts of T-2 and HT-2 contamination in oats, barley and wheat. T-2 toxin is considered the most toxic trichothecene and has immunosuppressive effects (Li et al., 2011; Rocha et al., 2005). Previous in vitro studies have shown optimum production of these two toxins after 10 days at 25 °C and 0.98 a_w_ on oat-based medium (Medina and Magan, 2011, 2010).

The phenotipic production of T-2 and HT-2 toxins is mainly triggered by the co-expresion of genes in three separate loci (*Tri5*, *Tri101* and *Tri1-Tri16* clusters). However, currently very few studies have been conducted on the kinetics of T-2 and HT-2 production and none on its associated gene expression by *F. langsethiae*. The objective of this study was thus to follow the growth, toxin production and expression of key *TRI* genes by RT-qPCR by *F.langsethiae* grown on oats-based media.

To develop the methodology, the recently sequenced *F.langsethiae Fl201059* was chosen for this study (LysØe et al., 2016). Different reference genes were tested including β-tubulin and citrate synthase as proposed by Ferruz et al. (2016). The best candidates as reference genes were chosen according to the best gene pairwise variation (V) and the best V of gene with other gene (M) values using geNorm. The gene expression of 4 structural genes (*TRI5*, *TRI7*, *TRI8*, *TRI16)* and two regulation genes (*TRI6*, *TRI15)* were studied to elucidate the kinetic pattern of gene expression in this species for 5 days. This was correlated with relative type A trichothecenes toxin production where possible. These results will help decipher the kinetics of various key genes involved in the biosynthesis of T-2 and HT-2 production in oat matrices. Those results will enable more detailed ecophysiological and molecular ecological studies to be carried out for developing minimisation strategies for these toxins in small grain cereals.

## References

Edwards, et al. *World Mycotoxin J*. **2009**, *2*, 173–179.Ferruz, et al. *Molecules*
**2016**, *21*, 449.Imathiu, et al. *Eur. J. Plant Pathol.*
**2010**, *126*, 203–216.Li, at al. *J. Agric. Food Chem.*
**2011**, *59*, 3441–3453.LysØe, et al. *Int. J. Food Microbiol.*
**2016**, *221*, 29–36.Medina and Magan. *Food Microbiol.*
**2011**, *28*, 392–398.Medina and Magan. *Int. J. Food Microbiol.*
**2010**, *142*, 365–369.Rocha, et al*. Food Addit. Contam.*
**2005**, *22*, 369–378.Torp and Nirenberg. *Int. J. Food Microbiol*. **2004**, *95*, 247–256.

### 7.39. Study of the Correlation between Aflatoxins B1 (AFB1) Levels and Mutation in the P53 Gene in Pakistani Patients Suffering from Liver Diseases inCluding Hepatocellular Carcinoma

Saleemi M.K. ^1,^*, Kanchanakhan N. ^2^, Pumpaibool T. ^2^, Siriwong W. ^2^, Khan A. ^1^, Robson M. ^3^, Khan M.Z. ^1^, Fayyaz A. ^1^, Naseem M.N. ^1^, Khatoon A. ^1^, Imran M. ^1^ and Abdullah I. ^4^

^1^ Department of Pathology, University of Agriculture Faisalabad, Pakistan

^2^ College of Public Health Sciences, Chulalongkorn University Bangkok, Thailand

^3^ Rutgers University, Newgersy, USA

^4^ PINUM, Cancer Hospital, Faisalabad Pakistan

* Correspondence: drkashif313@gmail.com

**Abstract:** Hepatocellular carcinoma (HHC) is one of the most important and common tumor in Pakistan. The cases of HCC have been continuously increasing in the country for the past few years. Among others, dietary exposure to aflatoxins B1 (AFB1) was described as one of the major HCC risk factors. Multiple genetic mutations and epigenetic changes are related with molecular pathogenesis of HCC. One of the mutations that may be correlated with higher incidence of HHC is on the *P53* tumor suppressor gene. The present study was designed to investigate the role AFB1 contaminated foods in the development of hepatocellular carcinoma in patients suffering from liver diseases including HCC. For this study, a total of 150 human blood and serum samples were collected from healthy donors and patients suffering from liver diseases including HCV and HBV attending care in hospital in the Faisalabad district of Pakistan. These blood samples were centrifuged to separate its constituents for further processing. . The serum was then used for the AFB1 quantitative analysis and the blood cells were used for DNA extraction using DNA extraction kit. PCR were performed to amplify the *P53* (tumor suppressor) gene variants in order to study the relation between AFB1 levels and P53mutations. In all samples, the PCR results showed a 254 bp product size from exon 7 for the *P53* gene. To study mutations in the *P53* gene, restriction analysis was performed by using Hae III restriction enzyme. In case of mutation in the *P53* gene in correlation with the presence of AFB1, a DNA product of 158 bp should be present, while in case of no mutation the enzymatic digestion will result in the production of two products of 92 bp and 66 bp. The restriction analysis indicated no mutation presumably related to AFB1 in the *P53* gene of the Pakistani population. The present study concluded that AFB1 was detected in the serum of individuals but that no mutation in the *P53* gene of these individuals was present.

### 7.40. Multi-Mycotoxin Analysis in Blood & Urine of Five European Populations: Characterizing Internal Dietary Mycotoxin Exposure in the EFCOVAL Cohort

De Ruyck K. ^1,2,^*, Ejedepang D.B.K. ^3^, Moerman J. ^4^, Said M. ^5^, De Boevre M. ^1,2^, Huybrechts I. ^6^ and De Saeger S. ^1,2^

^1^ Laboratory of Food Analysis, Ghent University, Ghent, Belgium

^2^ Cancer Research Institute Ghent (CRIG), Ghent, Belgium

^3^ Center for Statistics, Hasselt University, Hasselt, Belgium

^4^ Faculty of Pharmaceutical Sciences, Ghent University, Ghent, Belgium

^5^ Faculty of Medicine, Uppsala University, Uppsala, Sweden

^6^ International Agency for Research on Cancer (IARC), Lyon, France

* Correspondence: karl.deruyck@ugent.be

**Abstract:** Mycotoxin exposure of human populations is prevalently estimated in Europe by intersecting self-reported dietary consumption data with mycotoxin occurrence data from the European Food Safety Agency (EFSA). One database of these estimates was produced covering the European Food Consumption and Validation (EFCOVAL) research population. To assess the validity of this estimation method, and to further investigate the heterogeneity of internal mycotoxin exposure, plasma and urine from sample populations representing Belgium (*n* = 123), the Czech Republic (*n* = 118), France (*n* = 115), the Netherlands (*n* = 122), and Norway (*n* = 125) were assayed for 46 mycotoxins and metabolites. An optimized adaptation of salt-assisted liquid-liquid extraction (SALLE) was used to prepare biological fluids for analysis. The analysis itself was performed by an ultra performance liquid chromatographic and tandem mass spectrometric (UPLC-MS/MS) method on a Waters Acquity system coupled to a Waters Xevo TQ-S. Chromatographic separation was achieved using a Waters HSS T3 column, which uses a C18 stationary phase with proprietary end-capping for compatibility with both polar and non-polar analytes. This broad range of retention enabled simultaneous analysis of diverse mycotoxin classes, including aflatoxins, ergot alkaloids, fumonisins, ochratoxins, zearalenone, common trichothecenes such as deoxynivalenol, diacetoxyscirpenol, HT-2 toxin, and T-2 toxin, as well as some of these mycotoxins’ known major metabolites. Detailed results of the analysis will be discussed during this presentation.

### 7.41. Research on the Glucuronidation and Renal Excretion of Deoxynivalenol in Humans

Vidal A. ^1,^*, Claeys L. ^1^, de Boevre M. ^1^, Mengelers M. ^2^ and de Saeger S. ^1^

^1^ Laboratory of Food Analysis, Department of Bioanalysis, Faculty of Pharmaceutical Sciences, University of Ghent, Ghent, Belgium

^2^ Department of Food Safety, Centre for Nutrition, Prevention and Health Services, National Institute for Public Health and the Environment (RIVM), Bilthoven, the Netherlands

* Correspondence: arnau.vidalcorominas@ugent.be

**Abstract:** Deoxynivalenol (DON) is a frequently occurring mycotoxin in cereals and cereal products, consequently DON is one of the most common mycotoxin in our diets. Dietary intake studies showed high exposure(s) of consumers to this toxin with, in some countries like Belgium, appreciable percentages of (sub)populations exceeding the tolerable daily intake (TDI). Reliable biomarkers of exposure are DON and its glucuronide conjugates, which are the most common metabolites of DON found in the urine. Therefore, the analysis of DON-glucuronides in urine is crucial for the study of trichothecenes biomarkers, because a high percentage of DON excreted via urine is the glucuronide form. For the determination of glucuronides, a preliminary approach was developed based on the deglucuronidation of DON-glucuronides and subsequent determination of “total DON” (sum of free and released mycotoxins after hydrolysis). Afterwards, a direct method for quantification of glucuronides such as deoxynivalenol-3-glucuronide and deoxynivalenol-15-glucuronide, which are the more common glucuronides forms, was developed. Recently, correlations between the sum of urinary DON and its glucuronidated metabolites, and the DON intake have been found and applied in several studies. These investigations revealed the power of biomarker-driven work when compared to traditional dietary exposure assessment by analyzing foodstuffs. However, the absorption and elimination (metabolism and excretion) of DON in humans have not been studied quantitatively, so far. In addition, the high presence of DON conjugates in food like DON-3-glucoside or acetyl-deoxynivalenol add more uncertainties for the correlation between urinary DON and DON intake. Therefore, an intervention study was performed, and 20 volunteers (55% women and 45% men) were submitted to a DON-free diet for three days. After two days they received a single dose of DON (1 μg/kg body weight/day), and urine was collected for 24 h. The aims of this research were to study the absorption and elimination of DON, and to establish appropriate biomarker(s) of DON exposure. Consequently, the results can be used to estimate DON-intake by means of its biomarker(s).

### 7.42. Post-Harvest Interventions Decrease Infants’ Dietary Exposure To Aflatoxins And Fumonisins in Tanzania: A Cluster Randomized Controlled Trial

Kamala A. ^1,2,^*, Kimanya M. ^3^, Magoha H. ^4^, De Meulenaer B. ^1^, Kolsteren P. ^1^, Jacxsens L. ^1^, Haesaert G. ^5^, Kilango K. ^2^, Tiisekwa B. ^6^ and Lachat C. ^1^

^1^ nutriFOODchem Unit, Department of Food Safety and Food Quality, Partner in Food2Know, Faculty of Bioscience Engineering, Ghent University, Coupure Links 653, 9000 Ghent, Belgium

^2^ Directorate of Food Safety, Tanzania Food and Drugs Authority, P.O. Box 77150, Dar es Salaam, Tanzania

^3^ School of Life Sciences and Bio-Engineering, The Nelson Mandela African Institution of Science and Technology (NM-AIST), P.O. Box 447, Arusha, Tanzania

^4^ Department of Home Economics and Human Nutrition, Open University of Tanzania (OUT), P.O. Box 23409, Dar es Salaam, Tanzania

^5^ Department of Applied Biosciences, Faculty of Bioscience Engineering, Ghent University, Valentin Vaerwyckweg 1, BE-9000 Ghent, Belgium

^6^ College of Agriculture, Sokoine University of Agriculture, P.O. Box 3005, Morogoro, Tanzania

* Corresponding Author: analice.kamala@ugent.be

**Abstract:** A cluster randomized controlled trial was performed in three main maize producing agro-ecological zones of Tanzania to evaluate the efficacy of locally available post-harvest mitigation strategies in reducing infants’ dietary exposure to aflatoxins and fumonisins. A total of 300 (pairs of mother and child) participants were randomized to 15 intervention and 15 control villages. The interventions consisted of application of post-harvest techniques to reduce aflatoxin and fumonisin contamination in maize for the period of six months after harvest. The control group received routine agricultural extension services on good practices for handling crops. Fumonisin and aflatoxin contamination in breast milk and food samples were analyzed using HPLC techniques. Six months follow-up data were available for 261 households. The intervention significantly (*p* < 0.05) decreased aflatoxin B1, total aflatoxin and total fumonisin levels in maize: intervention effect μg kg^−1^ (95% CI) = −3.5 (−5.2,−2.0), −4.9 (−7.3,−2.5), and −405, (−647,−162) for aflatoxin B1, total aflatoxins and total fumonisins, respectively. Mean level of aflatoxin M1 in breast milk decreased significantly (*p* = 0.013), group difference −0.024 ng mL^−1^ (95% CI, −0.045, −0.004). The proportion of infants at risk of exposure to mycotoxin above 0.017 ng kg^−1^ bw day^−1^ and 2 μg kg^−1^ bw day^−1^ for aflatoxins and fumonisins respectively was significantly reduced (*p* < 0.05) by 20%, 22% and 36% for aflatoxin B1, total aflatoxins and total fumonisins, respectively. Prevalence of underweight was 6.7% significantly lower (*p* = 0.014) in the intervention group and group mean WAZ difference was 0.57 (0.16, 0.98; *p* = 0.007). Locally available post-harvest practices are effective in reducing the risk of infant’s dietary exposure to aflatoxins and fumonisins in rural Tanzania. Trial registration: ClinicalTrials.gov identifier: NCT02438774.

### 7.43. Effect of Groundnut Drying Methods on Drying Rate and Aflatoxin Contamination

Chimbaza M. *, Mwangwela A.M. and Kamthunzi W.

Department of Food science and Technology, Lilongwe University of Agriculture and Natural Resources, Malawi

* Correspondence: mchimbaza@gmail.com

**Abstract:** Groundnut or peanut (*Arachis hypogaea* L.), is an important food and fodder crop in the farming systems of developing countries like Malawi. Despite being a cash crop, groundnuts are highly recognized as sources of protein (23–25% content), fat (40–50%), oil (40–52% content), and carbohydrate (10–20%) depending on the variety. A major concern in groundnut production is aflatoxin contamination which negatively affects trade and wellbeing of humans. Aflatoxin contamination which is due to secondary metabolites of *Aspergillus flavus* and *A. parasiticus* may occur at any stage in the value chain; however, the drying stage is proven to be a critical stage of aflatoxin management. Therefore, this study evaluated twelve methods of drying groundnuts and their effect on the drying rate and aflatoxin contamination.

The trials were conducted on farm and off farm and drying methods were allocated randomly in a split-split plot design. Moisture content, temperature and relative humidity were recorded each day during the drying period in order to estimate drying rates. After drying was completed (less than 7% moisture content on each treatment) aflatoxin analysis was done using ELISA method.

Results indicated that there was no significant different in drying rates among groundnuts that were dried using off farm drying methods. It was discovered that off farm methods of drying dries groundnuts faster as compared to on farm drying methods. A significant difference in drying rates among on farm drying methods was observed. In terms of aflatoxin contamination; groundnuts which were dried on Windrows, racks and bare ground showed the highest levels of contamination, this is consistent with the findings of Hell et al., 2008, were grains which where dried on bare ground were reported to be more contaminated with aflatoxin (41.7%) than those dried on sheets or mats (25%). Drying on bare ground and windrow is not recommended since the crop is persistently exposed to soil contamination which is the source of fungi.

In conclusion, the study rejected the null hypothesis that there is no difference in drying rate and aflatoxin level of contamination, among groundnuts dried under different methods of drying. Off farm methods of drying dries groundnuts faster than on farm drying methods; however, fast drying negatively affect quality of groundnuts. Mandela and A frame methods of drying have been proven as best methods of controlling aflatoxin.

### 7.44. Fusarium (Modified) Mycotoxins in Nigerian Sorghum-Based Beer: Natural Occurrence and Influence of Traditional Processing Methods

Chilaka C.A. ^1,2,^*, De Boevre M. ^1^, Atanda O. ^3^ and De Saeger S. ^1^

^1^ Laboratory of Food Analysis, Department of Bioanalysis, Faculty of Pharmaceutical Sciences, Ghent University, Ghent, Belgium

^2^ Department of Food Science and Technology, College of Applied Food Science & Tourism, Michael Okpara University of Agriculture Umudike, Abia State, Nigeria

^3^ Department of Biological Sciences, McPherson University, Seriki Sotayo, Ogun State, Nigeria

* Correspondence: cynthia.chilaka@ugent.be

**Abstract:**
*Fusarium* fungi are common pathogens that cause several plant diseases and also produce mycotoxins. These mycotoxins have been implicated to cause a variety of toxic effects in humans and animals ranging from acute to chronic. Mycotoxins may co-occur and cause synergistic and/or additive health effects on the host. The co-occurrence of *Fusarium* mycotoxins with structurally-related compounds (modified mycotoxins) has recently become a food safety concern. To date, little information is known about the *Fusarium* mycotoxins in the Nigerian food system. The present study reports the natural occurrence of *Fusarium* (modified) mycotoxins in Nigerian sorghum-based beer (*burukutu* and *pito*). A total of 99 samples of *burukutu* (*n* = 54) and *pito* (*n* = 45) were collected from randomly selected markets in Nigeria, and analysed and quantified for *Fusarium* mycotoxins using a multi-mycotoxin liquid chromatography-tandem mass spectrometric (LC-MS/MS) method. Results revealed the occurrence of *Fusarium* mycotoxins in the samples with deoxynivalenol (DON) being the most dominant in *burukutu* and *pito* at the rate of 63% (range: 25–42 μg/L) and 69% (range: 28–49 μg/L), respectively. Co-occurrence of mycotoxins was observed in the products, with DON and DON-3-glucoside (*burukutu,* rate: 14%; *pito*, rate: 20%) being the most co-occurred mycotoxins in the two products.

To further gain a better understanding on the fate of *Fusarium* mycotoxins during the production of *burukutu* and *pito*, a laboratory-scale study on the influence of traditional processing methods on *Fusarium* mycotoxins was carried out. In this context, free mycotoxins including DON, fumonisin B_1_, zearalenone, T-2 toxin, and their metabolites were the main focus. Preliminary results showed a significant reduction (˃60%) of the free mycotoxins in *burukutu* and *pito*, however the reverse trend was observed for their metabolites, especially hydrolysed fumonisin B_1_ and DON-3-glucoside.

### 7.45. Synergic Potential of Pre-Milling and Milling Strategies to Minimize Mycotoxins and Increase Fiber Content of Wheat-Based Products

Suman M. ^1,^*, Tribuzio G. ^2^ and Arlotti G. ^3^

^1^ Barilla Advanced Laboratory Research, via Mantova 166, 43122 Parma, Italy

^2^ Barilla Agronomy Research, via Mantova 166, 43122 Parma, Italy

^3^ Barilla Milling Process Development, via Mantova 166, 43122 Parma, Italy

* Correspondence: michele.suman@barilla.com

**Abstract:** Several clinical studies from around the world show that a daily consumption of whole grain components can reduce the risk of cardiovascular disease and the development of diabetes, in addition also to a reduction in the risk of cancer and mortality.

Producers must commit to guarantee a high level of safety of whole grain food products, while developing creative recipes and food palatable proposals that encourage the public to use them.

In fact, at the same time food based on grains (e.g., pasta, bread, bakery products) account for the largest contribution to mycotoxin exposure in all age classes, in particular due to the mycotoxins produced by *Fusarium* spp.

Within post-harvest interventions devoted to minimize mycotoxins impact with respect to diet intake, one of the first effective actions is to integrate novel down-stream processing approaches.

Cleaning, debranning, peeling, soaking, dry- or wet- fragmentation, air separation etc. are efficient, proven and versatile, cost effective methods for companies to achieve high quality particle size reduction results and to allow more accurate separation of grain tissues with characterized different mycotoxin contamination levels.

This presentation will illustrate how the synergic potential of these pre-milling and milling strategies permit to achieve an accurate separation of grain tissues with different mycotoxin levels, detailing the composition of the internal tissues, minimizing the mycotoxin concentration and increasing contemporaneously the overall fiber contents in raw material selected fractions to be then (re-)combined for final safer wheat products destined for consumers.

In particular, in a close future scenario, bread and pasta could be then added with increased fiber content, up to 10% according to technological requirements.

### 7.46. Occurrence of Potentially Toxigenic Heat Resistant Moulds in Pasteurized High Acid Fruit Products

Santos J.L.P. ^1,^*, Samapundo S. ^1^, Höfte M. ^2^, Van Impe J. ^3^, Sant’Ana A.S. ^4^ and Devlieghere F. ^1^

^1^ Laboratory of Food Microbiology and Food Preservation, Department of Food Safety and Food Quality, Bioscience Engineering Faculty, Ghent University, Ghent, Belgium

^2^ Laboratory of Phytopathology, Department of Crop Protection, Bioscience Engineering Faculty, Ghent University, Ghent, Belgium

^3^ Chemical and Biochemical Process Technology and Control (BioTec), Department of Chemical Engineering, University of Leuven, Ghent, Belgium

^4^ Laboratory of Quantitative Food Microbiology, Department of Food Science, Food Engineering Faculty, University of Campinas, Campinas, Brazil

* Correspondence: juliana.lanepsantos@ugent.be

**Abstract:** The occurrence of heat resistant moulds (HRMs) in pasteurized high acid fruit products has been reported for many years. HRMs are often associated with spoilage incidents of these products. In addition to their ability to resist pasteurization processes (80–95 °C), and their ability to grow at very low pH values (3.0–4.0), several species of HRMs pose a risk to health due to mycotoxin production. Species of HRMs belonging to *Byssochlamys*, *Neosartorya*, *Talaromyces* and *Eupenicillium* genera which are associated with high acid fruit products are known to produce many toxigenic secondary metabolites such as byssotoxin A, byssochlamic acid, patulin, fumitremorgin A and C, verruculogen, fischerin, eupenifeldin, cytoxicity and aflatoxin B. However, very little information is available about the contamination degree which is essential information to perform quantitative risk assessments. Therefore, the aim of this study was to assess the occurrence (qualitatively and quantitatively) of potentially toxigenic HRMs in high acid fruit products. A total of 332 samples were analyzed from three processing plants: strawberry purée (*n* = 88), concentrate orange juice (*n* = 90) and apple purée (*n* = 154). HRMs were detected in heat treated (80 °C, 30 min) samples (100 g), thoroughly mixed with the molten Malt Extract Agar, distributed into Petri dishes and incubated at 30 °C for up to 30 days. The results were expressed as cfu/100 g. HRMs were then purified and identified morphologically to the genus level, following the identification keys proposed by Pitt and Hocking (2009) and Samson et al. (2010). Thereafter the HRMs were identified at the species level by means of gene sequencing. Following DNA extraction, PCR amplification and purification procedures, sequencing was done by LGC Genomics GmbH (Berlin, Germany) and identification using BLAST analysis and phylogenetic analysis. Aspergilli *Neosartorya*-type ascoma and *Byssochlamys* sp. were detected in samples from different fruit products and at different processing stages. The findings of this study may be used as an input for quantitative risk assessment of mycotoxins in pasteurized high acid fruit products.

### 7.47. Mycotoxin Bioremediation Via Laccase Mediator System

Loi M. ^1,2,^*, Fanelli F. ^1^, Liuzzi V.C. ^1^, Haidukowski M. ^1^, Cimmarusti M.T. ^1,2^, Logrieco A.F. ^1^ and Mulè G. ^1^

^1^ Institute of Sciences of Food Production, National Research Council of Italy (CNR), via Amendola 122/O, Bari 70126, Italy

^2^ Department of Economics, University of Foggia, via Napoli 25, Foggia 71122, Italy

* Correspondence: martina.loi@ispa.cnr.it

**Abstract:** The development of new, food grade and environmental friendly methods for mycotoxin reduction is a crucial and concrete struggle, especially with regards to food and feed supply chains. The application of biological methods for mycotoxin reduction is arising great interest in the scientific community since they allow to use mild conditions, to avoid the use harmful chemicals and to counteract the loss of the nutritional value or palatability of the detoxified material.

The degrading activity of two laccases from two edible fungi (*Pleurotus eryngii* and *P. pulmonarius*) towards aflatoxin B_1_ (AFB_1_), aflatoxin M_1_ (AFM_1_), fumonisin B_1_ (FB_1_), zearalenone (ZEN) and T2 toxin was evaluated [2]. Laccases were produced and purified to homogeneity and tested in in vitro degradation assays, performed in buffer solution (1 mM sodium acetate pH5). The effect of the laccase-mediator system was evaluated as well, by adding to the reaction natural and artificial redox mediators. Chemical analysis were performed after three days of static incubation at 25 °C. A significant reduction of all tested mycotoxins was measured in presence of a redox mediator, with degrading efficacies of 100% for AFM_1_ and ZEN, of 90% for AFB_1_ and of 40% for T2 toxin and FB_1_. *In matrix* degradation assays were performed for the first time in ZEN naturally contaminated maize flour and in AFM_1_ spiked milk. ZEN was reduced by 40%, while AFM_1_ was halved within few hours and completely degraded after three days.

The green feature, in vitro and *in matrix* effectiveness and multi mycotoxin degrading capability of laccase enzymes are the key attributes of a potential biotransforming agent. These results open new encouraging perspectives for mycotoxins bioremediation in the food and feed supply chains. To this aim, we plan to evaluate the toxicity of the degradation products, which is mandatory in order to develop a method for mycotoxins detoxification, through in vivo assays.

Studies are ongoing for the characterization of AFB_1_ degradation products by means of high resolution mass spectrometry analysis of ^13^C-labelled toxin. We are currently performing the optimization of reaction parameters through a multifactorial design approach, the evaluation of the degrading activity towards other toxins, such as patulin as well as the validation in other food matrices.

This work was financially supported by H2020-E.U.3.2-678781-MycoKey-Integrated and innovative key actions for mycotoxin management in the food and feed chain.

### 7.48. Application of Mycotoxin-Detoxifying Enzymes in The Bioethanol Process

Kotz D. ^1,^*, Rose S. ^1^, Schatzmayr D. ^1^, Trupia S. ^2^ and Schatzmayr G. ^1^

^1^ BIOMIN Research Center, BIOMIN Holding GmbH, Tulln, Austria

^2^ National Corn-to-Ethanol Research Center, Southern Illinois University Edwardsville, Edwardsville, IL, USA

* Correspondence: daniela.kotz@biomin.net

**Abstract:** The U.S. ethanol industry is continuously growing with a production of around 1 million barrels of ethanol per day in December 2016 (U.S. Energy Information Administration). During the production of 1 barrel of ethanol around 98 kg of Distiller Dried Grains with Solubles (DDGS) are produced (Bothast & Schlicher, 2005) which represents an interesting by-product for the feed industry. Feeding by-products of the bioethanol industry to livestock increases the likelihood of exposing animals to higher mycotoxin levels, as mycotoxins present in the raw materials (mainly corn) are not degraded but concentrated approximately threefold during the bioethanol production process. According to the latest results of the BIOMIN Mycotoxin Survey, all 79 U.S. DDGS samples tested were at least positive for one of the three major mycotoxins (deoxynivalenol, fumonisin or zearalenone) and 96% of the samples were contaminated with more than one mycotoxin. In the animal the mycotoxins can cause adverse health effects and impaired animal performance, implicating economic losses for the livestock industry.

Counter measures for the bioethanol producer so far comprise rejection of specific raw material batches showing high mycotoxin concentrations or blending raw material with high mycotoxin contamination levels with material low in mycotoxins. The application of mycotoxin-degrading enzymes directly in the bioethanol production process offers another way of diminishing the occurrence of mycotoxins in the production of biofuels and fermentation by-products. Lab-scale tests with mycotoxin degrading feed additives performed at the BIOMIN Research Center and the National Corn-to-Ethanol Research Center, Southern Illinois University Edwardsville, showed the degradation of fumonisin B_1_ (FB_1_) and the formation of the degradation product hydrolyzed FB_1_ proving the detoxification of the mycotoxin during the process. FUM*zyme*^®^ added either before liquefaction or before fermentation (10 U/kg corn and 100 U/kg corn) during the bioethanol production process with naturally contaminated corn (4879 ppb FB_1_) led to a ≥96% reduction of FB_1_ in the mash. By application of mycotoxin degrading additives directly in the bioethanol process high quality DDGS with low mycotoxin levels can be produced benefitting livestock producers and boosting bioethanol industry’s revenues.

Bothast & Schlicher, 2005. Biotechnological processes for conversion of corn into ethanol. Applied Microbiology and Biotechnology, 67(1), pp.19–25.

Weekly U.S. Oxygenate Plant Production of Fuel Ethanol. U.S. Energy Information Administration. https://www.eia.gov/dnav/pet/hist/LeafHandler.ashx?n=PET&s=W_EPOOXE_YOP_NUS_MBBLD&f=W (accessed on 13 February 2017).

### 7.49. Microbial Detoxification of Deoxynivalenol (don), Assessed Via a Lemna Minor l. Bioassay, through Biotransformation to 3-epi-don and 3-epi-dom-1

Vanhoutte I. ^1^, De Mets L. ^1^, De Boevre M. ^2^, Uka V. ^2^, Di Mavungu J.D. ^2^, De Saeger S. ^2^, De Gelder L.^1,^*^,†^ and Audenaert K. ^3,†^

^1^ Laboratory of Environmental Biotechnology, Department of Applied Biosciences, Faculty of Bioscience Engineering, Ghent University, 9000 Ghent, Belgium

^2^ Laboratory of Food Analysis, Department of Bioanalysis, Faculty of Pharmaceutical Sciences, Ghent University, 9000 Ghent, Belgium

^3^ Laboratory of Applied Mycology and Phenomics, Department of Applied Biosciences, Faculty of Bioscience Engineering, Ghent University, 9000 Ghent, Belgium

***** Correspondence: Leen.DeGelder@UGent.be

**Abstract:** Mycotoxins are toxic metabolites produced by fungi. To mitigate mycotoxins in food or feed, biotransformation is an emerging technology in which microorganisms degrade toxins into non-toxic metabolites. To monitor deoxynivalenol (DON) biotransformation, analytical tools such as ELISA and liquid chromatography coupled to tandem mass spectrometry (LC-MS/MS) are typically used. However, these techniques do not give a decisive answer about the remaining toxicity of possible biotransformation products. Hence, a bioassay using *Lemna minor* L. was developed. A dose–response analysis revealed significant inhibition in the growth of *L. minor* exposed to DON concentrations of 0.25 mg/L and higher. Concentrations above 1 mg/L were lethal for the plant. This bioassay is far more sensitive than previously described systems. The bioassay was implemented to screen microbial enrichment cultures, originating from rumen fluid, soil, digestate and activated sludge, on their biotransformation and detoxification capability of DON. The enrichment cultures originating from soil and activated sludge were capable of detoxifying and degrading 5 and 50 mg/L DON. In addition, the metabolites 3-epi-DON and the epimer of de-epoxy-DON (3-epi-DOM-1) were found as biotransformation products of both consortia. Our work provides a new valuable tool to screen microbial cultures for their detoxification capacity.

### 7.50. Mechanistic Insight into the Biosynthesis and Detoxification of Fumonisins

Sumarah M.W. ^1,^*, Renaud J.B. ^1^, Seifert K.A. ^2^ and Miller J.D. ^3^

^1^ Agriculture and Agri-Food Canada, London, Ontario, Canada

^2^ Agriculture and Agri-Food Canada, Ottawa, Ontario, Canada

^3^ Carleton University, Ottawa, Ontario, Canada

* Correspondence: mark.sumarah@agr.gc.ca

**Abstract:** Fumonisins are a group of economically important mycotoxins originally reported from *Fusarium* sp. and more recently from *Aspergillus*. The fumonisin scaffold is comprised of a polyketide backbone functionalized with two tricarballylic esters and an alanine derived amine. These functional groups are important for inhibition of sphingolipid biosynthesis in animals, plants, and yeasts. We reported for the first time the isolation and characterization of two classes of non-aminated fumonisins produced by *Aspergillus*. Using a *Lemna minor* (duckweed) bioassay, we then demonstrated that these new compounds were significantly less toxic in comparison to the equivalent animated fumonisins, confirming the previous speculation that the amine group was critical for toxicity. Time course fermentations monitoring fumonisin production indicated that a novel post-biosynthetic oxidative deamination was occurring. This enzymatic pathway was further supported by a feeding study with labelled FB_2_ that resulted in the replacement of the amine with a ketone. Work is underway to characterize this enzyme and to apply it to the detoxification of fumonisns. Additional research mapping all enzymes involved in fumonisin biosynthesis is providing new insight into their biosynthesis and an understanding as to why some strains with all of the FUM genes are non fumonisin producers.

### 7.51. Toxigenic Mould Respiration in Stored Cereals: Use of CO_2_ Production under Interacting Environmental Conditions on Dry Matter Loss and Mycotoxin Contamination as Tools for Post-Harvest Decision Support Systems

Garcia-Cela E., Kiatsi E., Medina A. and Magan N. *

Applied Mycology Group, Environment and AgriFood Theme, Cranfield University, MK43 0AL Cranfield, Bedfordshire, United Kingdom

* Correspondence: n.magan@cranfield.ac.uk

**Abstract:** Safe management of stored commodities requires the effective drying and monitoring of moisture content and temperature to avoid quality losses and potential contamination with mycotoxins. There is need to effectively monitor the stored commodities during short and medium term storage so that spoilage and nutritional losses can be minimized. At present, temperature (T) sensors and sometimes relative humidity (RH) sensors are used in silos to monitor quality and detect changes that can be related to spoilage and therefore quality deterioration on the grain due to mould growth and insect infestation. However, because grain is a good insulator changes in temperature occur slowly.

Most stored commodities respire naturally and produce CO_2_ which permeates the intergranular air spaces significantly more rapidly than changes in temperature in a grain bulk. Thus CO_2_ may provide a better early warning system than temperature to facilitate real time management of stored commodities. We have thus examined the relationship between respiration of wheat and maize grain stored under different water activity x temperature conditions inoculated with *Fusarium graminearum* and *Aspergillus flavus* respectively. The CO_2_ production changes have been used to calculate the relative dry matter losses and this has been related to the production of zearalenone and aflatoxin B_1_ respectively in these two cereals. This provides information on whether the EU legislative limits are being exceeded or not during short and medium term storage. By combining real time detection of CO_2_ in grain silos and linking this to biological models of boundary conditions for growth and mycotoxin production and to the DML/mycotoxin models, it will be possible to develop post-harvest Decision Support Systems for minimizing contaminated batches.

### 7.52. Controlling Mycotoxin Contamination in Maize Silages: A Survey of Flemish Maize Parcels

Vandicke J. *, Audenaert K. and Haesaert G.

Department of Applied Biosciences, Faculty of Bioscience Engineering, Ghent University, Ghent, Belgium

* Correspondence: jonas.vandicke@ugent.be

**Abstract:** Mycotoxins are toxic secondary metabolites produced by a variety of fungal species, such as Fusarium, Penicillium or Aspergillus, among others. Contamination of feed with mycotoxins can cause severe health problems in dairy cattle. Especially high yielding dairy cows with a high feed uptake and rapid ruminal flow are susceptible to gastroenteritis, reduced reproduction and reduced milk production, as a result of mycotoxin contamination. Maize silage is one of the main components of dairy feed in the region of Flanders, Belgium, and is therefore one of the main sources for mycotoxin uptake in dairy cows.

This research aims towards providing dairy farmers in Flanders with a user-friendly prediction model, able to foresee mycotoxin contamination based on weather, cultivation, harvest and silage conditions. This model will be constructed based on mycotoxin analyses of freshly harvested maize and maize silages across Flanders, and on research focusing on methods to prevent mycotoxin contamination.

During maize harvest in 2016, 91 samples were taken of Flemish maize parcels. These samples were analyzed for 23 different mycotoxins using LC-MS/MS. The results of these first 91 field samples indicate that nearly every maize parcel in Flanders contains at least one of the 23 mycotoxins analyzed. NIV was the most prevalent one, being present in all but one sample. DON and ZEA were found in 84 and 59 samples, resp. Concentrations went up to 2368 ppb for NIV and 2777 ppb for DON. No aflatoxins or fumonisins (except FB1 in 2 samples) were found. These results were then used to search for correlations in the concentrations of different mycotoxins, and to detect certain agronomic practices that are correlated with a higher or lower mycotoxin concentration. ZEN was found to be correlated with the presence of NIV and ENN B. DON and NIV showed little correlation. Agronomic practices that were investigated were crop rotation, tillage, fertilization, soil type, dry matter content and sowing density, among others. Further sampling is needed to validate the results of the first sampling year.

### 7.53. Trend Analyses and Modeling for Risk-Based Control of Mycotoxin in Feed Ingredients

van der Fels-Klerx H.J. *, Adamse P. and De Jong J.

Wageningen University and Research Centre (RIKILT), Wageningen, The Netherlands

* Correspondence: ine.vanderfels@wur.nl

**Abstract:** According to Regulation (EC) No 882/2004, Member States in Europe should establish and implement multi-annual control programs for contaminants in feed and food, to ensure that checks are regular and proportional to the risk. In the Netherlands for mycotoxins in feed and feed materials trend analyses and modelling are performed with the aim to obtain insights into which mycotoxin-feed materials are of highest concern. The combined results from the trend analyses and the model are used for setting priorities for the multi-annual control program for mycotoxins in feed and feed materials.

Using public and private data on the presence of mycotoxins in animal feed and feed materials, trend analyses are performed on the presence of the mycotoxin in specific feed materials over a period of several years. The model (named “RiskFeedModel”) for risk based monitoring ranks, per mycotoxin, the various feed materials based on their risk for animal and human health related to the presence of the particular mycotoxin in each ingredient. The model uses: imported volumes of feed ingredients, country of origin, used volumes of feed ingredients per animal type, compound feed composition per animal type, presence of the toxin in each ingredient, the consequence of the presence of the mycotoxin on animal and human health.

During the conference, the results of the trend analyses and model for the mycotoxins aflatoxin B1 and deoxynivalenol, and the implementation in the Dutch multi-annual control program, will be presented.

### 7.54. Three Modelling Approaches to Predict Deoxynivalenol Contamination Levels in Winter Wheat in The Netherlands

Liu C. ^1^, Manstretta V. ^2^, Rossi V. ^2^ and van der Fels-Klerx I. ^1,^*

^1^ Wageningen University & Research (RIKILT), Akkermaalsbos 2, 6708 WB Wageningen, The Netherlands

^2^ Department of Sustainable Crop Production (DI.PRO.VE.S), Università Cattolica del Sacro Cuore, via Emilia Parmense 84, 29122 Piacenza, Italy

* Correspondence: ine.vanderfels@wur.nl

**Abstract:** Mycotoxin contamination in small grain cereals is a worldwide challenge for animal and human health. Currently, operational models are available for forecasting the presence of deoxynivalenol in wheat, either empirical or mechanistic. This study compares different modelling approaches on predicting deoxynivalenol contamination levels in winter wheats, aimed to limit mycotoxin contamination in the Netherlands. These three approaches are empirical modelling (based on the model developed by van der Fels-Klerx in 2010), Bayesian network modelling and mechanistic modelling (based on the decision support system developed by Vittorio et al. in 2007). All three approaches use weather and agronomic data collected from the Dutch weather stations and field surveys between 2001 and 2016. The modelling methods and validation results will be presented during the conference.

These models can be used by farmers to optimize the agricultural management practices, such as fungicide application and harvest time. They can also be used by wheat collectors and by food safety authorities to locate high risk areas for a more targeted and cost-efficient sampling. All three models will be linked in to the dynamic component of MyToolBox for interactive use by farmers and advisors.

### 7.55. Associations between Fusarium Mycotoxin Accumulation in Oats and Weather Conditions

Hjelkrem A-G. R. ^1,^*, Aamot H.U. ^1^, Brodal G. ^1^, Strand E. ^1,2^, Torp T. ^1^, Dill-Macky R. ^3^, Edwards S. ^4^, Nordskog B. ^1^ and Hofgaard I.S. ^1^

^1^ Norwegian Institute of Bioeconomy Research (NIBIO), Ås, Norway

^2^ Norwegian Agricultural Extension Service, Ås, Norway

^3^ University of Minesota, St Paul, USA

^4^ Harper Adams University, Shropshire, UK

* Correspondence: anne-grete.hjelkrem@nibio.no

**Abstract:** High concentrations of the mycotoxins deoxynivalenol (DON), produced by *Fusarium graminearum*, and HT-2 and T-2 produced by *Fusarium langsethiae,* have occurred frequently in Norwegian oats in recent years. We have identified the main weather factors influencing mycotoxin accumulation in Norwegian oats. First, a mathematical model was developed and used to estimate developmental stages in oats (tillering, flowering etc.) based upon weather data. Weather summarisations within specific developmental stages were then calculated for a number of oat fields. A Spearman rank correlation factor was then calculated between mycotoxin-contamination in oats at harvest and the weather summarisations within each developmental stage. The most important weather variables identified were included in the development of empirical models with the aim to predict the risk of mycotoxin accumulation in harvested oats. The models should be useful to authorities and industry representatives to identify grain lots with potential food safety problems.

### 7.56. Don-Maize: A Prototype Mechanistic Model to Predict Deoxynivalenol and Zearalenone Contamination in Maize

Leggieri M.C. and Battilani P. *

Department of Sustainable Crop Production, Università Cattolica del Sacro Cuore, Piacenza, Italy

* Correspondence: paolo.battilani@unicatt.it

**Abstract:** Maize is mentioned as a key host crop for different mycotoxin producing fungi. Co-occurence of fungi can frequently be observed even if, in a certain year and area, a species of main concern can commonly be pointed out. In south Europe, mycotoxin contamination in maize is mainly due to fumonisins and, since 2003, aflatoxins, produced by *Fusarium verticillioides* and *Aspergillus flavus,* respectively. A severe deoxynivalenol (DON) and zearalenone (ZEN) contamination, due to *F. graminearum* infection, was instead reported in 2014.

Due to the climate change, with extreme events following each other during the crop growing season, the mycotoxin of main concern, or the co-occurrence of different toxins, became more frequent events. Predictive models, included among the supporting tools in managing mycotoxins in agri-food chains, acquired more relevance in these uncertain scenarios.

Several efforts have been devoted in the last 10 years to develop and validate predictive models for mycotoxin producing fungi. AFLA-maize and FER-maize, two mechanistic models able to predict the risk of aflatoxin and fumonisin contamination in maize, respectively, are crucial output. They gave good predictions in different maize growing areas worldwide. Poor attention was instead devoted to DON/ZEN in maize; therefore, the aim of this work was to develop a prototype mechanistic model to predict the behaviour of *F. graminearum* in maize and the risk of DON/ZEN contamination.

Systematic literature review regarding *F. graminearum* infection cycle in maize was managed. The relational diagram was developed following the principles of “system analysis”. State variables, rates and driving variables were determined and linked in a coherent framework. Quantitative data for each steps of the cycle were collected, stored in a data base, and mathematical functions developed.

The model predicts *F. graminearum* fungal development and DON and ZEN production (output) on a daily base using weather data (hourly air temperature, relative humidity and rain) as input. After a proper validation, the model will support stakeholders in: (a) describing the dynamic of DON/ZEN risk during the maize-growing season and at harvest, using real time data, (b) drawing different scenarios based on past and future (climate change) weather data.

This work was managed in the project “Integrated and innovative key actions for mycotoxin management in the food and feed chain—MycoKey”, GA n.678781, supported by European Commission in the H2020 framework program (www.mycokey.eu).

### 7.57. Improved Modelling of Fusarium to Aid Mycotoxin Prediction in UK Wheat

Edwards S.G. ^1,^* and Jennings P. ^2^

^1^ Harper Adams University, Newport, Shropshire TF10 8NB, UK

^2^ Fera Science Ltd., Sand Hutton, York YO41 1LZ, UK

* Correspondence: sedwards@harper-adams.ac.uk

**Abstract:** The aim of this study was to improve the prediction of fusarium mycotoxins in UK wheat. Collated data including previous datasets (2006–2011) were used to develop models to predict mycotoxin risk. Models were validated using separate dataset (2012–2013). A new agronomic factor determined was harvest date with a one month delay in harvest resulting in a large increase in risk for both DON and ZON, as was experienced with the delayed harvest of 2008. Prediction of mycotoxin late season, based on a combination of national fusarium head blight pathogen incidence (as recorded at growth stage 73) and agronomy was a better predictor of risk compared to weather variables and proved reasonable at the national and field scale. At field scale, the DON model was a better predictor of risk than the ZON model and could be used to predict ZON as well as DON risk. As false negatives have a greater consequence for the industry (consignments of wheat exceeding legal limits entering the food chain), then the probability of exceeding the legal limit was calculated and this was used to determine a lower risk threshold for predicting false negatives. If a threshold of 5% is set, then a sample is deemed to be below the legal limit if the probability that the actual concentration will exceed 1250 ppb DON is below 5%. With a 5% probability that a sample exceeded the DON limit, no samples above the legal limit were predicted not to exceed the DON limit (zero false negatives) and only 0.4% of ZON samples exceeded the ZON limit of 100 ppb but were predicted not to (false negatives). This study shows the benefit of using fusarium head blight incidence data as a measure of seasonal risk within a fusarium mycotoxin prediction model. Late season field scale risk prediction could be used by the cereal chain to determine which consignments of wheat require testing for fusarium mycotoxins prior to delivery into the human food chain.

### 7.58. High Throughput Ecophysiology and Modelling Approaches for Aspergillus Flavus Using Turbidimetric Readings

Medina A. ^1,^*, Aldars-Garcia L. ^2^, Bulla G. ^1^, Marin S. ^2^ and Magan N. ^1^

^1^ Applied Mycology Group, Environment and AgriFood Theme, Vincent Building, Cranfield University, Cranfield, Bedford MK43 0AL, UK

^2^ Applied Mycology Unit, Food Technology Department, University of Lleida, Avda. Rovira Roure, 191. 25198 Lleida, Spain

* Correspondence: a.medinavaya@cranfield.ac.uk

**Abstract:** The ability of environmental factors to modify the growth and toxin production by mycotoxigenic species is now widely recognised. Today, experiments are designed to study the effect of different antifungal compounds, culture media, modified water availability and different temperatures. If all the combinations should be tested, the experiments require long periods of time and imply large numbers of repetitions and a high number of Petri plates that should be measured daily to record colony diameters. Automated turbidimetric methods can monitor the growth of pure or mixed cultures and check the effect of single or multiple parameters in the same run. The main advantages are (i) the automatization of the system, (ii) the reduction of culture volumes, (iii) the continuous monitoring of fungal growth and (iv) accurate control of temperature.

In this study, we will present our latest data obtained with an automated method using the Bioscreen C that allows us to monitor the effect of different interacting environmental conditions (temperature, water availability, antifungal compounds at different concentration). For these studies, we have used *Aspergillus flavus* and aflatoxin production because of its toxicity and importance in developing minimisation strategies. Results are presented on the effect of environmental conditions on the growth and Aflatoxin B_1_ production in relation to the efficacy of (a) different antifungal compounds with multiple concentrations, (b) the effect on different carbon source utilisation patterns by using the Temporal Carbon Utilization Sequence (TCUS) and (c) intraspecific differences between growth and toxin production by high and low producing *A. flavus* strains. This will show that high throughput turbidimetric techniques provide advantages in evaluating the ecophysiology and molecular ecology of mycotoxigenic fungal species and impacts on growth and mycotoxin modulation. This approach provides significant benefits over traditional approaches for identifying the efficacy of mycotoxin minimisation strategies.

### 7.59. Multi Mycotoxin Contamination in Fermented Locust Beans (Parkia Biglobosa) and the Perception of Mycotoxin Contamination in Nigeria and South African Markets

Adekoya I. ^1,2,^*, Njobeh P. ^1^, Adaku C. ^1^, Obadina A. ^3^, Okoth S. ^4^, De Boevre M. ^2^ and De Saeger S. ^2^

^1^ Department of Biotechnology and Food Technology, University of Johannesburg, Johannesburg, South Africa

^2^ Laboratory of Food Analysis, Department of Bioanalysis, Ghent University, Ghent, Belgium

^3^ Department of Food Science and Technology, Federal University of Agriculture, Abeokuta, Nigeria

^4^ Department of Botany, School of Biological Sciences, University of Nairobi, Kenya

* Correspondence: olotu.ifeoluwa@gmail.com

**Abstract:** Fermented foods represent a significant part of the diet of people around the world with its provision of 20–40% of the food supply. However, the incessant proliferation of food commodities by fungi and their toxic metabolites (mycotoxins) coupled with their critical effect on food safety and health has prompted the need to assess their occurrence and evaluate the level of awareness of people on fungi and mycotoxins. Also, considering the ability of several fungi to produce more than one mycotoxin, multiple contaminations can be expected. To date, there is little or no information on multiple mycotoxin occurrences in fermented locust beans. In this study, the knowledge of fermented food vendors and processors on fungi and mycotoxin and the occurrence of mycotoxins in fermented locust beans consumed in Nigeria and South Africa were assessed. The baseline assessment survey showed that 66% of the vendors and processors (*n* = 106) had knowledge about fungi, and 46% stated that they could identify foods/crops contaminated with fungi while 24% attributed fungi contamination to poor storage conditions. Only 2% of the respondents were aware of mycotoxins production by fungi based on their level of education. Fermented locust beans (*n* = 126) were randomly collected from retail markets in 2015 from Southwest, Nigeria and Gauteng, South Africa. Previous studies by the authors have shown the presence of toxigenic fungi in the samples. Analytical methods using LC-MS/MS have been developed and validated for the detection and quantification of 26 mycotoxins. The expected results will give a comprehensive overview and insight into the safety of fermented locust beans, contribute to the formulation of food safety action plans and serve as a basis for mycotoxin awareness creation in both countries.

### 7.60. Risk Assessment of Mycotoxins Associated with Consumption of Stored Maize Grains by Infants and Children in Nigeria

Adetunji M.C. ^1,^*, Atanda O.O. ^1^ and Ezekiel C.N. ^2^

^1^ Department of Biological Sciences, McPherson University, Seriki Sotayo, Ogun State, Nigeria

^2^ Department of Microbiology, Babcock University, Ilishan Remo, Ogun State, Nigeria

* Correspondence: olusegunatanda@yahoo.co.uk

**Abstract:** Maize is a staple cereal that is used as complimentary or weaning food particularly among infants and young children (IYC) in Nigeria. In this study we determined the Probable Daily Intake (PDI) and characterized the risk assessment of infants and young children to some naturally occurring mycotoxins in stored maize grains from five agro-ecological zones of Nigeria. The mean national PDI of aflatoxin was estimated to be 1909.7 and 763.63 ng/kgbw/day respectively for the infants and children while that of fumonisins, ochratoxins, deoxynivalenol and zearalenone was 12,444.62, 1933.14, 637.63 for infants and 8296.40, 140.20, 255.04 for children. The highest risk of exposure to aflatoxins (3402, 1,360.82 ng/kgbw/day) and ochratoxins (732.11, 292.84 ng/kgbw/day) was found in Infants and children in the Derived Savannah (DS) zone while infants in the Northern Guinea Savannah (NGS) zone recorded the highest risk of exposure to fumonisins (16,983.15 ng/kgbw/day) and deoxynivalenol (629.40 ng/kgbw/day). Furthermore, the mean national margin of exposure was calculated to be 0.12 and 0.3 for infants and children thus indicating a public health concern. The mean national population at risk of liver cancer was estimated to be 152.69 and 61.07 cancer/year/100,000 population of infants and children respectively. The mean national TDI for fumonisins, ochratoxins and infants exposed to zearelenone contamination was more than 100% and was about 850-fold for fumonisins in the NGS zone. Infants and children in Nigeria are therefore vulnerable to the effect of mycotoxin contamination hence intervention strategies are needed across the zones.

### 7.61. Awareness and Perception about the Occurrence, Causes and Consequences of Aflatoxin Contamination in Burundi and Eastern Democratic Republic of Congo

Wiredu A.N. ^1,^*, Udomkun P. ^2^, Nielson F. ^2^, Vanlauwe B. ^3^ and Bandyopadhyay R. ^4^

^1^ International Institute of Tropical Agriculture (IITA), Nampula, Mozambique

^2^ International Institute of Tropical Agriculture (IITA), Bukavu, the Democratic Republic of Congo

^3^ International Institute of Tropical Agriculture (IITA), Nairobi, Kenya

^4^ International Institute of Tropical Agriculture (IITA), Ibadan, Nigeria

* Correspondence: n.wiredu@cgiar.org

**Abstract:** Despite efforts to reduce aflatoxin contamination and associated mycotoxin poisoning, the phenomenon continues to pose public health threat in food and feed commodity chains. To support effective development and deployment of technologies and strategies, this study examines awareness and perception of the occurrence, causes, and consequences of aflatoxin contamination among a cross section of 310 farmers in Burundi (160) and eastern Democratic Republic of Congo (DRC) (150). The results show about 53% aware rate within the sampled. Farmer-to-farmer information flow serves as important source of information about aflatoxins. While farmers in Burundi access their information from government extension services, those in eastern DRC obtain information through their own observation. Kendal’s concordance rank correlation analysis shown agreement in the perception of the farmers across the two locations. The results showed that the use of contaminated seeds potentially increases the prevalence of aflatoxin contamination. Severity on the other hand is associated with delayed harvesting and the extent of spread of the contamination. Biological factors such as pest and disease attacks also increases the prevalence and severity of aflatoxin contamination. Drought stress and high temperatures followed by high humidity towards harvesting periods increased the prevalence, severity and spread of aflatoxin contamination. The farmers also identified changes in taste, smell, and colour of agricultural produce as signs of contamination. They associated contamination with reported cases of liver infections and low resistance to diseases. This is further compounded by their inability to sell crop at true market values. The results suggest the need to increase awareness among farmers about aflatoxin contamination and associated effects. This require partnerships with actors in the food value chains. There is also the need to examine the extent to which technologies are suitable and affordable for farmers.

### 7.62. Current Trends in Sample Size in Mycotoxin Analysis in Grains: Are We Measuring Accurately?

Matumba L. ^1,^*, Whitaker T. ^2^, Slate A. ^2^ and De Saeger S. ^3^

^1^ Food Technology and Nutrition Group, Lilongwe University of Agriculture and Natural Resources (LUANAR),NRC Campus, P.O. Box 143, Lilongwe, Malawi

^2^ Biological and Agricultural Engineering Department, North Carolina State University, North Carolina, USA

^3^ Department of Pharmaceutical Bioanalysis, Campus Heymans, Faculty of Pharmaceutical Science, Ottergemsesteenweg 460, B-9000 Ghent, Belgium

* Correspondence: alimbikani@gmail.com

**Abstract:** Due to heterogeneous distribution of mycotoxins in food and feed, reliability of analytical measurement is greatly affected by sampling. High concentrations of mycotoxins have been found in individual kernels of corn, peanuts, and cottonseed and tree nuts thus heterogeneity of mycotoxins vary strongly amount of kernels per gram. It is well established that variance of estimated concentrations is inversely proportional to sample size. However, recent years have witnessed a revolution in sample size reduction with most laboratory increasingly grinding less than 1 kg of corn or peanuts, and extracting a sample than 10 g of dry grind. The present paper critically reviews the current trends in the sample sizes, sample grinders used, extraction vessels, et cetera over a period of two decades using selected mycotoxin journals and further compares these parameters against national and regional guidelines. The paper discusses the associated variance and concludes on the accuracy of the associated mycotoxin analytical results.

## 8. Poster Presentations

### 8.1. Novel Biocontrol Agents Against Fusarium Graminearum and Its Mycotoxins in Maize

Abdallah M.F. ^1,2,^*, De Boevre M. ^1^, De Saeger S. ^1^, Haesaert G. ^2^ and Audenaert K. ^2^

^1^ Department of Bioanalysis, Faculty of Pharmaceutical Sciences, Ghent University, Belgium

^2^ Department of Applied Biosciences, Faculty Bioscience Engineering, Ghent University, Belgium

* Correspondence: mohamed.fathi@ugent.be

**Abstract:** Fusarium Head Blight (FHB) is a devastating fungal disease which affects small grain cereals such as wheat and maize. Although FHB is caused by a species complex, *Fusarium graminearum* (Fg) is the most important member. Beside the economic losses due to the decrease in yield, the fungus has an impact on the quality due to the production of mycotoxins. Additionally, these mycotoxins have a serious impact on human and animal health upon consumption of the contaminated cereals. Driven by the awareness that reduced tillage systems result in soil structure improvement, conservation tillage practices are often implemented leaving more stubble/straw residues on the field. This organic material can serve as the primary inoculum of Fg. Over the last decade, different strategies for FHB management have been proposed. Biological control using beneficial or non-pathogenic bacteria and fungi is encouraged as it is a safe and sustainable long-term solution in comparison with chemical control. Although crop residues serve as primary inoculum of Fg, we hypothesize that these crop residues also harbor valuable antagonistic fungi which might be used as biocontrol agents. In the current project, several novel fungal endophytes and antagonists have been isolated from European and African crop residues. They were tested for their ability to control the growth of Fg and the production of its mycotoxins in vivo and in vitro.

New isolates of *Sordaria* spp, *Clonostachys* spp, and *Epicoccum* spp., were tested for their effects against Fg. In vitro plating assays and maize pot experiments have been performed for each isolated species to assess their biocontrol capacity against Fg. The obtained results indicate that the selected biocontrol agents have a promising effect on Fg growth. Furthermore, measuring the mycotoxin levels (deoxynivalenol, 15-acetyldeoxynivalenol, 3-acetyldeoxynivalenol and zearalenone) through a validated multi-mycotoxins LC-MS/MS method, shows that the selected biocontrol agents have also an inhibitory effect on mycotoxins production. Using a non-targeted approach, with Q-TOF LC/MS we investigated whether these biocontrol agents have a detoxification effect and/or produce inhibitory volatiles or other substances that may affect on the fungus metabolism. The project results will contribute to a great extend to the reduction of *fusarium* mycotoxins level in grain cereals especially wheat and maize.

The project is a part of the **MYCOKEY** project that aims at ‘*Integrated and innovative key actions for mycotoxin management in the food and feed chain’*. The project is funded by Horizon 2020.

### 8.2. In Vivo Study of the Modulating Effect of Citrulus Coloquintus Oil in Wistar Rats Poisoned by Ochratoxin A

Abdel-ilah A. ^1,^*, Meriem C.H. ^2^, Houcine B. ^3^, Imane Z. ^2^ and Daoudi C.S. ^2^

^1^ Salhi Ahmed Center University BP: 66 Naama 45000, Algeria

^2^ Research Laboratory of Naturel Products (LAPRONAT). Abou Bekr Belkaid University Tlemcen, Algeria

^3^ LASNABIO Research Laboratory. Abou Bekr Belkaid University Tlemcen, Algeria

* Correspondence: abdelillahamrouche@yahoo.fr

**Abstract:** The aim of this study was to evaluate in vivo the modulating impact of *Citrullus colocynthis* seed oil in the growing wistar rat poisoned by 250 micrograms of OTA.

Starting from the first observations, we noted that the weight change of the intoxicated rats marked a decrease compared to the control rats (8.58%). The kinetics of organ weight change for different groups of rats in the presence of the toxin show no significant differences for the kidney and heart. Moreover, the weight of the liver in the rats receiving the colocynth oil decreases significantly. An increase in liver and brain weight is noted in the presence of the toxin. However, the weight of the pancreas decreased significantly in rats in the presence of OTA having received colocynth oil. The lipid balance shows a decrease in the mean values of the triglyceridemia contrasting with a rise in cholesterolemia compared to the rats of the control group. In addition, an improvement in lipid balance was observed after supplementation of diets poisoned by 4% colocynth oil. The hepatic enzyme balance revealed a decrease in the level of ASAT in OTA poisoned rats receiving *Citrullus colocynthis* oil. The ASAT/ALAT ratio is not modified by adding the oil relative to the rats of the control batch but this ratio reaches a maximum in the intoxicated rats, note that this ratio tends towards a correction by adjuvant colocynth oil.

The colocynth oil has stabilizing or corrective effects on body weight, lipid and protein metabolism as well as expression of transaminases. These actions are signs of a possible modulation of the impact of mycotoxins.

### 8.3. Development of A Multiplex Dipstick Immunoassay for the Rapid Quantitative Determination of Fusarium Toxins in Cereals

Aggoun N. ^1,^*, Xhardé S. ^1^, Granier B. ^1^, Pascale M. ^2^, De Saeger S. ^3^, Logrieco A.F. ^2^ and Lattanzio V.M.T. ^2^

^1^ Unisensor, Diagnostic Engineering, Liège, Belgium

^2^ Institute of Sciences of Food Production, National Research Council of Italy, Bari, Italy

^3^ Ghent University, Faculty of Pharmaceutical Sciences, Department of Bioanalysis, Ghent, Belgium

* Correspondence: noreddine.aggoun@unisensor.be

**Abstract:** Development, validation and one-site testing of immunoassays for rapid mycotoxin detection is one of the priority tasks of the MycoKey project (http://www.mycokey.eu/), an EU multidisciplinary project, aimed to develop smart solutions to reduce the major occurring mycotoxins in economically important food and feed chains. In this framework, a multiplex dipstick immunoassay based method for the simultaneous quantitative determination of major *Fusarium* toxins, namely deoxynivalenol (DON), zearalenone (ZEA), and fumonisins (FUM) in cereals (wheat, barley and maize) is under development. The dipstick format is based on an indirect competitive approach. The DON/ZEA/FUM prototype is based on a nitrocellulose lateral flow device using a reader to enable quantitative determination of mycotoxin contamination in cereal extracts. Three test lines (mycotoxin–BSA conjugates) and one control line are located on the strip membrane, whereas labeled antibodies were freeze-dried within a microwell. The prototype strip test development included the following steps: definition of coating process and detection reagent formulation parameters; definition of assay parameters like incubation time, migration time, volume sampling to reach minimum performance requested in term of LoD (limit of detection), LoQ (limit of quantification); development of a sample preparation protocol allowing satisfactory mycotoxin recoveries. The total time of analysis is 25 min including pre analytical treatment.

Preliminary results showed the DON/ZEA/FUM prototype to be compatible with the minimum acceptable performances, and to be suitable for its application to the analysis of real samples containing the target mycotoxins at levels close to EU regulatory levels.

The present work has received funding by the European Union’s Horizon2020 Research and innovation programme under Grant Agreement No. 678781 (MycoKey).

### 8.4. Reduction of Deoxynivalenol and Zearalenone Contamination in Wheat Bran by Ozone Treatment

Alexandre A.P.S., Santos A.S., Costa N.S., Calori-Domingues M.A. * and Augusto P.E.D.

Department of Agri-food Industry, Food and Nutrition (LAN), Luiz de Queiroz College of Agriculture (ESALQ), University of São Paulo (USP), Piracicaba/SP, Brazil

* Correspondence: macdomin@usp.br

**Abstract:** One successfully alternative to reduce an unavoidable mycotoxin contamination in agricultural products is ozone gas (O_3_). Ozone has high oxidizing potential and can be generated electrically at the time of use. It is considered a green chemical process and is recognized as GRAS—*Generally Recognized As Safe*. The objective of this study was to evaluate the effectiveness of ozone treatment on the reduction of DON and ZEN in wheat bran under different exposure time. Naturally contaminated wheat bran samples with the co-occurrence of DON (2164 ± 296 μg kg^−1^)and ZEN (1093 ± 96 μg kg^−1^), exceeding the maximum limits (ML) in Brazilian and European Commission regulations, were evaluated. Ozone treatment was performed at a flow rate of 0.5 L min^−1^ and O_3_ concentration in the gas stream was 62 mg L^−1^. The initial sample moisture content was about 14%. Wheat bran samples (initial and ozone treated) were analyzed using methanol:water (80:20) as extraction solvent for ZEN and distilled H_2_O for DON. Immunoaffinity columns were used as cleaned up step and the mycotoxin detection/quantification was by HPLC/diode array detector for DON and HPLC/fluorescence for ZEN. DON and ZEN mean concentration in ozone treated wheat bran was significantly lower (*p* < 0.05) than the initial mycotoxin concentrations. For both mycotoxins no significant differences among the studied times was observed. The reduction of ZEN concentration (mean, 60%) was approximately twice the reduction of DON (mean, 32%). The higher ZEN reduction in wheat bran when compared to DON could be attributed to the different molecular structures of the mycotoxins. Therefore, further studies are needed to better understand the process.

Financial Support: São Paulo Research Foundation—FAPESP (grant # 2016/10732-7).

### 8.5. Impact of Environmental Conditions and Pre-Harvest Management Practices in Maize on the Occurrence of Fusarium Mycotoxins

Alfonso P. ^1^, Bervis N. ^1^, Lorán S. ^1^, Anadón R. ^2^, Ciércoles R. ^2^, Ariño A. ^1^ and Herrera M. ^1,^*

^1^ Instituto Agroalimentario de Aragón IA2 (Universidad de Zaragoza—CITA), Veterinary Faculty, 50013 Zaragoza, Spain

^2^ Scientific Technological Park Foundation Aula Dei (PCTAD), 50059 Zaragoza, Spain

* Correspondence: herremar@unizar.es

**Abstract:** The Food and Agriculture Organization of the United Nations (FAO) has estimated that approximately 25% of the crops worldwide are contaminated with mycotoxins. The *Fusarium* fungi are important in the cereal food chain because can reduce crop yields and contaminate maize grains with mycotoxins. A tool for the prevention and reduction of *Fusarium* mycotoxins in maize crops is the application of Good Agricultural Practices (GAP) during production and harvest, taking into account the local crops, climate, and agronomic practices.

The objective of the present work was to identify the major risk factors for mycotoxin contamination in locally grown maize as a basis to develop future strategies to minimize the risks associated with *Fusarium* toxins. For the 2016 crop season, 9 maize growing areas located in Aragón (NE Spain) were selected. Detailed information on agricultural practices were obtained from farmers, including date of harvest, previous crop, pests, crop residues management, irrigation and water stress between flowering and harvest. Maize samples were analyzed for deoxynivalenol (DON), fumonisins (FUM), zearalenone (ZEA) and T-2/HT-2 toxins by immunoassays based on lateral flow technique and confirmation by liquid chromatography with appropriate detectors.

DON and ZEA were detected in 7 and 8 out of 9 locations ranging from 630–4000 μg/kg and 70–320 μg/kg, respectively. Fumonisins occurred in 5 out of 9 locations at levels between 270 and 4000 μg/kg, while none of the areas showed T-2/HT-2 contamination. The highest levels of DON and ZEA that occurred in maize grown in warm areas may be related with high temperatures (>37 °C) during flowering coupled with sprinkler irrigation, as well as second-crop maize under direct sowing. Fumonisin occurrence was relevant in maize areas highly affected by corn-borers. These preliminary results show that local environmental conditions and agricultural practices can play a relevant role in mycotoxin contamination in maize. The study will be extended during at least two more crop seasons in order to have all the risk factors represented.

The present project will assist producers regarding the environmental and agronomic factors that promote infection, growth and toxin production in local cereal crops at the farm level, as well as those involved in the postharvest handling and processing of grains intended for food and feed.

Acknowledgments: Project RTC-2016-4833-2 (MINECO) and Grupo Consolidado A01 (Gobierno de Aragón-FEDER). N. Bervis thanks the grant FPU13/04238.

### 8.6. Water Relations of Aspergillus Flavus Strains Isolated from Chillies: Effect on Lag Phases, Growth and Aflatoxin B_1_ Production

Aljaza D. *, Medina A. and Magan N.

Applied Mycology Group/ Environment and AgriFood Theme/Cranfield University, Bedford, MK43 0AL, UK

* Correspondence: d.a.aljaza@cranfield.ac.uk

**Abstract:** Contamination of spices with aflatoxin B_1_ (AFB_1_) has resulted in EU legislative limits for this mycotoxin. Chillies are particularly prone to contamination with AFB_1_. This study has examined three *Aspergillus flavus* strains isolated from chillies in Iraq. The effect of interactions between temperature (15–37 °C) and water activity (0.995–0.90 a_w_) on lag phases prior to growth, mycelial growth and aflatoxin B_1_ production was examined on a 10% chilli-based medium. The lag phases prior to growth were delayed markedly by lower temperatures (15–20 °C) and a_w_ (0.928–0.90 a_w_). Optimum growth of *A.flavus* strains was at 37 °C and 0.982 a_w_ for the strains examined. The optimum temperature x a_w_ conditions for AFB_1_ production were at 30 °C and 0.982 a_w_ by two strains and 25 °C in 0.982 a_w_ by the third strain. AFB1 was produced in lower amounts at 35 and 37 °C and 0.995 a_w_. However, no production of AFB_1_ occurred at 15 and 20 °C at 0.90 and at 0.928 a_w_ respectively. Statistical analysis showed that temperature and a_w_ had a significant effect on lag time prior to growth (*p* < 0.05) for the strains. There was also a significant effect of temperature x a_w_ on relative growth and AFB_1_ production by the three strains (*p* < 0.05). These results are discussed in the context of minimisation strategies using different food grade preservatives.

### 8.7. Inhibition of Formation of Perithecia of Fusarium Graminearum by Antagonistic Isolates of Trichoderma spp.

Altomare C. *, Branà M. T., Gallo A., Cozzi G. and Logrieco A. F.

Institute of Sciences of Food Production, National Research Council, Italy

* Correspondence: claudio.altomare@ispa.cnr.it

**Abstract:** Fusarium head blight (FHB) is a world-wide occurring disease of wheat and other grain crops that causes yearly considerable losses in terms of yield and quality of grains. The severity of the disease is aggravated by intensive crop management and some cultural practices, such as monocropping and conservation tillage. Moreover a further increase of FHB is expected in temperate areas as a result of the global climate change. The infection of wheat heads is primarily caused by spores of *Fusarium graminearum* (teleomorph: *Gibberella zeae*) that infect the spikes at flowering and impair formation of the embryos and accumulation of starch in the endosperm of the developing kernels. Besides being small, shrunk and whitened, the infected kernels may also contain mycotoxins produced by *F. graminearum* (mainly deoxynivalenol and zearalenone), which enter the food and feed chains and pose safety concerns for human and animal health. The main source of inoculum for flowers infection are the ascospores, which are formed inside perithecia, the flask-shaped fruiting bodies of the fungus that are developed by the overwintering mycelium on the infected plant debris of previous susceptible crops. Since chemical control is difficult and raises environmental and safety concerns, prevention of perithecia formation and ascospore release appears a feasible means for FHB control. We investigated the capability of seven *Trichoderma* spp. strains to inhibit perithecia formation in dual culture tests. One isolate of *F. graminearum* was challenged with the antagonistic *Trichoderma* spp. strains on carrot-agar medium; after 7 days of co-culture the mycelium was peeled off the plates and production of perithecia was induced by fertilization of cultures. After 7 more days of incubation at 25 °C, the number of perithecia formed was assessed in the plate sectors that were pre-colonized by either *Trichoderma* or *Fusarium*. In the *Trichoderma* pre-colonized sectors, perithecia formation was inhibited by 80 to 100%. In the *Fusarium* pre-colonized sectors, perithecia formation was totally inhibited by 3 out of 7 tested *Trichoderma* isolates, while the other 4 isolates showed not significant perithecia inhibition. Further investigations on the mechanism of perithecia inhibition showed that the *Trichoderma* strains released unidentified metabolites that were able to reduce the number of perithecia formed. The reduction of number of perithecia formed by *F. graminearum* colonies exposed to *Trichoderma* cell-free metabolites ranged from 27% to 91%, depending on the *Trichoderma* strain. To explore the effect of *Trichoderma* metabolites on the regulatory mechanisms of perithecia formation, we carried out a preliminary study of genes involved in the perithecia developmental process. This study allowed to identify a first group of genes associated with different stages of the perithecia formation, whose expression rate in response to *Trichoderma* metabolites is under investigation.

This work was supported by H2020- MycoKey-(E.U.3.2-678781).

### 8.8. Challenges of Risk Assessment of Mycotoxins in Food and Feed Supply Chains-Apple Juice Processing and Maize Supply Chain as Examples

Aroud H.I. *, Zupaniec M. *, Kemmlein S., Schafft H. and Lahrssen-Wiederholt M.

Department Safety in the Food Chain, Federal Institute for Risk Assessment, Berlin, Germany

* Corresponding authors: Husam.Ibrahem-Aroud@bfr.bund.de (Project A); Milena.Zupaniec@bfr.bund.de (Project B)

**Abstract:** Occurrence of mycotoxins along the supply chain of food- and feedstuff is one of the major concerns of food safety especially with the increasing globalization and complexity of supply chains. A key challenge of risk assessment is the availability of sufficient data and information about the occurrence and the fate of specific mycotoxins along the supply chain. Two pilot projects, comprising analytical strategies of juice processing as well as approaches for supply chain analysis including logistics are being developed at the Federal Institute for Risk Assessment ( BfR), Germany, that aim to increase supply chain integrity.

Project A: “Occurrence and fate of mycotoxins along apple juice processing supply chain”:

A fast, reliable liquid chromatography/tandem mass spectrometric multi-mycotoxin method has been developed and being validated for the determination of multiple mycotoxins in clear and cloudy apple juices. The application of the validated method will help to identify the influencing factors as well as the relevant steps of the occurrence or/ and the reduction of mycotoxins along the apple juice processing supply chain. Generating data by testing apple juice samples in turn will provide information to assess the risk of the occurrence of these undesired substances in the final products.

Project B: “Effects of global supply chains on the contamination of agri-food”:

An approach for a risk-based supply chain analysis with the focus on logistics is being developed using the demonstrator maize and its contamination with mycotoxins. The approach combines a supply chain analysis comprising visualization/characterization and scenario-building with an hazard analysis consisting of vulnerability analysis (input of contaminants/ mycotoxins in the supply chain) and the analysis of critical points in terms of the occurrence of mycotoxins in each step of supply chain with the emphasis on the logistic chain (transport, handling and storage) as crucial part of the global supply chain of agri-food.

### 8.9. Impact of Changes in Temperature, Water Stress and Carbon Dioxide Concentrations on Growth and Ochratoxin a Production by Aspergillus Niger in Green Coffee Beans

Aukkasarakul S. ^1,^* and Somboonkaew N. ^2^

^1^ Mycology Laboratory, Postharvest and Processing Research and Development Division, Department of Agriculture, Bangkok, Thailand

^2^ Postharvest Technology of Horticultural Crops, Postharvest and Processing Research and Development Division, Department of Agriculture, Bangkok, Thailand

* Correspondence: suppara.au@gmail.com

**Abstract:** Climate change can affect the biodiversity of plant ecosystems including contaminant microorganism. *Aspergillus niger* is responsible for contamination of many stored food commodities resulting in contamination with ochratoxin A (OTA). OTA is classified as a human carcinogen and harmful to consumers and animals. The objectives of this study were to examine the impact of changes in temperature, water stress and carbon dioxide (CO_2_) levels on (a) growth and (b) OTA production by strains of *A. niger* in vitro and on green coffee beans. This type of knowledge is beneficial for providing ecological data to predict growth and OTA contamination of food commodities under climate change scenarios. Growth was measured on yeast extract sucrose agar (YES agar) for 10 days and for OTA production for 14 days. The range of conditions for *A. niger* growth were 17–38 °C, water activity (a_w_) 0.87–0.99 and CO_2_ 300–1000 ppm. Optimum temperature was 25 °C and CO_2_ 600 ppm and at 0.93, 0.95, 0.99 a_w_. No growth was observed in vitro at 0.75–0.85 a_w_. In addition, *A. niger* was inoculated in stored green coffee beans. At 35 °C + CO_2_ 300–1000 ppm with 80% RH, *A. niger* produced high levels of OTA (between 4.9–2.0 μg/kg). In contrast, at 15 °C + 300, 600 and 1,000 ppm CO_2_ with 60% RH, *A. niger* produced low levels of OTA in the range 0.8 to 1.8 ppb. These results are discussed in the context of the impact that climate change factors could have on OTA contamination of such economically important commodities.

### 8.10. Efficacy of an Anti-Mycotoxin Additive to Prevent Deleterious Effects of Zearalenone on Sexually Immature Gilts

Avantaggiato G. ^1,^*, Lizardo R. ^2^, Greco D. ^1^, D’Ascanio V. ^1^, Santovito E. ^1^, Marquis V. ^3^ and Auclair E. ^3^

^1^ Istituto di Scienze delle Produzioni Alimentari (ISPA-CNR), Bari, Italy

^2^ Nutrició Monogàstrics, IRTA, Constanti, Spain

^3^ Phileo Lesaffre Animal Care, Marcq-en-Baroeul, France

* Correspondence: giuseppina.avantaggiato@ispa.cnr.it

**Abstract:** Products containing yeast cell wall (YCW) can adsorb zearalenone (ZEA), and may play a beneficial role in reducing the overall impacts of a ZEA challenge on pigs. To the best of our knowledge, there is a lack of scientific data on the ability of YCW in reducing ZEA absorption in pigs proved by the biomarker approach. The aim was to examine the ability of a YCW supplemented diet in reducing the toxic effects of a chronic exposure to ZEA on growth performance, vulva size, serum hormones and organ weights of sexually immature gilts. The study assessed the effect of YCW on the excretion/accumulation of ZEA and its metabolites in biological samples. Twenty four sexually immature gilts (10.5 kg, initial body weight) were fed three diets for 42 days: Treatment 1 (T1) (88.9 μg Kg^−1^ ZEA) was used as control; Treatment 2 (T2) received naturally contaminated diet (465.6 μg Kg^−1^ ZEA); Treatment 3 (T3) received naturally contaminated diet (418.7 μg Kg^−1^ ZEA) supplemented with 2 Kg T^−1^ of YCW. During the 42 days experiment, gilts were fed with *ad libiitum* access to water and feed. At 1, 13, 27 and 42 days of treatment, gilts were individually placed in metabolic cages to collect 24 h-urine, 24 h-feces and blood. Gilts exposed for 42 days to ZEA contaminated diet (T2) showed a reduction in the average daily gain and final body weight, and an increase in the vulva size and genital organs weight as compared with the control group (T1) (*p* < 0.05). These detrimental effects were significantly lower in the animals (T3) receiving a YCW supplemented feed (*p* < 0.05). The analysis of ZEA and metabolites in blood, kidney and liver showed a concentration below the LOQs (≤0.07 μg g^−1^). In urine and feces, only ZEA, α-ZOL and β-ZOL were detected. Despite the low level of ZEA in control diet (T1), excretion of ZEA and metabolites was recorded in urine and feces during the all study, and was not affected by the time of exposure (*p* > 0.05). In urine samples of day 42, the mean amount (±SEM) of total metabolites (ZEA, α-ZOL and β-ZOL), normalized for the creatinine content, was 300 ± 95 nmol/mmol of creatinine (*n* = 8). During chronic exposure by the T2 diet, ZEA + metabolites content in urine increased substantially and significantly with respect to the control (*p* < 0.001). This increase showed an exponential relationship with the time, being 13034 ± 3357 nmol/mmol of creatinine at day 42. Animals treated with YCW (T3) excreted a significantly lower and constant amount of toxins during the time. The values of urinary excretion were not affected by the big variability encountered with T2. At day 42, the urines of T3 group had 68% less toxins than T2 (4189 ± 1132 nmol/mmol of creatinine) (*p* < 0.001). At this time, the mean percentages of dietary ZEA excreted as biomarker (ZEA + metabolites) in 24 h post dose urines were 9.1 and 4.1% of the ingested dose for the T2 and the T3 groups, respectively. In all cases, fecal excretion of dietary ZEA was higher than urinary excretion, and was not affected by the time of exposure. At day 42, mean percentage values were 9, 35 and 37% for T1, T2 and T3. YCW supplementation did not affect the amount of ZEA and metabolites excreted by the feces. These findings show that chronic exposure to ZEA leads to pernicious effects on gilts, but YCW addition to the diet can impact the absorption of ZEA and reduce these detrimental effects.

### 8.11. Effect of Acclimatisation of *Aspergillus Flavus* Strains on Aflatoxins Contamination and Colonisation of Pistachio Nuts

Baazeem A. *, Medina A. and Magan N.

Applied Mycology Group, AgriFood Theme, Cranfield University, Cranfield, Bedford MK43 0AL, U.K.

* Correspondence: a.a.baazeem@cranfield.ac.uk

**Abstract:**
*Pistachio nuts can be contaminated by Aspergillus flavus* under warm and humid conditions. This can result in contaminate with aflatoxin B1 (AFB1), classified as a class 1a carcinogen. There have been no studies on the impact that acclimatisation of *A. flavus* to elevated CO_2_ may have on colonisation and aflatoxin B_1_ contamination. Thus the objectives of this study were to examine whether acclimatisation to 1000 ppm elevated CO_2_ of *A. flavus* strains AB3 and AB10 (5 generations) affected AFB_1_ production and mycelial growth under interacting Climate Change conditions and compare this with non-acclimatised cultures. The results of this study showed that acclimatisation influenced growth of one strain while there was no significant effect on another strain when colonising pistachio nuts. For AFB_1_, the production was significantly stimulated after ten days colonisation after acclimatisation for one strain, while there was no significant increase for the other strain. This suggests that there may be intra-strain differences in effects of acclimatisation and this could influence mycotoxin contamination of such commodities as mixed population of contaminant fungi often occurs. More studies are needed on the acclimatisation of fungal pathogens and their effect on crops under climate change scenarios to obtain more accurate data on implications for mycotoxins contamination of economically important commodities.

### 8.12. Evaluation of Maize Hybrids Resistance to Aspergillus Ear Rot and Aflatoxin B_1_ Accumulation in Serbia

Bagi F. ^1,^*, Grahovac M. ^1^, Budakov D. ^1^, Jajić I. ^1^, Barošević T. ^1^, Savić Z. ^1^, Stojšin V. ^1^, Savić D. ^1^, Vučinić S. ^1^ and Stanko H. ^2^

^1^ Faculty of Agriculture, University of Novi Sad, Novi Sad, Serbia

^2^ Agrocentrum d.o.o., Bečej, Serbia

* Correspondence: bagifer@polj.uns.ac.rs

**Abstract:** Contamination of maize by *Aspergillus flavus* is a significant problem in agriculture due to the production of aflatoxins which are harmful for animal and human health. Cultural practices such as crop rotation, tillage, planting date, irrigation and optimal fertilization can minimize infection with *A. flavus* and subsequent aflatoxin accumulation. However, no control strategy is completely effective when environmental conditions are extremely favorable for growth of *A. flavus.* The most effective and economical method for reduction of *Aspergillus* ear rot and aflatoxin production is development of resistant maize genotypes. The aim of this research was to evaluate sensitivity of different maize hybrids to *A. flavus* infection and aflatoxin accumulation. Fifty maize hybrids belonging to different FAO maturity groups were evaluated for sensitivity to ear rot and aflatoxin acummulation during 2016 in field trials with artificial inoculations using colonized tootpicks method during ear development. Trial was set up in Sombor (Serbia). Two toxigenic fungal isolates of *A. flavus* were used for artificial inoculations. The isolates were obtained from maize seeds originating from locality Bečej (Serbia). Mycotoxin production potential of the isolates was confirmed by rapid screening of aflatoxin biosynthesis genes—Cluster Amplification Patterns (CAP) analysis. Inoculation was performed 10–14 days after 50% of plants reached silking phase. Inoculated ears were harvested when kernel reached 14% or less grain moisture. The ears were visually rated using a scale of 1 (complete absence of symptoms) to 7 (76–100% infected kernels). Aflatoxin B_1_ analyses was preformed by ELISA test. Results of this research indicate existence of significant differences in maize hybrids susceptibility to *Aspergillus* ear rot. The lowest susceptibility to *A. flavus* was recorded for hybrids: B10, A6, D8, B1 with disease intensity 1,23–1,5. The highest susceptibility to *A. flavus* was recorded for hybrids: E1, D1, D4, D10, E2, D3 with disease intensity 2,10–2,53. Aflatoxin B_1_ concentration in grain differed significantly among hybrids. Aflatoxin B_1_ was not observed in hybrids: D3, C2, D5. The lowest level of aflatoxin B_1_ was detected in hybrids: E2, D4, D1, E1, C1. The highest level of aflatoxin B_1_ was detected in hybrids: A18, A19, A11. This study could be of great interest for identification and characterisation of potential sources of resistance to *A. flavus* infection and aflatoxin production.

The study was conducted within project tasks of the MyToolBox project. This project received funding from the European Union’s Horizon 2020 research and innovation programme under grant agreement No. 678012.

### 8.13. Multi-Residue Analysis of 18 Regulated Mycotoxins by LC-MS/MS in Food Samples

Baker D. ^1^, Titman C. ^1,^*, Horner J. ^2^, Loftus N. ^1^ and Noe J. ^3^

^1^ Shimadzu, Manchester, UK

^2^ Scientific Analysis Laboratories, Cambridge, UK

^3^ Shimadzu Benelux, Hertogenbosch, NL

* Correspondence: chris.titman@shimadzu.co.uk

**Abstract:** Mycotoxins are one of the most important contaminants in food and feed due to their widespread distribution in the environment and toxic effects on humans and animals. Due to the risks posed by mycotoxins in food they are regulated globally, including, the EU, US, China, Singapore and Brazil. LC-MS/MS is the technique most commonly employed for mycotoxin quantitation in order to achieve the necessary low reporting limits in complex food and feed matrices. Due to the wide range of physical and chemical properties of mycotoxins, different LC-MS/MS methods are typically developed for small groups of compounds with similar properties. Whereas, in this study a single LC-MS/MS method has been developed for the determination of 18 mycotoxins in food safety.

Solvent extracts were provided by Scientific Analysis Laboratories (SAL, UK) following validated extraction protocols. Samples were analysed using the Nexera UHPLC and the LCMS-8060 triple quadrupole detector (Shimadzu, Japan). The scope of the method included aflatoxins (B1, B2, G1, G2), fumonisins (B1, B2, B3), ochratoxin A (OTA) and trichothecenes (3-acetyldeoxynivalenol (3AcDON), 15-acetyldeoxynivalenol (15AcDON), deoxynivalenol (DON), diasteoxyscripanol (DAS), fusarenon-X (FUS X), HT-2, neosolaninol (NEO), nivalenol (NIV), T2, zeareleonone (ZON)) with an analysis cycle time of 12.5 min. Calibration was performed using C13 internal standards spiked during sample extraction.

A single method was developed for the analysis of 18 regulated mycotoxins with a wide range of physical and chemical properties. This method achieves the required EU reporting limits (between 0.1–10 μg/kg) with linear regression coefficients R2 typically greater than 0.998. The LC mobile phase, column and gradient were all optimised and provided chromatographic resolution of 15-acetyldeoxynivalenol and 3-acetyldeoxynivalenol which are typically not separated on column. This preliminary data was achieved using a new PFP column (Maestro PFP). Commonly in mycotoxin analysis an ammonium acetate additive is used in the mobile phase, whereas in this study sensitivity was increased substantially by using an ammonium fluoride additive in the mobile phase. The final method achieved a fast injection to injection cycle time of 12.5 min.

A single LC-MS/MS method for quantification of 18 mycotoxins at EU reporting levels; including on-column separation of 15-acetyldeoxynivalenol and 3-acetyldeoxynivalenol.

### 8.14. Antifungal Activity of Allium Sativum Extracts on Mold

Barhege B.P. *

Laboratory of Biology, Department of Biology-Chemistry, ISP-Bukavu, DR.Congo

* Correspondence: bbarhege@yahoo.fr

**Abstract:** Fungi play an important role in organic compounds mineralization, but they are also responsible for several prejudices to human and his environment. Their effects on crops and preserved foods would cost more than $50 billion a year. Fruits, picked vegetables, and cereals, are very often spoiled by fungi. In 1985, 25% of the world's cereal crops were affected by mycotoxins secreted by some molds, mainly of the genus *Aspergillus, Penicillium, Fusarium, Claviceps and Alternaria*. Some mycotoxins, including aflatoxin, fumonisins, zearalenone, ochratoxin A, are carcinogenic. Aflatoxins can be produced before and after harvesting on many human and animal foods, and particularly on oilseeds, edible nuts and cereals. Some mycotoxins have the potential to alter immune responses and thus reduce resistance to infections when ingested, inhaled or absorbed through the skin. Fighting against mycotoxins means destroying their source of secretion, which are molds without which they cannot be produced. Thus, aqueous extracts of *Allium sativum* were tested in order to assess their effects on the growth and development of airborne molds.

To extract the active ingredient, 40 g of well-peeled *Allium sativum* pods were ground and then macerated in 400 mL of sterile distilled water. Aqueous concentrations of 0%, 10%, 20%, 30%, 40%, 50%, 60% of the basic liquid obtained after filtration were prepared and each of them served as solvent for the preparation of the medium culture (Sabouraud Agar) on which the molds were scattered. After five days incubation at 22 °C., the following results were obtained.


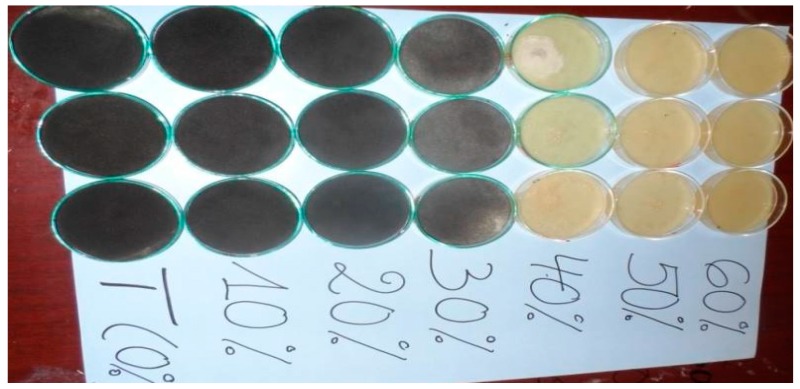


At 0%, 10%, 20%, 30%, all petri dishes showed dramatic growth of molds, given the absence or the low concentration of *Allium sativum* extracts in the solution. From 50%, no trace of mold was visible in Petri dishes (see photo above).

The extracts of *Allium sativum* inhibit the growth and development of molds from 50%. They can be applied in the control of mycotoxins which are their exclusive emanation.

### 8.15. Aflatoxin in Dairy Cow Feed and Raw Milk from Aragón (Spain)

Bervis N., Herrera M., Lorán S., Carramiñana J.J., Juan T., Herrera A. and Ariño A. *

Instituto Agroalimentario de Aragón IA2 (Universidad de Zaragoza—CITA), Veterinary Faculty, 50013, Zaragoza, Spain

* Correspondence: aarino@unizar.es

**Abstract:** Aflatoxins, a group of mycotoxins produced by *Aspergillus flavus* and *Aspergillus parasiticus*, are associated with different disorders in animals and humans. Aflatoxins of the B and G series can be formed both in the field and during storage of raw materials, grains, silage and other feedstuffs and its occurrence is related to environmental and agronomic conditions, pest attacks, and inadequate storage. In addition, the transformation of aflatoxin B1 from feedstuffs consumed by ruminants to aflatoxin M1 secreted in milk depends on several factors as contamination level, feed intake and cow metabolism among others.

The aims of the present study were: (1) to report the natural occurrence of aflatoxin B1, B2, G1 and G2 in cattle feed samples (*n* = 54) such as silage, unifeed and concentrated feed, and (2) to estimate the concentrations of aflatoxin M1 in corresponding raw milk samples (*n* = 22). All samples were collected from 8 dairy cow’s farms located in Aragón (NE Spain) at different times during 2016 and 2017. Aflatoxin analysis (B1, B2, G1, G2 and M1) were carried out by validated methods based on immunoaffinity clean-up (Aflatest WB and Afla M1 columns), determination with liquid chromatography with fluorescence detection, and confirmation by post-column photochemical derivatization (HPLC-FLD-PHRED).

The results showed that aflatoxin B1, B2, G1 and G2 were detected in 41%, 7%, 4% and 5% feedstuffs samples, respectively, while none of the samples were positive for the four aflatoxins. Concentrations detected did not exceed the maximum content (5 μg/kg) established for aflatoxin B1 in compound feed for dairy cattle by the European Union legislation. However, 12 out of 22 milk samples (54.5%) were positive for aflatoxin M1 and four of them (18%) were above the maximum content of 0.05 μg/kg set by current EU regulations.

Four dairy farms were responsible for aflatoxin M1 contamination in milk samples, but only in two farms there was a substantial carry-over of aflatoxin B1 to M1. In these farms both unifeed and concentrated feed samples were contaminated with aflatoxin B1 and the corresponding raw milk samples were subsequently contaminated with aflatoxin M1 at different sampling times during the year.

These results indicate the carry-over of aflatoxins from feed to milk resulting in human exposure to aflatoxin M1. Good farming practices focused on the prevention, reduction and selection of products intended for animal feed and routine monitoring of milk samples are needed to minimize aflatoxin risk in this region.

**Acknowledgments:** Projects AGL2014-57069-R (MINECO-FEDER) and Grupo Consolidado A01 (Gobierno de Aragón-FEDER). N. Bervis thanks the grant FPU13/04238.

### 8.16. Recombinant Production of AcAP1 Protease of Aspergillus Carbonarius to Study Its Involvement in OTA Degradation

Bleve G. ^1^, Conte A. ^1^, Perrone G. ^2^, Solfrizzo M. ^2^, Mita G. ^1^, Logrieco A.F. ^2^ and Gallo A. ^1,^*

^1^ Institute of Sciences of Food Production, National Research Council, Lecce, Italy

^2^ Institute of Sciences of Food Production, National Research Council, Bari, Italy

* Correspondence: antonia.gallo@ispa.cnr.it

**Abstract:** Ochratoxin A (OTA) undergoes to enzymatic biodegradation by proteolytic enzymes able to hydrolyze its amide bond with consequent formation of ochratoxin α (OTα) and L-β-phenylalanine. This mechanism can be regarded as a detoxification method since OTα and L-β-phenylalanine are considered as less and non-toxic, respectively.

Different microorganisms belonging to bacterial, yeast and fungal species have been reported to degrade OTA. Several enzymes may be involved in microbiological degradation of OTA, such as carboxypeptidase A, lipase, and acid proteases. Also *Aspergillus carbonarius*, one of the most important fungal producer of OTA and the major responsible of OTA contamination of grapes, wine and by-products, turned out to be able to degrade OTA. In the attempt to identify the enzyme able to degrade OTA in this microorganism, a protease encoding gene, located in the genomic region recognized as OTA cluster, has driven our attention. In particular, this gene, namely *Acap1* of *A. carbonarius* strain ITEM 5010, encodes for an aspartic protease and is located downstream of the core genes involved in OTA biosynthesis. *Acap1* gene was isolated and cloned for its characterization. The gene is 1367 bp long and the in silico analyses of the deduced protein sequence of 421 aa revealed that the AcAP1 protein shows the functional typical structure of aspartic protease enzymes. Aspartyl proteases are a highly specific family of proteases that tend to cleave dipeptide bond and they are optimally active at acidic pH.

Heterologous recombinant production of the AcAP1 protein has been carried out in order to verify the involvement of AcAP1 in the ability of A. carbonarius in OTA degradation and to analyze its structural and functional properties for a potential biotechnological use of the enzyme. Acap1 gene was cloned in two expression vectors (p426 and pYES), carrying a constitutive and an inducible promoter, respectively, in fusion with a sequence encoding for a His-tag at the 3’-terminus. Three different strains of Saccharomyces cerevisiae, carrying diverse genotypes, have been transformed. Data concerning the protein expression by yeast, evaluation of the protease activity, and purification of the recombinant protein will be produced.

The present work has received funding by the European Union’s Horizon2020 Research and innovation programme under Grant Agreement No. 678781 (MycoKey).

### 8.17. Growth of Pleurotus eryngii on Aflatoxin B_1_-Contaminated Substrate and Its Degradation

Branà M.T. ^1^, Cimmarusti M.T. ^1,2^, Haidukowski M. ^1^, Logrieco A.F. ^1,^* and Altomare C. ^1^

^1^ Institute of Sciences of Food Production, National Research Council of Italy, Bari, Italy

^2^ Department of Economics, University of Foggia, Foggia, Italy

* Correspondence: antonio.logrieco@ispa.cnr.it

**Abstract:** Aflatoxin B1 (AFB_1_) is the most harmful mycotoxin produced mainly by filamentous fungi *Aspergillus flavus* and *A. parasiticus*, which can occur as natural contaminants of many agricultural commodities, including maize. Several approaches have been experimented for the removal of aflatoxin from contaminated food and feed, including microbial degradation which, however, has not been so far implemented into practical technologies due to concerns on the quality and safety of the decontaminated materials. The aim of this study was to investigate the capability of the edible and cultivated mushroom *Pleurotus eryngii* (king oyster mushroom) to degrade AFB_1_. To this purpouse, nine isolates of *P. eryngii* were grown on one liquid and one agar medium supplemented with 500 ng/mL of AFB_1_. After 30 days of growth at 30 ± 1 °C both culture media were processed and analyzed for AFB_1_ by ultra performance liquid chromatography (UPLC/FLD). In the liquid medium (malt extract broth) all the isolates completely detoxified AFB_1_. In the solid medium (malt extract-agar supplemented with corn flour and wheat, MEASM) the detoxification ranged from 65 to 84%. In the perspective of developing a technology for bioconversion of aflatoxin-contaminated cereals into added-value feeds, we carried out a pilot study at a laboratory-scale on degradation of AFB_1_ incorporated in a mushroom cultivation substrate similar to that used in mushroom farms. One strain of *P. eryngii* (ITEM 13681) was able to bioconvert up to 86% of the AFB_1_ initially contained in the substrate in 28 days. This value did not change significantly until the maturation of fruit bodies, 42 day post inoculation. The presence of 25% aflatoxin-contaminated maize in the mushroom substrate did not result in a significant reduction of either biological efficiency or yield of the mushroom cultivation. Finally, the analysis of mature fruit bodies did not reveal the presence of detectable amounts of AFB_1_ or its metabolite aflatoxicol, thus ruling out translocation or “carry-over” of AFB_1_ through the fungal thallus. These findings make a contribution towards the development of a novel technology for remediation of AFB_1_-contaminated corn through the exploitation of the degradative capability of *P. eryngii*.

This work was supported by H2020-MycoKey-(E.U.3.2-678781).

### 8.18. Aflatoxin-Degradative Capability of a Crude Extract from the Spent Substrate of Pleurotus eryngii Cultivation

Branà M. ^1^, Sergio L. ^1^, Cimmarusti M.T. ^1,2^, Haidukowski M. ^1,^ Logrieco A. F. ^1^ and Altomare C. ^1,^*

^1^ Institute of Sciences of Food Production, National Research Council of Italy, Bari, Italy

^2^ Department of Economics, University of Foggia, Foggia, Italy

* Correspondence: claudio.altomare@ispa.cnr.it

**Abstract:** Biological degradation of mycotoxins is an emerging strategy for detoxification of agricultural commodities. In particular, enzymatic degradation of aflatoxin B1 (AFB_1_), the most harmful among the mycotoxin known which may occur as contaminant of most of food and feed, has lately raised considerable scientific interest. Ligninolytic enzymes, such as laccase and peroxidise, from white-rot fungi have been proven to be able to break the highly stable molecule of AFB_1_. However, the high cost of production and purification of these enzymes have limited their implementation into practical technologies aimed at reduction of aflatoxin contamination in the food and feed chains. Among the white-rot fungi there are also cultivable edible mushrooms, such as *Pleurotus* spp. Every year tons of spent mushroom substrate (SMS) are produced as a by-product of mushroom cultivation and disposed at a cost for farmers. However, SMS may still be a source of bioactive compounds, including ligninolytic enzymes potentially useful for degradation of aflatoxin. We investigated the AFB_1_-degradative activity of a crude extract (CE) of SMS and undertook a study for characterization of enzyme content and stability of the extracts. CE of SMS was obtained by an extraction buffer (sodium phosphate buffer 0.1 M, pH 7.3) and the extract was incubated with 1 μg/mL of AFB_1_ at 25 °C, under continuous shaking at 120 rpm for 1, 3 and 7 days; then the aflatoxin content was determined by ultra performance liquid chromatography (UPLC). After 1 day of incubation, the CE was able to degrade more than 50% of AFB_1_ and after 3 and 7 days of incubation the percentage of degradation reached the values of 75% and 90%, respectively. The CE contained a high level of laccase activity, quantified in 4 Units per gram of SMS dry weight (U/g DW) and low level of Mn-peroxidase (0.4 U/g DW), as determined by spectrophotometric assays. The enzymatic activity of the CE had its optimum at temperature ranging between 5 and 25 °C and at pH 4, and it was stable at +4 °C for about 60 days. Heat treatment at 100 °C for 10 min completely destroyed the degradative activity of CE, and freeze drying resulted in a decrease by 35% of the laccase activity.

Based on these preliminary results, SMS proved to be a suitable source of aflatoxin-degrading enzymes and the use of SMS and/or their CE for detoxification of aflatoxin-contaminated commodities, particularly those intended for feed, appears as a coinceivable technology for aflatoxin-free feed production. Further research is needed to improve the stability of SMS extracts and to implement their use in the pipeline of feed processing.

This work was supported by H2020-MycoKey-(E.U.3.2-678781).

### 8.19. Determination of Citrinin in Red Yeast Rice: A Comparison of Immunoaffinity Clean-Up with HPLC and LC-MS/MS

Brown P. ^1^, McGeehan J. ^1^, Wilcox J. ^1^, Manning E. ^1^, Milligan C. ^1,^* and Niemeijer R. ^2^

^1^ R-Biopharm Rhône, Glasgow, UK

^2^ R-Biopharm AG, Darmstadt, Germany

* Correspondence: claire@r-biopharmrhone.com

**Abstract:** Citrinin is produced by a number of *Aspergillus* and *Penicillium* fungi and has been found in a variety of foods such as grains, cheese and red yeast rice, the latter of which is regulated in the EU (Commission Regulation (EU) No. 212/2014). Although legislation is currently in place for only red yeast rice there is considerable interest in Europe with regards to levels in foods including cereals as this toxin is considered as a potential issue during storage often occurring simultaneously with ochratoxin A with both toxins considered as potential agents of Balkan endemic nephropathy.

R-Biopharm Rhône have developed a new immunoaffinity column that selectively isolates and concentrates citrinin from a wide range of commodities including cereals and red yeast rice. Red yeast rice is available as a loose powder and also as a food supplement prepared in tablet or capsule form. Samples were analysed using immunoaffinity clean up with HPLC and the results compared with an LC-MS/MS method.

### 8.20. A New *In Vivo* Model to Evaluate Mycotoxin Detoxification Solutions in Animals

Bruinenberg P. ^1,^* and Carillo C.A. ^2^

^1^ R&D, Trouw Nutrition, Amersfoort, The Netherlands

^2^ Poultry and Rabbit Research Centre, Trouw Nutrition, Casarrubios del Monte, Spain

* Correspondence: paul.bruinenberg@trouwnutrition.com

**Abstract:** While in vitro experiments are a key element of a prescreen strategy of a large number of candidate mycotoxin detoxifiers, in vivo studies are obligatory to evaluate the efficacy of novel mycotoxin solutions in animals. We have established and validated a novel bioassay unit that allows for fast in vivo screening of a large number of mycotoxin solutions in feed. This bioassay unit comprises of 104 wire cages of 39 × 80 × 60 cm, each of which can hold 6–10 chickens, dependent on the age and duration of the study. Each cage is equipped with an independent galvanized metal feeder trough of 36 cm and a nipple drinker connected to individual water tank. Heating, cooling and forced ventilation systems allow the building temperature to be managed according to animal requirements. The bioassay unit allows in vivo assessment of the effect of up to 20 different anti-mycotoxin agents simultaneously. In a pilot study we have evaluated the impact on broilers of a multiple mycotoxin challenge consisting of purified preparations of Aflatoxin B1, Ochratoxin A and T-2 toxin, each dosed at 15 times the EU recommendation for maximal tolerable concentration in feed. The effect of these mycotoxins was assessed by monitoring animal performance indicators (body weight, average daily feed intake, average daily gain and feed conversion rate), the relative weight of target organs such as liver, heart, spleen and on haematological and biochemical serum parameters. Over the complete study period no statistically significant differences were observed in the animal health biomarkers and performance indicators between the positive (no mycotoxins added in the feed) and negative control groups (with mycotoxins in the feed). The reason for the relative minor impact of multiple mycotoxins on broiler performance and health may be due to the high purity of the mycotoxin preparations used in this study. In the last years it has become clear that naturally mycotoxin-contaminated commodities, harbor masked mycotoxins that escape commonly used methods of analysis, but can release their toxic precursors after hydrolysis in animals. The results of our pilot study suggest that these masked mycotoxins and maybe other secondary fungal metabolites add to the overall toxicity. Another implication of this study could be that apart from a mycotoxin challenge, a secondary challenge with infectious disease is needed to achieve a significant animal response in this model. Future work will address both aspects to further optimize our in vivo model.

### 8.21. Comparison of Commercial Available Complex Mycotoxin Binders on *In Vitro* Mycotoxin Binding Properties

Bruneel B. ^1,^*, Segers L. ^1^, van der Aa A. ^1^, Detavernier C. ^2^, De Boevre M. ^2^ and De Saeger S. ^2^

^1^ Orffa Additives BV, Werkendam, The Netherlands

^2^ Laboratory of Food Analysis, Faculty of Pharmaceutical Sciences, Ghent University, Ottergemsesteenweg 460, 9000 Ghent, Belgium

* Correspondence: Bruneel@orffa.com

**Abstract:** Clay-based binders play an important role in the prevention of mycotoxicosis in livestock. Next to this, yeast-based organic components, other organic components and chemical substances can be a good addition to relieve the animal of mycotoxic stress, by binding or otherwise. Multiple in vitro trials were performed to assess the binding capacity of different types of binders. The in vitro experiments were designed in close collaboration with MYTOX (Ghent University, Belgium), and executed by the Laboratory of Food Analysis (Ghent University, Belgium). The mycotoxins ochratoxine A (OTA), zearalenone (ZEN), deoxynivalenol (DON), fumonisin B1/B2 (FUMB1, FUMB2), aflatoxin B1/B2/G1/G2 (AFB1, AFB2, AFG1, AFG2), HT-2 toxin (HT-2), T-2 toxin (T-2) and enniatin B (ENN B) were mixed into a buffer solution together with the different binders (0.5%) at pH 3 (one solution per binder). Under gentle, constant shaking (to mimic peristalsis of the gastro-intestinal tract), these solutions were kept at pH 3 for one hour, and analyzed by LC-MS/MS. The remaining solution was brought to pH 7 (by adding NaOH), to mimic the condition in the intestine, and kept stable for three hours. Afterwards a sample was analyzed by LC-MS/MS. Clay-based binders possess high binding properties towards the tested aflatoxins and ENN B. OTA and the tested trichothecenes (DON, T-2 and HT-2) were hardly bound by the majority of the tested binders, and there was a large variety between pH 3 and pH 7. For ZEN, a large variety could be observed between different binders. Clay-based binders and yeast-based binders show the highest binding efficiencies towards ZEN. For the tested fumonisins (FUMB1, FUMB2), many binders had a very high binding efficiency at pH 3 (as high as 100%), but poorly bound at pH 7 (as low as 0%). Based on these results, an optimal mixture of the ingredients with high-binding properties was designed (Excential Toxin Plus by Orffa). This mixture was compared to 11 commercially available mycotoxin binders in the same in vitro model. Five of them were products selected on the basis of their worldwide presence in the mycotoxin binder market. All products showed a very high binding of the tested aflatoxins and ENN B. Towards the binding of ZEN, there was a large variety between products. A pH effect could also be observed. The tested trichothecenes were difficult to bind at any pH, and only one product showed overall binding (DON excluded). As recovery of the tricothecenes (DON in particular) in the supernatant was high, biotransformation by any ingredient into less toxic metabolites by the commercial available binders was minimal. Fumonisins were difficult to bind, especially at pH 7, but some products were able to bind at both pH 3 and 7. It can be concluded from this last test that there are differences in mycotoxin binding efficiencies in vitro between commercial products, although some commercial binders have a higher binding efficiency towards specific mycotoxins.

### 8.22. The Effect of Phenolic Compounds on the Growth of Mycotoxin-Producing Fusarium Species in Maize Grain

Cassiem A., Viljoen A. and Rose L.J. ^1,^*

Stellenbosch University, Department of Plant Pathology, Private Bag X1, Matieland 7602, South Africa

* Correspondence: lindym@sun.ac.za

**Abstract:**
*Fusarium ear rot* (FER) and Gibberella ear rot (GER) are two important fungal diseases affecting maize *kernels. The main causal agents of FER are*
*Fusarium verticillioides*, *F. proliferatum* and *F. subglutinans*, while GER is caused by species within the *F. graminearum* species complex (FGSC); most notably *F. graminearum* s.s. and *F. boothii*. Infection of maize grain with *Fusarium* species reduces yield and quality. *Additionally, these fungi produce* mycotoxins that are associated with harmful effects in humans and animals. Host resistance is considered the best option to manage FER, GER and *Fusarium* mycotoxins. An important component of the resistance response in plants against fungal attack is the production of secondary metabolites such as phenolic compounds. The effect of phenolic compounds on the growth and toxin production of FER and GER pathogens was, therefore, investigated in this study. Six phenolic compounds; namely vanillic-, trans-ferulic-, caffeic-, p-coumaric-, chlorogenic- and sinapic acid; were evaluated at three concentrations (0.5; 1.5, 2.5 mM) against FER and GER pathogens. The *Fusarium* isolates were grown on PDA amended with phenolic compounds, and the colony diameter was measured after 5 days. The surface area of colonies was calculated and subjected to analyses of variance. Significant interactions between isolate (species), phenolic compound and phenolic concentration were determined. *Fusarium subglutinans* was most sensitive to all the phenolic compounds, and trans-ferulic acid was the most effective phenolic compound against all *Fusarium* species. The highest concentration (2.5 mM) of the phenolic compounds reduced fungal growth most. *Fusarium graminearum* s.s. and *F. boothii* was least affected by all the phenolic compounds. The effect of phenolic compounds on toxin production of FER pathogens was also assessed. Sterile rice, amended with phenolic compounds, were inoculated with fungal isolates, toxins were extracted after 1 week and analysed by liquid chromatography tandem mass spectrometry. None of the phenolic compounds reduced toxin production significantly when compared to the control. The exception was *F. verticillioides* isolate MRC 8559 with sinapic acid (0.5 mM), which produced less toxins and trans-ferulic acid (2.5 mM), which produced more toxins when compared to the control. This study demonstrates that phenolic compounds inhibit the growth of FER and GER pathogens, but not their mycotoxins.

### 8.23. Use of Toxicokinetic Modeling in Mycotoxin Research

Catteuw A. *, De Baere S., Lauwers M., Broekaert N., Antonissen G., Devreese M. and Croubels S.

Department of Pharmacology, Toxicology and Biochemistry, Faculty of Veterinary Medicine, Ghent University, Salisburylaan 133, 9820 Merelbeke, Belgium

* Correspondence: Amelie.Catteuw@UGent.be

**Abstract:** Mycotoxins are considered to be the most hazardous of all food and feed contaminants in terms of chronic toxicity^1^. Recent studies suggest that 72% of world’s cereals are contaminated. They cause important economic losses in livestock production, and are a hazard to public health and animal welfare. The European Food Safety Authority (EFSA) requests toxicological, including toxicokinetic, data in order to perform risk analysis concerning mycotoxins. In vivo toxicokinetic (TK) studies are studies that describe the fate of a toxin in the body, depending on absorption, distribution, metabolism and excretion (ADME) processes.

Toxicokinetic modeling, i.e., mathematical descriptions of the ADME processes, find its application in different fields of mycotoxin research. Usually compartmental modeling is used where the absorption and disposition of the toxin is described using one, two or more compartments. However, this requires intensive sampling strategies which is not always feasible for the animal. Therefore, more novel approaches including population TK (non-linear mixed effect modeling) and physiologically based TK (PBTK) are finding their way into toxicological modeling in veterinary medicine.

First, TK models are of importance in two out of four steps of toxin risk assessment, as defined by the European Commission^2^. TK models contribute to hazard characterisation, by describing the systemic exposure to the (modified) mycotoxin. This involves determination of the systemic absorption and bioavailability of the toxin, quantification of ADME processes and definition of possible species differences. Additionally, TK studies reveal possible relationship between systemic exposure and contamination levels of the toxin. Based on the risk assessment, implementations can be provided in the management of mycotoxin contamination, including maximum levels in food and feed and recommended good agricultural, storage and processing practices.

Second, TK models can be used for efficacy testing of candidate mycotoxin detoxifying agents. In recent guidelines, EFSA stated that in vitro tests do not fully prove the efficacy of mycotoxin detoxifying agent and that in vivo TK trials should be performed^3^.

Third, TK studies reveal biotransformation pathways of mycotoxins, and possible phase I and phase II metabolites that may be proposed as biomarkers in plasma, urine and/or faeces to determine mycotoxin exposure in different animal species. Examples of these three application fields and the different modeling methods will be presented at the conference.

## References

Streit et al., 2013, J Sci Food Agric, 93, 2892–2899.European Counsil, 178/2002, OJEC, 178, 1–24.EFSA, 2010, EFSA J, 8.

### 8.24. Fumonisin Occurrence in Wheat-Based Products for Human Consumption Marketed in Argentina

Cendoya E. ^1^, Nichea M.J. ^1^, Farnochi M.C. ^1^, Sulyok M. ^2^, Chulze, S. ^1^ and Ramirez M.L. ^1,^*

^1^ Micología, Departamento de Microbiología e Inmunología, Facultad de Ciencias Exactas Fco-Qcas y Naturales, Universidad Nacional de Rio Cuarto, Rio Cuarto, Argentina

^2^ Center for Analytical Chemistry, Department for Agrobiotechnology (IFA-Tulln), University of Natural Resources and Life Sciences, Vienna (BOKU), Tulln, Austria

* Correspondence: mramirez@exa.unrc.edu.ar

**Abstract:** Fumonisins are toxic fungal metabolites produced mainly by *Fusarium* species. Among all fumonisins, Fumonisin B_1_ (FB_1_) is the most significant in terms of occurrence and toxicity. In some human populations consumption of fumonisin-contaminated maize has been epidemiologically associated with esophageal cancer and neural tube defects. Fumonisins are geographically widely distributed and their natural occurrence has been reported mostly in maize, but also in other grains and grain-based products such as wheat, wheat-based foods, semolina, farro, bread and others. This is important since wheat is the most important cereal consumed by the Argentine population. In this country human consumption of products manufactured with wheat, either semolina or bread is much greater than for products made from other cereals. Moreover fumonisin presence in wheat samples has been reported in many countries including Argentina, but there is no available data on fumonisin prevalence in wheat-based products in Argentina. In the present study, 46 wheat-based samples, commonly used for human consumption, were analyzed in order to determinate the presence of FB_1_ and FB_2_. Samples (000 flour, 0000 flour, leavening flour, whole meal flour, bran, wheat semolina, and different mixtures for bakery products) were obtained from different local markets at Rio Cuarto city, Argentina in 2015. Mycotoxins were extracted using SAX affinity columns, and analysed by HPLC LC/MS/MS. Fumonisin (FB_1_ + FB_2_) contamination was present in 100% of total samples ranging from 0.05 to 18.94 ng/g (Median: 1.43 ng/g) and most of the samples had more FB_2_ than FB_1_. All 000 flour samples (12) were contaminated with both mycotoxins and 8 out of 12 had higher levels of FB_2_ than FB_1_. Regarding 0000 flour samples, 17 out of 18 samples were contaminated with FB_1_ and FB_2_, and 33.3% of the sample presented higher levels of FB_2_ than FB_1_. This is the first report of fumonisin occurrence in wheat-based products, used for human consumption, collected from supermarkets in Argentina.

### 8.25. Quantification of Multi-Mycotoxins in Animal Feeds from Gauteng Province Using Uhplc-Qtof-Ms/Ms

Changwa R. *, Abia W.A. and Njobeh P.B.

Department of Biotechnology and Food Technology, Faculty of Science, University of Johannesburg, P.O. Box 17011, Doornfontein Campus, Johannesburg, South Africa

* Correspondence: naledichangwa@gmail.com

**Abstract:** Mycotoxins are toxic fungal metabolites causing serious problems worldwide, with dairy farming largely affected. Additionally, the potential for carry-over of mycotoxins from animals to humans via consumption of contaminated animal by-products remains apparent, making mycotoxin surveillance and monitoring a necessity in preserving public health. The overall aims of this study were thus to establish an understanding of Gauteng province dairy farmer perception on mycotoxins and subsequently determine the degree with which their dairy cattle feeds are contaminated by mycotoxins. A socio-demographic survey was thus undertaken in the Gauteng Province of South Africa, where 13 dairy farms from various locations in the region participated by completing questionnaires. Additionally, a total of 40 dairy feeds and feed ingredients were collected and analyzed for multi-mycotoxin contamination. Estimated levels of aflatoxins (AFB_1_, AFB_2_, AFG_1_ and AFG_2_), fumonisin B_1_ (FB_1_), ochratoxin A (OTA), citrinin (CIT), zearalenone (ZEN), α-zearalenol (α-ZEL), β-zearalenol (β-ZEL), deoxynivalenol (DON), 3- and 15-acetyl-deoxynivealenol (ADONs), HT-2 toxin (HT-2) and beauvericin (BEA) were established using an ultra-high performance liquid chromatography coupled with quadrupole time-of-flight tandem mass spectrometry (UHPLC-QTOF–MS/MS) with limits of detection (LODs) and quantification (LOQs) in the range of 0.02–3.46 ppb and 0.06–11.52 ppb, respectively.

Perception studies revealed a limited understanding of mycotoxin legislation and modern forms of mitigation strategies the farmers may have access to. With regards to mycotoxin contamination, data revealed the highest incidence of 100% for AFG_2_ (range: <LOQ−116.1 ppb), α-ZEL (range: 0.98–13.24 ppb) and β-ZEL (range: 0.73–4.71 ppb), followed by AFB_2_ at 92.5% (range: <LOQ − 23.88 ppb), BEA at 90% (range: <LOQ − 55.99 ppb), HT-2 at 87.5% (range: <LOQ − 312.95 ppb) and FB_1_ at 85% (range:<LOQ − 1389.62 ppb). Both DON (range: <LOQ − 81.61 ppb) and ZEN (range: <LOQ − 28.04 ppb) each occurred in 60% of samples analyzed, whereas AFG_1_ (range: <LOQ − 19.96 ppb) and AFB_1_ (range: <LOQ − 3.33 ppb) were found at 55 and 47.5%, respectively. The incidence of 3- and 15-ADON was generally low (30%) ranging between <LOQ and 9.51 ppb. None of the samples analyzed contained CIT and OTA. Apart from those samples that exceeded FDA regulatory limit for total AFs in dairy feeds (20 ppb) due to the high amounts of AFG_2_ and AFB_2_, the levels of other mycotoxins found may be regarded as safe for dairy cattle production in South Africa. This study documents for the first time the natural occurrence of the cold climate-based HT-2 toxin in South African feeds with levels of the toxin as high as 312.9 ppb being detected, however below the EC recommendation level of 500 ppb for animal feeds.

### 8.26. Effect of Water Activity and Temperature on Growth of Fusarium Meridionale and Deoxynivalenol and Nivalenol Production

Chiotta M.L., Rybecky A.I. and Chulze S.N. *

epartment of Microbiology and Immunology, Faculty of Exact Sciences, Physic Chemistry and Natural Sciences National University of Río Cuarto, Río Cuarto, Córdoba, Argentina

* Correspondence: schulze@exa.unrc.edu.ar

**Abstract:** Members of the *Fusarium graminearum* species complex (FGSC) can infect several crops causing diseases such as *Fusarium* head blight in wheat, *Gibberella* ear rot and stalk rot in maize and root rot in soybean. These species can infected the grains which are often contaminated with type B trichothecenes such as deoxinivalenol (DON), nivalenol (NIV) and its acetylated forms. In a previous study done in Argentina was showed that *F. meridionale* was the second species within FGSC more frequently isolated from soybean and showed similar pathogenicity as *F. graminearum* sensu stricto. The aim of this study was: to determine the effect water activity, temperature and strain on *F. meridionale* growth and DON and NIV production on 2% (*w*/*v*) soybean agar. The optimal growth conditions for *F. meridionale* were 0.98–0.995 a_W_ and 25 °C. All the strains reached a higher growth rate at 20 °C in comparison with 30 °C and growth rate increased with increasing a_W_. The analysis on the effect of each factor (temperature, a_W_ and strain) and the combination of them showed that both temperature and a_W_ were the most significant parameters affecting *F. meridionale* growth. Deoxynivalenol production by *F. meridionale* was favoured at 25 °C and 0.96 a_W_ with levels ranging from 0.9 to 46.6 μg g^−1^. None of the strains evaluated showed high production levels at either 20 °C or 30 °C, being higher the production at 30 °C than at 20 °C. At 0.98–0.995 a_W_ and 30 °C conditions DON production increased; while at 20 °C different production patterns were observed depending on the strain and the a_W_ evaluated. As regards NIV production, the toxigenic profile was different depending on the strain. *Fusarium meridionale* B2300 produced the highest levels at 30 °C and 0.98 a_W_, while the optimal NIV production by *F. meridionale* F5043 and F5048 was observed at 20 °C and 0.98 a_W_. These results could be explained based on isolation region of the strain, since *F. meridionale* F5043 and F5048 were isolated from the same geographic area in Argentina, while *F. meridionale* B2300 was isolated from Brazil. Temperature and water activity were the most important factors that affect DON and NIV production, respectively (*p* < 0.05). Both DON and NIV production could be a toxicological risk in soybean, since these toxins could be produced in this crop at pre-harvest stage.

### 8.27. Evaluation of the Mushroom Pleurotus eryngii Mycelium as Biosorbent for Aflatoxin B_1_

Cimmarusti M.T. ^1,2^, Casamassima E. ^3^, Branà M.T. ^1^, Longobardi F. ^3^, Logrieco A.F. ^1^, Haidukowski M. ^1,^* and Altomare C. ^1^

^1^ Institute of Sciences of Food Production, National Research Council of Italy, Bari, Italy

^2^ Department of Economics, University of Foggia, Foggia, Italy

^3^ Department of Chemistry, University of Bari, Bari, Italy

* Correspondence: miriam.haidukowski@ispa.cnr.it

**Abstract:** Aflatoxin B1 (AFB_1_) is a major threat to human and animal health, due to its potent hepatotoxic, carcinogenic and mutagenic effects. Removal or inactivation of aflatoxin in food and feedstuff is difficult. Chemical and physical methods have been found to be effective in detoxification of AFB_1_ from various materials but their use in the practice is limited, due to safety issues and possible loss of nutritional value of the treated commodities. In the feed industry a proposed technology for detoxification of feedstuff is the dietary supplementation of inorganic or organic materials able to adsorb mycotoxins. Once the mycotoxin has been bound, absorption in the digestive tract of animals is strongly reduced. Some mineral adsorbents such as aluminosilicates, bentonites and activated carbons have showed good results but have some drawbacks, such as the negative impact on the nutritional quality of decontaminated feed. For this reason, interest in the use of natural microbial adsorbents has increased. In this study we investigated the capability of ground not-viable mycelium of the edible and cultivated mushroom *Pleurotus eryngii* to bind AFB_1_. *P. eryngii* strain ITEM 13681 was cultured on malt-extract broth for 20 days at 28 °C in the dark. For preparation of the biosorbent, the mycelium was harvested, autoclaved, lyophilized and finely ground to particles of size ≤500 μm. One hundred milligrams of the biosorbent were suspended in 5 mL of phosphate-buffer saline (PBS) pH 7 containing 2000 ng AFB_1_, and incubated overnight at 25 °C in a rotary shaker at 250 rpm. Then, the mycelium and the supernatant were separated by centrifugation and both the phases were analyzed for AFB_1_ content. Sixty-five percent of AFB_1_ was detected in the supernatant, while the mycelium was able to retain 6 ng of AFB_1_ per milligram of dry mycelium (35%). The effect of mycelium dosage was studied by testing different amounts of biomass (25, 50, 75, 100, 150, 200 mg/mL) in both acetate buffer (pH = 5) and PBS (pH = 7) containing 0.5 μg/mL AFB_1_. The suspensions were shaken for 90 min at 250 rpm, at 25 and 37 °C. Then the samples were centrifuged at 13000 rpm for 10 min and the pellets were washed three times and analyzed by UPLC/FLD. The efficiency of adsorption (%A) was calculated using following equation:%A = [(C_i_ − C_f_)/C_i_] × 100; where C_i_ was the initial and the C_f_ final concentration (supernatant plus washing solution) of mycotoxin. The biosorbtion showed to be both dosage- and temperature-dependant. In acetate buffer the mycelium adsorbed 44 ± 4% at the dosage of 200 mg and no significant adsorption at 25 mg. In PBS absorption at 37 °C ranged from 7 ± 5% at 25 mg of mycelium to 64 ± 6% at 200 mg, and at 25 °C it ranged from 3 ± 0% at 25 mg of mycelium to 26 ± 3% at 200 mg. Our results envisage a possible use of *P. eryngii* as a cheap and effective biosorbent for AFB_1_. This mechanism of AFB_1_-binding by *P. eryngii* mycelium is herein demonstrated for the first time. This work was supported by H2020- MycoKey-(E.U.3.2-678781).

### 8.28. Efficacy of Metabolites from Streptomyces AS1 to Control Growth and Ochratoxin A (OTA) and Gliotoxin Production by the Mycotoxigenic Spoilage Species, Penicillium Verrucosum and A. Fumigatus

Danial A.M. *, Medina A. and Magan N.

Applied Mycology Group, Environment and Agrifood Theme, Cranfield University, Cranfield, Beds. MK43 0AL, UK

* Correspondence: a.mohddanial@cranfield.ac.uk

**Abstract:** There is significant interest in the use of natural metabolites from microorganism to control spoilage fungi pathogens in staple food chains. The mycotoxigenic fungal spoilage species *P. verrucosum* contaminates cereals and cereal-based food products which can have impacts during downstream processing. Similar impacts have been reported for animal feed contaminated with *A. fumigatus* which is a health risk not only to livestock but also to humans. This has resulted in the development of strict legislative limits in many regions world-wide such as EU Commission Regulation has implemented the maximum levels of OTA in unprocessed (5.0 ug/kg) and processed cereal products (3.0 ug/kg). The aim of this study was to examine the efficacy of *Streptomyces* AS1 metabolites against *P. verrucosum* and *A. fumigatus* growth and OTA and gliotoxin production. An ethyl acetate (EA) extract (0–50 μg/mL) from the AS1 supernatant were screened for efficacy against growth and OTA and gliotoxin production by *P. verrucosum* and *A. fumigatus*, respectively using a semi-solid YES medium (yeast extract; 20 g/L, sucrose; 150 g/L, magnesium sulphate; 0.5 g/L and 0.05% agar) modified to 0.99 and 0.95 water activity (a_w_) using microtiter plates and the Bioscreen system. A spore concentration of 10^5^ spore/mL was used. The efficacy on growth was measured at 600 nm every 30 min for seven days at 25 °C for *P. verrucosum* and 37 °C for four days for *A. fumigatus*. In addition, OTA and gliotoxin were quantified using HPLC. The minimum inhibitory concentration (MIC) and ED_50_ of EA extract and the antifungal compounds were also determined. Overall, the growth of the fungi decreased as the concentration of EA extract increased. The growth of *P. verrucossum* was significantly decreased (*p* < 0.05) by >70% at 5–50 μg/mL at 0.99 a_w_ and by approx. 60% at 0.95 a_w_. For *A. fumigatus*, the growth was significantly decreased (*p* < 0.05) by >30% at 0.99 a_w_ and approx. 50% at 0.95 a_w_. The production of OTA and gliotoxin were suppressed in the presence of EA extract at both a_w_ levels. EA extract, at all concentration (5 to 50 μg/mL) at 0.95 and 0.99 a_w_ totally inhibited OTA production. For gliotoxin, higher EA extract (>30 μg/mL) was needed to totally inhibited production of gliotoxin at 0.99 a_w_. At 0.95 a_w_ no gliotoxin was detected in the control and all EA extract concentrations. This could be due to low a_w_ (0.95) which might not support the production of secondary metabolites by *A. fumigatus*. The MIC at both a_w_ levels were >50 μg/mL. For ED_50_, except *A. fumigatus* at 0.99 a_w_, the ED_50_ values were <10 μg/mL. Subsequently, a study was carried out to determine the potential antifungal compounds produced by AS1. Four major compounds were identified as cyclo(l-Pro-l-Tyr), valinomycin, cyclo(l-Pro-l-Val) and bafilomycin. This finding shows the potential application of AS1 EA extract which consists of four antifungal compounds as fungistatic antibiotics to control *P. verrucosum* and *A. fumigatus* in food and feed. We are very grateful to Dr M. Sulyok, IFA-Tulln, Austria for analyses of the AS1 extract.

### 8.29. Relationship between Physicochemical Properties of Montmorillonite Clays and Aflatoxin B_1_ Adsorption Efficacy

D’Ascanio V. ^1^, Greco D. ^1^, Menicagli E. ^2^, Biasci A. ^2^, Catucci L. ^3^ and Avantaggiato G. ^1,^*

^1^ Istituto di Scienze delle Produzioni Alimentari (ISPA-CNR), Bari, Italy

^2^ Laviosa Chimica Mineraria SpA, Livorno, Italy

^3^ Università degli Studi di Bari Aldo Moro, Dipartimento di Chimica, Bari, Italy

* Correspondence: giuseppina.avantaggiato@ispa.cnr.it

**Abstract:** Bentonite clays exhibit high adsorptive capacity for contaminants, including mycotoxins. Moderately mycotoxins contaminated grains that cannot be used as food are often directed to animal feed. Economically-feasible detoxification measures for contaminated feeds are needed. Incorporation of bentonites into aflatoxin-contaminated feed is a low cost measure that has shown (based on animal trials) the ability in reducing the bioavailability of these toxic and potent carcinogenic compounds. Recently, the use of bentonites as aflatoxin binder has been regulated (EU regulation No. 1060/2013). The aflatoxin adsorption efficiency of a bentonite depends strongly on the physical, chemical, and mineralogical properties of the smectite, the dominant clay mineral in bentonites. Although screening procedures to select effective aflatoxin binders have been proposed, a correlation between a single mineral property of smectites and aflatoxin adsorption has not been established. The objective of this study was to select effective smectites as aflatoxin adsorbents and to determine the effects of specific physical, chemical and mineralogical properties of the smectites on their adsorption capacity. Twenty nine smectite samples were selected from industrial products or reference minerals. Smectites were analyzed against the published selection criteria for aflatoxin adsorbents: mycotoxin adsorption parameters (maximum adsorption capacity and affinity) determined by the method of adsorption isotherms; pH; cation exchange capacity; particle size distribution determined by laser diffraction particle size analysis; mineralogical/structural compositions measured by X-ray Diffraction and X-ray Fluorescence methods; swell index and viscosity. The detailed mineralogy analysis showed that all smectites samples contained ≥70% of montmorillonite and little amount (<4%) of other minerals (feldspar, quartz and calcite). Montmorillonite is one of the most common smectites and the only bentonite acceptable as aflatoxin binder accordingly to the EU Regulation No. 1060/2013. For the first time, it was observed a strong correlation (*p* < 0.0001) between aflatoxin adsorption parameters (maximum adsorption capacity and affinity) and geological origin of smectites. In addition, mineralogical and chemical analysis confirmed that some physical and chemical properties of smectites correlated (*p* < 0.0001) with adsorption parameters (linear correlation). In accordance with previous observations, this study confirms that even though all bentonite samples contained predominantly montmorillonite their aflatoxin adsorption capacities differed substantially. This emphasizes the importance of evaluating the properties of the clays to assure adequate effectiveness before they are introduced in animal feed.

### 8.30. Development and Validation of an UPLC-MS/MS Multi-Method to Study the Fate of Relevant Mycotoxins in *In Vitro* Rumen Incubations

Debevere S. ^1,4,^*, De Baere S. ^1^, Haesaert G. ^2^, Rychlik M. ^3^, Fievez V. ^4^ and Croubels S. ^1^

^1^ Department of Pharmacology, Toxicology and Biochemistry, Faculty of Veterinary Medicine, Ghent University, Salisburylaan 133, 9820 Merelbeke, Belgium

^2^ Department of Applied Biosciences, Faculty of Bioscience Engineering, Ghent University, Ghent, Belgium

^3^ Chair of Analytical Food Chemistry, Technische Universität München, Freising, Germany

^4^ Department of Animal Production, Faculty of Bioscience Engineering, Ghent University, Ghent, Belgium

* Correspondence: sandra.debevere@ugent.be

**Abstract:** Mycotoxins are more and more associated with subclinical health problems for high productive dairy cows, reflected by vague and non-specific symptoms and periodic decrease in milk production. Indeed, the risk of mycotoxin contamination of dairy diets such as maize silage, a major forage in these diets, is high since they are susceptible to infection with toxigenic fungi. Moreover, considering the wide diversity of toxigenic fungal species on crops and the ability of several fungi to produce more than one mycotoxin, a multiple contamination can be expected. Hence, dairy cows are exposed to various mycotoxins which may lead to depletion of the detoxifying capacity of the microbiota in the rumen. In Belgium, the most important mycotoxins found in maize silage are deoxynivalenol (DON), nivalenol (NIV), zearalenone (ZEN), mycophenolic acid (MPA), roquefortine C (ROQ-C), citrinin (CIT) and enniatine B (ENN B).

The detoxifying capacity of ruminal microbiota for the previously mentioned mycotoxins, especially in combinations, is not well known. To answer this question, in vitro bovine and ovine rumen incubations with single mycotoxins and possible degradation of these mycotoxins after incubation was studied. To determine the mycotoxin concentrations in the samples, a sensitive and specific analytical method for the quantitative determination of the mycotoxins DON, NIV, ZEN, MPA, ROQ-C, CIT and ENN B as well as their metabolites deepoxy-deoxynivalenol (DOM-1), α-zearalenol (α-ZOL), β-zearalenol (β-ZOL), zearalanone (ZAN), α-zearalanol (α-ZAL) and β-zearalanol (β-ZAL) in rumen fluid using UPLC-MS/MS was developed and validated.

Rumen fluid sample clean-up consisted of salting-out liquid-liquid extraction (SALLE), followed by evaporation of the supernatant and resuspension of the dry residue. Sample extracts were analysed using an Acquity H-Class UPLC system with a HSS-T3 column, coupled to a Xevo^®^ TQ-S mass spectrometer (Waters, Zellik, Belgium). The method was in-house validated in accordance with the EU Directive 2002/657/EC and the following parameters were evaluated: linearity, accuracy, within-day and between-day precision, limit of quantification (LOQ), limit of detection (LOD), specificity and carry-over. The method was applied to the analysis of incubation samples and results will be presented at the congress. This research is funded by the government agency Flanders Innovation & Entrepreneurship (VLAIO-Belgium, LA 140971).

### 8.31. Ft-Ir Spectroscopy for Rapid Screening of Ochratoxin a in Wheat

De Girolamo A. ^1,^*, Cervellieri S. ^1^, Cortese M. ^1^, Porricelli A.C.R. ^1^, Pascale M. ^1^, Longobardi F. ^2^, von Holst C. ^3^, Saad A. ^4^ and Lippolis V. ^1^

^1^ Institute of Sciences of Food Production, National Research Council of Italy, Bari, Italy

^2^ University of Bari “Aldo Moro”, Department of Chemistry, Bari, Italy

^3^ European Commission, DG Joint Research Centre, 2400 Geel, Retiesweg 111, Belgium

^4^ Agricultural Engineering Research Institute, Agricultural Research Center (ARC), Giza, Egypt

* Correspondence: annalisa.degirolamo@ispa.cnr.it

**Abstract:** Ochratoxin A (OTA) is a mycotoxin produced by several species of the genera *Aspergillus* and *Penicillium*, and can be frequently found in a variety of foods and beverages, including cereals, coffee, cocoa, spices, beer, wine, grape juice, and dried fruits. Effective monitoring of OTA should be undertaken and achieved through reliable and rapid analysis. Therefore, increased efforts have been made to develop analytical methods suitable for rapid OTA screening.

In the present work the potential of using infrared spectroscopic for the screening of 229 wheat samples naturally contaminated with OTA in the range of <0.15–54 μg/kg was investigated. Samples were analysed by both Fourier transform near- and mid-infrared spectroscopy (FT-NIR, FT-MIR). After a suitable pretreatment of the raw spectral data (baseline in combination with standard normal variate), Partial-Least Squares-Discriminant Analysis (PLS-DA) and Linear Discriminant Analysis (LDA) classification models were used to differentiate highly contaminated durum wheat samples from low contaminated ones and the performances of the resulting models were compared. Models were developed using a *cut-off* limit set at 2 μg/kg OTA that is lower than the EC maximum limit for OTA in unprocessed durum wheat (i.e., 5 μg/kg). The spectral ranges considered were between 7500–4000 cm^−1^ for FT-NIR and 4000–400 cm^−1^ for FT-MIR. For each spectral range, the classification results of the external validation (70 samples) were expressed in terms of average prediction abilities and false compliant rates. The average prediction were 94% for FT-NIR range and 96% for FT-MIR range, independently from the classification model used (i.e., PLS-DA or LDA) thus confirming the reliability of the two statistical approaches used. False compliant rates of 9% were obtained for both spectral ranges and both classification models. These findings indicates that FT-NIR, as well as FT-MIR analysis, might be a promising, inexpensive and easy-to-use screening tool to rapidly discriminate wheat samples for OTA content and verify the compliance with the EU regulatory level. This work has been supported by the Italian Ministry of Education, University and Research (P.O.N. 2007–2013), project S.I.Mi.S.A. “New Strategies for Improvement of Food Safety: Prevention, Control, Correction”.

### 8.32. Hermetic Technology for Reduction of Aflatoxin Exposure in Women and Children under Five Years: A Case Study of Shamva and Makoni Districts, Zimbabwe

Dembedza M.P. ^1^, Nyanga L.K. ^1,^*, Manema L. ^1^, Benhura M.A. ^2^ and Chidewe C. ^2^

^1^ Institute of Food Nutrition and Family Sciences, University of Zimbabwe, Harare, Zimbabwe

^2^ Biochemistry Department, University of Zimbabwe, Harare, Zimbabwe

* Correspondence: nyangael@yahoo.com

**Abstract:** Exposure of women and children to aflatoxins has been reported in several parts of the world including Sub Saharan Africa. Aflatoxin B1 and Aflatoxin M1 are strongly associated with retarded growth in children and increased risk of contracting infectious diseases.Aflatoxin contamination represents one of the most challenging and prominent food safety threats in groundnuts, maize, rice and other grains. In Zimbabwe, maize is the main staple food, with highly seasonal production but it is consumed throughtout the year. Unfortunately the traditional and conventional storage practices cannot guarantee protection against major storage pests and pathogens. Hermetic technology, which involves the use of airtight containers such as metal silos, has been developed and proven to be effective in protecting the harvested grains from attack not only from storage insect but also from rodent pests. However, their effectiveness in reducing mycotoxins contamination in grain is poorly understood. This study therefore, seeked to investigate the efficacy of hermetic storage technology (metal silos and hermetic bags) in reduction of aflatoxin contamination in maize grain and hence, the reduction in exposure of womenand children to these toxins, in Shamva and Makoni districts, Zimbabwe. In the study women and children from households using hermetic technology for storing their maize, and from households using conventional methods were tested for aflatoxin exposure using AFM1 biomarker in urine and breast milk from the women for one full storage season in 2015/2016. Aflatoxin M1 is the most potent metabolite of AFB1 and its detection in urine and breastmilk is a useful tool to estimate ingestion. Aflatoxin M1 in urine and breastmilk of the women and children were analysed by immunoaffinity clean up and using high performance liquid chromatography coupled with a fluorescence detector. In general, the numbers of AFM1 positive urine and breastmilk samples were lower when food supplies were plentiful, but gradually increased with food shortages. The occurrence and levels of AFM1 in urine of women and children, and breastmilk of women from households using hermetic technology to store maize grain was less than those found in women and children from households using conventional methods. The prevalence of AFM1 positive urine samples increased from 3.3 to 7.1%, 10 to 53.4% and 5.1 to 89% during the storage season for women from household using the metal silos, hermetic bags and conventional storage facilities respectively. For breastmilk, the prevalence increased from 0 to 20.6%, 0 to 32.2% and 0 to 87.5% during the storage season for women from household using the metal silos, hermetic bags and conventional storage facilities respectively. The mean levels of AFM1 in urine ranged from 8.6 ± 4.38–42.7 ± 20.29 μgL^−1^, 29.8 ± 9.92–87.6 ± 63.84 μgL^−1^ and 22.5 ± 8.73–161.5 μgL^−1^ during the storage season for women from household using the hermetic metal silos, hermetic bags and conventional storage facilities respectively. Therefore, we concluded that heremtic technology was effective in reducing exposure of women and children to aflatoxins.

### 8.33. Mip Loaded Porous Scaffolds as Spe Sorbent for Ergot Alkaloids

De Middeleer G. ^1,2,^*, Dubruel P. ^2^ and De Saeger S. ^1^

^1^ Laboratory of Food Analysis, Department of Bioanalysis, Faculty of Pharmaceutical Sciences, Ghent University, Belgium

^2^ Polymer Chemistry and Biomaterials Group, Department of Organic and Macromolecular Chemistry, Faculty of Sciences, Ghent University, Belgium

* Correspondence: gilke.demiddeleer@ugent.be

**Abstract:** Mycotoxins are food and feed contaminants produced by various fungal species. Although these secondary metabolites are only present in ppb-ppt concentrations, they are toxic to humans and animals. Within the group of mycotoxins, ergot alkaloids are produced by *Claviceps* species and they occur in cereal-based products. Since infected crops can be processed for consumption, the economic impact should not be underestimated. Therefore, rapid, sensitive and accurate analysis is a strict requirement to guarantee and secure food and feed safety. In general, multi-mycotoxin analysis includes rapid screening and confirmatory methods. Selective recognition elements are required such as molecularly imprinted polymers (MIP) which can bind with different target analytes. This research aims to use MIP for the development of a solid phase extraction (SPE) column prior to ergot alkaloid LC-MS/MS analysis. This application needs to cover the extraction of the six major ergot alkaloids and their corresponding epimers.

Sub-micrometer sized MIP for ergot alkaloids analysis were produced. Equilibrium binding experiments indicated that MIP particles bind higher amounts of metergoline template molecules compared to non-imprinted particles. In addition, different conditions were tested to evaluate MIP binding characteristics for a mixture of the six major ergot alkaloids and their corresponding epimers. Since the final application implies MIP to be immobilized onto poly-ε-caprolactone (PCL) structures, the particles were deposited by means of Pluronic^®^ F127 bismethacrylate (PF127-BMA) hydrogel building blocks. The optimization of the immobilization protocol and selection of the optimal hydrogel building block concentration were first examined on 2D PCL-spincoated glass plates. These immobilization experiments and sol-gel tests showed that 7.5 w %ε and 10 w % of PF127-BMA resulted in successful particle immobilization and respectively a 87.4% and 83.3% gel-fraction of the corresponding hydrogel network. Second, MIP particles were immobilized on 3D structures. Therefore, the Bioplotter^TM^ technology was used to produce 3D PCL scaffolds which were characterized by micrometer sized interconnective pores. The immobilization of MIP onto these scaffolds was successful as shown by SEM analysis. In a next step, the functionality of the MIP particles onto the 3D structures was investigated to examine whether the MIP binding capacity was still sufficient after immobilization on 3D scaffolds. A maximum metergoline rebinding percentage of 44.87 ± 8.30% was obtained. The authors thank the agency for Innovation by Science and Technology (IWT) for the financial support.

### 8.34. Towards A New Spe Sorbent for the Detection of Deoxynivalenol Based on Antibody Loaded Porous Poly-ε-Caprolactone Scaffolds by the Aema Grafting Technology

De Middeleer G. ^1,2,^*, Dubruel P. ^2^ and De Saeger S. ^1^

^1^ Laboratory of Food Analysis, Department of Bioanalysis, Faculty of Pharmaceutical Sciences, Ghent University, Belgium

^2^ Polymer Chemistry and Biomaterials Group, Department of Organic and Macromolecular Chemistry, Faculty of Sciences, Ghent University, Belgium

* Correspondence: gilke.demiddeleer@ugent.be

**Abstract:** Deoxynivalenol (DON) is the most frequently detected mycotoxin within the trichothecenes group. This mycotoxin is mainly produced by *Fusarium graminearum* and *F. culmorum* and most often occurs in grains such as wheat, rye and rice. Exposure of human and animals to DON can result in both acute and chronic health effects ranging from gastroenteritis to inhibition of the protein synthesis. Various analytical methods exist for accurate identification and quantification of the DON content in different matrices. In this respect, recognition elements for a selective detection are of high interest. Antibodies are still the golden standard and are widely used. This research aims to use anti-DON antibodies for the development of a new type of solid phase extraction (SPE) sorbent based on loaded poly-ε-caprolactone scaffolds (PCL).

In a first step, secondary rabbit anti-mouse antibodies were successfully immobilized onto 2D PCL surfaces by applying the 2-aminoethyl methacrylate (AEMA) grafting technology. The presence of AEMA molecules was confirmed by static contact angle (SCA) measurements and X-ray photoelectron spectroscopy (XPS). Next, both an ionic and covalent immobilization model for secondary antibodies were investigated in which four different concentrations were tested. The combination of XPS and radiolabeling results indicated an inefficient immobilization when using the ionic model. Since in the covalent model the 0.0084 μg/μL antibody solution resulted in the highest absolute nitrogen percentage (10.13% ± 0.37) (XPS) and a twentyfold difference between the non-specific and specific grafted amount (radiolabeling), these parameters were selected. Subsequently, covalent immobilization of a 0.0084 μg/μL secondary antibody solution was transferred to the 3D PCL scaffolds. PCL-AEMA grafted scaffolds exhibited a total average of 11.74 ± 0.10 μg grafted antibody whereas non-specific immobilization resulted in 5.89 ± 0.42 μg. The homogeneity of the antibody distribution in the center of the scaffolds and the position of the antibodies on the pores of the carrier structures were confirmed by μCT-SPECT analysis. However, this new SPE sorbent was not capable of sufficiently retaining and/or eluting DON. This can among other be related to an insufficient amount of grafted antibodies or incorrect orientation of the secondary antibodies on the PCL scaffolds. Further optimization is still required to use this type of sorbents in new SPE applications for the detection of mycotoxins. The authors thank the agency for Innovation by Science and Technology (IWT) for the financial support.

### 8.35. Unraveling the Zearalenone Degradation Pathway Using a Poly-Omics Approach

De Mets L. ^1,^*, De Gelder L. ^1^ and Audenaert K. ^2^

^1^ Laboratory of Environmental Technology, Department of Applied Biosciences, Ghent University, Belgium

^2^ Laboratory of Applied Mycology and Phenomics, Department of Applied Biosciences, Ghent University, Belgium

* Correspondence: laura.demets@ugent.be

**Abstract:** Mycotoxins, toxins produced by fungi, are an increasing global threat. These toxins, some of the most toxic and mutagenic substances, pose a serious health risk for humans and livestock. With at least 25% of the world’s food crops contaminated with mycotoxins and climate changes favouring fungal growth, the need for an efficient crop protection system is imperative. As the use of fungicides and good agricultural practices has been proven to be insufficient, the use of microbial detoxification of mycotoxins is gaining momentum. Actinobacteria are known for their versatility in degradation pathways and are a good starting point in the search for mycotoxin degraders.

Currently, a screening of Actinobacteria, including *Streptomyces* sp. and *Rhodococcus* sp., is being conducted. The most promising strains and their degradation pathways will be fully characterized using a poly-omics approach. The full genome of selected strains will be de novo sequenced and used as a reference for RNAseq. Together with a metabolomics analysis, we hope to pinpoint important genes, enzymes and degradation steps. This will allow us to unravel the degradation pathway of zearalenone: a gateway towards practical application in pre- and post-harvest remediation.

### 8.36. Investigations on the Occurrence of Zearalenone in Cocoa-Containing Products

Dinkelacker J.C. ^1^, Hain M. ^1^, Geisen R. ^2^, Schmidt-Heydt M. ^2^, Usleber E. ^1^, Klaffke H. ^3^ and Gross M. ^4,^*

^1^ Dairy Sciences, Institute of Veterinary Food Science, Veterinary Faculty, Justus-Liebig University, Giessen, Germany

^2^ Max Rubner-Institut, Karlsruhe, Germany

^3^ Federal Institute for Risk Assessment, Berlin, Germany

^4^ Veterinary Food Diagnostics, Institute of Veterinary Food Science, Veterinary Faculty, Justus-Liebig-University, Giessen, Germany

* Correspondence: madeleine.gross@vetmed.uni-giessen.de

**Abstract:** Cocoa-containing products are consumed in high amounts all over the world. In Europe, the average yearly per capita consumption in 2014 was between 0.98 kg (Bulgaria)–11.5 kg (Germany). In Belgium, the consumption was 3.8 kg (Statistica, 2017).Therefore, the dietary exposure to natural toxins via chocolate might be of relevance for a high percentage of consumers in Europe. So far, only aflatoxins and ochratoxin A have been reported as natural contaminants in cacao and products thereof. The *Fusarium* mycotoxin zearalenone (ZEN) is frequently found as a contaminant in all major cereal grains, and less frequently has also been found in other plant materials. In this study, cocoa-containing dairy products and various forms of chocolate were analyzed for ZEN by enzyme immunoassay (EIA, detection limit 2 μg/kg).Two samples of ready-to-use whipped cream spray containing cocoa were found positive for ZEN at 5–6 ng/g. Seven out of 28 samples of milk mix drinks with cocoa were positive for ZEN at levels of 1.5–6.5 μg/kg (mean 3.6 μg/kg). Chocolate desserts (*n* = 63) were frequently positive (52%) for ZEN, at levels of 1.1–9.8 μg/kg (mean 3.4 μg/kg). The contamination levels corresponded with the percentage of cocoa in a product and with the type of cocoa. Further analysis of chocolate confirmed this trend. All samples of dark cocoa chocolate (*n* = 11) and white cocoa chocolate (*n* = 2) were positive for ZEN at 3–29 μg/kg, while 75% of brown milk chocolates (*n* = 4) were positive at 2.3–5.2 μg/kg. Selective confirmatory analyses by HPLC-FLD and LC-MS/MS qualitatively confirmed the presence of ZEN, although quantitative agreement was poor. To check the hypothesis that cacao was the source of ZEN, cacao beans were purchased via internet from three different sources. Shells but not nibs from Venezuelan and western African beans were positive for ZEN (approx. 10 ng/g). Mycological culture of beans on malt extract agar yielded three morphologically different fungal isolates, two were tentatively identified as *Aspergillus* spp and were negative for ZEN. One fungal isolate was identified as *Eurotium rubrum* by morphological means and by ITS sequencing, which after culture on malt extract agar for 13 days produced ZEN at levels of approx. 100 ng/g agar plug. *E. rubrum* is not known to produce ZEN and further analysis to confirm these results are needed. This study is the first one about natural occurrence of ZEN in cocoa-containing food products, further extending the mycotoxin spectrum in this commodity.

### 8.37. Analysis of Fumonisins and Their Hydrolysis Products in Biological Matrices

Dohnal I. ^1,^*, Bichl G. ^1^, Schwartz-Zimmermann H.E. ^2^ and Schatzmayr D. ^1^

^1^ BIOMIN Research Center, Tulln, Austria

^2^ Christian Doppler Laboratory for Mycotoxin Metabolism and Center for Analytical Chemistry, Department of Agrobiotechnology (IFA-Tulln), University of Natural Resources and Life Sciences, Vienna (BOKU), Tulln, Austria

* Correspondence: ilse.dohnal@biomin.net

**Abstract:** Fumonisins, mainly fumonisin B_1_ (FB1) and to a lesser extent fumonisin B_2_ (FB2) and B_3_ (FB3), are among the most prevalent mycotoxins worldwide. The most affected commodity is maize. In an annual global mycotoxin survey of animal feed, conducted by BIOMIN, over 2900 samples of maize were analysed in 2016. In 84% of the samples, fumonisins could be detected with an average concentration of 2.6 mg/kg (FB1 + FB2) in all positive samples.

Although the bioavailability of fumonisins is low, they have a considerable negative impact on livestock health and performance. An effective strategy to counteract fumonisins is their enzymatic hydrolysis by the fumonisin carboxylesterase FumD in the gastrointestinal tract of animals. FumD is the active component of the feed additive FUM*zyme* and has been shown to remove both tricarballylic acid side chains of fumonisins. The resulting hydrolysed fumonisins (HFB1, HFB2 and HFB3) have been demonstrated to be much less toxic than the parent fumonisins.

For a comprehensive evaluation of FUM*zyme*, we developed and validated LC-MS/MS-based analysis methods for FB1, FB2 and FB3, as well as for their partially and fully hydrolysed forms, in different biological matrices, including porcine serum and urine. The limited bioavailability of fumonisins makes their analysis in these body fluids challenging, as the analyte concentrations are low and classical enrichment strategies (e.g., by commercial immunoaffinity columns or strong anion exchange) for fumonisins are not applicable to the hydrolysed forms.

We show that the use of stable isotope labelled internal standards improved accuracy (measured as recovery of spiked serum and urine samples) to values between 80% and 110% for all analytes. Moreover, precision of analysis was improved for most analytes by addition of internal standards. The stable isotope labelled standards for fully and partially hydrolysed fumonisins were generated in house by enzymatic hydrolysis of commercially available fully ^13^C isotope labelled FB1, FB2 and FB3.

### 8.38. Enzymatic Degradation of Fumonisins in the Gastroinstenial Tract of Broilers

Doupovec B. ^1,^*, Masching S. ^2^, Bichl G. ^1^, Gruber-Dorninger C. ^1^ and Schatzmayr D. ^1^

^1^ BIOMIN Research Center, Tulln, Austria

^2^ BIOMIN Holding GmbH, Getzersdorf, Austria

* Correspondence: barbara.doupovec@biomin.net

**Abstract:** Fumonisins (FUM) are a group of mycotoxins mainly produced by *Fusarium* species. FUM exert various toxic effects in animals. Poultry species are known to be relatively resistant to FUM, but immune and digestive functions of birds may be impaired by FUM contamination levels below EU guidance levels which may lead to economic losses. A strategy to counteract FUM contamination of feed is the degradation of FUM by a novel feed additive that contains purified fumonisin carboxylesterase FumD (commercial name FUM*zyme*^®^), an enzyme that transforms FUM to non-toxic hydrolysed FUM. To prove the effectiveness of FUM counteracting strategies in vivo, a suitable biomarker has to be analysed. The aim of this study was to evaluate the efficacy of FumD as a FUM degrading feed additive for broiler chickens by determining the concentrations of FUM and their degradation products in excreta as biomarkers of exposure.

Broiler chickens (Ross 308, 1 day old) were randomly assigned to 4 groups with 8 replicate pens (20 birds per pen). The positive control group (A) received feed contaminated with 15 mg FUM/kg. The additive group (B) received feed contaminated with 15 mg/kg FUM and supplied with 0.2% mycotoxin counteracting additive containing FumD. The negative control group (C) received uncontaminated feed. A fourth group (D) received uncontaminated feed with 0.2% mycotoxin counteracting additive. The trial period amounted to 28 days. Birds had free access to mashed feed and water. We determined performance parameters including live weight, weight gain, feed consumption and feed conversion rate. Concentrations of fumonisin B1 (FB1) and its degradation product hydrolysed FB1 (HFB1) were determined in excreta. Statistical analysis was done with ANOVA (IBM SPSS 22.0).

Both additive diets (B and D) had a numerical but—as expected at this contamination level—not statistically significant positive effect on weight and weight gain within the trial period. Compared to the positive control group, the additive group showed significantly lower levels of FB1 and concomitantly significantly higher levels of HFB1 in excreta indicative of a gastrointestinal degradation of FB1 by fumonisin esterase FumD. Consequently, application of FumD as a feed additive represents a promising strategy for counteracting negative effects of FUM contaminated feed on the health of broiler chickens.

**Keywords**: fumonisins; biomarker; feed additive; faeces; broiler

### 8.39. In Vitro Metabolism of Enniatin B1 and Deoxynivalenol by Salmon Liver Microsomes

Faeste C.K. ^1,^*, Johny A. ^1^ and Ivanova L. ^2^

^1^ Toxinology Research Group, Norwegian Veterinary Institute, Oslo, Norway

^2^ Section for Chemistry, Norwegian Veterinary Institute, Oslo, Norway

* Correspondence: christiane.faste@vetinst.no

**Abstract:** Aquaculture production in Norway has grown almost exponentially in the last decades and is depending on the exploitation of non-marine protein sources for aquafeeds. In consequence, plant materials are increasingly used in fish feed, currently making up to 70% of the diet in salmon farming as compared to only 10% in 1990. This complete change has led to new challenges regarding fish health and welfare, and product quality. A major new hazard connected with the transition to agricultural resources is exposure of the fish to mycotoxins and a potential carry-over into fish-derived products. The metabolic fate of important mycotoxins in fish is thus of interest both with regard to fish health and food safety.

In the present study, we have investigated the in vitro metabolism of enniatin B1 (ENNB1) and deoxynivalenol (DON) in liver microsomes prepared from on-growing farmed Atlantic salmon. ENNB1 and DON belong to the most prevalent fungal derivatives in grain in temperate climate zones and have been detected in the liver and edible muscle of different fish from aquaculture (Tolosa et al., 2014; Bernhoft et al., 2017). The microsomal assay conditions were optimised by using different mycotoxin concentrations and incubation times, with the aim of either maximum metabolite production or linear kinetics of ENNB1 and DON depletion. Metabolites were characterised by liquid chromatography high-resolution mass spectrometry (LC-HRMS), and compared to known metabolism products in other species. The incubation of both mycotoxins together indicated that DON could have the potential to slow down EnnB1 metabolism.

## References

Tolosa, J.; Font, G.; Mañes, J.; Ferrer, E.; Natural occurrence of emerging Fusarium mycotoxins in feed and fish from aquaculture. *J. Agric. Food Chem.*
**2014**, *62*, 12462–12470.Bernhoft, A.; Høgåsen, H.R.; Rodenlund, G.; Ivanova, L.; Berntssen, M.H.G.; Alexander, J.; Eriksen, G.E.; Fæste, C.K. Tissue distribution and elimination of deoxynivalenol and ochratoxin A in dietary exposed Atlantic salmon (*Salmo salar*). *Food Add. Contam. A*
**2017**, doi:10.1080/19440049.2017.1321149.

### 8.40. Detection of Genetic Diversity in Iranian Aspergillus flavus Isolates by Ssrs

Fani S.R. ^1,^*, Azmoun H. ^2^, Zamanizadeh H.R. ^2^ and Moradi M. ^3^

^1^ Plant Protection Research Department, Yazd Agricultural and Natural Resources Research and Education Center, AREEO, Yazd, Iran

^2^ Department of Plant Pathology, Science and Research Branch, Islamic Azad University, Tehran, Iran

^3^ Pistachio Research Center, Horticultural Sciences Research Institute, Agricultural Research, Education and Extension Organization (AREEO), Rafsanjan, Iran

* Correspondence: rezafani52@gmail.com

**Abstract:**
*Aspergillus flavus*, the opportunistic, soil-borne and cosmopolitan filamentous fungus is the main producer of hepatocarcinogenic secondary metabolites known as aflatoxins in different crops. The fungus is a complex of toxigenic and nontoxigenic strains. Toxigenicity is regulated by different genes involved in aflatoxin gene cluster. The best strategy currently being applied to mitigate aflatoxins is the use of indigenous atoxigenic VCGs as biocontrol agents in the crop environment to competitively exclude and displace with toxigenic VCGs. The study was aimed to investigate the distribution of genetic variation in 46 L-strain *A. flavus* (23 toxigenic + 23 atoxigenic) isolated from pistachio soil and nut samples collected in different agroecological zones of Iran using six microsatellite loci. Isolates were obtained by dilution plate technique and AFPA medium. Species-specific primers (FLAVIQ1/FLAVIQ2) were used to validate species affiliation of *A. flavus* isolates. The microsatellite markers for loci AF13, AF17, AF25, AF43, AF64 and AF66 were applied to understand genetic diversity in 46 isolates. Results indicated that the number of alleles and the size were ranged from 0–5 and 100–1500 bp, respectively. The isolates were categorized in 17 groups based on the allele amplification patterns. Nine groups were multiple-member including, 2–12 isolates and eight of them were single-member. Seven multiple-groups have either toxigenic or atoxigenic isolates and two remaining groups have only 4 and 2 atoxigenic members. Eight single-member groups including, 4 and 4 toxigenic and atoxigenic isolates, respectively. The isolates of group with four atoxigenic isolates were belonged to Khorasan Razavi (*n* = 3) and Esfahan (*n* = 1) regions. Detection of five genes of the aflatoxin biosynthetic pathway includes *nor*1, *afl*R, *est*A, *ver*1, *avn*A and three flanking region including C1, C3 and C4 among aflatoxin gene cluster of these atoxigenic strains showed that deletion of gene loci in the cluster ranging from 2 to 4. The competitive ability and consequently the ability to limit aflatoxins production were 66.77, 74.22% and 94, 93% for aflatoxins B1 and B2 by Khorasan Razavi and Esfahan isolates respectively under laboratory condition (*p* ≤ 0.05). The overall conclusion is that a combination of these isolates was appropriated for field application after supplementary studies.

This Research was financially supported by The Center for Innovation and Technology Cooperation (CITC) of I. R. of Iran Presidency.

### 8.41. Atoxigenic Iranian Aspergillus flavus Isolates Performance for the Biocontrol of Aflatoxin

Fani S.R. ^1,4,^*, Moradi M. ^2^, Probst C. ^3^, Zamanizadeh H.R. ^4^, Mirabolfathy M. ^5^, Haidukowski M. ^6^ and Logrieco A.F. ^6^

^1^ Plant Protection Research Department, Yazd Agricultural and Natural Resources Research and Education Center, AREEO, Yazd, Iran

^2^ Pistachio Research Center, Horticultural Sciences Research Institute, Agricultural Research, Education and Extension Organization (AREEO), Rafsanjan, Iran

^3^ Washington State University, Irrigated Agriculture Research and Extension Center, Prosser, WA, USA

^4^ Department of Plant Pathology, Science and Research Branch, Islamic Azad University, Tehran, Iran

^5^ Iranian Research Institute of Plant Protection, Agricultural Research, Education and Extension Organization (AREEO), Tehran, Iran

^6^ National Research Council Institute of Sciences of Food Production, Bari, Italy

* Correspondence: rezafani52@gmail.com

**Abstract:** The control of aflatoxins, a group of potent carcinogenic mycotoxins, on pistachio nuts is one of the most serious challenges for consumers, producers and exporters. Among all biological control agents, application of native atoxigenic isolates of *Aspergillus flavus* is the most promising strategy for aflatoxin mitigation through competitive exclusions. To assess competitive ability of atoxigenic strains to reduce aflatoxins, YES medium was co-inoculated with atoxigenic (*n* = 57) and toxigenic (*n* = 1) isolates (all originating from Iranian pistachio orchards) in two-party mixtures with the same conidial concentrations (4000 conidia/per isolate). The aflatoxin B1 + B2 content of YES media was measured using ultra performance liquid chromatography with fluorescence detector (UPLC/FLD). The competitive ability and consequently the ability to limit aflatoxin production was highly variable among all tested atoxigenics and ranged from less than 20% to more than 90% average reduction. Statistical analyses indicated that the 57 atoxigenic isolates could be clustered in five groups. Group 2 isolates (*n* = 20, 34.48% of all isolates) showed the highest potential to competitively exclude toxigenic isolates and subsequently reduce the amount of aflatoxins B1 and B2 produced by 94% and 93%, respectively. Group 4 isolates (*n* = 9, 15.52% of all isolates) ranked second in efficacy reducing aflatoxins B1 and B2 by 66.77 and 74.22%. Reduction of aflatoxins B1 and B2 by group 3 isolates (*n* = 10, 17.24% of all isolates) averaged 37.6 and 55%. A minimal aflatoxin reduction was achieved by isolates in group 1 (*n* = 17, 29.31% of isolates), with an average reduction of 1.47 and 17.64% for aflatoxins B1 and B2, respectively. Unexpectedly, two isolates in group 5 produced more aflatoxin B1 and B2 than the toxigenic isolate alone in the competition test (*p* ≤ 0.05). Detection of six genes of the aflatoxin biosynthetic pathway including *nor*1, *afl*R, *est*A, *ver*1, *avn*A and three flanking region including C1, C3 and C4 among aflatoxin gene cluster of atoxigenic strains showed that deletion of gene loci in the cluster have different patterns among the isolates and deletion ranging from 2 to 7 gene was observed. Atoxigenic isolates foundin group 2 could be candidates for field testing. Further investigations are required to assess gene flow.

### 8.42. Development of a Multi Mycotoxins Analysis Method for Animal Feed Samples Using Agilent 6460 Lc-Ms/Ms

Farkas H. ^1,^*, Raj J. ^1^, Cepela R. ^1^, Vukovic G. ^2^ and Vasiljevic M. ^1^

^1^ Patent Co, Vlade Ćetkovića 1A, 24 211 Mišićevo, Serbia

^2^ Institute of Public Health of Belgrade, Belgrade, Serbia

* Correspondence: hunor.farkas@patent-co.com

**Abstract:** Mycotoxins, secondary metabolites of moulds, are commonly found in food and feed samples. These mycotoxins are toxic to humans and animals. Therefore, it is necessary to test animal feeds for mycotoxins contamination. The aim of this work was to develop a fast and simple UHPLC-based multi-mycotoxin method for the determination and accurate quantitation of all mycotoxins regulated in feed (EU Directive 2002/32/EC, 2006/576/EC and 2013/165/EU) by liquid chromatography coupled with tandem mass spectrometry. The method is based on “dilute and shoot” principle. It involves two step extraction and centrifugation of the extracts. To compensate the matrix effects in electrospray ionization the extracts are mixed with [^13^C] labelled internal standards for each group of mycotoxins (^13^C AB1, ^13^C DON, ^13^C ZON, ^13^C OTA, ^13^C FB1 and ^13^C T-2) before injection onto LC-MS/MS. The method was successfully validated on corn, compound feed, wheat, barley, soya meal, wheat bran, sunflower meal and TMR. Method performance parameters were obtained by in-house validation. The blank samples were spiked with a mixture of 11 mycotoxin standard on two level (LOQ and 10 × LOQ) in 12 replicates. The RSD_r_ of the method were between 2.5% and 13.4% and the apparent recoveries were between 62% and 115% for all analytes. It is concluded that the “dilute and shoot” method with addition of [^13^C] labelled internal standard is capable for determining all EU regulated mycotoxins in animal feed and compound feed.

### 8.43. Gaseous Allyl Isothiocyanate to Protect Soya and Black Beans Against the Growth of Aspergillus parasiticus and Aflatoxin Production

Ferreira Lopes L. ^1^, Rampazzo Favoretto V. ^2^, Zucareli C. ^2^, Meca G. ^3^ and Bittencourt Luciano F. ^1,^*

^1^ Department of Animal Science, School of Life Sciences, Potifícia Universidade Católica do Paraná, Rua Imaculada Conceição 1155, 80901-215 Curitiba, Paraná, Brazil

^2^ Department of Agronomy, Centro de Ciências Agrárias, Universidade Estadual de Londrina, 86051-990, Londrina, Paraná, Brazil

^3^ Laboratorio de Química de los Alimentos y Toxicología de la Facultat de Farmàcia, Universitat de València. Av. Vicent Andrés Estellés s/n, 46100 Burjassot, España

* Correspondence: fernando.luciano@pucpr.br

**Abstract:** Soya beans have been widely planted as source of vegetable oil, biodiesel and as a major ingredient in foods and animal feed. Brazil is the second largest producer (113,923 million tons in the 2016/2017 harvest) and the largest exporter of soy in the world. In addition, black beans are a staple food in Brazil, with a production of 3.07 million tons in 2016/2017, 22% higher than the previous year. As a tropical country, Brazil has a favorable climate for the growth of spoilage fungi in grains and, consequently, mycotoxins are commonly found in stored crops, which poses as a threat to the health of humans and animals. Therefore, allyl isothiocyanate, a natural volatile compound derived from brown and black mustard, was used to fumigate soy and black beans and avoid the growth of aflatoxin-producer *Aspergillus parasiticus*. Samples containing 50 g of soy or black beans were inoculated with 104 spores/g of A. parasiticus CECT 2681 and stored in hermetic glass jars. The relative humidity was controlled using saturated solutions of KCl, which gives a RH of 85%, simulating the average RH of plantation fields in some areas of Southern Brazil. Samples were treated with 0; 1; 5 or 25 μL/L of gaseous AITC and analyzed after 35 days. Microbial counting was performed by homogenizing and serially diluting the samples in peptone water and plating in acidified (pH 3.5) potato dextrose agar. Aflatoxins were extracted, concentrated with immunoaffinity columns and quantified by HPLC-FLD. Humidity of the grains was also followed during the storage. Results have shown that a 2 and 4 log CFU/g reduction of A. parasiticus in soy was produced by 5 μL/L and 25 μL/L of gaseous AITC, respectively. Similarly, 2 and 5-log CFU/g reduction of A. parasiticus was found in black beans treate with 5 μL/L and 25 μL/L of AITC. Moreover, aflatoxin production was totally inhibited by 25 μL/L of AITC in both grains, whereas ~90% inhibition was found at 5 μL/L. Gaseous AITC at 1 μL/L did not inhibit fungal growth in soy and aflatoxin production in soy and black beans. Humidity of soya beans increased from 14 to 18% and from 14.5 to 19% in black beans during storage. Therefore, fumigation of AITC may be an alternative to avoid the growth of A. parasiticus in stored beans and soya. Since these beans are thermally processed before consumption, AITC will evaporate and may not impact their sensory characteristics.

### 8.44. Metagenomic Analysis of an Industrial-Scale Biogas Plant Fed with Contaminated Maize Silage

Ferrara M. ^1^, Liuzzi V.C. ^1^, Fanelli F. ^1^, Haidukowski M. ^1^, Cimmarusti M.T. ^1,2^, Casaletta E. ^3^, Logrieco A.F.^1^, Huson D.H. ^4^ and Mulè G. ^1,^*

^1^ Institute of Sciences of Food Production, National Research Council, Bari, Italy

^2^ Department of Economics, University of Foggia, Foggia, Italy

^3^ AUSTEP S.P.A., Milan, Italy

^4^ Center for Bioinformatics, University of Tübingen, Tübingen, Germany

* Correspondence: giuseppina.mule@ispa.cnr.it

**Abstract:** Biogas production represents one of the most economically attractive alternative technology for biofuel production from renewable resources. Generally, biogas plants are fed with agricultural residual products and food wastes, but the rising up of agricultural products contaminated by mycotoxins, such as maize silage not suitable for animal feeding, has pointed the question on the possibility to use this agricultural productfor biogas production. In this regards, a preliminary metagenomic analysis of microbial community residing in a mesophilic industrial-scale biogas fermenter, daily fed with contaminated maize silage, has been carried out to characterize the evolution of microbial community under the operating conditions and the mycotoxin content. Sample were collected from a biogas plant consisting of a three steps production taking place in a bioreactor, post-reactor and a storage tank. Total DNA was extracted from samples belonging to each steps of biogas production. Metagenomic analysis was carried out by analyzing the V4 variable region of bacterial and archaeal 16S rRNA gene.

Mycotoxin content was analyzed in maize silage feeding the biogas plant and in the digestate from bioreactor, post-reactor and storage tank by immunoaffinity column clean-up (Myco6in1^+®^) and detected with liquid chromatography-tandem mass spectrometry (LC-MS/MS). Over 3 million high quality reads (about 1 GB) were generated on the Ion Torrent S5 Sequencing System. About 2.4 million reads were assigned for 16S analysis. In detail, metagenomic analysis revealed that Bacteria superkingdom was dominant (~96%) along the production steps, whereas Archaea were less represented (~4%). Within Bacteria the most abundant phylum was Firmicutes, mostly represented by Clostridia, followed by Bacteroidetes and Synergistetes. Within the superkingdom of Archaea, only microorganisms belonging to the phylum of Euryarchaeota were detected. Within Euryarchaeota the dominant genera were *Methanosarcina* and *Methanoculleus*. Chemical analysis on maize silage feeding the plants showed an initial mycotoxin contamination by DON (410 μg/kg), FB_1_ (3570 μg/kg), FB_2_ (810 μg/kg) and T-2 toxin (20 μg/kg), while AfB_1_, HT-2 Toxin, NIV, OTA and ZEA were not detected. After the first step of biogas production, a complete reduction of DON and T-2 content was achieved. These preliminary results suggest a possible absorption/degradation of mycotoxins in bioreactor tank and therefore further studies are needed to better elucidate the possible involvement of specific microbial taxa capable of mycotoxins reduction and the enzymatic pathways potentially involved in mycotoxin degradation.

### 8.45. Cost-Effective Sampling and Analysis of a Cereal Batch for Mycotoxins

Focker M. ^1^^,^*, van der Fels-Klerx H.J. ^1^ and Oude Lansink A.G.H.M. ^2^

^1^ RIKILT, Wageningen University and Research, Akkermaalsbos 2, 6708 WB Wageningen, The Netherlands

^2^ Business Economics, Wageningen University. Hollandseweg 1, 6706 KN, Wageningen, The Netherlands

* Correspondence: marlous.focker@wur.nl

**Abstract:** In order to avoid that cereal batches, that are highly contaminated with mycotoxins, end up in the feed or food chain, batches need to be sampled and tested. The precision of a sampling and analytical plan (S&A) is evaluated by its total variance. This variance is caused by the fact that mycotoxins are not homogeneously distributed throughout the cereal batch. The total variance of a S&A plan is the sum of the variance due to taking samples at different locations in the batch, the variance due to preparing the sample for the analysis and the variance due to the detection method used to analyse the sample. The aim of this study is to establish a cost-effective S&A plan to determine the mycotoxin concentration in a cereal batch. Operating Characteristic curves showing the probability to accept the batch for each S&A plan at a range of concentrations were drawn and the number of correct decisions made by each sampling plan were estimated. An optimization model was used to maximize the number of correct decisions, subject to the budget constraint. This research focused on sampling for aflatoxins in a maize batch and DON in a wheat batch. Two approaches were considered: one according to the sampling plan prescribed in the European Commission Regulation where many incremental samples are collected from the batch, put together in one aggregate sample, that is sent to a laboratory, where a subsample is analysed with a precise chromatographic method, e.g., LC/MS. The other approach was the on-site detection approach: a few samples are taken from the batch and are each analysed with a rapid method, e.g., lateral flow devices (commonly called dipsticks). For a given budget, results showed that taking many incremental samples and analysing a subsample with LC/MS led to the highest number of correct decisions. However, analysing a few samples with dipsticks on-site can give quite a fast and fair estimation of the batch concentration. Using dipsticks is especially suitable for DON in wheat, as DON is more homogeneously distributed throughout the batch than aflatoxins, and therefore, a lower amount of samples are needed to achieve the same accuracy.

The study was conducted as part of the MyToolBox project. This project received funding from the European Union’s Horizon 2020 research and innovation programme under grant agreement No. 678012. Additional funding for this study was received from the ministry of Economic Affairs in the Netherlands.

### 8.46. Development of a Rainbow Lateral Flow Immunoassay for Multi-Mycotoxin Detection

Foubert A. ^1,^*, Beloglazova N. ^1^, Gordienko A. ^2^, Tessier M.D. ^3^, Drijvers E. ^3^, Hens Z. ^3^ and De Saeger S. ^1^

^1^ Laboratory of Food Analysis, Faculty of Pharmaceutical Sciences, Ghent University, Ghent, Belgium

^2^ Chemistry Institute, Department of General Inorganic Chemistry, Saratov State University, Saratov, Russia

^3^ Department of Inorganic and Physical Chemistry, Faculty of Sciences, Ghent University, Ghent, Belgium

* Correspondence: astrid.foubert@ugent.be

**Abstract:** Today, with the increased regulatory requirements in food safety, the demand for rapid, sensitive and accurate methods to detect biological and chemical contaminants in food and feed has increased. In particular, tests that can be completed within minutes would enable processors to take quick corrective actions when contaminants are detected, which is also the case for mycotoxins. Hence, rapid methods like the lateral flow immunoassay (LFIA) are rapid, user-friendly and sensitive on-site tests suitable for this purpose. Here, we present the development of a multi-mycotoxin LFIA system by using quantum dots (QD) as innovative label. QDs, small semiconductor nanoparticles, are one of the most promising labels due to their unique spectral properties. They are characterized by a high fluorescence quantum yield, stability against photobleaching, and size-tunable absorption and emission bands. They allow simultaneous use of multiple QDs with different spectral characteristics (multiplexing). The stable photoluminescence makes QDs ideal nanoprobes for chemical, biomedical and therapeutic labeling and imaging.

In this work a multi-mycotoxin LFIA based on the use of green, red and orange-emitted QDs was developed and is able to detect four mycotoxins i.e., deoxynivalenol (DON), zearalenone (ZEN) and T2/HT2 in different matrices (barley and wheat). The test is based on an indirect competitive approach. First, the QDs were solubilized by coating them with amphiphilic polymer (green) or silica (red and orange). Next, the specific antibodies (Abs) were coupled with these water-soluble QDs by carbodiimide chemistry which also made a comparison possible of these differently coated QDs with regard to their bioconjugation. The mycotoxin ovalbumin (OVA) conjugates (DON-OVA, ZEN-OVA and T2-OVA) were synthesized and immobilized as three test lines on the membrane. The test is completed within 15 min and there is no need for any mathematical or statistical processing of the obtained results. This detection method is a user-friendly and sensitive detection method with cut-offs (DON: 1000 μg/kg, ZEN: 80 μg/kg, T2/HT2: 80 μg/kg) according to EU legislation. Moreover, the QD-based LFIA was also compared with gold nanoparticle-based LFIA. This showed that the QD-based LFIA was more sensitive and resulted in less use of antibody and antigen.

This work is financial supported by the BOF Special Research Fund from Ghent University, GOA project No. 01G02213.

### 8.47. Disposition of Enniatin B and B1 in Broiler Chickens and Pigs: Comparative Oral Bioavailability and Toxicokinetics

Fraeyman S. ^1^, Devreese M. ^1^, Antonissen G. ^1,2^, De Baere S. ^1^, Rychlik M. ^3^, Ivanova L. ^4^, Faeste C. ^5^ and Croubels S. ^1,^*

^1^ Department of Pharmacology, Toxicology and Biochemistry, Ghent University, Merelbeke, Belgium

^2^ Department of Pathology, Bacteriology and Poultry Diseases, Ghent University, Merelbeke, Belgium

^3^ Chair of Analytical Food Chemistry, Technische Universität München, Freising, Germany

^4^ Department of Chemistry, Norwegian Veterinary Institute, Oslo, Norway

^5^ Toxinology Research Group, Norwegian Veterinary Institute, Oslo, Norway

* Correspondence: siska.croubels@ugent.be

**Abstract:** The emerging *Fusarium* toxins enniatin B (ENN B) and enniatin B1 (ENN B1) are frequently encountered feed contaminants. Ninety-two percent of the feed samples analysed by Streit et al. (2013) were contaminated with ENN B and B1, with maximum levels up to 780 μg/kg and 2690 μg/kg for ENN B and ENN B1^1^, respectively. Notwithstanding their high prevalence, little is known about the possible effects of ENNs on animal health. To elucidate the absorption, distribution, metabolism and excretion (ADME) processes of these mycotoxins in broiler chickens and pigs, species-specific toxicokinetic studies were performed. Plasma concentrations were determined with an in-house developed and validated LC-MS/MS method and screened for main phase I and II metabolites using UHPLC-HR-MS. The absolute oral bioavailability (F%) and main toxicokinetic characteristics were calculated using two-compartmental (pigs) and non-compartmental analysis (broiler chickens).

Remarkable differences were observed between broiler chickens and pigs. ENN B and ENN B1 are poorly absorbed after oral administration in broiler chickens, with an F of 11% and 5%^2^, respectively. In contrast, ENN B1 was almost completely absorbed in pigs (F = 91%)^3^. In broiler chickens, the total body clearance (Cl) and volume of distribution (V_d_) were 7.1 and 6.6 L/h/kg BW and 34 and 25 L/kg BW for ENN B and ENN B1, respectively^2^. In pigs, Cl was much lower, namely 1.9 L/h/kg BW. The V_d_ in the central and peripheral compartment in pigs was 0.57 and 0.69 L/kg BW, respectively^3^. In pigs, hydroxylated, carbonylated and carboxylated metabolites of ENN B1 were detected^4^, whereas in chickens hydroxylated and carboxylated metabolites of ENN B and B1 were found^2^. Results could indicate a higher susceptibility towards the negative health effects of ENNs for pigs due to the high F% and lower Cl. On the other hand, both the low F% and relatively high Cl of ENN B and B1 in broiler chickens is in accordance with the EFSA statement that systemic adverse health effects of ENNs in poultry are unlikely^5^. The study demonstrates the necessity to perform species-specific toxicokinetic studies.
Streit, E. et al. *Toxins*
**2013**, 5, 504–523.Fraeyman, S. et al. *J. Agric. Food Chem.*
**2016**, *64*, 7259–7264.Devreese, M. et al. *Food Chem. Toxicol.*
**2014**, *63*, 161–165.Ivanova, L. et al. *Food Chem. Toxicol.*
**2017**.European Food Safety Authority. *EFSA J.*
**2014**, *12*, p 174.

### 8.48. Differences in the Beauvericin Gene Cluster and Toxin Production in Fusarium subglutinans and Fusarium temperatum

Fumero M.V. ^1,^*, Villani A. ^2^, Susca A. ^2^, Haidukowski M. ^2^, Cimmarusti M.T. ^2^, Leslie J.F. ^3^, Toomajian C. ^3^, Chulze S. ^1^ and Moretti A. ^2^

^1^ Department of Microbiology and Immunology, Faculty of Exact Sciences, Physic Chemistry and Natural Sciences, National University of Río Cuarto, Río Cuarto, Córdoba, Argentina

^2^ Institute of Sciences of Food Production, CNR, Bari, Italy

^3^ Department of Plant Pathology, Kansas State University, Manhattan, Kansas, USA

* Correspondence: mariaveronicafumero@gmail.com

**Abstract:** Beauvericin (BEA) is a secondary metabolite produced by many species of *Fusarium. F. circinatum*, *F. fujikuroi*, *F. mangiferae*, *F. nygamai*, *F. proliferatum*, *F. sacchari*, *F. oxysporum* and *F. temperatum* are producers, while species such as *F. verticillioides*, *F. thapsinum* and *F. subglutinans* are non-producers. Wheat, rice, corn, barley and cereal products are the commodities most commonly contaminated with BEA. Beauvericin is a cyclic hexadepsipeptide that incorporates into biological membranes where it complexes with essential cations, increasing membrane permeability and altering cellular homeostasis. BEA is cytotoxic to some cell lines in vitro and potentially genotoxic to human lymphocytes, where it is associated with chromosomal aberrations. Genes encoding BEA biosynthesis were first described for *F. fujikuroi*. In this and other *Fusarium* species, including *F. mangiferae*, *F. proliferatum* and *F. oxysporum*, a cluster of four genes, including a non-ribosomal peptide synthase (NRPS22) and three accessory genes with transport and regulatory functions, are responsible for toxin biosynthesis. *Fusarium subglutinans* and *F. temperatum* are both maize pathogens that are closely related and indistinguishable based solely on morphology. BEA production distinguishes these species, however, as *F. temperatum* can produce BEA but *F. subglutinans* cannot. We recovered 25 *Fusarium* strains from maize harvested in Argentina. Based on partial EF-1α, β-tubulin and RPB2 sequences, 13 strains were identified as *F. subglutinans* and 12 as *F. temperatum*. BEA production was evaluated after growth in YES medium for 14 days at 25 °C. None of the 13 *F. subglutinans* strains produced BEA, while 9/12 of the *F. temperatum* strains produced BEA at between 7 and 400 μg/g. Genomic comparison of two species highlighted for the first time the presence in both species of BEA gene cluster, containing all four genes found in the cluster in *F. fujikuroi*. The *BEA1* gene (NRPS22), sequence in *F. temperatum* was intact and the same as that found in *F. fujikuroi* and the other known BEA-producing species of *Fusarium*. The *BEA1* gene of *F. subglutinans*, however, contained a unique 190 bp intron, absent in five BEA-producer species (Ff, Fp, Fm, Fo, Ft), and two SNPs that result in a truncated protein, based on “*in silico*” protein translation analysis. We think that these differences suffice to keep *F. subglutinans* from producing BEA.

This research was supported by the joint research project CNR-CONICET.

### 8.49. Development and Validation of an Hplc-Ms/Ms Method for the Determination of Aflatoxin Biomarkers in Chicken Excreta

Jurisic N. ^1,^*, Schwartz-Zimmermann H.E. ^1^, Kunz-Vekiru E. ^1^, Schatzmayr D. ^2^, Moll D. ^2^, Schweiger W. ^2^, Fowler J. ^3^ and Berthiller F. ^1^

^1^ Christian Doppler Laboratory for Mycotoxin Metabolismus and Center for Analytical Chemistry/Department of Agrobiotechnology (IFA-Tulln)/University of Natural Resources and Life Sciences/Vienna, Austria

^2^ BIOMIN Research Center/BIOMIN Holding GmbH/Tulln, Austria

^3^ Department of Poultry Science/University of Georgia/Athens, GA, USA

* Correspondence: nada.jurisic@boku.ac.at

**Abstract:** Aflatoxins are among the most potent natural carcinogens and as such the European Commission has enacted harsh maximum levels also for poultry feed (20 μg/kg), because depending on the species, poultry is considered relatively to very susceptible to the toxin. To establish reliable biomarkers for aflatoxin B_1_ (AfB_1_) in poultry we have conducted an experiment where chickens were fed with known concetrations of AfB_1_. Here we report on establishing and validation of biomarkers of exposure in chicken excreta. During a 21 day animal trial, 168 one-day-old chicks were divided into 24 pens and assigned to three different feed regimen: A) toxin-free diet; B) diet supplemented with 20 μg of AfB_1_/kg and C) supplemented with 500 μg of AfB_1_/kg. Chicken excreta were collected per pen after 7, 14, and 21 days. We developed and validated an analytical method for the determination of aflatoxin B_1_, B_2_, G_1_, G_2_, M_1_, P_1_, Q_1_ and the aflatoxin B_1_-*N*^7^-guanine adduct in freeze-dried chicken excreta. This method is based on sample extraction with acidified aqueous acetonitrile, followed by solid phase extraction and concurrent determination by liquid chromatography coupled to tandem mass spectrometry. Absorbed AfB_1_ and its metabolites are excreted in urine, while elimination through feces is a route for both unabsorbed AfB_1_ and biliary excreted metabolites formed from the absorbed toxin in chickens. Both should be reproducible in bird excreta. In freeze-dried samples collected from pens subjected to low concentrations of AfB_1_ we detected on average 1.6 ng/g AfB_1_, 0.1 ng/g AfB_2_, 0.6 ng/g AfG_1_ and 0.09 ng/g AfM_1_ whereas the aflatoxin B_1_-*N^7^*-guanine adduct was detected only in two samples collected after 21 days at concentrations of 0.6 ng/g and 0.2 ng/g. Moreover, average contents in all samples collected from birds with high AfB_1_ level in feed were 14.3 ng/g AfB_1_, 1.1 ng/g AfB_2_, 4.6 ng/g AfG_1_, 0.2 ng/g AfG_2_, 1.0 ng/g AfM_1_ and 0.7 ng/g of aflatoxin B_1_-*N*^7^-guanine adduct. Therefore, our results show that urinary AfM_1_, commonly used as biomarker of exposure in humans and pigs, and urinary aflatoxin B_1_-*N*^7^-guanine adduct which is a biomarker of aflatoxin exposure in humans can also be used as biomarkers in chickens.

### 8.50. Management Strategy of Contamination with Deoxynivalenol and Aflatoxins on Agrofood Chain in the Context of Climate Change Expected for Romania

Gagiu V. ^1,^*, Mateescu E. ^2^, Iorga E. ^1^ and Belc N. ^1^

^1^ National R&D Institute for Food Bioresources—IBA Bucharest, Bucharest, Romania

^2^ National Administration for Meteorology (METEO-Romania), Bucharest, Romania

* Correspondence: valeria.gagiu@bioresurse.ro

**Abstract:** Cereals contamination with mycotoxins under influence of the climate change expected at the European and Romanian level requires an integrated research that will represent basis for the implementation of management strategies on the agrofood chain in Romania. Differences in environmental conditions of the Romanian regions significantly influence the distribution of specific toxigenic fungi and mycotoxins, requiring monitoring and characterization of the contamination risk. Specialized scientific papers related on the climate change impact have estimated more obvious climate changes in the intramontane regions of Romania which will lead to quantitative and qualitative changes in cereal crops, as well as to the contamination pattern with fungi and mycotoxins. Regions with a potential risk of cereal contamination with deoxynivalenol coincide with the mining regions; as a result, heavy metals can migrate into the soil under heavy weather conditions and, together with mycotoxins, have an increased toxic effect, causing a higher incidence of cancer.

The general objectives of the project have an innovative approach, consisting of: (1). Correlation of the agro-climatic factors with quantitative (productivity) and qualitative (physico-chemical; microbiological—*Fusarium* sp., *Aspergillus* sp.; chemical—deoxynivalenol, aflatoxins and heavy metals) indicators of grain and food at regional and national level. (2). Identification and quantification of new chemical and biological risk factors in risk areas. (3). Integrated management of cereal and food contamination with fungi and mycotoxins in Romania. (4). Developing risk maps for grain and food contamination with deoxynivalenol and total aflatoxins. (5). Elaboration of a national management strategy for cereal and food contamination with fungi and mycotoxins in Romania.

Research activities combine: time scale (5 agricultural years), measurement scale (aprox. 3600 samples analysed by the ELISA method; temperature and precipitations) and geographic scale (6 agricultural regions). To analyze the quantitative and qualitative indicators of cereals and food under agroclimatic conditions from Romania, all data are organized in geo-referential databases, integrated in geographic information system and statistically analyzed based on the following criteria: interannual and interregional variations, geographical position, index of aridity, type of soil from the place of cereal cultivation. For detection of the trend for quantitative and qualitative indicators of cereals, developing of the risk maps and contamination scenarios, consortium uses all data which are available starting with 1 January 2011 until 31 August 2016.

The scientific results will help to develop a strategy for managing the effects of climate change on quantitative and qualitative grain indicators (with emphasis on grain contamination wih deoxynivalenol and aflatoxin) by the Competent Authorities of Romania.

### 8.51. Aspergillus carbonarius Proteases And Their Possible Activity in Ochratoxin a Degradation

Gallo A. ^1^, Ferrara M. ^2^, Bleve G. ^1^, Cervini C. ^2^, Magistà D. ^2^, Piemontese L. ^3^, Epifani F. ^2^, Logrieco A.F. ^2^, Solfrizzo M. ^2^ and Perrone G. ^2,^*

^1^ National Research Council, Institute of Sciences of Food Production, Lecce, Italy

^2^ National Research Council, Institute of Sciences of Food Production, Bari, Italy

^3^ Università degli Studi di Bari “Aldo Moro”, Dipartimento di Farmacia-Scienze del Farmaco, Bari, Italy

* Correspondence: giancarlo.perrone@ispa.cnr.it

**Abstract:**
*Aspergillus carbonarius* is the main responsible fungus of ochratoxin A (OTA) contamination of grapes and derived products. Recently, the biosynthetic mechanism of this mycotoxin has been mainly elucidated by experiments of knocking out of the key biosynthetic genes. The mutant strains of *A. carbonarius*, in which the *AcOTAnrps* gene had been disrupted, was unable to produce OTA but retained its ability to degrade OTA into OTα when it was grown in presence of exogenous OTA.

Microbial degradation of OTA is due to the enzymatic cleavage of the amide bond between L-β-phenylalanine and OTα by proteolytic proteins. Then, an in silico screening has been made on the available genome sequence of *A. carbonarius* ITEM 5010 to identify genes encoding proteases and to investigate their involvement in the OTA degrading activity of *A. carbonarius*. Preliminary transcriptomic analysis allowed selecting eight protease encoding genes that were expressed at increased level during OTA production. From the analysis of functional domains of the deduced protein sequences, four identified genes encode for aspartic proteases, three of them encode for serine proteases and one for a metalloprotease. Wild type and three mutant strains of *A. carbonarius* ITEM 5010 (*ΔAcOTAnrps*, *ΔAcOTApks*, *ΔAcOTAhal*) previously obtained and resulted to be unable to produce OTA, have been incubated in presence of OTA under different conditions and time of growth. Expression levels during growth and activation rate of the selected protease genes are under investigation in order to establish their involvement in the degradation activity of *A. carbonarius* strains.

The present work has received funding by the European Union’s Horizon2020 Research and innovation programme under Grant Agreement No. 678781 (MycoKey).

### 8.52. Potential Biological Control Agents from Strains Isolated from Gm and Non-Gm Brazilian Maize for Control of Aspergillus flavus and Aflatoxin B_1_ Production

Gasperini A.M. *, Medina A. and Magan N.

Applied Mycology Group, Environmental and AgriFood Department- SWEE, Cranfield University, Bedford, UK

* Correspondence: a.marcongasperini@cranfield.ac.uk

**Abstract:** Contamination of maize by *Aspergillus flavus* and the production of aflatoxins imposes an extensive socio-economic cost. Among the measures studied to reduce the risk of mycotoxins, biocontrol has been considered a promising technology for sustainable agriculture. A potential option for *A. flavus* management in the field has largely been focused on the use of atoxigenic isolates of *A. flavus* which may be able to compete with the toxigenic strains by displacing them and reducing aflatoxin B_1_ (AFB_1_) contamination. Thus, the aim of this work was to identify potential biological control agents isolated from distinct Brazilian GM/non-GM maize cultivars for control of AFB_1_. The interaction of toxigenic and atoxigenic *A. flavus* strains and other candidate species isolated from maize on relative competitiveness under different water availabilities (water activity (a_w_) 0.98 and 0.95) at 30 °C showed that *A. flavus* was largely dominant against other species tested and there was no inhibition of growth when paired with atoxigenic *A. flavus* strains. Control of AFB_1_ production using different initial inoculum ratios of 4 atoxigenic strains of *A. flavus* (AFL^−^) isolated from Brazilian GM/non-GM maize with 3 different toxigenic *A. flavus* strains was examined. The mixtures of spores of the strains were spread-plated on maize meal agars using a GM maize cultivar (P30F53F) and its respective non-GM isogenic (P30F53) cultivar as substrates. The AFB_1_ content was evaluated by HPLC-FLD after 7 and 14 days of incubation. All the AFL^−^ strains were able to reduce the AFB_1_ production in both 0.98 and 0.95 a_w_ treatments after 7 and 14 days at 30 °C. The ratios of 50:50 and 25:75 of AFL^+^: AFL^−^ were not statistically different at 0.95 a_w_ and the overall relative reduction of AFB_1_ at the end of the incubation period was 46–100%. There was no difference in the results obtained using GM and non-GM maize as a nutritional matrix in terms of AFB_1_ production or the relative amounts of control achieved. These results are a good method for effective screening of candidates for choosing the best strains for in vivo assays. Studies are in progress to examine in situ impacts on GM- and non-GM maize grain stored under different environmental conditions. Molecular techniques are also being employed to identify whether the atoxigenic *A. flavus* strains lack some key biosynthetic genes necessary for AFB_1_ production.

This research was supported by CAPES Foundation, Ministry of Education of Brazil—Project BEX 12937/13-4.

### 8.53. Temperature-Time Numerical Modelling of the Thermal Degradation of Aflatoxin B_1_ (Afb_1_) Using Response Surface Methodology

Gbashi S. ^1^, Madala N. ^2^, Adebo O.A. ^1^ and Njobeh P.B. ^1,^*

^1^ Department of Biotechnology and Food Technology, Faculty of Science, University of Johannesburg, P.O. Box 17011, Doornfontein Campus, 2028, Gauteng, South Africa

^2^ Department of Biochemistry, University of Johannesburg, P.O. Box 524, Auckland Park, 2006, South Africa

* Correspondence: pnjobeh@uj.ac.za

**Abstract:** The application of heat to biological systems is known to speed up chemical reactions and degrade bioactive components in proportion to the amount of heat energy supplied and time of exposure. Aflatoxin B_1_ (AFB_1_) is the most potent naturally occurring carcinogen known to man, however, studies on its thermal stability are limited and the available reports are conflicting. The present study systematically investigated the thermal degradation profile of AFB_1_ in pure form and when spiked into a food matrix (maize), as a function of temperature and exposure time using response surface methodology. Adopting the central composite design (CCD) approach, a set of statistically designed experiments were conducted and the model fitted to the experimental data. The resultant models were well fit (R^2^ = 0.97 and 0.92 for AFB_1_ standard and maize flour spiked with AFB_1_ respectively) and adequately described the thermal degradation kinectics of AFB_1_ as a second-order inverse reaction. The pareto plots showed significant (*p* < 0.05) interactive effects of temperature and exposure time on the thermal degradation of AFB_1_. Optimized conditions (i.e., minimum temperature and time required for complete degradation of AFB_1_) were 120 °C/5 min and 200 °C/60 min for pure AFB_1_ and AFB_1_ spiked into maize flour respectively, thus suggesting that matrix effect strongly affected the thermal stability of AFB_1_. Our observations herein could be critical for food safety applications targeted at reduction or complete elimination of AFB_1_ from food using heat processing while maintaining optimal food quality factors.

### 8.54. Development And In Vitro Efficacy Assessment of a New Multi-Mycotoxins Adsorbing Additive

Greco D. ^1^, D’Ascanio V. ^1^, Treglia A. ^1^, Santovito E. ^1^, Zanelli L. ^2^ and Avantaggiato G. ^1,^*

^1^ Istituto di Scienze delle Produzioni Alimentari (ISPA-CNR), Bari, Italy

^2^ Feed Industry Service (FIS) Srl, Lodi, Italy

* Correspondence: giuseppina.avantaggiato@ispa.cnr.it

**Abstract:** A variety of physical, chemical, and biological methods are proposed for the decontamination of mycotoxin-containing feedstuffs, but large-scale and practical methods are currently not available. The use of feed additives that reduce animal exposure to mycotoxins is regarded as an effective way to improve animal welfare. According to the European Regulation No. 386/2009, mycotoxin adsorbents are intended as nutritionally inert feed additives, which when incorporated into contaminated feeds diminish the absorption of mycotoxins from the gastrointestinal tract, thus preventing or reducing mycotoxicosis in livestock and poultry and carryover of mycotoxins into animal products. Various materials have been tested as mycotoxin adsorbents, and several studies have shown that these materials have high affinity for mycotoxins by the formation of stable linkages which can occur in several liquid systems. However, the main drawback in the use of these additives is that most mycotoxin adsorbents appears to bind to only a limited group of mycotoxins while showing very little or no binding to others. The aim of this work was the development of a new mineral-based material showing multi-mycotoxin adsorption efficacy. Twenty materials including sodium or calcium smectites from different sources, humic substances (humates), leonardite coals, organic polymers obtained from agricultural by-products were tested for their ability in binding simultaneously AFB_1_, ZEA, OTA, FB_1_ and DON from liquid buffers simulating gastric and intestinal pH values. Under restrictive experimental conditions, i.e., low adsorbent dosage (0.1% *w*/*v*) and high mycotoxin concentration (1 μg/mL), materials adsorbed the mycotoxins of interest with different extent depending on their source and medium pH. All of them did not bind DON. Three materials (an organic polymer and two Na-smectites) adsorbed significant amounts (>70%) of AFB_1_, ZEA, OTA and FB_1_ at different pH values. Thereof, a two-component mixture containing a sodium smectite and a bio-polymer was prepared and analyzed by equilibrium adsorption isotherms to determine the extension of the adsorption for each mycotoxin/material combination, the strength of the binding, and the equilibrium adsorption parameters (maximum adsorption capacity, adsorption affinity and heterogeneity of the adsorption mechanism). In addition, the effect of adsorbent dosage, pH, and gastrointestinal digestion were assessed. This is the first time that a smectite-based product was found effective in adsorbing in vitro several mycotoxins and in reducing the bioaccessible fraction of toxins in the chyme obtained after digestion of a multi-mycotoxins contaminated feed meal.

This research was supported by ISPA-CNR and FEED INDUSTRY SERVICE grant agreement.

### 8.55. A Bacterial Reductase Is Responsible for Fusarium Mycotoxin-Degrading Activity

He W.J. ^1,2^, Zhang L.M. ^3^, Yi S.Y. ^1,4^, Tang X.L. ^1,4^, Yuan X.S. ^1,4^, Guo M.W. ^1,4^, Bo Qu ^1,4^, Li H.P. ^1,2^ and Liao Y.C. ^1,4,5,^*

^1^ Molecular Biotechnology Laboratory of Triticeae Crops, Huazhong Agricultural University, Wuhan 430070, China

^2^ College of Life Science and Technology, Huazhong Agricultural University, Wuhan 430070, China

^3^ State Key Laboratory of Magnetic Resonance and Atomic and Molecular Physics, Wuhan Centre for Magnetic Resonance, Wuhan Institute of Physics and Mathematics, the Chinese Academy of Sciences, Wuhan 430071, China

^4^ College of Plant Science and Technology, Huazhong Agricultural University, Wuhan 430070, China

^5^ National Center of Plant Gene Research (Wuhan), Huazhong Agricultural University, Wuhan 430070, China

* Correspondence: yucailiao@mail.hzau.edu.cn

**Abstract:** Deoxynivalenol (DON) and its acetylated forms are the most abundant mycotoxins produced by *Fusarium* head blight pathogens, which infect the floret tissues of wheat, barley, maize and other small grain cereals in the field and colonize the developing grains. *Fusarium* toxins thus directly accumulate in the harvested grains, entering food/feed items such as flour and animal feed. DON has serious toxic effects on human and farm animals and is also phytotoxic, damaging plant tissues and acting as a virulence factor that stimulates fungal infection. Degradation of mycotoxins by microorganisms is a promising approach for detoxification of agricultural products. A soil bacterial strain that has the ability to degrade DON was isolated. Incubation of *Fusarium*-infected wheat grains with this bacterial strain completely eliminated DON. DON is catabolized by this strain into compounds with no detectable phytotoxicity, 3-oxo-DON and 3-epi-DON, via two sequential reactions. Comparative analysis of genome sequences from two DON-degrading strains, and one non-DON-degrading strain, combined with functional screening of a bacterial genomic BAC library led to the discovery that a novel reductase is responsible for oxidation of DON into 3-oxo-DON. DON-degrading activity is completely abolished in a mutant bacterial strain where the reductase gene is disrupted. Recombinant reductase protein expressed in *Escherichia coli* catalyzed the reversible oxidation/reduction of DON at a wide range of pH values and temperatures. The strain and recombinant reductase also catabolized zearalenone and the aldehydes glyoxal and methyglyoxal. This strain and the reducatse gene are promising agents for the control of *Fusarium* pathogens and detoxification of mycotoxins in plants and in food/feed products.

### 8.56. Detection of Nivalenol Synthesis Gene in Fusarium Species of Madder Seeds

Hosseini S.A.E. ^1^, Abedi-Tizaki M. ^2^, Ashkezary M.D. ^2^ and Fani S.R. ^1,^*

^1^ Plant Protection Research Dept., Yazd Agricultural and Natural Resources Research and Education Center, AREEO, Yazd, Iran

^2^ Department of Biology, Islamic Azad University, Ashkezar Branch, Ashkezar, Iran

* Correspondence: rezafani52@gmail.com

**Abstract:** Madder (*Rubia tinctorum* L.), an herbaceous perennial plant species is an economically important source of red pigment that used for medical and industrial applications and is widely cultivated in Yazd province, Iran. The madder roots contain different polyphenolic compounds and the source of red dyes known as rose madder. Different mycotoxigenic fungi are associated with seeds that affect quality of madder. *Fusarium* species are known to produce a wide range of mycotoxins kown as trichothecens such as nivalenol (NIV) in different growth stage of madder. The study was aimed to identify of nivalenol producer of *Fusarium* species isolates from seed of madder. Fifty six seed samples was collected from the main planting areas, including Bafgh and Ardakan during 2012–2014. Fungal isolates were isolated on potato dextrose agar (PDA) and carnation leaf agar (CLA) media. To extract genomic DNA mycelia were produced using potato dextrose broth (PDB). Total genomic DNA was extracted using CTAB method. Molecular identification of *Fusarium* species was performed using species-specific primers FEF1/2, SEM-1/2 and FpoF/R. The responsible gene for NIV production was detected using *Tri*13 primers. A high performance liquid chromatography (HPLC) used to confirm the ability the isolates to produce NIV. Overal, 249 fungal isolates were obtained from madder seed including *Fusarium*, *Aspergillus*, *Penicillium*, *Alternaria*, *Rhizoctonia* and *Rhizpous.* The highest frequency were belonged to the *Fusarium* isolates (*n* = 177). Among *Fusarium* species, *F*. *solani* (*n* = 55) and *F*. *oxysporum* (*n* = 41) were the most frequent species. The other species were included *F*. *equiseti* (*n* = 37), *F. semitectum* (*n* = 24) and *F*. *Poae* (*n* = 20). Molecular assays based on *Tri13* gene primers shown the ability to produce NIV in three recent *Fusarium* species with a visible 415 bp band. Analytical assays confirmed the potential ability of NIV production in the three *Fusarium* species The results of this study showed that fungi associated with madder seeds are able to produce trichothecene mycotoxins that they can be dangerous for consumers. This is the first report of fungi mycotoxins producing on seeds madder in Yazd province, therefore it should be consider more efforts to reduce the mycotoxin producer in seed of madder.

### 8.57. Use of Certified Reference Materials for the Determination of Mycotoxins

Irene Hahn *

Romer Labs Diagnostic GmbH, Tulln, Austria

* Correspondence: irene.hahn@romerlabs.com

**Abstract:** In the field of mycotoxin analysis, especially at low regulatory limits, different factors influence the quality of analytical data on contaminants in foods and animal feeds. Due to the heterogeneous distribution of mycotoxins in agricultural crops, sampling represents an important and crucial step and representative sampling is essential for the precision and accurate determination of mycotoxin levels. The use of proper and validated analytical methods, as well as the participation in proficiency testing and the use of accurately characterized reference materials further ensure quality assurance.

Several interlaboratory studies have demonstrated high between-laboratory standard deviations and non-traceable results. Consequently, standardization of appropriate analytical methods is needed. For gaining reliable and comparable quantitative data for mycotoxins, the use of suitable calibrants and certified reference materials (CRMs) of defined concentration and stated purity is required.

The regular use of CRMs improves the accuracy and thereby the comparability of analytical data during routine analysis (in terms of trueness, comparability and traceability). CRMs can be grouped in pure substances (standards), standard solutions (calibrants) and matrix materials (spiked or naturally contaminated). However, there is still a lack of accurately characterized reference materials that are commercially available. CRMs are characterized by a metrologically valid procedure for one or more specified properties, accompanied by a certificate that provides the value of the specified property, its associated uncertainty, and a statement of metrological traceability. Certain requirements for the competence of CRM producers have to be fulfilled according ISO 17034. Initially, the purity of the starting material has to be determined accurately (achieved by quantitative nuclear magnetic resonance). Subsequently, the assessment of the stability and homogeneity of the produced CRM is required. In addition to the certified value, the determination of the individual uncertainties (based on the preparation and stability of the calibrant in solution) as well as the calculation of the combined and expanded uncertainty is essential.

Within this talk, the differences between common reference materials and certified reference materials for the analysis of mycotoxins as well as the ensuing consequences will be discussed. In addition, the production and characterization of a certified reference material using the examples of deoxynivalenol, zearalenone, nivalenol and ochratoxin A in acetonitrile will be described. The technical requirements and challenges for the producer accredited according ISO 17034 will be mentioned. Furthermore, this presentation will highlight possible application of certified reference materials as well as the resulting benefits.

In summary, the proper use of CRMs is required to ensuring the quality of analytical data on contaminants in foods and animal feeds.

### 8.58. Qtl Mapping and Gwas Analysis for Fusarium Kernel Rot Resistance in the Magic Maize Population

Lanubile A. ^1,^*, Septiani P. ^2^, Busconi M. ^1^, Stagnati L. ^1^, Inzé D. ^3^, Morgante M. ^4^, Pè M.E. ^2^, Dell’Acqua M. ^2^ and Marocco A. ^1^

^1^ Department of Sustainable Crop Production, Università Cattolica del Sacro Cuore, Piacenza 29122, Italy

^2^ Institute of Life Sciences, Scuola Superiore Sant’Anna, Pisa 56127, Italy

^3^ Vlaams Instituutvoor Biotechnologie, Ghent B-9052, Belgium

^4^ Institute of Applied Genomics, Udine 33100, Italy

* Correspondence: alessandra.lanubile@unicatt.it

**Abstract:**
*Fusarium* ear rot (FER), caused by *Fusarium verticillioides*, is a major threat to maize yield and grain quality worldwide. Genomic loci responsible for natural disease resistance can be identified through quantitative trait loci (QTL) mapping that allows the development of resistant lines for the sustainable control of FER. A Multi-parent Advance Generation Intercross (MAGIC) population in maize was recently developed, providing a mean to conduct high-definition QTL mapping on small sets of highly diverse recombinant lines. We performed an in vitro assay for *Fusarium* kernel rot resistance using a rolled towel method in a set of 400 MAGIC maize recombinant inbred lines (RIL). For each RIL, 20 kernels as control and 20 kernels inoculated with *F. verticillioides* conidia were germinated for seven days. We measured infection severity level (SEV), seedling weight (PW), and length (PL). QTL analysis was performed on RIL whose haplotypes were reconstructed from 50K single-nucleotide polymorphism data. We identified 11 high confidence QTL for the considered traits. We found two QTL on chromosome 4 and one QTL on chromosome 5 for SEV, confirming that *Fusarium* kernel rot resistance is controlled by multiple loci with low effect. To guide the identification of candidate genes within the identified QTL, we exploited transcriptomic and sequencing information generated on the founder lines. We tested the differential expression of genes in the QTL confidence intervals matching the founder effects at the QTL as estimated by the mapping model. We identified 32 suggestive candidates for the three traits. Finally, we imputed the full genome sequence of the founder lines on the reconstructed RIL haplotypes to perform GWAS in the QTL regions and narrow down the confidence intervals. We conclude that the rolled towel assay applied on the MAGIC maize population is a fast and cost-effective method to identify QTL and candidate genes for disease resistance in maize.

### 8.59. Genomic Characterization of Trichoderma atrobrunneum (t. Harzianum Species Complex) Strain Item 908, a Biocontrol Agent of Fusarium Graminearum

Liuzzi V.C., Altomare C., Logrieco A., Fanelli F. * and Mulé G.

Institute of Sciences of Food Production, National Research Council, Italy

* Correspondence: francesca.fanelli@ispa.cnr.it

**Abstract:**
*Trichoderma atrobrunneum* F.B. Rocha, P. Chaverri& W. Jaklitsch strain ITEM 908 (formerly known as *T. harzianum* ITEM 908), is a biocontrol strain that is being registered under the European Union regulation as an active ingredient for the production of commercial biopesticides. The strain ITEM 908 proved to be able to inhibit completely the formation of peritheciaby*Fusarium graminearum* in dual cultures and to release in the agar medium metabolites that reduce the number of perithecia by over 70% (Altomare et al., poster presentation in this Congress). Therefore, ITEM 908 appears to be a good candidate biocontrol strain for prevention of Fusarium Head Blight (FHB) in the field by treatment of plant residues of the preceeding crop, thus reducing the primary inoculum. For the univocal characterization of this commercially valuable isolate and as a base for further studies aimed at elucidating its mechanisms of action and its physiological and molecular interactions with plants and target pathogens and pests, we sequenced the whole genome of the strain ITEM 908. The genome was sequenced on an Ion S5 platform generating around 7 M bpand assembled using the Spades v5.0 software. The resulting genome sequence has an estimated size of 39,131,654 bp. The reference gene sequences of ITS and TEF1 were extracted from the genome assembly. The identification of the strain ITEM 908 at species level was obtained by phylogenetic analysis with Maximum likelihood (ML) analysis performed with one-hundred ITS-TEF1manually concatenated datasetretrieved from sequences of *Trichoderma* spp. deposited in GenBank. The analysis placed ITEM 908 within the *T. atrobrunneum* group, close to *T. afroaharzianum* and *T. guizhouense*. Genome was annotated using the Augustus v3.1 software and 8649 genes were predicted. Approximately 3000 different *pfam* domains were detected and used to group genes within functional categories, including glycoside hydrolases, proteases and gene involved in stress tolerance and in secondary metabolites biosynthesis. Among these, 20 putative PKS, 8 putative NRPS and 5 putative PKS-NRPS were identified. Moreover, thesecretome of *T. atrobrunneum* ITEM 908, consisting of 761 proteins, was in silico predicted by the software SignalP (http://www.cbs.dtu.dk/services/SignalP/), that detects the presence of the secretion signal peptide at the N-terminus in amino acid sequences of a protein. The preliminary analysis of predicted genes highlights the potential ability of *T. atrobrunneum* ITEM 908 to produce a broad range of enzymes involved in the biocontrol activity.

This work was supported by H2020-MycoKey-(E.U.3.2-678781).

### 8.60. Systematic Assembly and Annotation of Wgs Data Facilitates Comparative Analysis of Secondary Metabolites Gene Clusters in Fusarium Species

Liuzzi V.C. ^1^, Fanelli F. ^1^, Chiara M. ^2^, Leslie J.F. ^3^, Logrieco A.F. and Mulè G. ^1,^*

^1^ Institute of Sciences of Food Production (ISPA), National Research Council, Bari, Italy

^2^ Dipartimento di Bioscienze, Università degli Studi di Milano, Milan, Italy

^3^ Department of Plant Pathology, Kansas State University, Manhattan, USA

* Correspondence: giuseppina.mule@ispa.cnr.it

**Abstract:** In the agro-food sector the management of NGS sequencing data have recently emerged due to the lack of coordinated scientific efforts for the development of guidelines and best practices for the storage and handling of such big-data. In this respect the case of toxigenic fungi like Fusarium spp, is emblematic and very fragmentary genomic sequencing data are available, often not annotated and deposited in redundant databases. This lack prevents the possibility to perform systematic comparative genomic studies, and limit the possibility to extract sensible information regarding the evolutionary history of these species. The study of secondary metabolites (SM) biosynthetic gene clusters is crucial for the understanding of the evolutionary mechanisms that drive species differentiation, pathogenicity, host specialization and to identify targets for development of novel strategies against these pathogens. It was demonstrated that some clusters evolve rapidly through multiple rearrangements, duplications, losses and horizontal gene transfer, the evolutionary mechanisms by which they are acquiredand maintained are not completely clear. The aim of this work was the development of guidelines and best practices for the assembly and annotation of WGS data derived from toxigenic and phytopatogenic fungi genomic projects. We have performed a comparative genomic analysis of different *Fusarium* species, focusing on secondary metabolites (SM) biosynthetic gene clusters. The workflow was structured as follow: retrieval of *Fusarium* genome sequencing data available from public NGS sequencing data archives; sequencing of new genomes of different species of *Fusarium* available in the AgroFood Microbial Culture Collection of the ISPA; assembling genomes using the Spades v5.0 software; annotation of genomes using the Augustus v3.1 software; retrieval of information concerning known/unknown-SM clusters; annotation of Pfam domains (http://pfam.xfam.org/) and prediction of the SM biosynthetic gene clusters using the collected SM clusters as models; calculation of clusters of orthologous genes and gene families; studying the presence/absence, composition, order, orientation of each gene within clusters and the distribution of clusters among the isolates; construction of “pancluster” phylogenetic trees based on the distance matrices; collecting all the data obtained in a *Fusarium*SM cluster database that can be interrogated by specific softwares. Preliminary data show that phylogenies and SM cluster distribution among the isolates included in the current study are coherent with published data and recapitulate the discontinuous distribution of SM cluster and thus in the genetic potential of species to produce secondary metabolites. Detailed analysis of individual clustersenabled the identification and the study of different mechanism of inheritance of SM cluster, thus showing the advantages of systematic strategies for the analysis and annotation of these genomes.

This work was supported by H2020-MycoKey-(E.U.3.2-678781).

### 8.61. In Vitro Effects of Essential Oils on the Mycelial Growth and Mycotoxin Production by Aspergillus parasiticus

Lorán S. *, Bervis N., Herrera M., Rota C. and Ariño A.

Instituto Agroalimentario de Aragón IA2 (Universidad de Zaragoza—CITA), Veterinary Faculty, 50013, Zaragoza, Spain

* Correspondence: sloran@unizar.es

**Abstract:** Aflatoxins are a group of mycotoxins produced mainly by two species of the genus *Aspergillus*, *A. flavus* and *A. parasiticus*. These toxins may be found in different agricultural products which represent a significant risk that concerns to food safety. In fact, due to their toxicological effects in humans and animals and the increasing occurrence in European countries they are receiving great attention. Therefore, different strategies both at pre-harvest and post-harvest stages are being applied to reduce the entry of aflatoxins to the food and feed chains.

In this work, we have evaluated the ‘in vitro’ effect of essential oils (EOs) (*Origanum virens*, lavandin Abrial and lavandin Grosso), chemically characterized and obtained from controlled plantations, on the growth and aflatoxin production by reference strain *Aspergillus parasiticus* CECT 2682.

Minimal inhibitory concentration (MIC) and minimal fungicide concentration (MFC) were determined by the tube dilution method in flasks containing 10 mL of sterile Yeast Extract Sucrose (YES) broth added with the EOs at different concentrations (0.2; 0.4; 0.6; 0.8; 1; 3 and 5 μL/mL) and inoculated with 100 μL of a 10^6^ spores/mL suspension. After incubation at 25 °C for 10 days the weight of the mycelia was determined and compared with the control (YES broth inoculated with *A. parasiticus* without EO). The antimycotoxigenic activity was evaluated as well in the two concentrations of the EO right before the MIC value. In so doing, the culture media were extracted with methanol:water (80:20), filtered and purified with immunoaffinity columns. Finally, aflatoxins were determined by HPLC-FLD-PHRED.

The results showed variations in the antifungal and antimycotoxigenic properties of the EOs evaluated. The most effective one was *Origanum virens*, with MIC and MFC values of 0.6 μL/mL. Meanwhile, EOs from lavandin showed a similar activity: MIC values of 3 μL/mL and MFC of 3 μL/mL (lavandin Grosso) and 5 μL/mL (lavandin Abrial). In most cases the EO concentrations right before the MIC value strongly reduced the synthesis of aflatoxins, except for lavandin Abrial. It may be concluded that tested EOs, particularly *O. virens*, may constitute an alternative to synthetic chemical agents against molds and mycotoxins which should be further studied.

**Acknowledgments:** Projects JIUZ-2014-CIE-05 (University of Zaragoza); Grupo Consolidado A01 (Gobierno de Aragón-FEDER); AGL2014-57069-R (MINECO-FEDER). N. Bervis thanks the grant FPU13/04238. To Jesús Burillo (CITA), who kindly supplied the essential oils.

### 8.62. MycoKey—A Project in a Global Context: Concerns and Challenges for an Integrated and Innovative Approach to Ensure Food Safety Throughout the Food and Feed Chain

Logrieco A.F. ^1,^*, Feng J. ^2^, Hao Z. ^2^, Battilani P. ^3^, De Saeger S. ^4^, Waalwijk C. ^5^, Vanderlee T. ^5^, Vogelgsang S. ^6^, Laitila A. ^7^, Pascale M. ^1^, Moretti A. ^1^, Cito N.M. ^1^ and Avantaggiato G. ^1^

^1^ Institute of Sciences of Food Production, National Research Council, Italy

^2^ Institute of Plant Protection, Chinese Academy of Agricultural Sciences, China

^3^ Department of Sustainable Crop Production, Università Cattolica del Sacro Cuore, Piacenza, Italy

^4^ Laboratory of Food Analysis/Faculty of Pharmaceutical Sciences, Ghent University, Belgium

^5^ Plant Research International, Droevendaalsesteeg 1, 6708 PB Wageningen, The Netherlands

^6^ Research Group Ecology of Noxious and Beneficial Organisms, Research Division Plant Protection, Agroscope, Zurich, Switzerland

^7^ VTT Technical Research Centre of Finland, Espoo, Finland

* Correspondence: antonio.logrieco@ispa.cnr.it

**Abstract:** MycoKey (http://www.mycokey.eu) aims to generate innovative and integrated key solutions that will support stakeholders to achieve efficient and sustainable mycotoxin management strategies along the food and feed chain. The project utilises a multi-disciplinary approach by involving a variety of different research skills and by openly sharing innovative tools. Furthermore, research outputs such as scientific datasets will be available by open access publications. This approach is needed to tackle the complex problem of mycotoxin menace, both on the European and the global level, and to communicate practical and viable solutions to the food and feed chain players. Toxigenic fungi attack several crops throughout large geographic areas, with different implications and effects on food and feed production, food consumption and health. The problems caused by toxigenic fungi are of high importance in Europe, which is reflected by several interventions that have been supported by the EU Commission in the past 20 years. Furthermore, mycotoxin contaminations appear also severe in countries like China, which has a growing food and feed production, an increasing share of contaminated batches and a significant international trade of commodities. MycoKey addresses the main affected crops—maize, wheat and barley (with less extent dried fruits and grapes)—with their associated toxigenic fungi and related mycotoxins (aflatoxins, deoxynivalenol and other trichothecenes, zearalenone, ochratoxin A and fumonisins) in both Europe and China, thanks to a strong interaction between 32 European and 11 Chinese partners. The project foresees the generation of innovative solutions by integrating key information for mycotoxin management into an ICT tool (MycoKey App) for smart farming systems. It will provide stakeholders with rapid and customised information on contamination risk/levels, decision support and practical economically sound suggestions for intervention where needed. Other methodologies, strategies and tools are being developed for prevention and monitoring as well as for cost-effective interventions in the field, during storage, processing, and transportation. Remediation strategies for alternative and safe ways to use contaminated batches will be investigated as well. All actions conducted by MycoKey will contribute to processes and communication that will ultimately result in an improved mycotoxin management, while providing input for legislation, as well as for enhanced knowledge and strengthened, interdisciplinary networks.

### 8.63. Reduction In Vitro of the Ochratoxin a Using the Digestive Enzyme Pancreatin

Luz C. ^1^, Ferrer J. ^1^, Luciano F.B. ^2^, Mañes J. ^1^ and Meca G. ^1,^*

^1^ Laboratorio de Química de los Alimentos y Toxicología de la Facultat de Farmàcia, Universitat de València. Av. Vicent Andrés Estellés s/n, 46100 Burjassot, España

^2^ Departamento de ciência animal, Escola de Ciências da Vida, Pontificia Universidade Católica do Paraná. Rua Imaculada Conceição 1155, 80901-215 Curitiba, Paraná, Brasil

* Correspondence: giuseppe.meca@uv.es

**Abstract:** Ochratoxin A (OTA) is a mycotoxin produced by the metabolism of fungus belonging to the genus *Aspergillus* and *Penicillium*. It is classified by the International Agency for Research on Cancer (IARC) as possibly carcinogenic to humans (group 2B) and it has been proven to be nephrotoxic and hepatotoxic, amongst other toxic properties. Human exposure to OTA occurs mostly by dietary intake, since it’s a common contaminant in many foodstuffs such as cereal and coffee grains, grapes, etc. In the same way, the possibility that farming animals may result intoxicated by exposure to contaminated agricultural products and fodder represents a risk for public health.

Pancreatin consists of enzymes from cow or pig pancreas, namely amylase, protease, and lipase, which digest starch, protein, and lipids, respectively. Pancreatic enzymes are used in modern medicine mainly for treating exocrine pancreatic insufficiency, a condition in which food is not properly digested because the pancreas does not make an adequate amount of digestive enzymes. Other potential uses, which have less supportive evidence, include immune stimulation, tissue repair, blood clot treatment, and as a general digestive aid.

In the present study, the capacity of pancreatin to reduce the OTA present in PBS medium 0.2 mM pH 3.5 y 7 was evaluated. The bioaccessibility of OTA was also determined after simulated digestion in the stomach, duodenum and colon compartments, using different concentrations of pancreatin. The determination and quantification of OTA was performed by liquid chromatography coupled to a fluorescence detector. The concentration of pancreatin and OTA used were 100–1000 IU protease/mL and 250–1000 ppb respectively. The results obtained showed that the OTA concentration in the PBS mediums suffered a total reduction up to 98–100% at pH 7, whereas at pH 3.5 was not observed any reduction of the bioactive compound studied. The bioaccessibility of OTA was significantly reduced in the colon compartment, resulting in a bioaccessibility reduction variable from 94 to 99%, whereas in the small intestine compartment the OTA bioaccessibility was not significantly reduced.

### 8.64. Aflatoxin Risk Management in Commercial Groundnut Products in Malawi (Sub-Saharan Africa): A Call for a More Socially Responsible Industry

Magamba K. ^1^, Matumba L. ^1,^*, Matita G. ^2^, Gama A.P. ^3^, Singano L. ^4^, Monjerezi M. ^5^ and Njoroge S.M.C. ^6^

^1^ Food Technology and Nutrition Group, Lilongwe University of Agriculture and Natural Resources (LUANAR),NRC campus, P.O. Box 143, Lilongwe, Malawi

^2^ Veterinary Sciences, LUANAR (NRC campus), P.O. Box 143, Lilongwe, Malawi

^3^ Food Science and Technology Department’ LUANAR (Bunda campus), P.O. Box 219, Lilongwe

^4^ Department of Agricultural Research Services, Chitedze Research Station, P.O. Box 158, Lilongwe, Malawi

^5^ Department of Chemistry, Chancellor College, University of Malawi, P.O. Box 280, Zomba, Malawi

^6^ International Crops Research Institute for the Semi-Arid Tropics (ICRISAT), P.O. Box 1096, Lilongwe, Malawi

* Correspondence: alimbikani@gmail.com

**Abstract:** To elucidate the impact of aflatoxin management interventions, we conducted a follow-up study, to determine the incidence of aflatoxin contamination in 67 samples of raw groundnut, groundnut flour, and peanut butter which were purchased from vendors and supermarkets in Lilongwe, Malawi. Aflatoxin was estimated by a fluorometric method using affinity columns and was detected in all samples. Total aflatoxin levels ranged from 1.5 to 1200 μg/kg in raw groundnuts and 83 to 820 μg/kg in groundnut flour from vendors. In branded groundnut flour and peanut butter from supermarkets, aflatoxin levels ranged from 13 to 670 μg/kg and 1.3 to 180 μg/kg, respectively. About 93, 88, 78 and 72% of samples analyzed contained aflatoxin levels above regulatory limit used in Malawi (3 μg/kg), EU (4 μg/kg), most developing countries (10 μg/kg), and the USA (20 μg/kg), respectively. Considering the difficulty of achieving an efficient government regulation system in the sub-Saharan region due to resource constraints, future efforts should seek a holistic approach to tackling the aflatoxin problem. In this regard, we recommend the promotion of a socially responsible groundnut processing industry that has consumer welfare as its central feature. In addition, consumer education, especially on health hazards associated with aflatoxins and visual identification of potentially contaminated groundnuts, should be intensified.

**Keywords:** aflatoxin; commercial groundnut products; Malawi; risk management

### 8.65. Efficacy of Electrolyzed Oxidizing Water (Eow) on Grape Berries for Inactivating of Aspergillus carbonarius Growth and Ochratoxin a Production

Magistà D., Cozzi G., Gambacorta L., Solfrizzo M., Logrieco A.F. and Perrone G. *

National Research Council, Institute of Sciences of Food Production, Bari, Italy

* Correspondence: giancarlo.perrone@ispa.cnr.it

**Abstract:** Contamination of vineyards from black Aspergilli is a well-known condition that cause the accumulation of ochratoxin A (OTA) in grapes and derived products. This contamination is strongly related to climatic conditions, geographical regions (South Mediterranean climate is highly conducive), grape varieties, damage by insects, although, great variations may occur from one year to another. Among the black Aspergilli commonly found in infected grapes, *Aspergillus carbonarius* is considered the main responsible of OTA contamination, with *A. niger* at secondary extent. To minimise the black Aspergilli infection and limit OTA concentrations in grapes, several strategies are commonly adopted, including the implementations of good agricultural practices and the use of pesticides and fungicides. These strategies are essential to manage the problem, but since they are insufficient when extremely favourable condition occurs in the vineyard, new strategies, aimed to reduce OTA risk in vineyards, are necessary. In this respect, implementation of electrolysed oxidising water (EOW) in agriculture has arising during the last decade as an interesting alternative to replace or limit the use of chemicals. The efficacy of EOW was also demonstrated in post-harvest for reduction of gray mold and brown rot on surfaces of peaches and grapes.

In this study, we screened for the first time the efficacy of EOW generated by EVA System^®^ 100 apparatus (Industrie De Nora S.p.A., Milan, Italy) at different concentrations of free chlorine (ranging from 0.0125 to 0.4 g/L) on conidial germination and growth of *A. carbonarius* and *A. niger*. A good fungicidal activity was achieved after 2–10 min treatment with EOW containing 0.4–0.2 g/L of free chlorine, although *A. carbonarius* conidia were more resistant to EOW than *A. niger* conidia. Then EOW at 0.4 g/L free chlorine was tested on detached berries of Primitivo and Aglianico wine grape varieties that were singularly infected with *A. niger* and *A. carbonarius*. Treatments with Switch (cyprodinil and fludioxonil) at 0.8 g/L, a fungicide regularly used in vineyard against black Aspergilli, were used as positive controls. Percentage of infected berries, McKinney index, and OTA concentrations were used to evaluate the efficacy of the EOW treatment. On Aglianico grape berries EOW and Switch produced an almost complete reduction on percentage of infection, McKinney index and OTA concentration compared to the control. On Primitivo grape berries EOW treatment reduced more than half the percentage of infections and McKinney index for both *A. carbonarius* and *A. niger*, although Switch showed a better performance. A significant reduction of OTA concentration was observed for EOW and Switch treatments. These results evidence for the first time that EOW is effective to reduce black Aspergilli inoculum and OTA contamination on grape berries.

The present work has received funding by the European Union’s Horizon2020 Research and innovation programme under Grant Agreement No. 678781 (MycoKey).

### 8.66. A Mechanistic Model for Prediction of the Risk Posed by Fusarium Graminearum Ascospores to Wheat

Manstretta V. ^1,^*, Gourdain E. ^2^ and Rossi V. ^3^

^1^ Horta S.R.L., via E. Gorra 55, 29122 Piacenza, Italy

^2^ ARVALIS—Institut du végétal, Station Expérimentale, 91720 Boigneville, France

^3^ Di. Pro. Ve. S., Department of Sustainable Crop Production, Università Cattolica del Sacro Cuore, via Emilia Parmense 84, 29122 Piacenza, Italy

* Correspondence: v.manstretta@horta-srl.com

**Abstract:** A model to estimate the risk posed by *Fusarium graminearum* ascospores to wheat was developed following a mechanistic approach. The model steps leading to ascospores infection on heads included into the model are: (i) maturation of perithecia and ascospores; (ii) ascospore discharge; (iii) ascospore survival after discharge; and (iv) ascospore germination. The first step considers that perithecia, the fruiting bodies containing ascospores, are produced on previous crop debris when suitable weather conditions occur; the proportion of the mature perithecia is then calculated as a function of temperature and moisture of the crop debris, the latter being calculated, in turn, based on weather conditions. Ascospores are discharged from perithecia when rain or appropriate vapour pressure deficit conditions occur; the proportion of the discharged ascospores is calculated as function of temperature, relative humidity and time. After discharge, ascospores can experience unfavorable weather conditions and loose viability; the proportion of ascospores surviving under non-optimal conditions is calculated as function of temperature and relative humidity. Finally, germination of viable ascospores is calculated by mean of equations based on temperature and time at high relative humidity.

The model was validated by using independent field data collected in France and Italy between 1999 and 2009; 39 wheat crops having a cereal as pre-crop, untreated against Fusarium Head Blight, and with no, minimum or non-turning soil tillage were considered for validation purposes. For each crop, the model was run and model outputs (i.e., ascospore germination values) were accumulated for a period of four weeks around wheat flowering. Accumulated model outputs were then compared with the amount of deoxynivalenol (DON) accumulated in kernels at harvest. A significant correlation was found between model output and DON content in kernels (Pearson’s correlation coefficient *r* = 0.92 and *r* = 074 for French and Italian data, respectively) indicating that the model provided accurate prediction of the risk for DON accumulation in wheat kernels.

### 8.67. Mandela Cock *Versus* Windrow Groundnut Drying Technique: A Paired Comparison of Aflatoxin Contamination and Seed Germination

Matumba L. ^1,^*, Singano L. ^2^, Tran B. ^3^, Mukanga M. ^4^, Makwenda B. ^5^, Kumwenda W. ^5^, Mgwira S. ^6^, Phiri S. ^5^, Mataya F. ^5^, Mthunzi T. ^7^, Alfred S. ^7^, Madzivhandila T. ^7^, Mugabe J. ^8^ and Chancellor T. ^3^

^1^ Lilongwe University of Agriculture and Natural Resources (NRC Campus), Lilongwe, Malawi

^2^ Department of Agricultural Research Services, Chitedze Research Station, Lilongwe, Malawi

^3^ Natural Resources Institute-University of Greenwich, Chatham, Maritime Kent, UK

^4^ Zambia Agriculture Research Institute (ZARI), Lusaka, Zambia

^5^ The National Smallholder Farmers' Association of Malawi (NASFAM), Lilongwe, Malawi

^6^ Lilongwe University of Agriculture and Natural Resources (Bunda Campus), Lilongwe, Malawi

^7^ Food, Agriculture and Natural Resources Policy Analysis Network, Pretoria, South Africa

^8^ Forum for Agricultural Research in Africa (FARA), Accra, Ghana

* Correspondence: alimbikani@gmail.com

**Abstract:** Prompt moisture content reduction in harvested groundnuts is critical for safe storage. In most parts of sub-Saharan Africa, moisture content reduction is practically achieved by natural solar drying. In particular, the groundnuts are traditionally cured in the field using inverted windrow drying methodology. However, recently, Mandela cock technique, a ventilated stack of groundnut plants with a chimney at the center has been introduced in the southern Africa region and is believed to reduce the risk of aflatoxin contamination and maintain seed viability. A study involving 29 farmers across 3 districts in Malawi was carried out in 2016 to systematically compare the performance of the two drying techniques with respect to aflatoxin control and seed quality. Paired *t*-test results indicate that Mandela cock groundnut drying technique significantly (*p* < 0.10) increased the risk of aflatoxin contamination and lowered seed percentage germination compared to the traditional inverted windrow drying. Considering that the Mandela cock method was introduced in the region without conducting efficacy trials the present findings clearly demonstrate the need for strict government regulation and technology validation if farmers are to benefit.

### 8.68. A Survey of Citrinin and Ochratoxin a in Food and Feed on the Belgian Market

Meerpoel C. ^1,2^^,^*, di Mavungu J.D. ^1^, Huybrechts B. ^3^, Tangni E. ^3^, Devreese M. ^2^, Croubels S. ^2^ and De Saeger S. ^1^

^1^ Department of Bioanalysis, Laboratory of Food Analysis, Faculty of Pharmaceutical Sciences, Ghent University, Ghent, Belgium

^2^ Department of Pharmacology, Toxicology and Biochemistry, Faculty of Veterinary Medicine, Ghent University, Salisburylaan 133, 9820 Merelbeke, Belgium

^3^ Veterinary and Agrochemical Research Center (CODA-CERVA), Tervuren, Belgium

* Correspondence: celine.meerpoel@ugent.be

**Abstract:** Mycotoxins are important contaminants in the food chain and can cause serious toxic effects. In 2012, the European Food Safety Authority (EFSA) published a scientific opinion on citrinin (CIT) whereby the need for additional quantitative occurrence and toxicity data was emphasized in order to perform a risk assessment regarding CIT in food and feed. Since citrinin often co-occurs with ochratoxin A (OTA), it is interesting to investigate the presence of both mycotoxins in food and feed. The aim of this study is therefore to gather information on the (co-)occurrence of CIT and OTA in feed and different foodstuffs available on the Belgian market with the prospect of identifying all relevant sources of intake and their importance.

In a first stage, a UPLC-ESI^+/−^-MS/MS method for the simultaneous analysis of CIT and OTA in feed and several cereal-based food products was developed. The mycotoxins were extracted from these matrices using a QuEChERS-based extraction method (Quick, Easy, Cheap, Effective, Rugged and Safe) without any further clean-up step. Final extracts were analyzed using a Waters Acquity UPLC system coupled to a Xevo TQ-S mass spectrometer equipped with an electrospray interface operated in both positive and negative ionization mode. Chromatographic separation was achieved using an Acquity UPLC HSS T3 column by applying gradient elution and the total run time was 10 min. This method was validated for several parameters such as specificity, linearity, apparent recovery, limit of detection, limit of quantification, precision and measurement uncertainty following the criteria mentioned in Commission Regulation No. 401/2006/EC and Commission Decision No. 2002/657/EC.

Further, the method will be applied for the analysis of 400 food (cereal products, fruit and vegetable juices, herbs and spices, nuts and seeds, alcoholic beverages, baby food, soy and vegetarian products, food supplements and meat products) and 100 feed samples that will be collected from the Belgian market over a period of 2 years. The results of the first set of collected samples will be presented.

All collected data and results of the chemical analyses will be brought together in a databank in order to perform a risk assessment in Belgium (exposure assessment and risk characterization for both Belgian population and pig and poultry sector).

This research was financially supported by the Federal Public Service Health, Food Chain Safety and Environment (Project RT 16/6308).

### 8.69. Subcritical Water Extraction as an Alternative to Extract Trichothecenes from Cereal Matrices

Miró-Abella E. ^1,2,^*, Herrero P. ^2^, Canela N. ^2^, Arola L. ^3^, Ras R. ^2^, Fontanals N. ^1^ and Borrull F. ^1^

^1^ Department of Analytical Chemistry and Organic Chemistry, Universitat Rovira i Virgili, Tarragona, Spain

^2^ Centre for Omic Sciences (COS), Universitat Rovira i Virgili, Reus, Spain

^3^ Unitat de biotecnologia—EURECAT, Reus, Spain

* Correspondence: eugenia.miro@fundacio.urv.cat

**Abstract:** In recent times, the consumption of cereals like spelt, oat and quinoa, has grown since the augmented interest in eating habits. The fact of increase the consumption of cereals, leads at the same time to an augment in the potential ingestion of mycotoxins ^1,2^. For that, friendly effective routine analysis must be carried out in order to quantify and prevent their presence in cereals. Often, the developed methods are unable to extract and detect the presence of masked mycotoxins, especially trichothecene derivatives. For that, a subcritical water extraction (SWE) followed by solid-phase extraction clean-up and ultra-high performance liquid chromatography coupled with tandem mass spectrometry detection is developed and reported for the first time, for the determination of 6 trichothecenes from different cereals. Deoxynivalenol, deoxynivalenol-3-glucoside, 3-acetyl-deoxynivalenol, 15-acetyl-deoxynivalenol, HT-2 toxin and T-2 toxin were extracted from three different cereals (spelt, millet and oat) and two pseudocereals (quinoa and sesame seeds) using acidified water (1% formic acid) as the extraction solvent. This SWE, followed by the straight-forward clean-up step, achieved good performance with acceptable matrix effects (from 15% to −45%), and method quantification limits between 0.4 μg kg^−1^ (for T-2 toxin) and 20 μg kg^−1^ (for deoxynivalenol-3-glucoside). The present method enabled a selective extraction while low matrix effect levels, which involves the quantification of the target analytes at very low concentrations and the possibility to apply it for the selective detection of the natural presence of trichothecenes. Thus, it was possible to detect at least one trichothecene in millet and in oat studied samples. The performance of the method may indicate a benefit of using alternative solvents, such as water, able to obtain results as sensitive and reliable as those provided by organic solvents.
Berti, C.; Riso, P.; Brusamolino, A.; Porrini, M. Effect on appetite control of minor cereal and pseudocereal products. *Br. J. Nutr.*
**2005**, *94*, 850–858.Miró-Abella, E.; Herrero, P.; Canela, N.; Arola, L.; Borrull, F.; Ras, R.; Fontanals, N. Determination of mycotoxins in plant-based beverages using QuEChERS and liquid chromatography-tandem mass spectrometry. *Food Chem.*
**2017**, *229*, 366–372.

### 8.70. Fungal and Mycotoxins Contamination of Poultry Feeds from Feed Manufacturer in Selected Provinces of South Africa

Mokubedi S.M. ^1,3,^*, Njobeh P.B. ^1,3^ and Phoku J.Z. ^2,3^

^1^ Department of Biotechnology and Food Technology, University of Johannesburg, Johannesburg, South Africa

^2^ Department of Biomedical Technology, University of Johannesburg, Johannesburg, South Africa

^3^ Food, Environment and Health Research Group, University of Johannesburg, Johannesburg, South Africa

* Correspondence: sharonmaphala@gmail.com

**Abstract:** In total, 110 samples of poultry feed from a manufacturer in five selected provinces of South Africa were screened for fungal species (spp.) by means of a serial dilution technique followed by DNA sequencing. The data revealed samples contaminated with *Aspergillus* spp. being the most dominant species followed by *Fusarium* spp. then *Penicillium* spp. Furthermore, the presence of some mycotoxins in the samples were evaluated using high performance liquid chromatography (HPLC) and liquid chromatography-mass spectrometry (LC/MS). The contamination level of zearalenone (ZEA) was the highest with a maximum value of 1209.00 μg/kg followed by fumonisins (FB) (957.51 μg/kg), deoxynivalenol (DON) (195.46 μg/kg), and ochratoxin A (OTA) (43.91 μg/kg). The maximum values of aflatoxin G_1_, G_2_, B_1_, and B_2_ (40.93 μg/kg, 14.25 μg/kg, 3.95 μg/kg, 0.55 μg/kg, respectively) were the lowest with the samples contaminated with at least two of the aflatoxins. As poultry and poultry products are the most affordable protein source, persistent co-occurrences of these contaminants in poultry feeds remains a threat to poultry health and the economy of the industry. Thus, more strategies for feed security and regulatory standards are required ceaseless affiliated with changing weather conditions.

**Keywords**: mycotoxins; fungi; poultry feed; health; South Africa

### 8.71. Improved Procedure for Assessment of Competitiveness of Non-Toxigenic Isolates of Aspergillus flavus to Mitigate Production of Aflatoxins by Highly Toxic Isolates

Moradi M. ^1,^*, Fani S.R. ^2^ and Madani M. ^3^

^1^ Pistachio Research Center, Horticultural Sciences Research Institute, Agricultural Research, Education and Extension Organization (AREEO), Rafsanjan, Iran

^2^ Plant Protection Research Department, Yazd Agricultural and Natural Resources Research and Education Center, AREEO, Yazd, Iran

^3^ Former research associate, Soil Science Department, Faculty of Agriculture, University of Manitoba, Winnipeg, Canada

* Correspondence: moradi@pri.ir

**Abstract:** Aflatoxins are carcinogenic secondary metabolites produced by some species of *Aspergillus* on different crops with potential of causing disease and death in animals and humans. Non-toxigenic strains of *Aspergillus flavus* have been successfully used to reduce aflatoxins in some agricultural crops through competition with toxigenic populations. Although analytical and immunoassay methods for screening of non-toxigenic isolates are accurate and reliable, but require expensive laboratory equipment and supplies. We developed qualitative cultural methods with more efficient results in term of cost and time as an alternative to screen competitive ability a large volume of non-toxigenic isolates to interfere with aflatoxin production by highly toxigenic isolates. Here, in this study, cultural methods based on fluorescence detection (FD) and ammonia vapor (AV) were used to analyses 270 *A. flavus* isolates from different agro ecological zone collected in Kerman province, Iran. Results showed that 17 isolates were non-toxigenic which further were confirmed by Thin Layer Chromatography (TLC). Identification of non-toxigenic *A. flavus* isolates was performed using both diagnostic PCR with species-specific primers (FLAVIQ1/FLAVIQ2) and morphological analysis. To evaluate competitive ability among non-toxigenic isolates of *A. flavus*, with highly toxigenic isolate, rice flour, coconut agar and coconut broth medium substrates were inoculated with the same conidial concentrations of both strains (4000/for each strain), simultaneously. Rice flour substrate was used to quantify the content of aflatoxin in media with co-inoculations and toxigenic isolate using method of thin layer chromatography plates and scanning densitometer. Culture media were subjected to intensity measurement of color change on exposing to ammonium hydroxide vapor. The reduction rates of aflatoxin B_1_ in co-inoculations were varied and ranged from 2% to 82%. Based on the intensity of colony color changes, the competitive effects of the isolates were classified in five groups. Non-toxigenic isolates with high competitiveness have shown low color changes in culture media and high aflatoxin reduction in TLC assays with a ratio of more than 78%. The method has been successfully applied to access the competitiveness of yeast isolates to interfere with aflatoxin production by highly toxigenic strain. Presented method will improve efficiency of preliminary screening of non-toxigenic isolates used to mitigate of aflatoxin production in animal and human food and feed as a low cost, simple and quick method.

### 8.72. Determination of Fungal Contamination in Food Spices: A Case Study of One Main Distributor in South Africa

Motloung L. ^1,^*, Njobeh P.B. ^1^, De Boevre M. ^2^, Audenaert K. ^3^, Phoku J.Z. ^4^ and De Saege, S. ^2^

^1^ Department of Biotechnology and Food Technology, Faculty of Science, University of Johannesburg, P.O. Box 17011, Doornfontein Campus, Johannesburg, South Africa

^2^ Laboratory of Food Analysis, Faculty of Pharmaceutical Sciences, Ghent University, Ottergemsesteenweg 460, 9000 Ghent, Belgium

^3^ Faculty of Bioscience Engineering Ghent University, Valentin Vaerwyckweg, 1, BE-9000 Ghent, Belgium

^4^ Department of Biomedical Technology, Faculty of Health Sciences, University of Johannesburg, P.O. Box 17011, Doornfontein Campus, Johannesburg, South Africa

* Correspondence: lmotloung91@gmail.com

**Abstract:** Foods such as spices and herbs, which are used throughout food processing are often predisposed to toxigenic fungi. Exposure to these contaminants presents a health hazard to humans, thus, it is imperative to monitor highly-consumed spices for fungal growth with subsequent mycotoxin analysis. A total of 70 samples (5.0 g each) of dried food spices [coarse chilli (*n* = 14), ground chilli (*n* = 4), paprika (*n* = 7), ginger (*n* = 5), chicken (*n* = 8), onion (*n* = 8), beef (*n* = 5), Mexican chilli (*n* = 9), vegetable (*n* = 1), fruit chutney (*n* = 4), and cheese (*n* = 5)] were analysed for fungal contamination following the spread plate technique and subsequent fungal species isolated were identified by sequencing the ITS4–ITS5 region. Thereafter, fumonisin (FB), aflatoxin (AF) and ochratoxin (OT) producing fungi were confirmed using a PCR and QPCR technique. The fungal load was also counted and expressed as colony forming units per gram of sample (cfu/g). Results obtained showed a total of 29 fungal isolates belonging mainly to the *Aspergillus*, *Penicillium*, *Allophoma* and *Phoma* genera. Sequence analysis of the internal transcribed spacers (ITS) regions of the nuclear encoded rDNA revealed significant alignments for various fungi and in order of incidence, *A*. *niger* (31%) was dominant followed by *A*. *awamori* (14%), *A*. *tubingensis* (14%), *Penicillium* spp. (10%), *A*. *clavatus* (7%), and *Phoma medicaginis* (7%), meanwhile *P*. *commune*, *P*. *crustosum*, *A*. *costaricensis*, *A*. *piperis*, and *Allophoma zantedeschiae* were each recovered at low frequency rate in 3.3% of samples analysed. Fungal contamination levels varied among the spices with the highest fungal load of 109 × 10^1^ cfu/g of sample recorded for ground chilli samples. FB producing fungus (*A*. *niger*) were detected in 61% of similar samples, AF producing fungi (*A*. *flavus* and *A*. *parasiticus*) in 67%, and OT producing fungi (*A*. *niger* and *A*. *tubingensis*) in 23% of the samples under study. With the observation that toxigenic fungi were recovered, it is highly expected that similar samples could be contaminated with associated mycotoxins that may pose health risk to consumers.

### 8.73. Detection of Diverse Fungi Metabolites in Fish Feeds from Nigeria

Olorunfemi M.F. ^1,2,^* and Odebode A.C. ^1^

^1^ Department of Botany, University of Ibadan, Ibadan, Nigeria

^2^ Mycotoxin Unit, Nigerian Stored Products Research Institute, Ibadan, Oyo State, Nigeria

* Correspondence: mfolorunfemi@gmail.com

**Abstract:** Fish feed is an indispensable necessity for fish farming but prone to contamination from diverse range of microorganisms and their metabolites. In this study, fungi metabolites contamination of locally formulated fish feeds from Southwestern Nigeria were determined.

Ninety-four fish feed samples intended for juvenile *Clarias gariepinus* (Cat fish) were randomly collected from warehouses within Southwestern, Nigeria. The spectrum of fungi metabolites including mycotoxins in the feeds was assessed using a Liquid Chromatography-Tandem Mass Spectrometry (LC-MS/MS).

A total of 84 metabolites from diverse fungi (*Aspergillus*, *Fusarium*, *Penicillium*, *Alternaria*, *Clavicep* species and other fungi) were found in the fish feeds. Aflatoxin B_1_, fumonisin B_1,_ zearalenone and deoxynivalenol were detected in 91 (97.9%), 83 (88.3%), 93 (99%) and 82 (87.2%) of the analyzed feeds. Aflatoxin B_1_ was the most prevalent aflatoxin occurring at concentrations ranging from 0.70 μg/kg–550.78 μg/kg with mean of 108.2 μg/kg while fumonisin B_1_ was the most occurring fumonisin found at levels up to 6097.90 μg/kg with mean of1003.5 μg/kg. Total enniatins {A, A1, B, B1}, beauvericin and moniliformin which are new emerging *fusarium* mycotoxins were quantified at high frequency in 94 (100%), 94 (100%) and 91 (96.8%) of samples respectively.

Considering the array and levels of fungi metabolites found in the fish feeds including those with known toxicities, the fish industries in South-western Nigeria may be at risk of economic losses due to mycotoxicoses in the fish species.

**Keywords**: Metabolites; fish feeds; mycotoxicoses; economic losses

### 8.74. Experimental Mould Growth and Mycotoxin Diffusion in Different Food Items

Olsen M. ^1,^*, Gidlund A. ^2^ and Sulyok M. ^3^

^1^ National Food Agency, Department of Risk Benefit Assessment, Uppsala, Sweden

^2^ National Food Agency, Department of Biology, Uppsala, Sweden

^3^ Center for Analytical Chemistry, Department of Agrobiotechnology (IFA-Tulln), University of Natural Resources and Life Sciences, Vienna (BOKU), Tulln, Austria

* Correspondence: monica.olsen@slv.se

**Abstract:** Isolates of *P. commune*, *P. crustosum*, *Penicillium expansum*, *P. roqueforti and Aspergillus versicolor*, were inoculated on different food items (hard cheese, crème fraiche, tomato purée, apple and blueberry jam) and incubated at 15 °C for 14 days at 50% RH. After incubation the food samples were divided into 3 subsamples; A was 0–2 cm from the surface and including the fungal colony, subsample B was 2–4 cm and subsample C was the rest from >4 cm from the surface. The subsamples were analysed with a multianalyte method capable of identifying more than several hundreds of fungal metabolites. The outcome showed that mouldy food can contain a cocktail of bioactive secondary metabolites including mycotoxins and sometimes at high concentrations. Measurements of the diffusion of fungal metabolites from the colony on the surface (layer A) into the food (layer B and C) showed that the fungal metabolites do not diffuse more than 2 cm into the inner core of the hard cheese. On the other hand in more liquid foods, such as crème fraiche, fruit jams and tomato purée, the toxins diffused quite readily throughout the entire food sample. The levels of patulin found in the apple jam indicate that the tolerable daily intake for patulin may easily be exceeded even if the mouldy layer A is removed. This limited study calls for more similar studies to be performed to give risk managers a sound basis for advice to consumers. This work is published in World Mycotoxin Journal [World Mycotoxin Journal, 2017; 10 (2): 153–161] and the presentation of the results here is in agreement with the journal.

### 8.75. Approaches in the Management of Fusarium Head Blight of Wheat in Argentina

Palazzini J.M. ^1,^*, Yerkovich N. ^1^, Palacios S. ^1^, Roncallo P. ^2^, Cantoro R. ^1^, Echenique V. ^2^, Torres A. ^1^, Ramírez M.L. ^1^, Karlovsky P. ^3^ and Chulze S. ^1^

^1^ Department of Microbiology and Immunology, Faculty of Exact Sciences, Physic Chemistry and Natural Sciences National University of Río Cuarto, Río Cuarto, Córdoba, Argentina

^2^ CERZOS-CONICET, Department of Agronomy, UNS–CCT CONICET, Bahía Blanca, Argentina

^3^ Molecular Phytopathology and Mycotoxin Research, Georg-August-University, Goettingen, Germany

* Correspondence: jpalazzini@exa.unrc.edu.ar

**Abstract:**
*Fusarium* Head Blight (FHB) is a devastating disease that causes extensive yield and quality losses to wheat, barley and other small cereal grains in humid and semi-humid regions of the world. Members within the *Fusarium graminearum* species complex are the main pathogens associated with the disease, being *F. graminearum* sensu stricto the main pathogen isolated in Argentina. Chemical treatments, crop rotation, breeding for resistance and tillage practices are among the main strategies to control the disease. The application of antagonists and other chemicals such as chitosan are additional strategies to be used as a part of an integrated pest management. The aims of this study were: to evaluate the control effect of *Bacillus velezensis* RC218, *Streptomyces albidoflavus* RC 87B, chitosan and a combination of them on FHB disease incidence and severity on durum wheat under field conditions; to evaluate the effect of *Bacillus velezensis* RC218 (Bvel), *F. graminearum* (Fgram) and their interaction on the induction of salicylic acid (SA) and jasmonic acid (JA) at different period times under greenhouse conditions ; to carry out a survey of new potential biocontrol agents against *F. graminearum* and the evaluation of them under in vitro and greenhouse conditions. Biocontrol at field level showed effectiveness of the two biological control agents and chitosan in reducing both FHB disease incidence (29.5–63% reduction) and severity (25–50% reduction). Under greenhouse conditions, the phytohormone analysis showed that the production of JA was induced after *F graminearum* inoculation at 48 and 72 h, meanwhile JA levels were reduced in the co-inoculated treatment. No differences in JA or SA induction were observed between the *B. velezensis* treatment and the control. In the spikes inoculated with *F. graminearum*, SA production was early induced (12 h) as it was shown for initial FHB basal resistance; meanwhile the late induction of JA reveals a defense state against the hemibiotrophic pathogen *F. graminearum*. New bacterial strains were isolated from wheat soil samples and evaluated under in vitro conditions through an index of dominance assay as antagonist of *F. graminearum*. A total of eight strains were selected to evaluate the ability to control FHB under greenhouse conditions. Three out of eight strains evaluated were able to significantly reduce FHB severity under greenhouse conditions by up to 63%.

The present work has received funding by the European Union’s Horizon 2020 Research and innovation programme under Grant Agreement No. 678781 (MycoKey).

### 8.76. Alternative Biosensing Strategies for the Detection of Mycotoxins and Mycotoxigenic Fungi

Peltomaa R. ^1^, Benito-Peña E. ^1^, Barderas R. ^2^, Patiño B. ^3^, Sauer U. ^4^, Meucci S. ^5^ and Moreno-Bondi M.C. ^1,^*

^1^ Department of Analytical Chemistry, Complutense University, Madrid, Spain

^2^ Biochemistry and Molecular Biology I Department, Complutense University, Madrid, Spain

^3^ Department of Microbiology III, Complutense University, Madrid, Spain

^4^ Center for Health and Bioresources, AIT Austrian Institute of Technology GmbH, Tulln, Austria

^5^ Micronit Microfluidics BV, Enschede, The Netherlands

* Correspondence: mcmbondi@ucm.es

**Abstract:**
*Fusarium* genus contains many plant-pathogenic fungi, such as *F. verticillioides* and *F. proliferatum*, which are widely distributed in wild and cultivated plant species and produce a range of highly toxic mycotoxins. *Fusarium* contamination can significantly reduce the quality and yield of agricultural products, and moreover, can compromise food safety if mycotoxins enter the food chain. Detection of *Fusarium* is particularly difficult due to the genus diversity and the mold presence at low concentrations the environment. We have developed optical genosensors for the detection of closely related species *F. verticillioides* and *F. proliferatum* based on species-specific oligonucleotide probes designed for the intergenic spacer region of the ribosomal DNA. Magnetic bead -based sandwich hybridization assays allowed detection of the synthetic DNA in low picomolar range and were further applied to the detection of naturally contaminated maize samples. Developed genosensor is integrated on a microfluidic platform to develop a small-footprint tool able to perform the DNA hybridization and detection steps in one device. The platform is fabricated with optically transparent polymer and endowed with an integrated heater which allows temperature control inside the meandering channel.

Apart from monitoring the presence of mycotoxigenic fungi in crops, actual mycotoxin contamination of food must be evaluated in order to meet the regulatory limits set by international authorities. Immunoassays are widely used but often these competitive assays depend on chemical conjugation of the toxin to a carrier molecule. Alternatively, mimotopes, or epitope mimics, have been developed to avoid this cumbersome conjugation step and the toxicity caused by the toxin-conjugate. We have developed of novel mimotopes for the detection of fumonisin B_1_ and T-2 toxin using phage display technology. The mimotopes were selected from a peptide library and used in a competitive phage-based ELISA. Furthermore, for the detection of fumonisin B_1_ the synthetic counterpart of the phage-displayed mimotope was spotted onto a microarray using neutravidin and biotin-linker on the peptide. Microarray-based immunoassay showed improved sensitivity compared to the phage-based ELISA and allowed quantification of fumonisin B_1_ in spiked corn and wheat samples at the levels set by the European legislation. The microarray holds promise for future adaptation to include other mycotoxins for multiplex detection.

### 8.77. Ecophysiology of Fusarium Thapsinum and Fusarium Andiyazi Isolated from Sorghum Grains in Argentina and Mycotoxin Accumulation

Pena G.A. ^1^, Sulyok M. ^2^, Cavaglieri L.R. ^1^ and Chulze S.N. ^1,^*

^1^ Department of Microbiology and Immunology, Faculty of Exact Sciences, Physic Chemistry and Natural Sciences, National University of Río Cuarto, Río Cuarto, Córdoba, Argentina

^2^ Center for Analytical Chemistry, Department of Agrobiotechnology (IFA-Tulln), University of Natural Resources and Life Sciences, Vienna Konrad Lorenzstr. 20, A-3430 Tulln, Austria

* Correspondence: schulze@exa.unrc.edu.ar

**Abstract:** Sorghum (*Sorghum bicolour* L) is the fourth most important summer crop in Argentina, ranking the country second as sorghum exporter in the world. Sorghum grains are used in Argentina for feeding beef and dairy cattle and also for bioethanol production. *Fusarium thapsinum* and *F. andiyazi* are two important pathogens causing grain mold and sorghum stalk rot in sorghum. *Fusarium thapsinum* produces high levels of moniliformin, fusaric acid and trace amounts of fumonisins while *F. andiyazi* produces neither fumonsin nor moniliformin. The knowledge on the range of environmental conditions that allow these species to growth and produce mycotoxins in sorghum grains is relevant to develop future prevention and control strategies. The aim of this study was to determine the effect of interacting conditions of temperature, water activity (a_W_) and incubation time on fungal growth and moniliformin (MON), fusaric acid (FA) and fusarin C production by strains of *F. thapsinum* and *F. andiyazi* isolated from sorghum grains in Argentina. Two strains of *F. thapsinum* (RCFT06 and RCFT08) and one of *F. andiyazi* (RCFA09) were grown on irradiated sorghum grains adjusted to 0.95, 0.98 and 0.99 a_W_ and incubated at 15, 25 and 30 °C during 28 days. The growth rate (mm/day) was determined and toxin production was evaluated at 7, 14, 21 and 28 days by a multi-toxin method based on HPLC-MS/MS. All the interacting conditions were evaluated by triplicate. Maximum growth rates were obtained at the highest a_W_ (0.99 a_W_) and 25 °C for both *F. thapsimun* and *F. andiyazi*, whereas fungal growth decreased significantly as the a_W_ of the grains was reduced (*p* < 0.05). Moniliformin, FA and fusarin C were produced by both *F. thapsinum* RCFT06 and RCFT08 while *F. andiyazi* RCFA09 only produced FA and fusaric C and no MON levels. The levels of mycotoxins produced were dependent on the a_W_, temperature and incubation time. Maximum MON production was observed by *F. thapsinum* RCFT06 and *F. thapsinum* RCFT 08 at 0.99 a_W_ and 30 °C after 28 days of incubation. Also, both *F. thapsinum* strains showed maximum FA and fusarin C production at 0.99 a_W_ and 30 °C but after 14 days of incubation. *Fusarium andiyazi* produced maximum levels of fusarin C at 0.99 a_W_ and 25 °C after 28 days, and maximum levels of FA under the same conditions but after 14 days of incubation. The production of FA by *F. thapsinum* and *F. andiyazi* is important because it may enhance the toxic effects of other mycotoxins such us moniliformin and fusarin C. The environmental conditions for the development of sorghum grains under field conditions could be favorable for toxin accumulation according to the results obtained in the present study.

### 8.78. Good Practices for Preserve Biodiversity of Mycotoxigenic Fungi

Perrone G. ^1^, Frisvad J.C. ^2^, Samson R.A. ^3^, Leslie J.F. ^4^, Waalwijk C. ^5^ and Logrieco A.F. ^1,^*

^1^ Italian National Research Council, Institute of Sciences of Food Production, Bari, Italy

^2^ Department of Biotechnology and Biomedicine, Technical University of Denmark, Lyngby, Denmark

^3^ Westerdijk Fungal Biodiversity Institute, Uppsalalaan 8, NL-3584 CT Utrecht, The Netherlands

^4^ Department of Plant Pathology, Kansas State University, Manhattan, KS 66506, USA

^5^ Plant Research International, Droevendaalsesteeg 1, 6708 PB Wageningen, The Netherlands

* Correspondence: antonio.logrieco@ispa.cnr.it

**Abstract:** In recent years a rising common concern is looking at biodiversity concept with a new sight, attempting to evaluate its economical value, as ground step for supporting measures proposed by national governments and international committees. Although this utilitarian view applied to a complex concept could cause an underestimation of the true potential of biological resources, nowadays a wide spectrum of direct and indirect quantifiable values has been recognized as tightly correlated to biodiversity. Microorganism like fungi play a major role in bio-regulatory systems and could represent an extraordinary source of biodiversity and of new compounds; particular relevance is occupied worldwide by strains belonging to toxigenic genera of *Aspergillus*, *Alternaria*, *Fusarium*, and *Penicillium* representing a great biodiversity for fungal biology. A critical aspect is the quick deposition and right preservation of “wild” toxigenic fungal strains (TFS) in order to avoid potential loss of their metabolic profile, toxigenicity and pathogenicity. In addition, the biochemical profile should be done using the proper media and incubation conditions and confirmed by HPLC and HRMS methods. This is especially important when new records are being provided, or unexpected mycotoxin production by new fungal species. Then, the importance to deposit of “key” TFS in a non-profit Culture Collection by providing a pure monosporic culture. “Key” strain should be clearly identified on basis of ex-type strain, phylogenetic and biochemical uniqueness, genome sequence-data, strains associated to unique or extreme ambient. It is evident that a biological resources, on which public data has been generated, must be available to research community to check when erroneous results are discovered or when new advanced technologies are available for further study and characterization. In this respect, public service culture collections have been performing this function always by providing optimal environment for long-term maintenance and skilled personnel in identification and managing of fungal strains.

Recently, great advance has been done in biodiversity by biochemical and molecular characterization of toxigenic fungi, though data streams from culture collections, research centers, and scientific community are not yet fully integrated with those from biochemical and molecular studies. We wish to stress the importance of using the right approaches in new advance studies on biochemical and molecular characterization of TFS to better understand and preserve the taxonomic diversity and guarantee metabolic properties of the toxigenic fungi community.

### 8.79. Inhibitory Effect of Ozone Against Aspergillus flavus and Penicillium Nordicum Growth

Petrenkova V. ^1^, Taran G. ^2^, Sokol T. ^1^, Pugach S. ^2,^*, Zamuriev A. ^2^ and Opalev P. ^2^

^1^ Plant Production Institute of National Academy of Agrarian Sciences of Ukraine, Kharkiv, Ukraine

^2^ National Science Center “Kharkov Institute of Physics and Technology”, National Academy of Sciences of Ukraine, Kharkiv, Ukraine

* Correspondence: pugach@kipt.kharkov.ua

**Abstract:** Each type of parasitic fungi has its own physiological features and methods of effect on nutritious substrate respectively. The development of such fungi as Aspergillus flavus and Penicillium nordicum (Penicillium nordicum) in wheat and barley grains during their storage leads to deterioration of the product quality, as a result of which such a grain does not meet the requirements when used for food purposes. To achieve the goal the following tasks are included in the research plan: to select the ozone dose and the exposure time of its effect on preventing the growth of mycelium Aspergillus flavus and Penicillium nordicum in pure culture; to select the dose of ozone and the exposure of its effect on limiting the development of infection on artificially contaminated wheat and barley grains. As a result of the studies the inhibitory effect of the ozone-air mixture on vital activity of the fungal mycelium was found. Thus, Aspergillus flavus, grown for three days in nutrient agar medium, completely lost its viability when exposed to the ozone of 0.1 g О_3_/m^3^ for 72 h and 48 h, as at second plating in the nutrient medium the fungal colonies did not germinated. The pure culture of this fungus at the age of seven days was not viable when treated with a dose of 0.04 g О_3_/m^3^ for 72 h and 0.1 g О_3_/m^3^ for 24 h. A positive effect of treatment modes with ozone concentration of 0.04 g О_3_/m^3^ at exposure time of 72 h and 0.1 g О_3_/m^3^ at exposure time of 48 h regarding the degradation of three-day culture Penicillium nordicum was observed and seven-day culture Penicillium nordicum lost its vitality at ozone concentration of 0, 04 g О_3_/m^3^ and 0.1 g О_3_/m^3^ and the same exposure time of 24 h.

Seeds were infected with spores of Aspergillus flavus and Penicillium nordicum and then treated with ozone-air mixture at ozone concentration of 0.1 g О_3_/m^3^ and exposure time of 4, 8, 72 h. A complete inhibiting of fungi development in grains was observed at a mode of 0.1 g О_3_/m^3^ for 72 h. As a result of the phyto-examination of grains treated with the ozone-air mixture and fungi placing into a pure culture no growth of fungi colonies was observed. Thus, the ozone-air mixture has an inhibitory effect on the development of Aspergillus flavus actively growing in a pure culture (three-day) at ozone concentration of 1.0 g О_3_/m^3^ and exposure time of 48 h and on fully grown fungus (seven-day)–0.04 g О_3_/m^3^ at exposure time of 72 h. An inhibitory effect on the development of Penicillium nordicum actively growing in a pure culture was observed when treated with a dose of 0.04 g О_3_/m^3^ at exposure time of 72 h and 0.1 g О_3_/m^3^ at exposure time of 48 h. An inhibitory effect on the development of fully grown Aspergillus flavus was observed when treated with doses of 0.04 g О_3_/m^3^ and 0.1 g О_3_/m^3^ at exposure time of 24 h. Elimination of fungi viability has been achieved during a treatment at ozone concentration of 0.1 g О_3_/m^3^ and exposure time of 72 h.

### 8.80. Fluorescence Polarization Immunoassays for the Determination of Fusarium Toxins

Porricelli A.P.R. ^1^, Pascale M. ^1^, Cortese M. ^1^, De Saeger S. ^2^, Li P. ^3^, Logrieco A.F. ^1^ and Lippolis V. ^1,^*

^1^ Institute of Sciences of Food Production, National Research Council of Italy, Bari, Italy

^2^ Laboratory of Food Analysis, Faculty of Pharmaceutical Sciences, Ghent University, Ghent, Belgium

^3^ Key Lab for Mycotoxins Detection, Oil Crops Research Institute of the Chinese Academy of Agricultural Sciences, Wuhan, China

* Correspondence: vincenzo.lippolis@ispa.cnr.it

**Abstract:**
*Fusarium* toxins, a group of mycotoxins, can be produced by *Fusarium* fungi under temperate climatic conditions on agricultural commodities, mainly cereals, in field as well as during storage. As a defensive response of the host plant, *Fusarium* toxins can be metabolized by forming modified mycotoxins, often called “masked” mycotoxins. It has been shown that many modified forms are hydrolysed into the parent mycotoxin during digestion. In order to protect consumer health from the risk of exposure to modified and parent forms of *Fusarium* toxins, the development of rapid, sensitive and reliable methods for their simultaneous determination in cereals is highly demanded. Currently, fluorescence polarization immunoassay (FPIA) is getting the attention as a screening tool in food safety control due to its simplicity, rapidity, cheapness and reliability. The focus of our work is to develop and validate quantitative FPIAs for simultaneous determination of DON and its acetylated (3-acetyl-DON, 15-acetyl-DON) and glycosylated forms (DON-3-glucoside) and T-2/HT-2 toxins and their glycosylated forms (T2-glucoside, HT2-glucoside) in wheat. A fluorescein-label (tracer) of DON (DON-FL) and four T2- and HT2-fluerescein tracers (T2-FL, HT2-FL_1a_, HT2-FL_1b_ and HT2-FL_2_) were synthesized and purified. The assessment of the antibody-tracer binding was performed using four DON monoclonal antibodies (MAbs), and ten T2-glucoside MAbs, one HT-2 MAb and two T-2 MAbs, at different concentrations. Concerning the FPIA for the determination of DON, its acetylated and glycosylated forms, the highest antibody-tracer binding was observed for the clone 22/DON-FL combination, while in the FPIA for the determination of T-2/HT-2 toxins and their glycosylated forms, the highest bindings were observed for thirteen T2-glucoside MAbs with T2-FL and HT2-FL_1b_ combinations, as well as for HT-2 MAb/HT2-FL_1a_ combination and for T-2 MAb/T2-FL and HT2-FL_1a_ combinations. Competitive FPIAs were performed with the selected antibody combinations. In particular, FPIA for the determination of DON, its acetylated and glycosylated forms showed IC_50_=23.5 ng/mL for DON and exhibited 204%, 45% and 11% as cross-reactivity, respectively, for 3-acetyl-DON, DON-3-glucoside and 15-acetyl-DON. While, among the selected combinations for T-2 and HT-2, the HT-2/HT2-FL_1a_ combination exhibited 80% as cross-reactivity for T-2 and its glycosylated form and the highest sensitivity, with IC_50_ = 2.0, 2.6 and 2.7 ng/mL for HT2, T-2 and T2-glucoside, respectively. These findings showed the applicability of the developed FPIAs to the determination of parent and modified mycotoxins, expressed as sum, in solution.

### 8.81. Isolation and Identification of Fungi from Spices Consumed in Military Collective Restoration in Tunisia

Saidi R. ^1,^*, Rhaiem B.M. ^1^, Jemili B. ^2^ and Gargouri S. ^2^

^1^ Military laboratory for food analysis, General direction for military health, Department of National Defense, Tunisia

^2^ Research Unit on Molecular Epidemiology of Invasive and Nosocomial Mycoses, service of parasitology, Tunis Main military Instruction Hospital, Tunisia

* Correspondence: raedsaidi@yahoo.com

**Abstract:** Spices are natural products obtained from parts of certain plants, widely used in food preparation. They may be exposed to a wide range of microbial and fungal contamination. Molds reduce the quality of spices, and create a potential risk for human health with the production of toxic metabolites known as mycotoxins. The objective of this study was, to examine the mold profile of spices consumed in collective military restoration in Tunisia, to isolate and identify Aspergillus strains, potential producers of mycotoxins, to highlight their risk assessment.

A total of 213 dried and ground samples, representing five different types of spices (Curcuma, paprika, coriander, caraway and cumin), in packaged and loose form, were collected between 2014 and 2016 from different military collective restoration covering the entire territory of Tunisia. All samples were collected in sterilized polyethylene bags, with an average size ranged between 250 g and 1 kg, and taken to the Laboratory. Dilution method according to the standard NFV 08 .059 May 2003, with Sabouraud Chloramphenicol agar medium was used to determine total fungal counts, in duplicates. The fungal isolates were transferred to sterilized plates for purification and identification according to morphological and microscopic characteristics. All analyzed samples and isolated Aspergillus strains were conserved for further analysis. SPSS software was used for data analysis.

Fungal contamination of spices showed a large variation, depending on their types and forms. Contamination was too high in paprika with a level between 104 and 105 UFC/g. Three fungal genera were identified: *Mucor* spp. (54%), *Aspergillus* spp. (35%), and *Penicillium* spp. (1%). A total of 95 strains of five *Aspergillus* species were identified with the dominance of: *A.niger* (61%) and *A.flavus* (30%), which is in agreement with the results found by Boukhari et al. (2007). Contamination by *Aspergillus* was similar in both packaged samples (33%) and loose samples (35%). No significant correlation between *Aspergillus* contaminations and the geographic origins of samples: southern sector (40%), middle sector (32%), northern sector (37.5%).

*A. niger* and *A. flavus* were found in 36% of spices samples analyzed. Those two species are known as producers of mycotoxins. Following these results, a further study will be carried out on the research and quantification of Aflatoxin B1 and Ochratoxin A in all samples studied.

### 8.82. Deoxynivalenol and Fumonisins during the Production of Wheat Bread and Cornflakes

Schaarschmidt S. *, Lahrssen-Wiederholdt M. and Fauhl-Hassek C.

Department Safety in the Food Chain, German Federal Institute for Risk Assessment (BfR), Berlin, Germany

* Correspondence: sara.schaarschmidt@bfr.bund.de

**Abstract:** The trichothecene deoxynivalenol (DON) and the fumonisins B1 and B2 (FB1 and FB2) are common *Fusarium* mycotoxins in wheat and maize, respectively. To protect consumers’ health, DON and FB1 + FB2 maximum levels (MLs) are set by the European Union (EU) food law [1] for cereal raw materials and different products, with lower MLs for the final food products. All EU mycotoxin MLs are defined on the product “as is” basis.

In the present study, data collected from literature regarding the fate of mycotoxins during cereal processing were compared with the legal obligations in the EU. Thus, dilution or concentration effects were taken into account to estimate the underlying processing factors on an “as is” basis. Processing factors for DON and FB1/FB2 were estimated for the production of wheat bread and of cornflakes, respectively. In doing so, the effect of cleaning (which strongly depends on the quality of a batch) was not considered, because the EU MLs for unprocessed cereals also apply to cleaned grains. However, this might lead to some underestimation of the effect of processing since typically cleaning operations are performed in a milling plant before processing of grains (even if a preliminary cleaning had already taken place).

Regarding the production of white bread, changes in DON contaminations during milling (fractionation) and baking appear to be sufficient to comply with legal obligations. But meeting the legal limit is more challenging for wholemeal bread as well as for bakery products characterized by low moisture and high cereal content—particularly when they contain wholemeal and/or bran. Here, raw materials of higher quality, improved technological processes, and/or monitoring of mycotoxins would be helpful to overcome compliance issues.

Cornflakes can be produced either via extrusion cooking of maize flour or by traditional pressure cooking of maize flaking grits. Milling to maize flour might not always meet the legal requirements, despite a higher FB1+FB2 ML for maize milling fractions with lower particle size (≤500 μm). The reduction during secondary processing of the maize flour appears as sufficient to meet the limit and might (partly) also compensate an insufficient reduction during primary processing. For grit-based cornflakes, the estimated processing factors were always lower than the changes in the FB1 + FB2 MLs.
Commission Regulation (EC) No. 1881/2006 of 19 December 2006 setting maximum levels for certain contaminants in foodstuffs. *Off. J. Eur. Commun. L*
**2006**, *364*, 5–24. Consolidated version of 1 April 2016 including amendments.

The work was done in preparation for tasks of the MyToolBox project. This project has received funding from the European Union’s Horizon 2020 research and innovation programme under grant agreement No. 678012.

### 8.83. Impact of Fusarium Mycotoxins on the In Vitro Activity of Six Major Cytochrome P450 Enzymes in Porcine Hepatic Microsomes

Schelstraete W. *, Devreese M. and Croubels S. *

Laboratory of Pharmacology and Toxicology, Department of Pharmacology, Toxicology and Biochemistry, Faculty of Veterinary Medicine, Ghent University, Salisburylaan 133, 9820 Merelbeke, Belgium

* Corresponding authors: wim.schelstraete@ugent.be; siska.croubels@ugent.be

**Abstract:** Cytochrome P450 enzymes (CYP450) are catalytic oxido-reductases capable of metabolizing a wide variety of endogenous and xenobiotic compounds. A principal function of these CYP450 is to improve elimination of such substances by biotransformation to more polar and water soluble metabolites. However, some xenobiotics can inhibit or induce CYP450 activity, and co-ingestion of these compounds with substrate drugs can lead to an altered disposition of these substrate drugs. This has been associated with a number of clinically relevant drug-drug or drug-food interactions. Nonetheless, regarding drug-food contaminant interactions, literature reports are scarce. Mycotoxins are highly prevalent food and feed contaminants produced by several fungal species. Pigs are very sensitive to the toxic effects of mycotoxins, in particular deoxynivalenol (DON) and zearalenone (ZEA). Moreover, the inhibitory impact of T-2 toxin (T-2) on the hepatic CYP3A activity in pigs was already demonstrated. In addition, the similarities between porcine and human CYP450 enzymes suggest that the pig can serve as a suitable animal model for drug metabolism and safety studies in humans.

The aim of the current study was to investigate the impact of four principal *Fusarium* mycotoxins (DON, ZEA, T-2 and fumonisin B1) on the in vitro activity of six major porcine CYP450 enzymes (CYP1A, CYP2A, CYP3A, CYP2C, CYP2D and CYP2E). A screening of the mycotoxins’ inhibition potential was performed by incubating each of the four mycotoxins with selected probe substrates for each of the six CYP450 enzymes (phenacetin, coumarin, midazolam, tolbutamide, dextromethorphan and chlorzoxazone for CYP1A, CYP2A, CYP3A, CYP2C, CYP2D and CYP2E, respectively). From this screening assay, mycotoxins showing an enzyme activity inhibition of more than 20% were selected for the establishment of full inhibition profiles. These profiles allowed the determination of the type of inhibition by nonlinear regression methods and statistical evaluation of the selected model. Inhibitory constants (Ki) were calculated as recommended by the EMA. The results will be presented at the conference. They contribute to the insights, and knowledge of the impact of mycotoxins on human and veterinary drug metabolism, pharmacokinetics and ultimately on clinical efficacy.

### 8.84. Occurrence of Mycotoxins in Feed Produced in Russia and Analyzed by Multi-Mycotoxin Lc-Ms/Ms Method

Selimov R. *, Metalnikov P., Komarov A. and Batov I.

The Russian State Center for Quality and Standardization of Veterinary Drugs and Feed (“VGNKI”), 5 Zvenigorodskoye highway, Moscow, Russia

* Correspondence: renatselimov@yandex.ru

**Abstract:** The study was aimed to determine the occurrence of wide range of mycotoxins in feed produced in Russia. The survey included regulated mycotoxins but was also targeted to achieve data on emerging mycotoxins providing that previous data on occurrence of emerging toxins in Russia was low. A multi-mycotoxin LC-MS/MS method for simultaneous determination of 50 mycotoxins was applied for analysis of 123 samples of grain-based animal feed including combined feeds and feed ingredients. The method was based on “dilute and shoot” approach because no appropriate clean-up technique was available for such a broad spectrum of toxins. Samples were extracted using mixture of acetonitrile/water/acetic acid (79/20/1 respectively) and extracts analyzed by LC-MS/MS in two separate injections, for positive and negative mode of ionization. Mycotoxins were determined in 88 samples (72%). Twenty six percent of samples were simultaneously contaminated by mycotoxins at numbers ranging from 4 to 12. Among regulated mycotoxins T-2 toxin (mean concentration 73 μg/kg), zearalenon (mean concentration 11 μg/kg) and fumonisins (mean concentration of sum of fumonisin B1 and fumonisin B2 was 1074 μg/kg) were determined with the most frequency. Among non-regulated mycotoxins the Alternaria toxins (alternariol, alternariol methylether, tentoxin, tenuazonic acid) were detected in 50% samples and showed the highest prevalence. Other frequently detected mycotoxins were HT-2 toxin, beauvericin, mycophenolic acid and moniliformin. Relying on the obtained data the mycotoxin profiles for different types of grain-based animal feed produced in Russia were determined. The presented study was an initial stage of long-term monitoring program aimed to assess risks associated with emerging mycotoxins in Russia.

### 8.85. Aflatoxin Reduction in Maize by Advanced Grain Cleaning Solutions

Slettengren K. ^1,^*, Hirschberger M. ^1^, Graeber M. ^1^, Reichel M. ^2^ and Pascale M. ^3^

^1^ Bühler AG, Uzwil, Switzerland

^2^ Eurofins, Germany

^3^ Institute of Sciences of Food Production, National Research Council (ISPA-CNR), Bari, Italy

* Correspondence: katarina.slettengren@buhlergroup.com

**Abstract:** Grain cleaning is the most effective post-harvest measure to reduce elevated mycotoxin levels. The focus lies on the efficient removal of mould-infested grains and grain fractions on the basis of features such as size, density and optical properties. In this study, the reduction of total aflatoxins (AFB_1_, AFB_2_, AFG_1_, AFG_2_) in naturally contaminated maize was tested in three cleaning steps using industrial-scale cleaning Bühler machines. The first step included (i) the Grain Plus for mechanical size separation and dust removal by aspiration, the second (ii) separation based on density differences with a Concentrator, and the third (iii) optical sorting with SORTEX. Four batches of maize (about 3 tons each) with different levels of aflatoxin contamination were used for the trials. Furthermore, different process settings and cleaning intensities were tested. Sampling (3 replicates/batch) was performed according to the Commission Regulation N. 401/2006 and the collected samples were analyzed by HPLC/FLD with photochemical derivatization. In addition, the incoming material was analyzed by the Eurofins’ Rapidust^®^ system for on-site sampling and analysis of mycotoxins in grains. First of all, trials once again highlighted the difficulties of sampling for aflatoxins. Samples showed a large variability with respect to aflatoxin level. However, high levels of aflatoxin contamination were observed in the removed product streams, with values up to 250 μg/kg. Consistent results were achieved by calculating the aflatoxin level of the incoming material from the removed products taking into account the mass balance. These values compared well with the analyzed levels from the Rapidust^®^.

Aflatoxin levels were reduced from about 10 and 20 μg/kg to 3–4 μg/kg and 2–3 μg/kg for the low and high contaminated material, respectively. The results showed that (i) with the Grain Plus an aflatoxin removal of 10–15% was achieved by size separation and aspiration; (ii) with the Concentrator, another 10–70% aflatoxins was removed by density separation and finally, (iii) another 20–90% aflatoxins was removed by optical sorting with SORTEX. In conclusion, the combination of the tested cleaning machines could allow a total aflatoxin removal of 60–90% in maize.

This work was supported by the MYCOKEY project which has received funding from the European Union’s Horizon 2020 research and innovation programme under Grant Agreement No. 678781.

### 8.86. MAIZE Survey on AFLATOXIN Contamination in ROMANIA

Smeu I. *, Cucu E.M. and Dobbre A.A.

Microbiology-ELISA Laboratory, National R&D Institute for Food Bioresources IBA-Bucharest, Bucharest, Romania

* Correspondence: irina.smeu@bioresurse.ro

**Abstract:** Mycotoxin contamination represents a clear public health concern. In this context, 168 maize samples from all around Romania, along with information regarding the specific fields’ location and the applied agronomic practices were collected and investigated regarding the incidence of total aflatoxins (AFLA). 14 samples, from the southern, southeastern and southwestern regions registered AFLA levels higher than the limit of 1000 ppb, settled by the Commission Regulation (EC) No. 1881/2006 for maize to be subjected to soring or other physical treatment before human consumption or use as an ingredient in foodstuffs. The highest AFLA level was 39,756 ppb, noted by a maize sample received from the southern part of Romania (Argeș county). When referring to the analyzed samples, the mycotoxin contamination was independent of the type of hybrid, but strongly influenced by the differences of pedo-climatic conditions between counties. The southern counties proved to represent critical risk areas for aflatoxin contamination on maize crops.

### 8.87. Deoxynivalenol and T-2 Toxin as Major Concern in Durum Wheat from Italy

Somma S., Haidukowski M., Ghionna V., Masiello M., Cimmarusti M.T., Logrieco A.F. and Moretti A. *

Institute of Sciences of Food Production, National Research Council (ISPA-CNR), Bari, Italy

* Correspondence: antonio.moretti@ispa.cnr.it

**Abstract:** One of the most devastating disease of wheat is represented by Fusarium Head Blight (FHB), caused by a complex of *Fusarium* species. Most of them are able to produce a wide range of mycotoxins, (trichothecenes above all) that can be accumulated in wheat kernels at maturity. The *Fusarium* species occurrence is variable in different geographical areas, according to the different environmental conditions and is subjected to a continuous evolution in distribution, due to climate change.

About 140 durum wheat field samples were randomly collected in different regions of Italy (18, 70 and 53 samples from Northern, Central and Southern Italy, respectively) in three consecutive years (2013–2015) and analyzed for *Fusarium* species and their related mycotoxin occurrence. Representative amounts of grounded wheat kernels were chemically analyzed for deoxynivalenol (DON), nivalenol (NIV), zearalenone (ZEA), T-2 and HT-2 toxin detection. Mycotoxin contamination varied according to year of sampling and geographical areas. The highest mycotoxin contamination was observed in 2014 sampling year, while in 2015, which was characterized by little rain, very low levels of all the analyzed mycotoxins were detected. Deoxynivalenol was detected at relevant levels (average 240 μg/kg) only in samples collected in Central and Northern Italy, while T-2 and HT-2 toxins occurred at higher levels (up to 460 μg/kg) in samples collected in Southern Italy. Surprisingly, about 80% (35 out of 42) of wheat samples from Southern Italy in 2013 and 2014 sampling years showed T-2 and HT-2 toxins levels over the European Union recommended limits for unprocessed wheat (100 μg/kg). The identification of *Fusarium* species isolated from wheat kernels reflected the mycotoxin occurrence. A higher incidence of *F. graminearum sensu stricto* was observed in wheat samples from Northern Italy, mostly contaminated by DON while *F. langsethiae* was frequently isolated from wheat samples from Southern Italy, mostly contaminated by T-2 and HT-2 toxins. These data showed that a real mycotoxin risk related to *Fusarium* mycotoxins does exist along the whole Italy, but they vary according with the geographical areas and year of sampling.

A monitoring of *Fusarium* species distribution and related mycotoxin occurrence should be required in each Country for several years to observe the starting situation and its evolution in order to identify new potential risks for wheat products.

### 8.88. MycoKey: Importance of Global Network of Mycotoxigenic Fungi

Susca A. ^1,^*, Perrone G. ^1^, Moretti A. ^1^, Waalwijk C. ^2^, Audenaert K. ^3^, Mule G. ^1^, Hao Z. ^4^ and Logrieco A.F. ^1^

^1^ Italian National Research Council, Institute of Sciences of Food Production, Bari, Italy

^2^ Business unit Biointeractions and Plant Health, Wageningen Plant Research, Wageningen, The Netherlands

^3^ Laboratory of Applied Mycology and Phenomics, Department of Applied Biosciences, Faculty of BioscienceEngineering, Ghent University, Ghent, Belgium

^4^ Institute of Plant Protection, Chinese Academy of Agricultural Sciences, Beijing, P. R. China

* Correspondence: antonella.susca@ispa.cnr.it

**Abstract:** Living biomass on the planet is represented for 50% by microorganisms that provide an important source of genetic information for both molecular biology and biotechnology. Fungi play a major bio-regulatory role in natural ecosystems and represent an extraordinary source of new compoundsof great ecological relevance. In particular, the toxigenic fungi (TF) produce a large series of secondary metabolites, that may accumulate in final products of agro-food plants. These compounds possess a wide range of biological activities with a high impact on plant, human and animal health.An important category of these specialised metabolites are formed by mycotoxins, due to the detrimental effect on other organisms, including humans and animals. Therefore, incorrect identification of TF will have negative consequences on the accurate evaluation ofexposure risk for the consumption of contaminated food.

Currently, many studies on the characterization of TF at genetic and biochemical level generate a huge amount of oftenunrelated and not well organized data. On the other hand, the scientific community can take advantage from both a more rational organization of such data and extensive sharing of the organisms that produce these compounds. To further progress of the general knowledge on TF, fundamental steps are needed including reduction of overlaps and optimization of the efforts at global level. To facilitate merging of information and preserve natural biodiversity, important objects should be pursued such as: (i) identification and characterization of TFs using a standard and polyphasic approach; (ii) organization and sharing of data; (iii) deposition of strains in well recognized Culture Collections.

The Horizon 2020 EU project MycoKey (Grant 678781) aims to reduce mycotoxin contamination in food and feed crops. Among the activities in the project, great attention is made on the careful deposition of toxigenic fungi and the harmonization of relevant information related to TFsand (changes in) their global occurrence. Datasets include DNA sequences, secondary metabolites profiles, and metadata on their geographic occurrence and ecological niches. Sharing knowledge and biological materials will ultimately provide an effective contribution to mycotoxin risk management.

### 8.89. Consumer Advice on Handling of Mouldy Foods

Svanström A. * and Olsen M.

National Food Agency, Department of Risk Benefit Assessment, PO Box 622, SE-751 26 Uppsala Sweden

* Correspondence: asa.svanstrom@slv.se

**Abstract:** This report summarizes the scientific basis underlying the Swedish National Food Agency’s advice to consumers on how to handle mouldy foodstuffs, e.g., bread, fruit and dairy, to avoid mycotoxins. The report is part of a larger work where the advice for consumers, presented on NFAs webpage for instance, are reviewed and updated. To investigate whether there is scientific support for the soundness of various risk mitigation measures, such as cutting off the moulded parts of the affected food and eating the rest, a major literature review was conducted.

Many potent toxin producing moulds have been isolated from bread and for instance aflatoxin can be produced in very high levels. It has also shown that mycotoxins easily spread in bread, contaminating also unaffected parts. In fresh fruit, spreading of toxins like ochratoxin A and patulin have been studied, showing that and that unaffected parts of the fruit may contain high levels of toxins. In pears for instance, about 1000 μg/kg of patulin have been measured in the fresh area of the fruit, on the opposite side of the affected parts (Sulyok et al., 2010). There is likely a correlation between how far the toxin diffuses and how much water the fruit contains. Properties of different toxins, such as size and polarity, may also affect how they are spread in foods. The bulk of data that can be found on the development of mycotoxins in moulding foods was generated during the 60 s, 70 s and 80 s and is largely based on few observations. There is therefore considerable uncertainty whether different risk reducing strategies can lower exposure of mycotoxins to an acceptable level.

Due to the lack of sound sscientific evidence and the principle that “safe is better than sorry” the advice for consumer should be to discard foods that are mouldy. Other means of minimizing the risk of mycotoxins does not seem to be sufficiently well-substantiated in the literature. In order to reduce food waste, and at the same time minimize the risks of mycotoxins, food should be stored in a way that prevents it from going mouldy, e.g., freezing.

More studies on the formation and distribution of mycotoxins in foods are needed. Results from such studies could be used to model growth and mycotoxin production, which would provide a better understanding of the problem. There is also a need to investigate how our handling of food that has gone mouldy at the consumer level can impact the exposure of mycotoxins and also for behavioral studies to clarify what people in general do with mouldy foods.

### 8.90. Multi-Mycotoxin Survey for Corn and Finished Feed 2016

Taschl I. *, Jenkins T. and Kovalsky P.

BIOMIN Holding GmbH, Erber Campus 1, 3131 Getzersdorf, Austria

* Correspondence: ines.taschl@biomin.net

**Abstract:** Mycotoxins are a large family of toxic fungal metabolites which occur worldwide in various cereals and other feed commodities. During the whole chain from field to feeding, mycotoxins can be produced by fungi in plant material. Their presence poses a risk to animal production. The occurrence and levels of a wide range of fungal secondary metabolites including established and emerging mycotoxins was assessed in the world supply of corn and finished feed.

To test the occurrence of multiple mycotoxin metabolites, a method based on Liquid Chromatography coupled with tandem Mass Spectrometry was used (LC-MS/MS method, Spectrum 380^®^). The method assesses the levels of in addition to the major regulated and guideline mycotoxins aflatoxins (Afla), zearalenone (ZEN), deoxynivalenol (DON), fumonisins (FUM), T-2 toxin (T-2) and ochratoxin A (OTA). In 2016, a total number of 323 corn and 433 finished feed samples were analyzed from over 40 countries around the world.

Among the most common mycotoxin metabolites found in finished feed samples was the *Penicillium* toxin brevinamid F, detected in 94% of all samples at an average of 81 ppb. Also *Aspergillus* toxins were detected in a high amount: asperglaucide occurred in 93% and tryptophol in 89% of all finished feed samples. The *Fusarium* mycoestrogen ZEN (a cause of reproductive issues in livestock) was present in 88% of all tested finished feed samples. In total, 74% of all finished feed samples contained between 20 and 50 fungal secondary metabolites. Considering corn samples, the emerging *Fusarium* mycotoxin moniliformin (94% occurrence) and the major mycotoxin fumonisin B1 (86% occurrence) represented the most common toxins. The Type B trichothecenes DON and nivalenol (both *Fusarium* mycotoxins) were detected in 41% and 40% of all corn samples at an average of 558 ppb and 85 ppb, respectively. Most corn samples (61%) contained between 20 and 40 different fungal secondary metabolites. OTA was the least prevalent major mycotoxin; detected in less than 1% of all tested corn samples.

The multi-mycotoxin LC-MS/MS method offers more detailed results than common testing methods. While some of the major *Fusarium* mycotoxins were frequently detected, the high prevalence of many emerging mycotoxins and other fungal secondary metabolites in corn and finished feed highlights the requirement for more research on the effect of these further compounds on animal productivity and health.

### 8.91. Relative Matrix Effects in LC-ESI-MS/MS Based Mycotoxin Determination and Their Contribution to the Measurement Uncertainty

Stadler D., Krska R. and Sulyok M. *

University of Natural Resources and Life Sciences, Vienna (BOKU), Department of Agrobiotechnology (IFA-Tulln), Center for Analytical Chemistry, Konrad Lorenz Str. 20, 3430 Tulln, Austria

* Correspondence: michael.sulyok@boku.ac.at

**Abstract:** In the recent years, the LC-ESI-MS/MS based multi-analyte approach has been demonstrated to be a powerful technique for the simultaneous determination of mycotoxins in food and feed [1]. One significant drawback of the ESI source is its high susceptibility to matrix effects. To obtain a robust LC-ESI-MS assay, matrix effects have to be minimized or compensated. For the presented method, sample clean-up needs to be kept at a minimum due to the chemical diversity of the targeted analytes. The use of stable isotopically labelled internal standards is not feasible because of limited availability and high costs. Therefore, the quantification of mycotoxins is increasingly based on the analysis of diluted crude extracts and external- or matrix-matched calibration.

In everyday practice the calibration curve is constructed from a single lot of a matrix. However, the degree of ion suppression for an analyte may vary in different lots of the same matrix, which is referred to as relative matrix effect. This effect has already been addressed in quantitative bioanalysis in the course of drug development, but remains unstudied for multi-analyte methods and unmentioned in the official guidelines [2].

In order to quantify relative matrix effects (RSD_SSE_), signal suppression/enhancement values (SSE) were obtained for seven *different lots of the same matrix* for 70 analytes in 7 matrixes. Relative matrix effects were calculated as the relative standard deviation (RSD) of the SSE values. Depending on the matrix, 80 to 100% of the investigated analytes were free of relative matrix effects (RSD_SSE_ < 15%). For analyte-matrix combinations with RSD_SSE_ > 15%, the influence of relative matrix effects on the measurement uncertainty was determined. Our findings highlight the need to consider relative matrix effects during initial method validation and in the official guidelines for LC-ESI-MS/MS based determination of mycotoxins.
